# A Mendelian analysis of the relationships between immune cells and breast cancer

**DOI:** 10.3389/fonc.2024.1341292

**Published:** 2024-01-24

**Authors:** Xin Wang, Haoyu Gao, Yiyao Zeng, Jie Chen

**Affiliations:** ^1^ Division of Breast Surgery, Department of General Surgery, West China Hospital, Sichuan University, Chengdu, China; ^2^ Breast Center, West China Hospital, Sichuan University, Chengdu, China; ^3^ Division of Cardiovascular Surgery, Department of General Surgery, West China Hospital, Sichuan University, Chengdu, China; ^4^ Department of Cardiology, Dushu Lake Hospital Affiliated to Soochow University, Medical Center of Soochow University, Suzhou Dushu Lake Hospital, Suzhou, Jiangsu, China

**Keywords:** immune cells, breast cancer, genetic approaches, Mendelian randomization, analysis

## Abstract

**Background:**

Emerging evidence showed immune cells were associated with the development of breast cancer. Nonetheless, the causal link between them remains uncertain. Consequently, the objective of this study was to investigate the causal connection between immune traits and the likelihood of developing breast cancer.

**Methods:**

A two-sample Mendelian randomization (MR) analysis was conducted to establish the causal relationship between immune cells and breast cancer in this study. Utilizing publicly accessible genetic data, we investigated causal connections between 731 immune cells and the occurrence of breast cancer. The primary approach for exploring this relationship was the application of the inverse-variance-weighted (IVW) method. Furthermore, sensitivity analyses, encompassing the leave-one-out analysis, Cochran Q test, and Egger intercept test were performed to validate the reliability of the Mendelian randomization results. Finally, we used Bayesian Weighted Mendelian Randomization (BWMR) approach to test the results of MR study.

**Results:**

According to the Bonferroni correction, no immune trait was identified with a decreased or increased risk of overall breast cancer risk. As for the ER+ breast cancer, 6 immune trait was identified after the Bonferroni method. the IVW method results showed that CD45RA- CD4+ %CD4+ (p-value:1.37×10^−6^), CD8dim %T cell (p-value:4.62×10^−43^), BAFF-R on IgD+ CD38- unsw mem (p-value:6.93×10^−5^), CD27 on PB/PC (p-value:2.72×10^−18^) lowered the risk of breast cancer. However, CD19 on IgD- CD38br (p-value:1.64×10^−6^), CD25 on IgD+ CD38dim (p-value: - ∞) were associated with a higher risk of developing breast cancer. As for the CX3CR1 on CD14+ CD16- monocyte (p-value: 1.15×10^−166^), the IVW method clearly demonstrated a protective effect against ER- breast cancer. For the above positive results, BAFF-R on IgD+ CD38- unsw mem was the sole association linked to reduced breast cancer risk using the BWMR method. The intercept terms’ p-values in MR-Egger regression all exceeded 0.05, indicating the absence of potential horizontal pleiotropy.

**Conclusion:**

Through genetic approaches, our study has illustrated the distinct correlation between immune cells and breast cancer, potentially paving the way for earlier diagnosis and more efficient treatment alternatives.

## Introduction

Cancer has become a major problem worldwide, and despite medical advances, it remains the second leading cause of death. A study has suggested that the worldwide cancer burden is anticipated to increase by nearly 50% over the next two decades (1). As per the Cancer Statistics report for 2022, breast cancer makes up nearly one-third of diagnoses in women and prostate cancer constitutes 27% of male diagnoses (2). Considering the significant menace cancer poses to human health, early cancer screening and prevention hold paramount significance.

Immune cells increase the likelihood of cancer development and support all phases of tumorigenesis. Cancer cells, along with neighboring stromal and inflammatory cells, participate in coordinated interactions that lead to the creation of an inflamed tumor microenvironment (TME). Cells within the TME exhibit a high degree of flexibility, consistently altering their phenotypic and functional traits. Tumor-associated macrophages (TAMs) play a crucial role in the tumor microenvironment. High levels of TAM infiltration have been associated with poor prognosis in breast cancer. The balance between M1 (anti-tumor) and M2 (pro-tumor) macrophages is crucial in determining the impact on tumor progression (3). HER2-positive breast cancers often show distinct immune profiles. Studies have explored the interaction between HER2 status, TILs, and response to HER2-targeted therapies (4). In addition, Gaynor J Bates et al. explored the quantification of regulatory T cells in breast cancer patients and identified an association between increased Treg infiltration and high-risk disease and late relapse. Tregs were implicated in promoting immune evasion. Bell D, et al. examined the distribution of immature and mature dendritic cells in breast carcinoma tissue. Understanding the localization and function of dendritic cells contributes to our knowledge of antigen presentation and immune responses in breast cancer (5). But some immune cells help suppress the development of tumor cells. Tumor-infiltrating lymphocytes (TILs), particularly in triple-negative breast cancer (TNBC), have been associated with better prognosis. Higher levels of TILs have been linked to improved overall survival and response to certain therapies (6). These references provide a starting point for exploring the intricate relationship between immunophenotypes and breast cancer development or progression. It’s important to note that the field is dynamic, and ongoing research continues to refine our understanding of the immune landscape in breast cancer. Always refer to the latest literature for the most up-to-date information.

Mendelian randomization (MR) is a primarily employed analytical technique in epidemiological investigations for inferring causality. It is crucial that the causal inference derived from MR is logically sound and substantiated (7, 8). This study conducted a thorough two-sample Mendelian randomization (MR) analysis to establish the causal link between immune cell signatures and breast cancer. Gaining insights into the risk factors linked to the progression of breast cancer will contribute to the development of innovative treatments for this aspect.

## Method

### Data sources for exposure data

We conducted an evaluation of the causal connection between 731 immune cell signatures and breast cancer using a Mendelian randomization analysis. The 731 immunophenotypes consist of median fluorescence intensities (MFI) (n=389), absolute cell (AC) counts (n=118), relative cell (RC) counts (n=192) and morphological parameters (MP) (n=32). The first three types include myeloid cells, B cells, mature stages of T cells, monocytes, TBNK (T cells, B cells, natural killer cells), CDCs and Treg panels, while the latter type comprises CDCs and TBNK panels. For the exposure instrument, we employed the summary statistics from a recent extensive genome-wide association study (GWAS) conducted on blood cell traits by the Blood Cell Consortium (BCX). This GWAS encompassed a vast cohort of 563,085 individuals of European descent (9). Around 22 million SNPs, genotyped using high-density arrays, underwent the process of imputation using a reference panel derived from Sardinian sequences (10). The associations were assessed while accounting for covariates, including sex, age, and age squared.

### Data sources for outcome data

The overall breast cancer data (including 15680 cases and 167189 controls) used in this study were derived from the FinnGen database (https://finngen.gitbook.io/documentation/), with the current version (release 11, data release date: May 8, 2023). In addition, the 69,501 ER-positive (ER+) breast cases and 21,468 ER-negative (ER-) breast cases were from the Breast Cancer Association Consortium (BCAC) in the GWAS database. Because it was based on publicly available aggregated data, no additional ethical approval or consent to participate was required.

### Selection of genetic variants

The instrumental variables (IVs) at a P value less than 5×10^−8^ were selected, because of the available single nucleotide polymorphisms (SNPs) limited in number (11). To obtain IVs from independent loci, we used the “TwoSampleMR” software package to set the linkage disequilibrium threshold with R^2^<0.001 and kb=10000. T Subsequently, essential details such as the effective allele and effective size (comprising β value, standard error, and P-value) of each SNP are extracted for the computation of the F-statistic to assess potential bias from weak instrumental variables (IVs). An F-statistic exceeding 10 is considered adequate for mitigating any bias arising from weak IVs. When no expose-related SNPs were present in the outcome data, we conducted a follow-up analysis by finding and selecting suitable proxy SNPs (r^2^> 0.8). Finally, SNPs with palindromic structures are automatically excluded during the analysis. More importantly, the selected genetic variances are significantly related to breast cancer only through immune cells, not associated with confounders. In addition, several potential confounding factors may influence the relationship between immune cells and breast cancer. We conducted additional queries for these SNPs in the PhenoScanner database (http://www.phenoscanner.medschl.cam.ac.uk/), excluding SNPs linked to alternative potential confounders like gender, educational attainment, smoking, body mass index, total cholesterol, Age at menarche, alcohol intake frequency, family history of cancer, other personal history of cancer. The following genetic variants, namely rs10758669, rs2049045, rs61739285, rs10146962, and rs7082470, were excluded from the analysis on account of age at menarche. Additionally, rs439401 and rs13344267 were omitted due to considerations related to total cholesterol levels. Variants rs10406080, rs6440013, rs754388, rs17437411, rs60699901, rs62501136, rs165944, rs880749, and rs2267373 were excluded from the study based on body mass index criteria. Similarly, rs63750417 and rs492602 were disregarded due to the frequency of alcohol intake. Lastly, rs17360661 was excluded from the analysis due to a family history of cancer.

### Statistical analysis

Multiple statistical approaches were employed, encompassing the inverse‐variance weighted (IVW) method, MR-Egger, weighted median, weighted mode, simple mode, and MR-Pleiotropy residual sum and outlier (MR-PRESSO) tests. The IVW model is the main analytical method to test causality by performing a meta-analysis of each Wald ratio of valid SNPs included, which yielded the most accurate effect estimates, and it served as the primary analysis in nearly all MR investigations (12). In contrast, the MR-Egger analysis can still work when all SNPS are invalid, which was evaluated as horizontal pleiotropy. The slope of MR-Egger shows the relationship between them when the intercept term has no statistical significance or zero. The Cochran Q test is employed to access heterogeneity among the selected SNPs, with heterogeneity indicated when the value falls below 0.05. Then, the MR-PRESSO test conducts a comprehensive assessment for heterogeneity to identify potential outliers within the SNP data, subsequently deriving an adjusted association outcome after eliminating these potential outliers. We utilized the odds ratio (OR) with its associated 95% confidence interval (CI) to gauge the causal relationship between the variables. To avoid horizontal pleiotropy caused by a single SNP, the “leave‐one‐out” analysis was performed. If the SNP in the analysis is less than 3, it will be excluded. Furthermore, scatter plots and funnel plots were employed. Scatter plots indicated that the outcomes remained unaffected by any outliers. Funnel plots confirmed the stability of the correlation and indicated the absence of heterogeneity.

### Bonferroni method for correction

For a more robust elucidation of causality, we applied the Bonferroni method to set multiple test significance thresholds across various classification levels, considering the count numbers within each type (4.2×10^−4^ (0.05/118) for AC counts, 1.3×10^−4^ (0.05/389) for MFI counts, 1.6×10^−3^ (0.05/32) for MP counts, 2.6×10^−4^ (0.05/192) for RC type).

To tackle the complexities arising from the polygenic nature of complex immune traits and the widespread occurrence of pleiotropy, we applied a Bayesian Weighted Mendelian Randomization (BWMR) approach for causal inference (13). This method explicitly considers the uncertainty associated with weak effects stemming from polygenicity and addresses the violation of the Instrumental Variable (IV) assumption due to pleiotropy through outlier detection using Bayesian weighting. To enhance the computational stability and efficiency of causal inference with BWMR, they have developed a Variational Expectation-Maximization (VEM) algorithm, which shown to be statistically efficient and computationally stable. Thus, we used this method to test the results by the IVW method.

**Figure 1:**
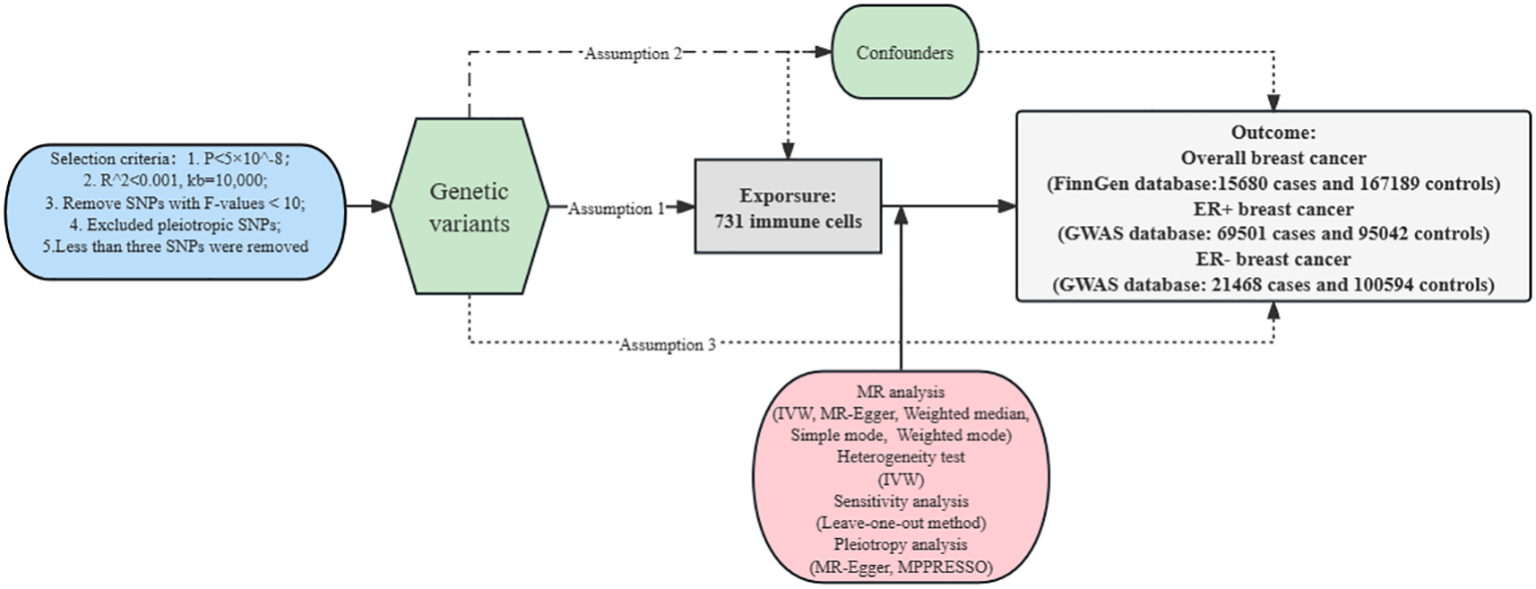
The design of Mendelian randomization analysis between immune cells and breast cancer. Assumption 1, the selected genetic variances are robustly associated with exposure; Assumption 2, the selected genetic variances are not associated with confounders; Assumption 3, the selected genetic variances are significantly related to breast cancer only through immune cells. SNPs, single-nucleotide polymorphisms. LD, linkage disequilibrium; IVW, inverse variance weighted; LOO, leave-one-out; MR, Mendelian randomization.

Our analysis was complied with a standard MR guideline (14) and our analysis was performed in the R program using the “TwoSampleMR”, “ggplot2” and “MR-PRESSO” packages. **Figure 1** shows the specific MR study design.

## Results

**Figure 2:**
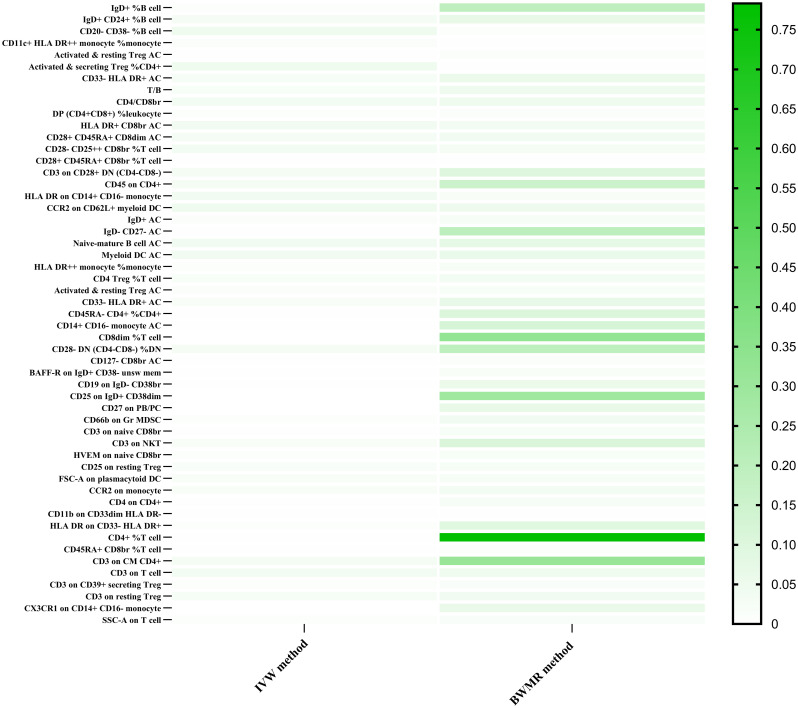
The Heatmap showed the Bayesian Weighted Mendelian Randomization (BWMR) approach to test the positive results between immune traits and breast cancers after the IVW method.

To investigate the causal impacts of breast cancer on immune cells, an MR analysis was conducted, with the IVW method being the primary analytical approach employed. As for the overall breast cancer, no immune trait was identified after the Bonferroni method. With a significance level of 0.05, we detected 18 indicative immunophenotypes. IgD+%B cell (p-value=0.0066; B cell panel), CD20-CD38-%B cell (p-value=0.0457; B cell panel), CD11c+ HLA DR++ monocyte %monocyte (p-value=0.0127; cDC panel), CD28- CD25++ CD8br %T cell (p-value=0.0339; Treg panel), Activated & resting Treg AC (p-value=0.0018; Treg panel), and T/B (p-value=0.0223; TBNK panel), DP (CD4+CD8+) %leukocyte (p-value=0.0087; TBNK panel), HLA DR+ CD8br AC (p-value=0.0386; TBNK panel), CD45 on CD4+ (p-value=0.0335; TBNK panel) had a positive correlation effect on the risk of breast cancer. While IgD+CD24+%B cell (p-value=0.0282; B cell panel), CD28+ CD45RA+ CD8dim AC (p-value=0.0284; Treg panel), CD28+ CD45RA+ CD8br %T cell (p-value=0.0013; Treg panel), CD3 on CD28+ DN (CD4-CD8-) (p-value=0.0322; Treg panel), Activated & secreting Treg %CD4+ (p-value=0.0456; Treg panel), CD33- HLA DR+ AC (p-value=0.0249; Myeloid cell panel), CD4/CD8br (p-value=0.0355; TBNK panel), HLA DR on CD14+ CD16- monocyte (p-value=0.0397; mature stages of T cells panel) and CCR2 on CD62L+ myeloid DC (p-value=0.0483; cDC panel) exhibited an elevated risk of breast cancer development. Among all these positive results, CD28+ CD45RA+ CD8br %T cell has been proven to have a positive negative effect by MR-Egger (p-value=0.0008), weighted median (p-value=0.0127), weighted mode methods (p-value=0.0324). The above analysis was shown in **Supplementary Table 1**. After Bayesian Weighted Mendelian Randomization (BWMR) approach testing, we found positive results for CD20- CD38- %B cell (p-value=0.0094), CD11c+ HLA DR++ monocyte %monocyte (p-value=0.0021), Activated & resting Treg AC (p-value=0.0130), Activated & secreting Treg %CD4+ (p-value=0.0041), T/B (p-value=0.0479), CD4/CD8br (p-value=0.0496), DP (CD4+CD8+) %leukocyte (p-value=0.0147), HLA DR+ CD8br AC (p-value=0.0369), CD28+ CD45RA+ CD8dim AC (p-value=0.0409), CD28- CD25++ CD8br %T cell (p-value=0.0335), CD28+ CD45RA+ CD8br %T cell (p-value=0.0061). The results were shown in **Figure 2** and **Supplementary Table 5**.

**Figure 3:**
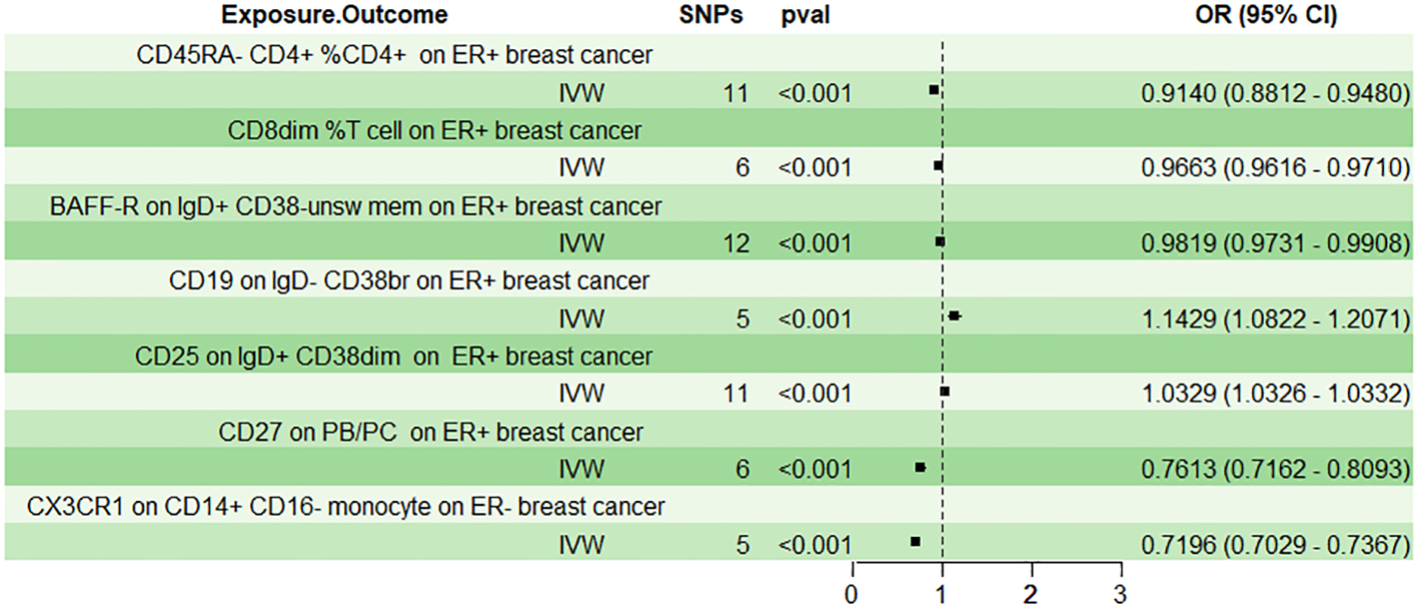
The effect of immune cells on breast cancer used the inverse‐variance weighted (IVW) method after the Bonferroni method.

In the context of ER+ breast cancer, we identified 27 suggestive immunophenotypes at a significance level of 0.05, which summarized in **Supplementary Table 2**. After employing the Bonferroni method through the IVW approach, six immune traits were discerned. Results from the IVW method indicated that CD45RA- CD4+ %CD4+ significantly decreased the risk of breast cancer (mature stages of T cells panel; Odds ratio: 0.9140, 95%CI: 0.8810 - 0.9480, p-value: 1.37×10−6), and this was corroborated by the weighted mode (Odds ratio: 0.9498, 95%CI: 0.9084 - 0.9931, p-value: 0.0471) and MR-Egger method (Odds ratio: 0.8747, 95%CI: 0.8114 - 0.9430, p-value: 0.0068). Furthermore, CD8dim %T cell was associated with a lowered risk of breast cancer as per the IVW method (TBNK panel; Odds ratio: 0.9663, 95%CI: 0.9616-0.9710, p-value: 4.62×10−43), and this was supported by the weighted mode (Odds ratio: 0.9681, 95%CI: 0.9533-0.9832, p-value: 0.0093) and weighted median (Odds ratio: 0.9671, 95%CI: 0.9474-0.9871, p-value: 0.0014). BAFF-R on IgD+ CD38- unsw mem (B cell panel; Odds ratio:0.9819, 95%CI:0.9731-0.9908, p-value:6.93×10^−5^) and CD27 on PB/PC (B cell panel; Odds ratio:0.7162, 95%CI:0.7162-0.8093, p-value:2.72×10^−18^) were both linked to a decreased risk of breast cancer according to the IVW method, and these findings were consistent with the results from the weighted mode and weighted median. Conversely, CD19 on IgD- CD38br (B cell panel; Odds ratio:1.1429, 95%CI:1.0821-1.2071, p-value:1.64×10^−6^) was associated with an increased risk of breast cancer across the IVW method, weighted mode, and weighted median. Similarly, CD25 on IgD+ CD38dim (B cell panel; Odds ratio:1.0329, 95%CI:1.0326-1.0332, p-value: - ∞) was linked to a higher risk of breast cancer based on the IVW method, weighted median, and MR-Egger. The above analysis was shown in **Table 1** and **Figure 3**. Following the Bonferroni method applied to the six aforementioned immune cells showing positive correlations, our analysis revealed that BAFF-R on IgD+ CD38- unsw mem was the sole association linked to reduced breast cancer risk using the BWMR method. The results were shown in **Figure 2** and **Supplementary Table 5**.

**Table 1:** The Mendelian analysis showed the causal effect between immune cells and breast cancers after Bonferroni method.

Immune traits	Outcome	Inverse variance weighted		MR-Egger		Weighted median		Weighted mode		Simple mode	
		OR (95% CI)	P value	OR (95% CI)	P value	OR (95% CI)	P value	OR (95% CI)	P value	OR (95% CI)	P value
CD45RA- CD4+ %CD4+	ER (+) breast cancer	0.9140(0.8812-0.9480)	1.37*10^-6^	0.8747(0.8114-0.9430)	0.0068	0.9422(0.8876-1.0001)	0.0505	0.9498(0.9084-0.9931)	0.0471	1.0010(0.9379-1.0685)	0.9756
CD8dim %T cell	ER (+) breast cancer	0.9663(0.9616-0.9710)	4.63*10^-43^	0.8449(0.6524-1.0940)	0.2702	0.9671(0.9474-0.9871)	0.0014	0.9681(0.9533-0.9832)	0.0093	1.0021(0.8897-1.1286)	0.9741
BAFF-R on IgD+ CD38- unsw mem	ER (+) breast cancer	0.9819(0.9731-0.9908)	6.93*10^-5^	0.9805(0.9596-1.0020)	0.1049	0.9879(0.9740-1.0021)	0.0939	0.9884(0.9793-0.9976)	0.0318	0.9833(0.9608-1.0062)	0.1794
CD19 on IgD- CD38br	ER (+) breast cancer	1.1429(1.0822-1.2071)	1.64*10^-6^	1.3879(1.0975-1.7550)	0.0715	1.1197(1.0204-1.2287)	0.0171	1.1653(1.0784-1.2591)	0.0180	0.9968(0.7948-1.2502)	0.9794
CD25 on IgD+ CD38dim	ER (+) breast cancer	1.0329(1.0326-1.0332)	0	1.0613(1.0466-1.0762)	1.53*10^-5^	1.0233(1.0058-1.0410)	0.0086	1.0259(0.9938-1.0590)	0.1464	0.9861(0.9188-1.0585)	0.7074
CD27 on PB/PC	ER (+) breast cancer	0.7614(0.7162-0.8093)	2.27*10^-18^	1.8823(0.2686-13.1929)	0.5590	0.7851(0.6721-0.9171)	0.7614	0.7510(0.6562-0.8596)	0.0088	1.0144(0.6697-1.5363)	0.9490
CX3CR1 on CD14+ CD16- monocyte	ER (-) breast cancer	0.7196(0.7029-0.7367)	1.15*10^-166^	0.6111(0.2584-1.4450)	0.3437	0.7327(0.6133-0.8754)	0.0006	0.7190(0.6193-0.8348)	0.0123	1.1004(0.6831-1.7726)	0.7143

Seven suggestive immunophenotypes were identified at the significance of 0.05, and only one immune trait was identified in ER- breast cancer after the Bonferroni method (see **Supplementary Table 3**). As for the CX3CR1 on CD14+ CD16- monocyte (Monocyte type, IVW: Odds ratio:0.7196, 95%CI:0.7029-0.7367, p-value: 1.15×10^−166^), the IVW method and weighted median clearly demonstrated a protective effect against ER- breast cancer. But after the BWMR test, CX3CR1 on CD14+ CD16- monocyte did not show a positive result. The results were shown in **Figure 2** and **Supplementary Table 5**.

**Figure 4:**
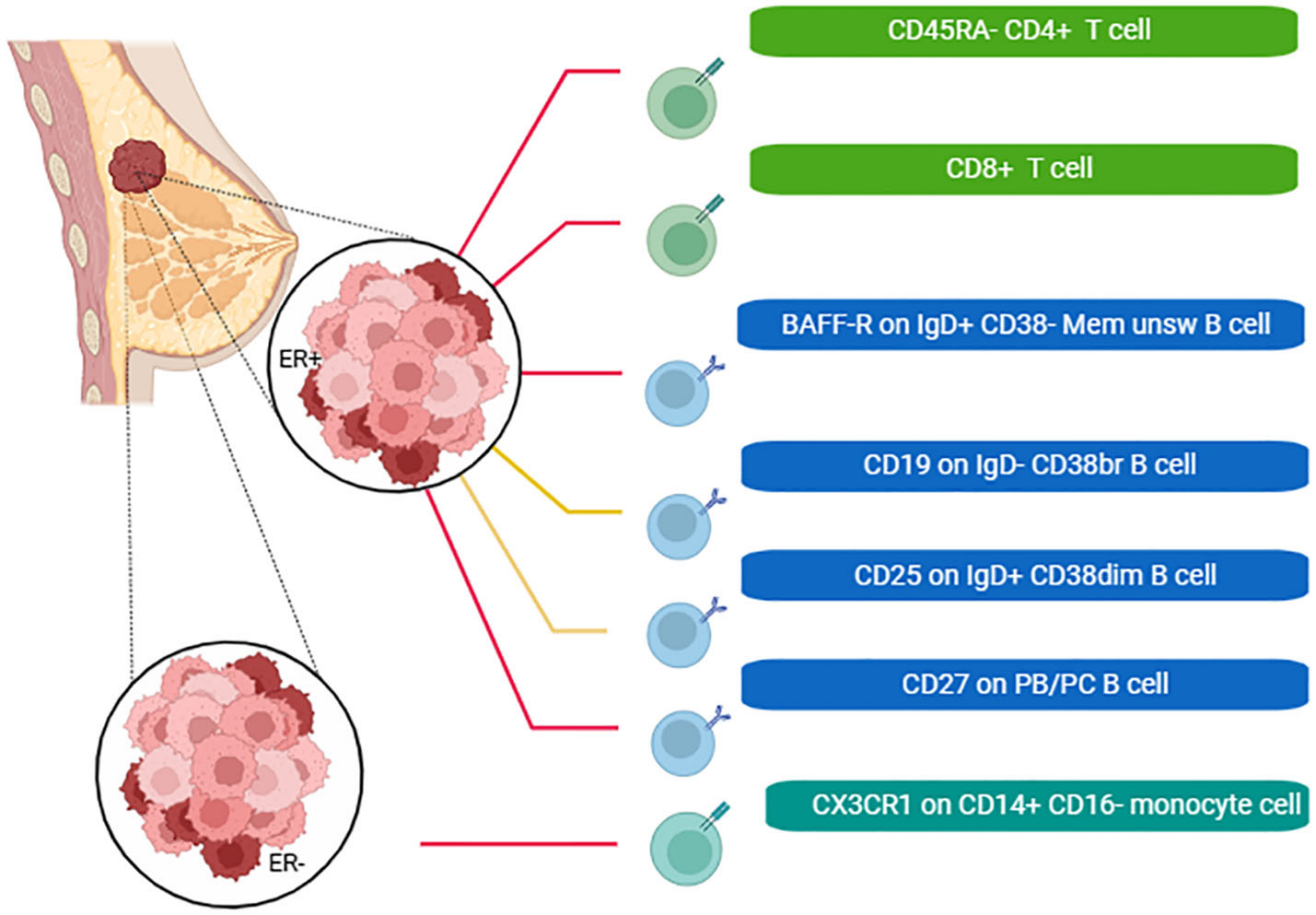
A schematic summary figure for the positive results after the Bonferroni method. The red line indicated that the immune cells could reduce the incidence of breast cancer, while the yellow line was the opposite (Created with BioRender.com).

We also plotted a schematic summary figure for the positive results in **Figure 4**. would be helpful for general audience to comprehend the results We employed Cochrane’s Q test and MR Egger regression analysis to assess the extent of heterogeneity and horizontal pleiotropy (see **Supplementary Table 4**). Consistently across all reported outcomes, these tests consistently indicated an absence of heterogeneity (p > 0.05). Furthermore, both the intercept term in MR-Egger regression and MR-PRESSO analysis indicated the absence of significant overall horizontal pleiotropy. The Leave-one-out sensitivity analysis for the associations was shown in **Supplementary Figure S1**, while the scatter and funnel plots of each pair of associations were shown in **Supplementary Figures S2**, **S3**.

## Discussion

Utilizing a substantial volume of publicly accessible genetic data, we investigated the causal association between 731 immune cell signatures and breast cancer. To the best of our knowledge, this study represents the inaugural Mendelian randomization (MR) analysis exploring the causal connection between numerous immunophenotypes and breast cancer. Our findings indicated that 53 immunophenotypes spanning four types of immune signatures (MFI, RC, AC, and MP) causally influences breast cancers. After the Bonferroni method, 7 immunophenotypes were found to be associated with breast cancer.

Ruffell et al. observed that breast cancer tissue contained infiltrates dominated by CD8+ and CD4+ lymphocytes, with fewer NK cells and B lymphocytes, while myeloid cells including macrophages, mast cells, and neutrophils were more pronounced in normal breast tissue (15). Among these immune phenotypes, the most significant reduction in breast cancer is CD4+ %T cell. CD4+% cells are mainly considered as helper cells for activating CD8+effector T cells, and there is evidence that CD4+% T cells also have independent functions in promoting anti-tumor immunity. Research has found that a subgroup of CD4+%T cells can produce a cytolytic effect on tumor cells expressing MHC II; Moreover, CD4+%T cells have demonstrated the capability to eradicate tumor cells lacking MHC-II expression through the mobilization of myeloid cells (16, 17). The Thomas Tüting team found that CD4+T cells could also independently eliminate formed tumors like CD8+cytolytic T cells, engage with CD11c+MHC-II+antigen presenting immune cells, and indirectly lead to the elimination of tumors. Meanwhile, it was further revealed that CD4+T cells, in conjunction with IFN-activated mononuclear phagocytes, collectively instigated an indirect inflammatory process resulting in tumor cell death (18).. In recent years, CAR-T technology has been used to modify CD4+ T cells, and anti-tumor ability does not depend on cytotoxicity, but indirectly acts on tumor cells through the production of interferon-γ (IFN-γ), a large area and a long distance (19). In addition, the successful application of Adoptive cell transfer (ACT) immunotherapy with CD4+T cells in clinical research holds immense significance for the prospective treatment of cancer patients, undoubtedly paving the way for advancements in future therapies. By releasing the CD4+T cell effector function, it can immune escape the killing of tumors (20). In addition, CD45RA- CD4+ %CD4+ was also proven to be significantly linked with a decreased risk of breast cancer (21).

Monocytes, the main subpopulation of ‘classical’ CD14^+^CD16^-^ monocytes, can differentiate into dendritic cells and macrophages, which can participate in the host’s anti-tumor response. In addition, CX3CL1 has antitumor effects by recruiting anti-tumor immune cells into the tumor microenvironment to control tumor growth (22). And we found that they could reduce the risk of ER breast cancer. Some results showed that CD14+CD16+ monocytes CD14+CD16+ monocytes could serve as a valuable indicator for the early detection of breast cancer, but our analysis found no evidence of this (23).

New research findings suggested that enhanced clinical outcomes may be linked to various facets of the humoral immune response, characterized by B-lymphocyte infiltration into tumors and the expression of antibodies in lesions or circulation (24). Tumor-infiltrating B lymphocytes (TIL-B) clustered within the tertiary lymphoid structure may exhibit antigen-educing phenotypes, and autoantibodies are believed to trigger tumor cell clearance (25). Robert J. Harris et al. found a positive correlation between IgG+-based clonal expansion of B-lymphocyte immune system highly infiltrated response cells of breast cancer, and IgG-like regulatory signals and patient prognosis (26). Compared to CD27^+^IgG^-^ B cells, the CD27^+^IgG^+^ B cells markedly elevated expression of IFN-γ, which was consistent with our finding that CD27 on PB/PC reduces the risk of breast cancer.

The obtained results are robust and remained unaffected by horizontal pleiotropy and other potential confounding factors, ensuring the reliability and validity of the findings. However, our study also has limitations. Firstly, we didn’t investigate the immune cell and other cancer phenotypes, such as lung cancer, thyroid cancer, colorectal cancer, etc. Secondly, the majority of our data pertains to individuals of European ancestry, so further research is necessary to ascertain whether our findings extend to Asian and other ancestral groups. Finally, despite conducting multiple sensitivity analyses to assess the assumptions of the Mendelian randomization study, it is not possible to entirely eliminate the potential for confounding bias and/or horizontal pleiotropy.

The implications of immune cells in breast cancer have significant implications for future studies and clinical practice. Further investigation into the role of immune cells can guide the development of targeted immunotherapies. Research focusing on harnessing the body’s immune response to specifically target breast cancer cells may offer new treatment modalities. Identifying specific immune cell profiles, such as CD45RA- CD4+ %CD4+, CD8dim %T cell, BAFF-R on IgD+ CD38- unsw mem, CD27 on PB/PC, CD19 on IgD- CD38br, CD25 on IgD+ CD38dim, CX3CR1, as prognostic and predictive biomarkers is an ongoing area of research. Future studies may explore the dynamic changes in immune cell composition throughout the course of the disease and in response to different treatments. Stratifying patients based on their immune profiles may become essential for tailoring treatment strategies. Future studies could focus on refining subtyping based on the immune microenvironment, allowing for more precise treatment selection.

## Conclusions

Through the application of Mendelian randomization analysis, we have effectively showcased the causal associations between multiple immunophenotypes and breast cancer. This underscores the intricate and multifaceted nature of interactions between the immune system and breast cancer within our findings. Our discoveries broaden the scope of immunological insights and offer valuable indications for breast cancer prevention, potentially facilitating earlier diagnosis and the development of more efficacious treatment alternatives.

## Data availability statement

The original contributions presented in the study are included in the article/**Supplementary Material**. Further inquiries can be directed to the corresponding author.

## Author contributions

XW: Conceptualization, Data curation, Formal analysis, Writing – original draft. HG: Formal analysis, Methodology, Validation, Writing – original draft. YZ: Investigation, Software, Supervision, Writing – original draft. JC: Supervision, Validation, Visualization, Writing – review & editing.

## Supplementary material

Supplementary Figure 1 The sensitivity analysis of immune cells on overall breast cancer, ER+ breast cancer and ER- breast cancer.

Supplementary Figure 2 The scatter plots of the associations between immune cells on overall breast cancer, ER+ breast cancer and ER- breast cancer.

Supplementary Figure 3 Funnel plot to assess heterogeneity between immune cells on overall breast cancer, ER+ breast cancer and ER- breast cancer.

## References

[1] Kocarnik JM Compton K Dean FE Fu W Gaw BL Harvey JD. Cancer incidence, mortality, years of life lost, years lived with disability, and disability-adjusted life years for 29 cancer groups from 2010 to 2019: A systematic analysis for the global burden of disease study 2019. JAMA Oncol (2022) 8(3):420–44. doi: 10.1001/jamaoncol.2021.6987 PMC8719276 34967848

[2] Sung H Ferlay J Siegel RL Laversanne M Soerjomataram I Jemal A. Global cancer statistics 2020: GLOBOCAN estimates of incidence and mortality worldwide for 36 cancers in 185 countries. CA Cancer J Clin (2021) 71(3):209–49. doi: 10.3322/caac.21660 33538338

[3] Qian BZ Pollard JW. Macrophage diversity enhances tumor progression and metastasis. Cell (2010) 141(1):39–51. doi: 10.1016/j.cell.2010.03.014 20371344 PMC4994190

[4] Perez EA Ballman KV Tenner KS Thompson EA Badve SS Bailey H. Association of stromal tumor-infiltrating lymphocytes with recurrence-free survival in the N9831 adjuvant trial in patients with early-stage HER2-positive breast cancer. JAMA Oncol (2016) 2(1):56–64. doi: 10.1001/jamaoncol.2015.3239 26469139 PMC4713247

[5] Bell D Chomarat P Broyles D Netto G Harb GM Lebecque S. In breast carcinoma tissue, immature dendritic cells reside within the tumor, whereas mature dendritic cells are located in peritumoral areas. J Exp Med (1999) 190(10):1417–26. doi: 10.1084/jem.190.10.1417 PMC2195690 10562317

[6] Loi S Michiels S Salgado R Sirtaine N Jose V Fumagalli D. Tumor infiltrating lymphocytes are prognostic in triple negative breast cancer and predictive for trastuzumab benefit in early breast cancer: results from the FinHER trial. Ann Oncol (2014) 25(8):1544–50. doi: 10.1093/annonc/mdu112 24608200

[7] Davey Smith G Hemani G. Mendelian randomization: genetic anchors for causal inference in epidemiological studies. Hum Mol Genet (2014) 23(R1):R89–98. doi: 10.1093/hmg/ddu328 PMC4170722 25064373

[8] Timpson NJ Wade KH Smith GD. Mendelian randomization: application to cardiovascular disease. Curr Hypertens Rep (2012) 14(1):29–37. doi: 10.1007/s11906-011-0242-7 22161218

[9] Orrù V Steri M Sidore C Marongiu M Serra V Olla S. Complex genetic signatures in immune cells underlie autoimmunity and inform therapy. Nat Genet (2020) 52(10):1036–45. doi: 10.1038/s41588-020-0684-4 PMC8517961 32929287

[10] Sidore C Busonero F Maschio A Porcu E Naitza S Zoledziewska M. Genome sequencing elucidates Sardinian genetic architecture and augments association analyses for lipid and blood inflammatory markers. Nat Genet (2015) 47(11):1272–81. doi: 10.1038/ng.3368 PMC4627508 26366554

[11] Yu XH Yang YQ Cao RR Bo L Lei SF. The causal role of gut microbiota in development of osteoarthritis. Osteoarthritis Cartilage (2021) 29(12):1741–50. doi: 10.1016/j.joca.2021.08.003 34425228

[12] Hemani G Zheng J Elsworth B Wade KH Haberland V Baird D. The MR-Base platform supports systematic causal inference across the human phenome. Elife (2018) 7:e34408. doi: 10.7554/eLife.34408 29846171 PMC5976434

[13] Zhao J Ming J Hu X Chen G Liu J Yang C. Bayesian weighted Mendelian randomization for causal inference based on summary statistics. Bioinformatics (2020) 36(5):1501–8. doi: 10.1093/bioinformatics/btz749 31593215

[14] Burgess S Davey Smith G Davies NM Dudbridge F Gill D Glymour MM. Guidelines for performing Mendelian randomization investigations: update for summer 2023. Wellcome Open Res (2019) 4:186. doi: 10.12688/wellcomeopenres.15555.1 32760811 PMC7384151

[15] Ruffell B Au A Rugo HS Esserman LJ Hwang ES Coussens LM. Leukocyte composition of human breast cancer. Proc Natl Acad Sci U S A. (2012) 109(8):2796–801. doi: 10.1073/pnas.1104303108 PMC3287000 21825174

[16] Śledzińska A Vila de Mucha M Bergerhoff K Hotblack A Demane DF Ghorani E. Regulatory T cells restrain interleukin-2- and blimp-1-dependent acquisition of cytotoxic function by CD4(+) T cells. Immunity (2020) 52(1):151–66.e6. doi: 10.1016/j.immuni.2019.12.007 31924474 PMC7369640

[17] Corthay A Skovseth DK Lundin KU Røsjø E Omholt H Hofgaard PO. Primary antitumor immune response mediated by CD4+ T cells. Immunity (2005) 22(3):371–83. doi: 10.1016/j.immuni.2005.02.003 15780993

[18] Kruse B Buzzai AC Shridhar N Braun AD Gellert S Knauth K. CD4(+) T cell-induced inflammatory cell death controls immune-evasive tumours. Nature (2023) 618(7967):1033–40. doi: 10.1038/s41586-023-06199-x PMC10307640 37316667

[19] Boulch M Cazaux M Cuffel A Guerin MV Garcia Z Alonso R. Tumor-intrinsic sensitivity to the pro-apoptotic effects of IFN-γ is a major determinant of CD4(+) CAR T-cell antitumor activity. Nat Cancer (2023) 4(7):968–83. doi: 10.1038/s43018-023-00570-7 PMC10368531 37248395

[20] Waldman AD Fritz JM Lenardo MJ. A guide to cancer immunotherapy: from T cell basic science to clinical practice. Nat Rev Immunol (2020) 20(11):651–68. doi: 10.1038/s41577-020-0306-5 PMC7238960 32433532

[21] Bailur JK Gueckel B Derhovanessian E Pawelec G. Presence of circulating Her2-reactive CD8 + T-cells is associated with lower frequencies of myeloid-derived suppressor cells and regulatory T cells, and better survival in older breast cancer patients. Breast Cancer Res (2015) 17(1):34. doi: 10.1186/s13058-015-0541-z 25849846 PMC4377034

[22] Bronger H Magdolen V Goettig P Dreyer T. Proteolytic chemokine cleavage as a regulator of lymphocytic infiltration in solid tumors. Cancer Metastasis Rev (2019) 38(3):417–30. doi: 10.1007/s10555-019-09807-3 PMC6890590 31482487

[23] Feng AL Zhu JK Sun JT Yang MX Neckenig MR Wang XW. CD16+ monocytes in breast cancer patients: expanded by monocyte chemoattractant protein-1 and may be useful for early diagnosis. Clin Exp Immunol (2011) 164(1):57–65. doi: 10.1111/j.1365-2249.2011.04321.x 21361908 PMC3074217

[24] Scott AM Wolchok JD Old LJ. Antibody therapy of cancer. Nat Rev Cancer (2012) 12(4):278–87. doi: 10.1038/nrc3236 22437872

[25] Rossetti RAM Lorenzi NPC Yokochi K Rosa M Benevides L Margarido PFR. B lymphocytes can be activated to act as antigen presenting cells to promote anti-tumor responses. PloS One (2018) 13(7):e0199034. doi: 10.1371/journal.pone.0199034 29975708 PMC6033398

[26] Harris RJ Cheung A Ng JCF Laddach R Chenoweth AM Crescioli S. Tumor-infiltrating B lymphocyte profiling identifies igG-biased, clonally expanded prognostic phenotypes in triple-negative breast cancer. Cancer Res (2021) 81(16):4290–304. doi: 10.1158/0008-5472.CAN-20-3773 PMC7611538 34224371

