# Supplementary material for: A Mendelian analysis of the relationships between immune cells and breast cancer
Source: Front Oncol. 2024 Jan 24;14:1341292. doi: 10.3389/fonc.2024.1341292 (PMC10847340; doi:10.3389/fonc.2024.1341292)
Supplement: Supplementary Figure 1 — The sensitivity analysis of immune cells on overall breast cancer, ER+ breast cancer and ER- breast cancer. [file DataSheet_1.pdf]

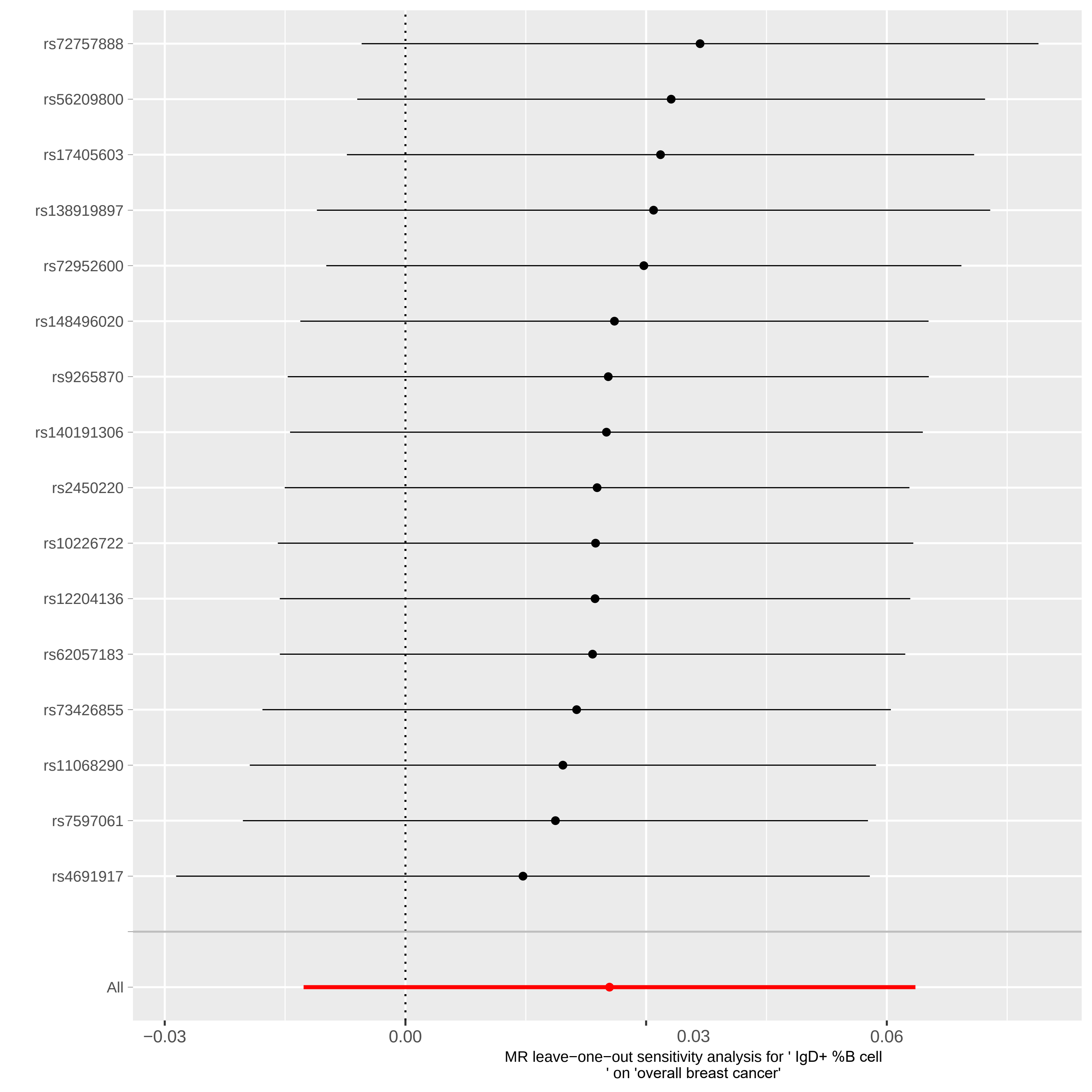
rs72757888
rs56209800
rs17405603
rs138919897
rs72952600
rs148496020
rs9265870
rs140191306
rs2450220
rs10226722
rs12204136
rs62057183
rs73426855
rs11068290
rs7597061
rs4691917
All
−0.03
0.00
0.03
0.06
MR leave−one−out sensitivity analysis for ' IgD+ %B cell
' on 'overall breast cancer'

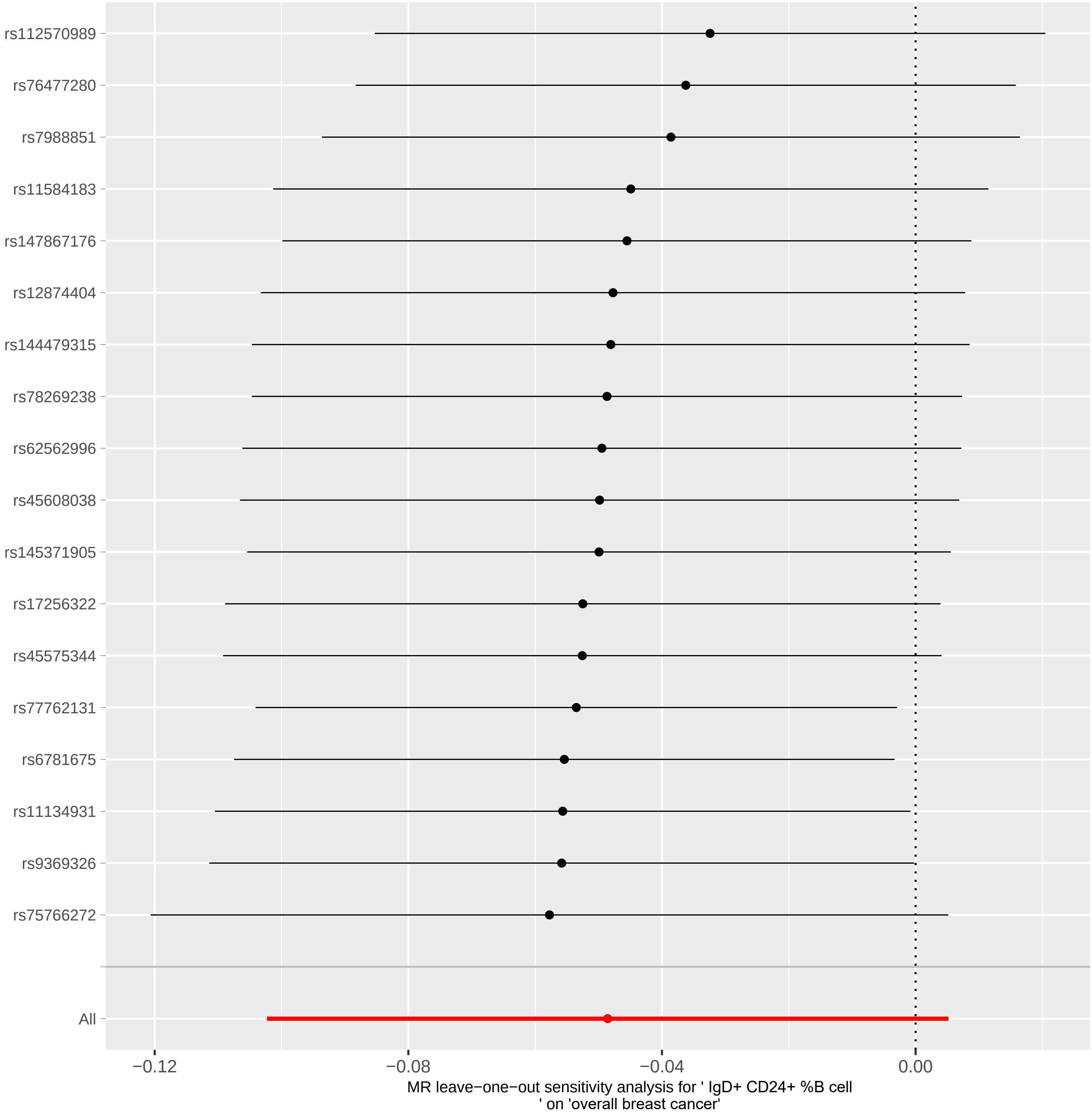
rs112570989
rs76477280
rs7988851
rs11584183
rs147867176
rs12874404
rs144479315
rs78269238
rs62562996
rs45608038
rs145371905
rs17256322
rs45575344
rs77762131
rs6781675
rs11134931
rs9369326
rs75766272
All
−0.12
−0.08
−0.04
0.00
MR leave−one−out sensitivity analysis for ' IgD+ CD24+ %B cell
' on 'overall breast cancer'

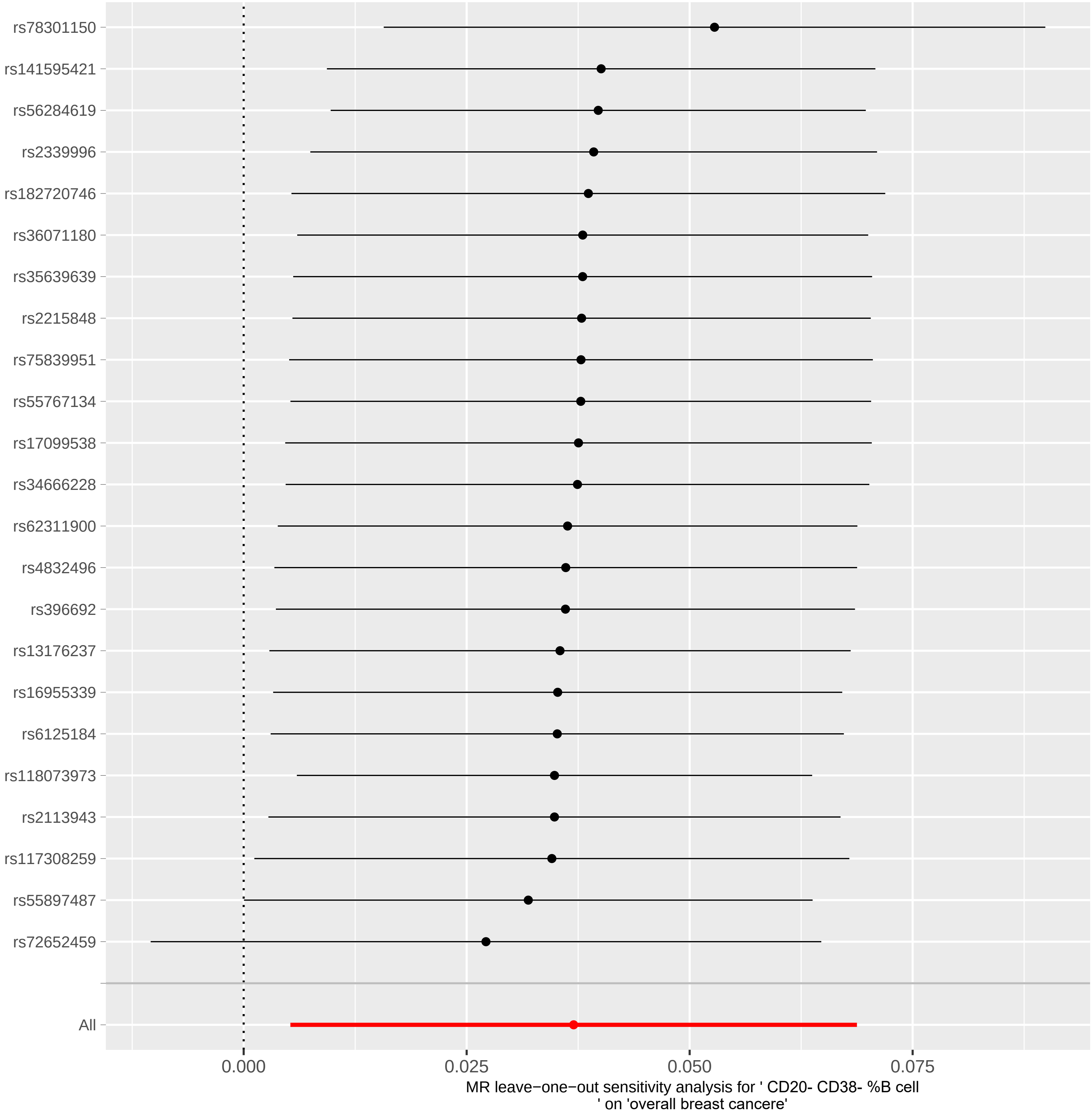
rs78301150
rs141595421
rs56284619
rs2339996
rs182720746
rs36071180
rs35639639
rs2215848
rs75839951
rs55767134
rs17099538
rs34666228
rs62311900
rs4832496
rs396692
rs13176237
rs16955339
rs6125184
rs118073973
rs2113943
rs117308259
rs55897487
rs72652459
All
0.000
0.025
0.050
0.075
MR leave−one−out sensitivity analysis for ' CD20- CD38- %B cell
' on 'overall breast cancere'

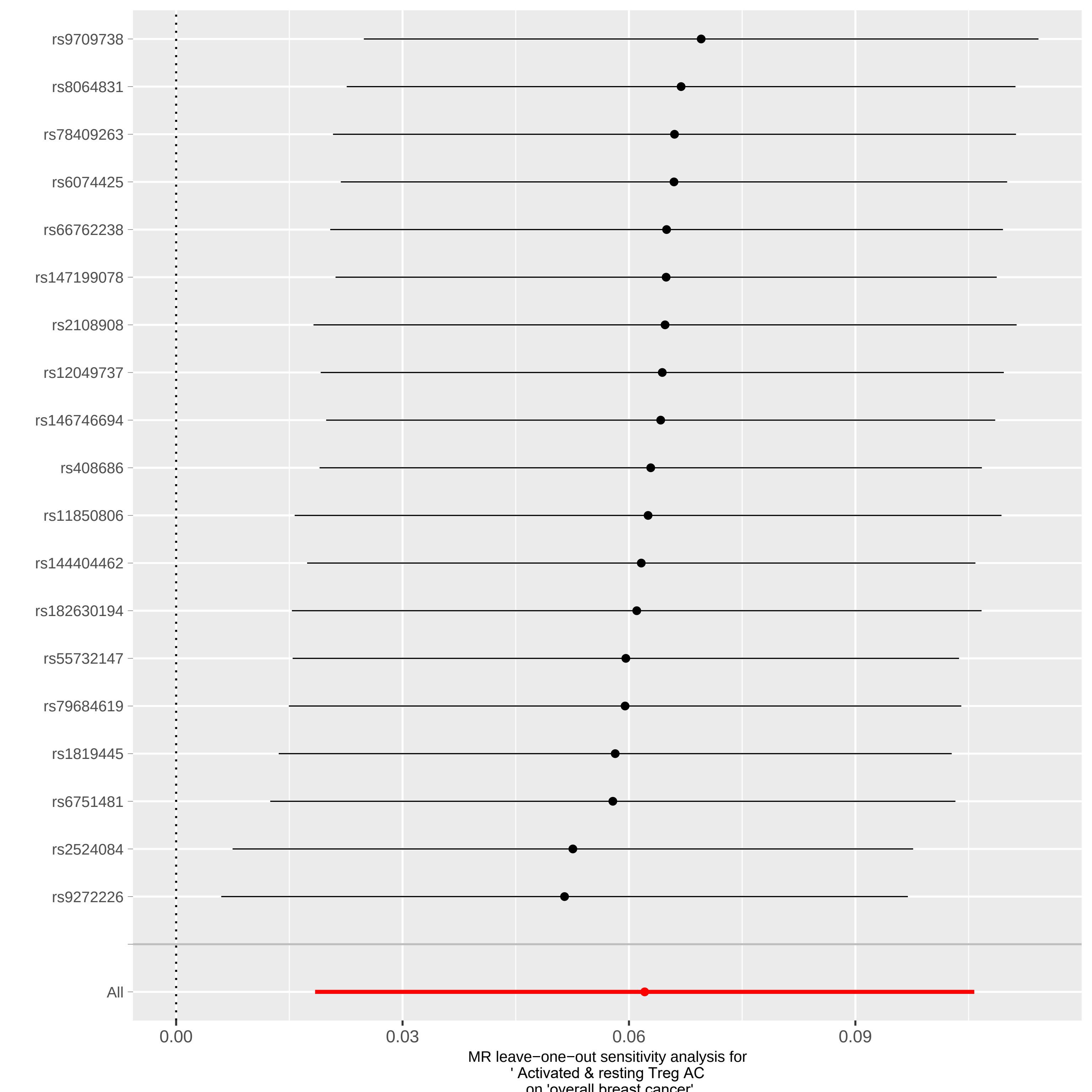

rs9709738
rs8064831
rs78409263
rs6074425
rs66762238
rs147199078
rs2108908
rs12049737
rs146746694
rs408686
rs11850806
rs144404462
rs182630194
rs55732147
rs79684619
rs1819445
rs6751481
rs2524084
rs9272226
All
0.00
0.03
0.06
0.09
MR leave−one−out sensitivity analysis for
' Activated & resting Treg AC
on 'overall breast cancer'

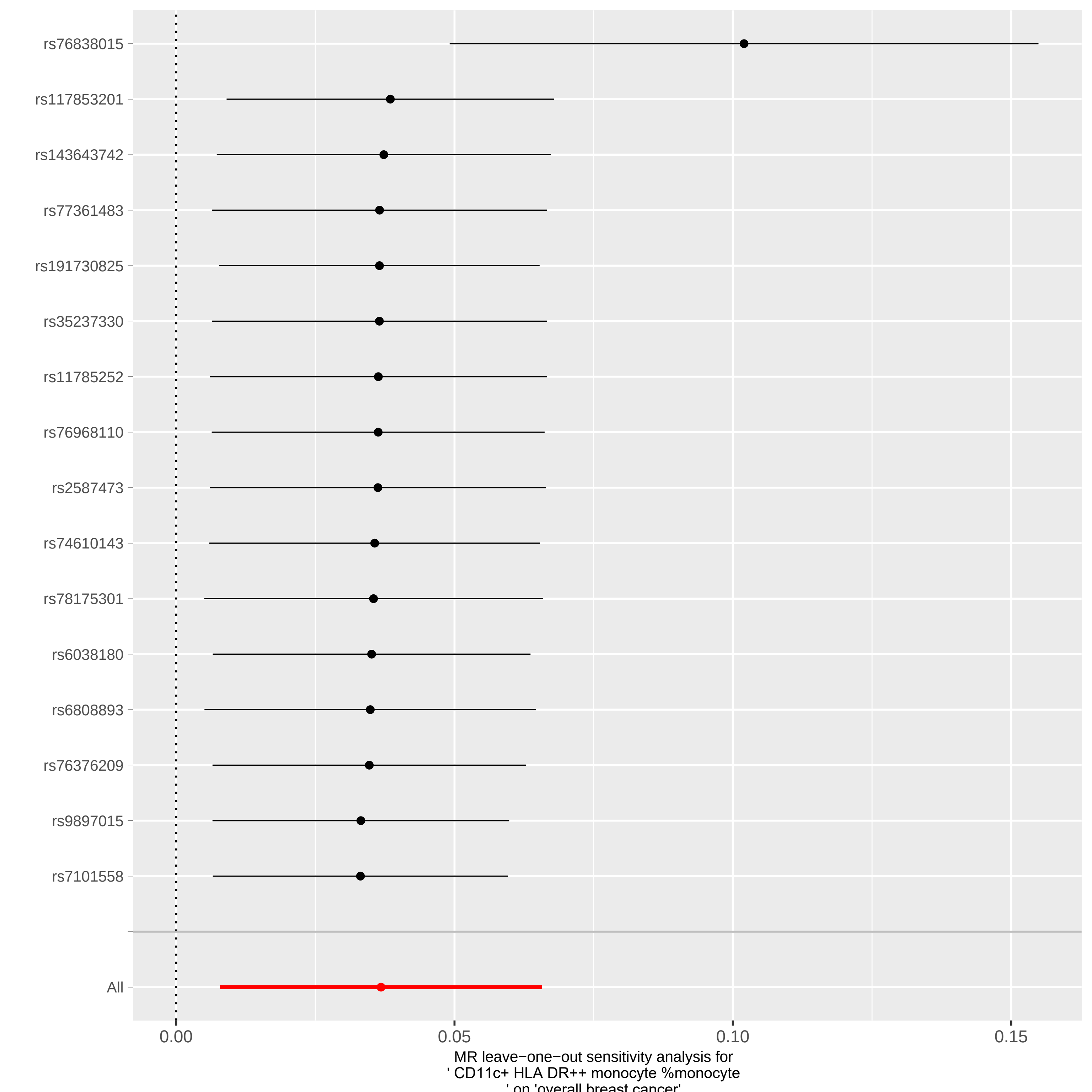
rs76838015
rs117853201
rs143643742
rs77361483
rs191730825
rs35237330
rs11785252
rs76968110
rs2587473
rs74610143
rs78175301
rs6038180
rs6808893
rs76376209
rs9897015
rs7101558
All
0.00
0.05
0.10
0.15
MR leave−one−out sensitivity analysis for
' CD11c+ HLA DR++ monocyte %monocyte
' on 'overall breast cancer'

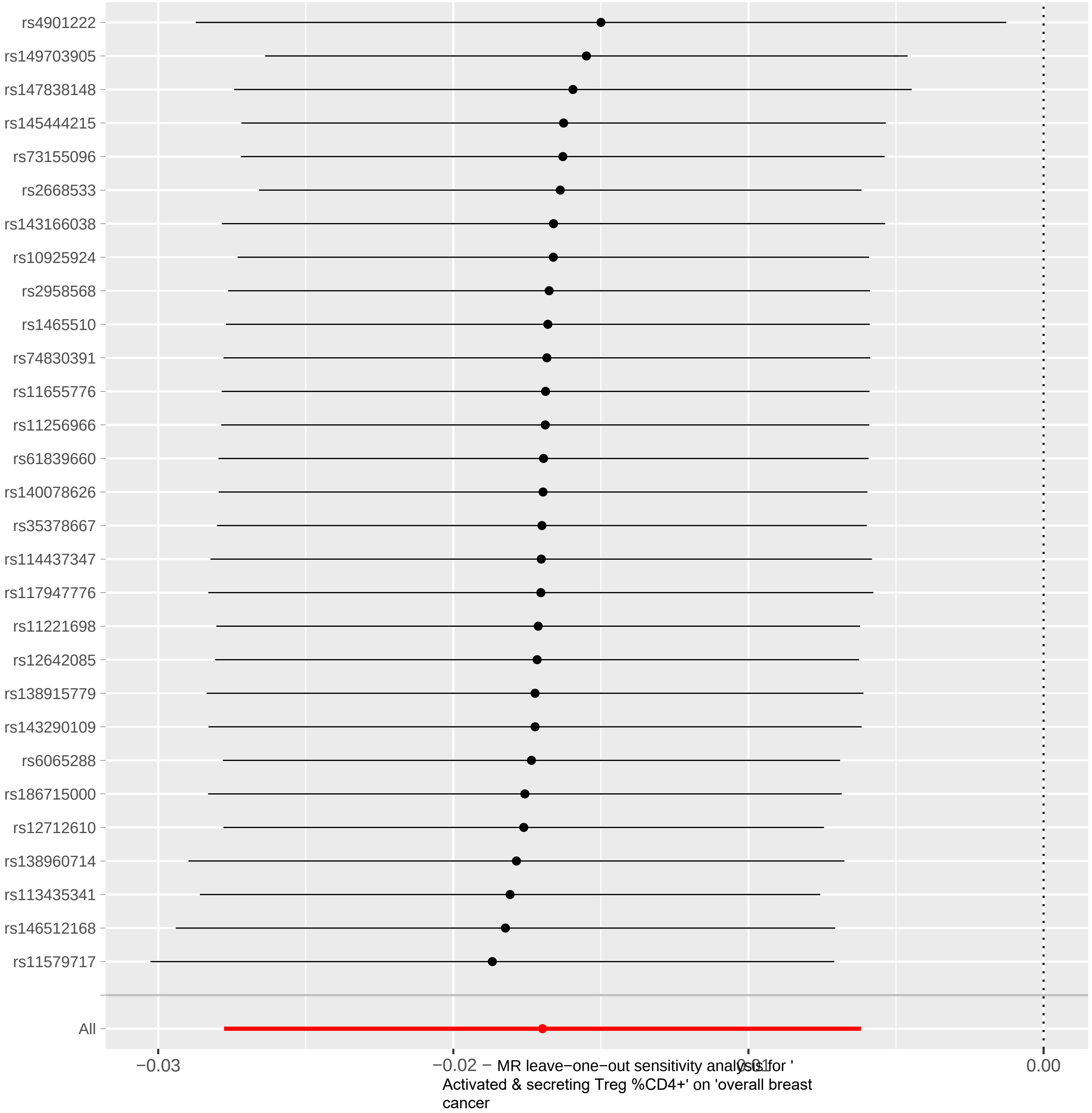
rs4901222
rs149703905
rs147838148
rs145444215
rs73155096
rs2668533
rs143166038
rs10925924
rs2958568
rs1465510
rs74830391
rs11655776
rs11256966
rs61839660
rs140078626
rs35378667
rs114437347
rs117947776
rs11221698
rs12642085
rs138915779
rs143290109
rs6065288
rs186715000
rs12712610
rs138960714
rs113435341
rs146512168
rs11579717
All
−0.03
−0.02
−0.01
0.00
MR leave−one−out sensitivity analysis for 'Activated & secreting Treg %CD4+' on 'overall breast cancer

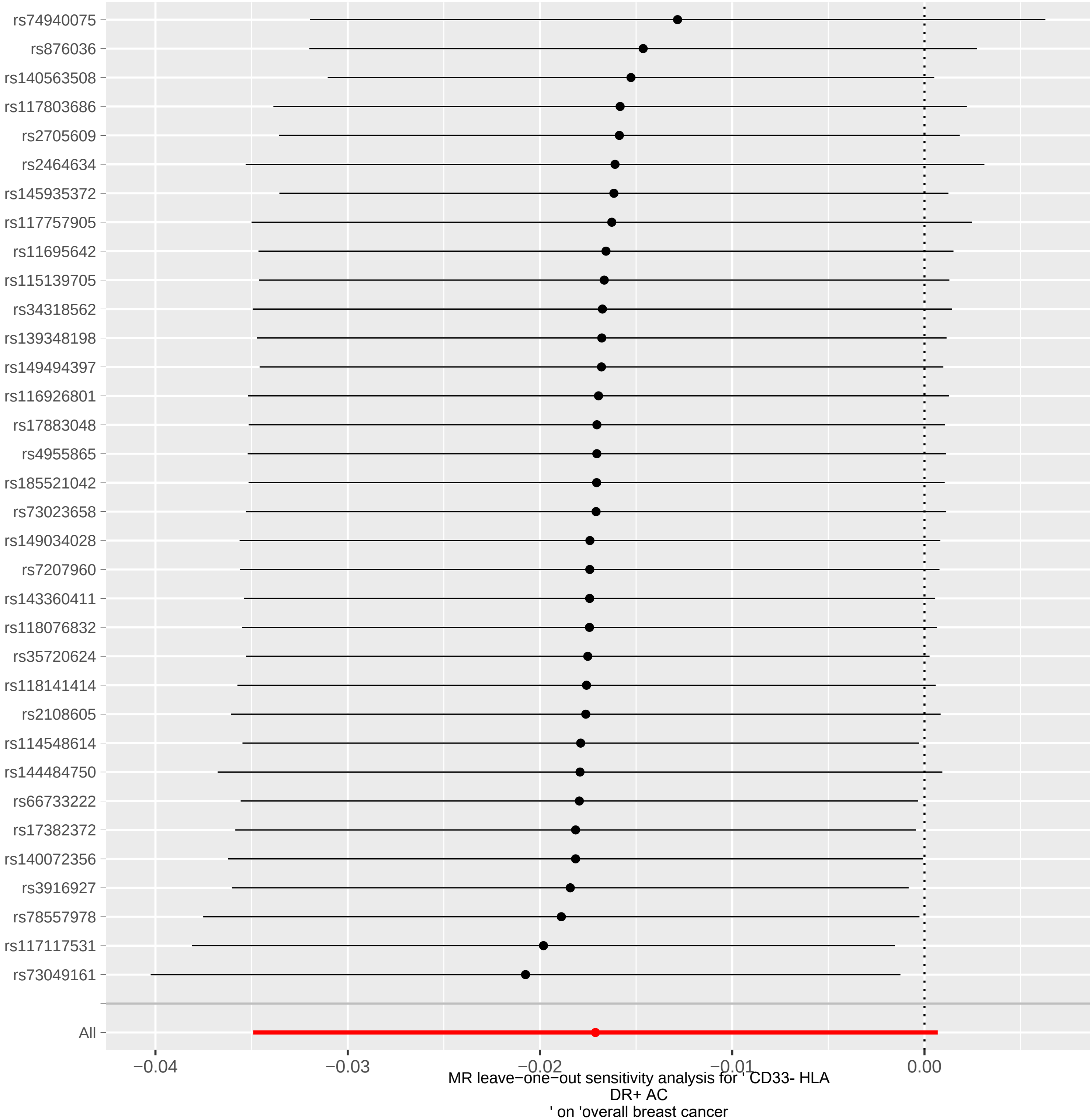
rs74940075
rs876036
rs140563508
rs117803686
rs2705609
rs2464634
rs145935372
rs117757905
rs11695642
rs115139705
rs34318562
rs139348198
rs149494397
rs116926801
rs17883048
rs4955865
rs185521042
rs73023658
rs149034028
rs7207960
rs143360411
rs118076832
rs35720624
rs118141414
rs2108605
rs114548614
rs144484750
rs66733222
rs17382372
rs140072356
rs3916927
rs78557978
rs117117531
rs73049161
All
−0.04
−0.03
−0.02
−0.01
0.00
MR leave−one−out sensitivity analysis for ' CD33- HLA
DR+ AC
' on 'overall breast cancer

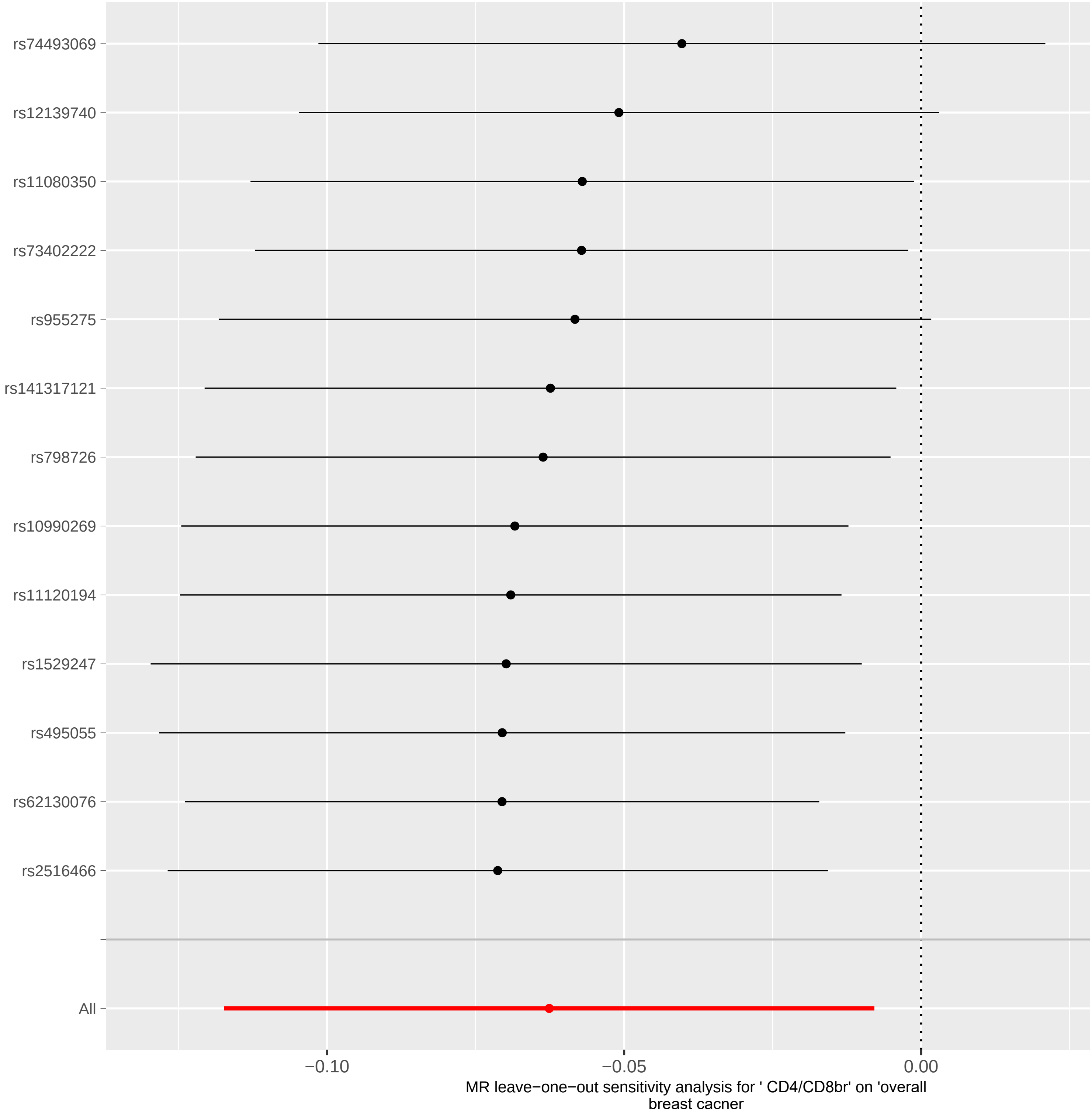

rs74493069
rs12139740
rs11080350
rs73402222
rs955275
rs141317121
rs798726
rs10990269
rs11120194
rs1529247
rs495055
rs62130076
rs2516466
All
−0.10
−0.05
0.00
MR leave−one−out sensitivity analysis for ' CD4/CD8br' on 'overall
breast cacner

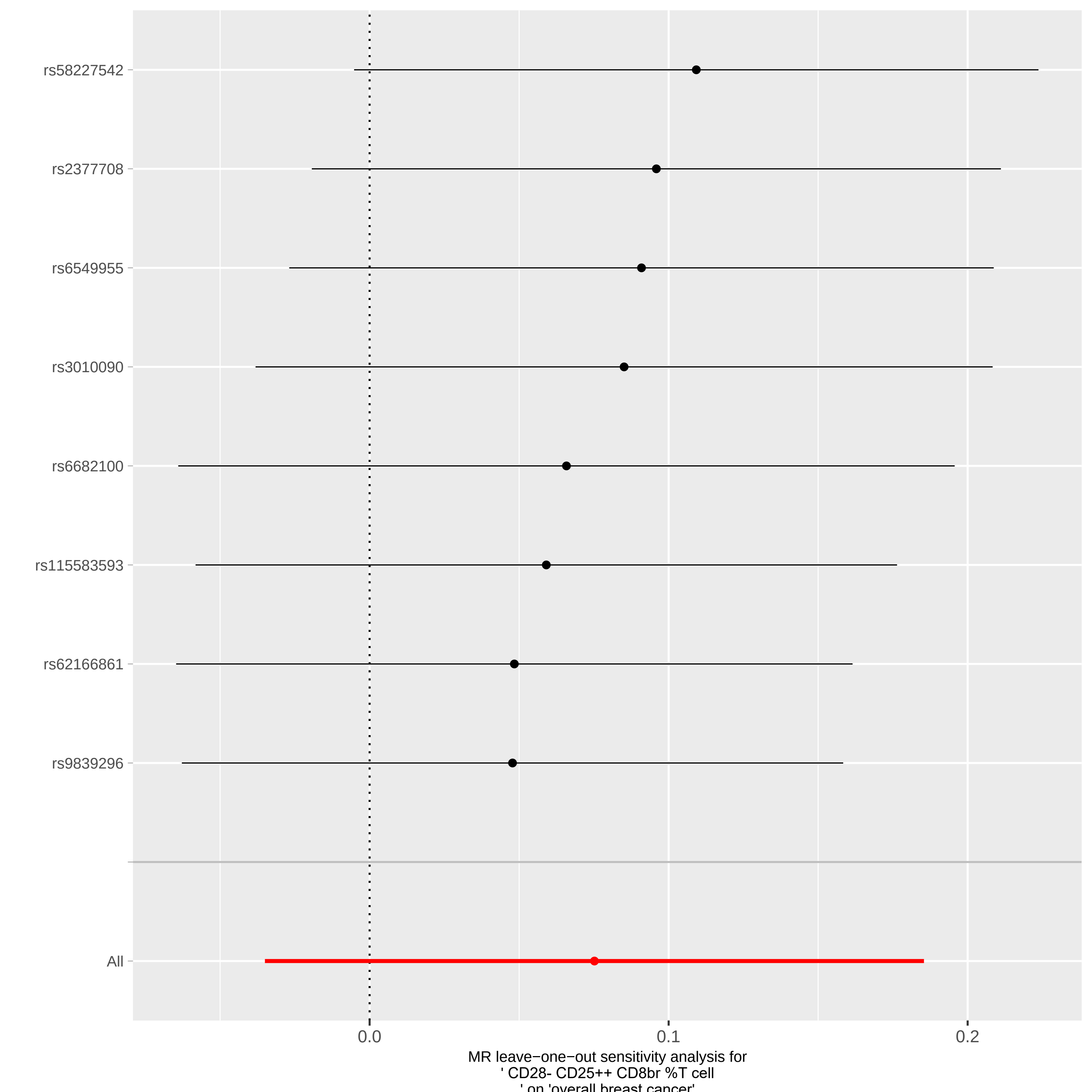

rs58227542
rs2377708
rs6549955
rs3010090
rs6682100
rs115583593
rs62166861
rs9839296
All
0.0
0.1
0.2
MR leave−one−out sensitivity analysis for
' CD28- CD25++ CD8br %T cell
' on 'overall breast cancer'

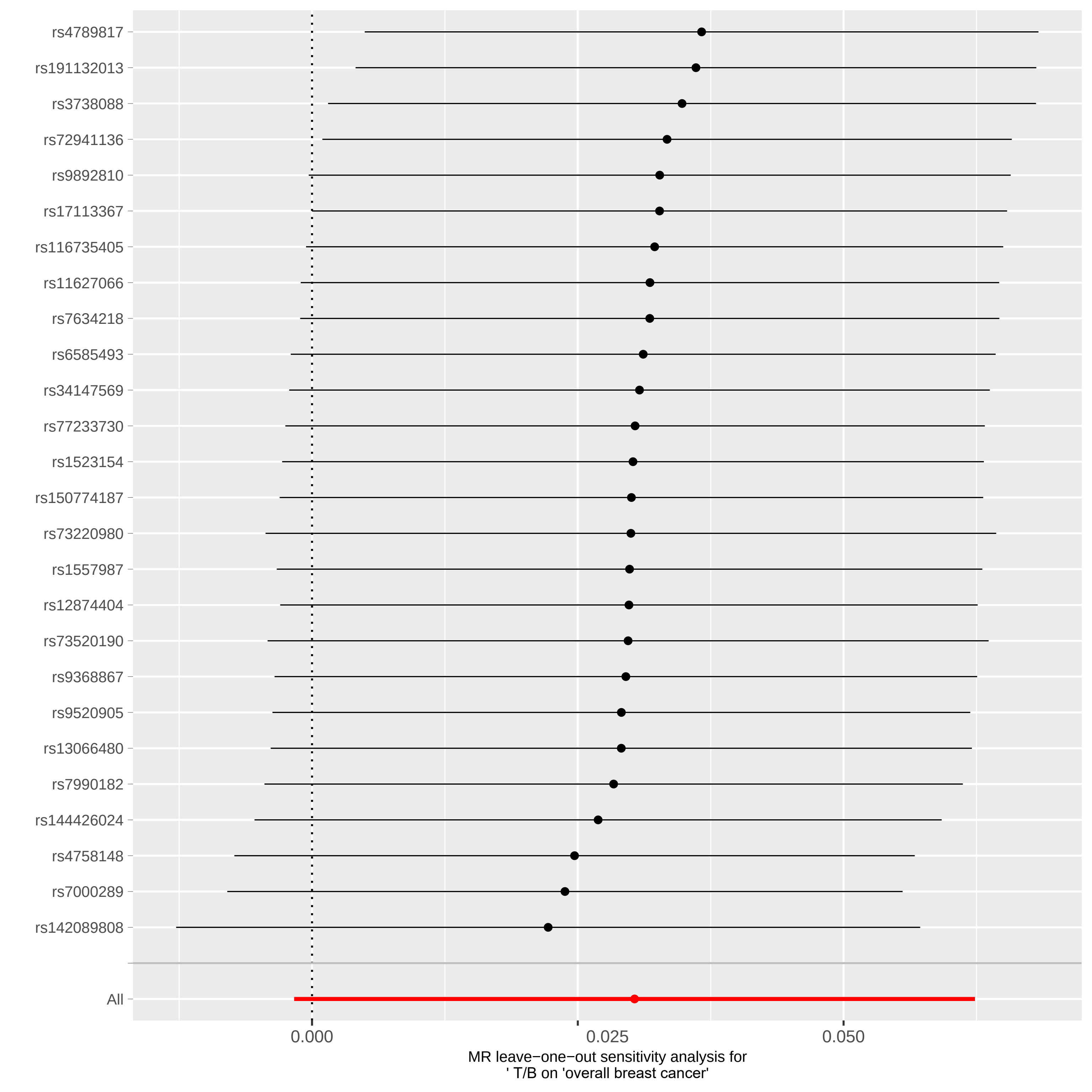

rs4789817
rs191132013
rs3738088
rs72941136
rs9892810
rs17113367
rs116735405
rs11627066
rs7634218
rs6585493
rs34147569
rs77233730
rs1523154
rs150774187
rs73220980
rs1557987
rs12874404
rs73520190
rs9368867
rs9520905
rs13066480
rs7990182
rs144426024
rs4758148
rs7000289
rs142089808
All
0.000
0.025
0.050
MR leave−one−out sensitivity analysis for
' T/B on 'overall breast cancer'

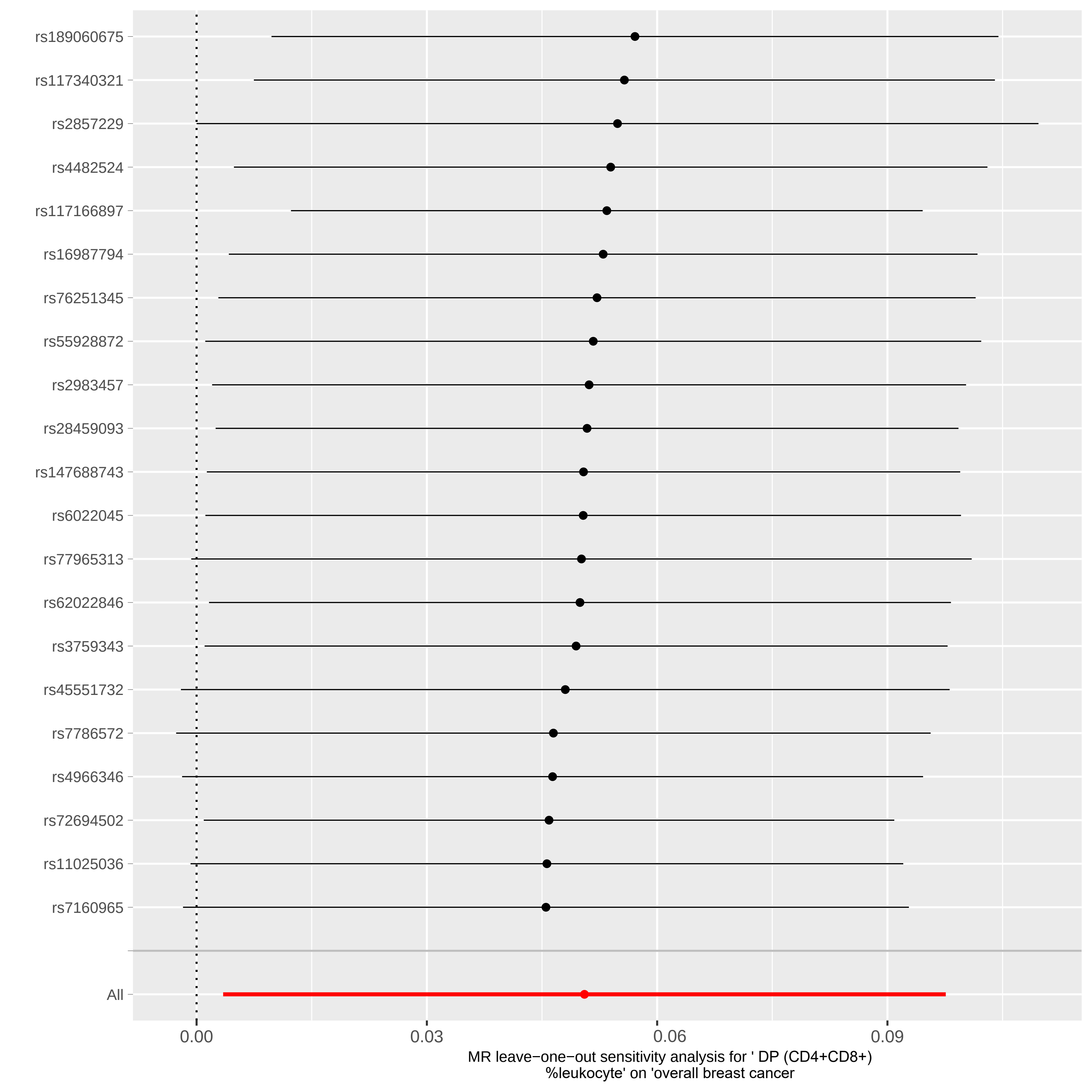

rs189060675
rs117340321
rs2857229
rs4482524
rs117166897
rs16987794
rs76251345
rs55928872
rs2983457
rs28459093
rs147688743
rs6022045
rs77965313
rs62022846
rs3759343
rs45551732
rs7786572
rs4966346
rs72694502
rs11025036
rs7160965
All
0.00
0.03
0.06
0.09
MR leave−one−out sensitivity analysis for ' DP (CD4+CD8+)
%leukocyte' on 'overall breast cancer

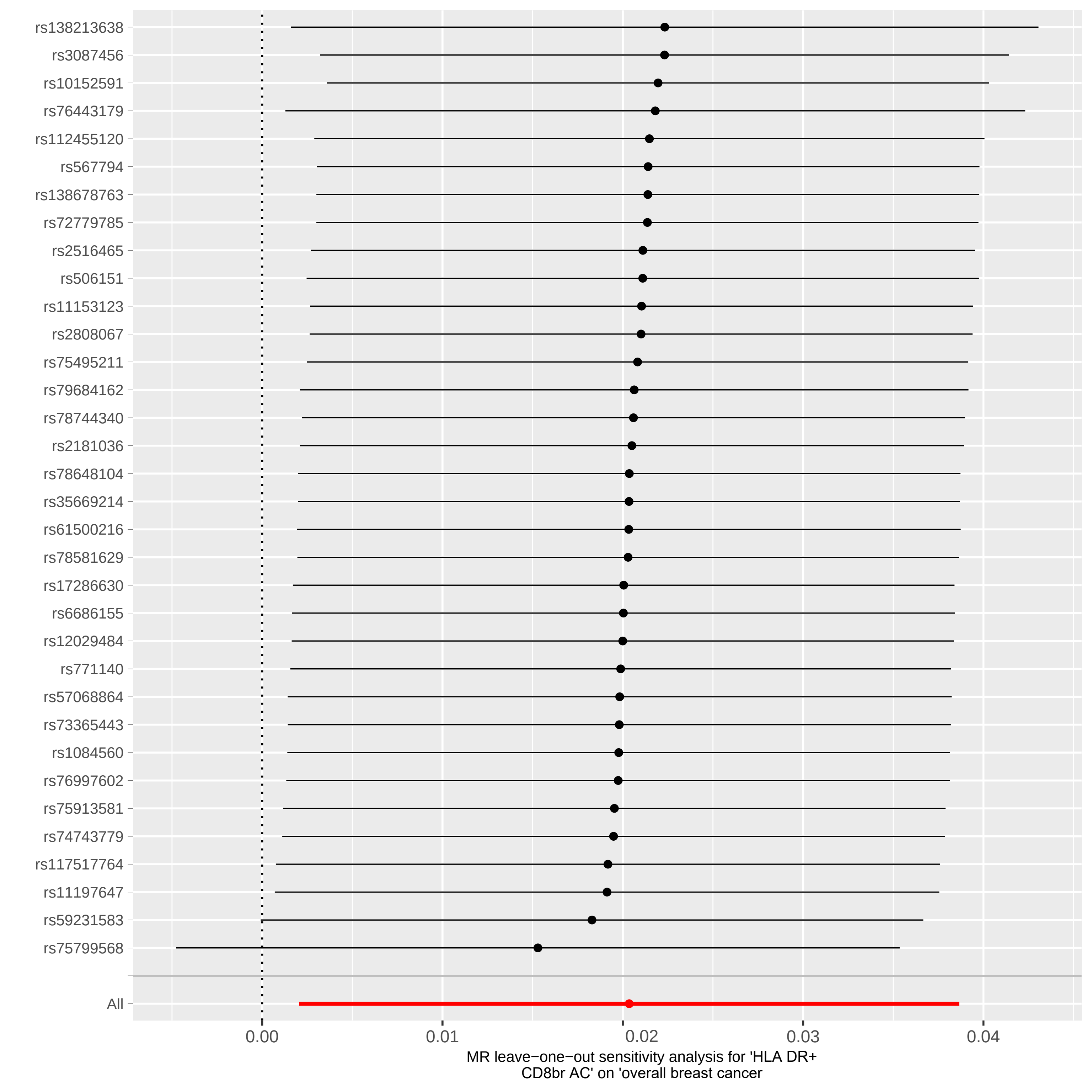

rs138213638
rs3087456
rs10152591
rs76443179
rs112455120
rs567794
rs138678763
rs72779785
rs2516465
rs506151
rs11153123
rs2808067
rs75495211
rs79684162
rs78744340
rs2181036
rs78648104
rs35669214
rs61500216
rs78581629
rs17286630
rs6686155
rs12029484
rs771140
rs57068864
rs73365443
rs1084560
rs76997602
rs75913581
rs74743779
rs117517764
rs11197647
rs59231583
rs75799568
All
0.00
0.01
0.02
0.03
0.04
MR leave−one−out sensitivity analysis for 'HLA DR+
CD8br AC' on 'overall breast cancer

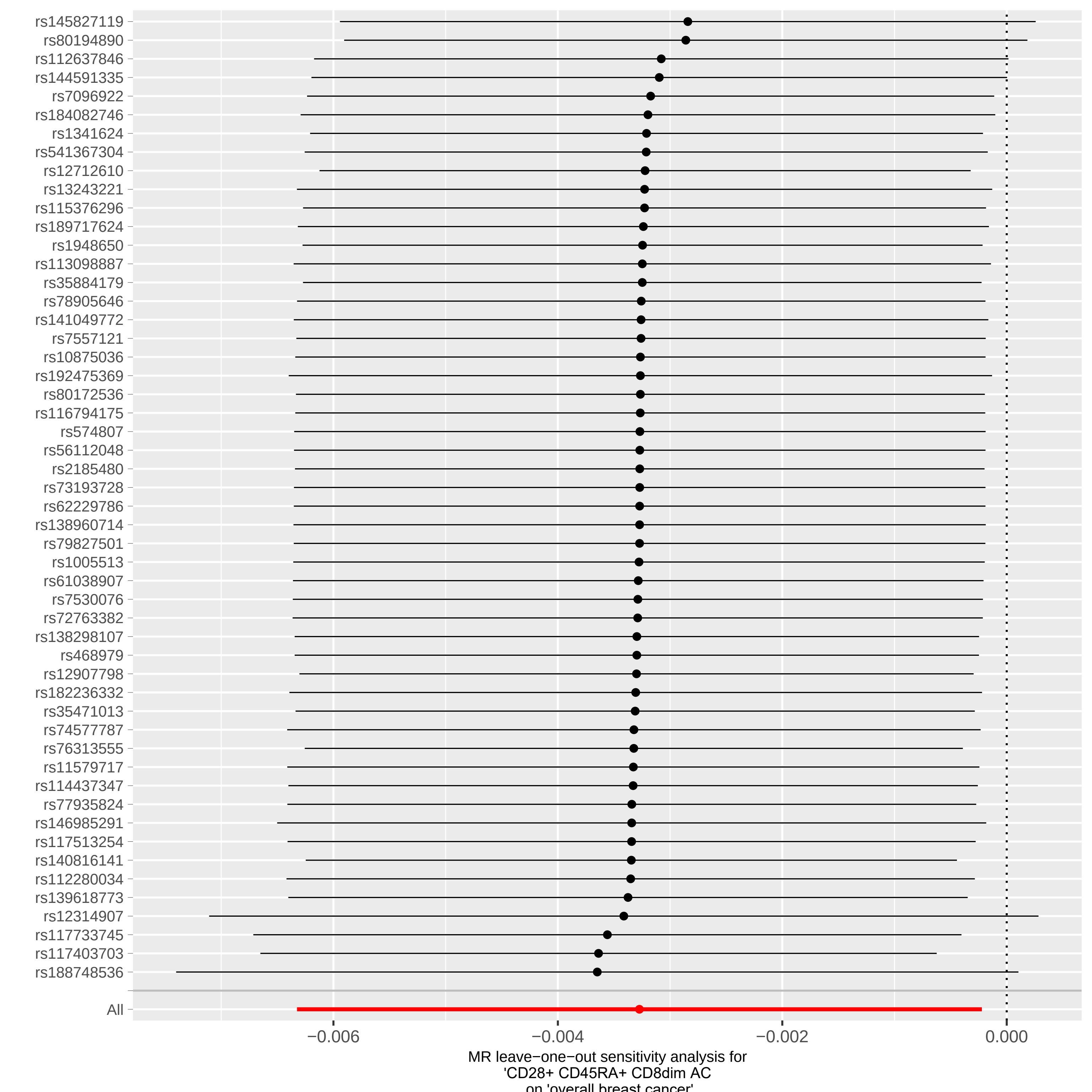
rs145827119
rs80194890
rs112637846
rs144591335
rs7096922
rs184082746
rs1341624
rs541367304
rs12712610
rs13243221
rs115376296
rs189717624
rs1948650
rs113098887
rs35884179
rs78905646
rs141049772
rs7557121
rs10875036
rs192475369
rs80172536
rs116794175
rs574807
rs56112048
rs2185480
rs73193728
rs62229786
rs138960714
rs79827501
rs1005513
rs61038907
rs7530076
rs72763382
rs138298107
rs468979
rs12907798
rs182236332
rs35471013
rs74577787
rs76313555
rs11579717
rs114437347
rs77935824
rs146985291
rs117513254
rs140816141
rs112280034
rs139618773
rs12314907
rs117733745
rs117403703
rs188748536
All
−0.006
−0.004
−0.002
0.000
MR leave−one−out sensitivity analysis for
'CD28+ CD45RA+ CD8dim AC
on 'overall breast cancer'

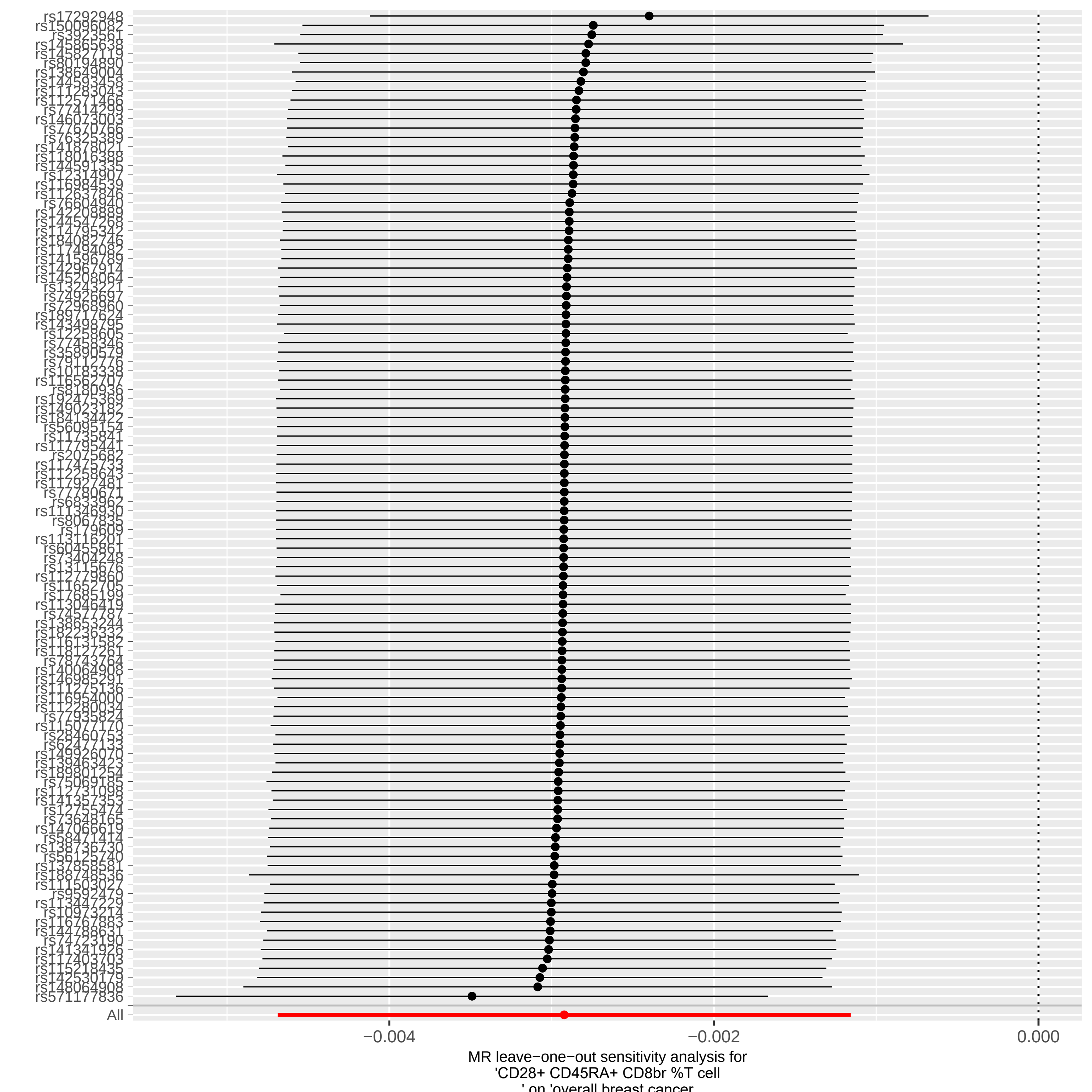

rs17292948
rs571177836
All
−0.004
−0.002
0.000
MR leave−one−out sensitivity analysis for
'CD28+ CD45RA+ CD8br %T cell
' on 'overall breast cancer

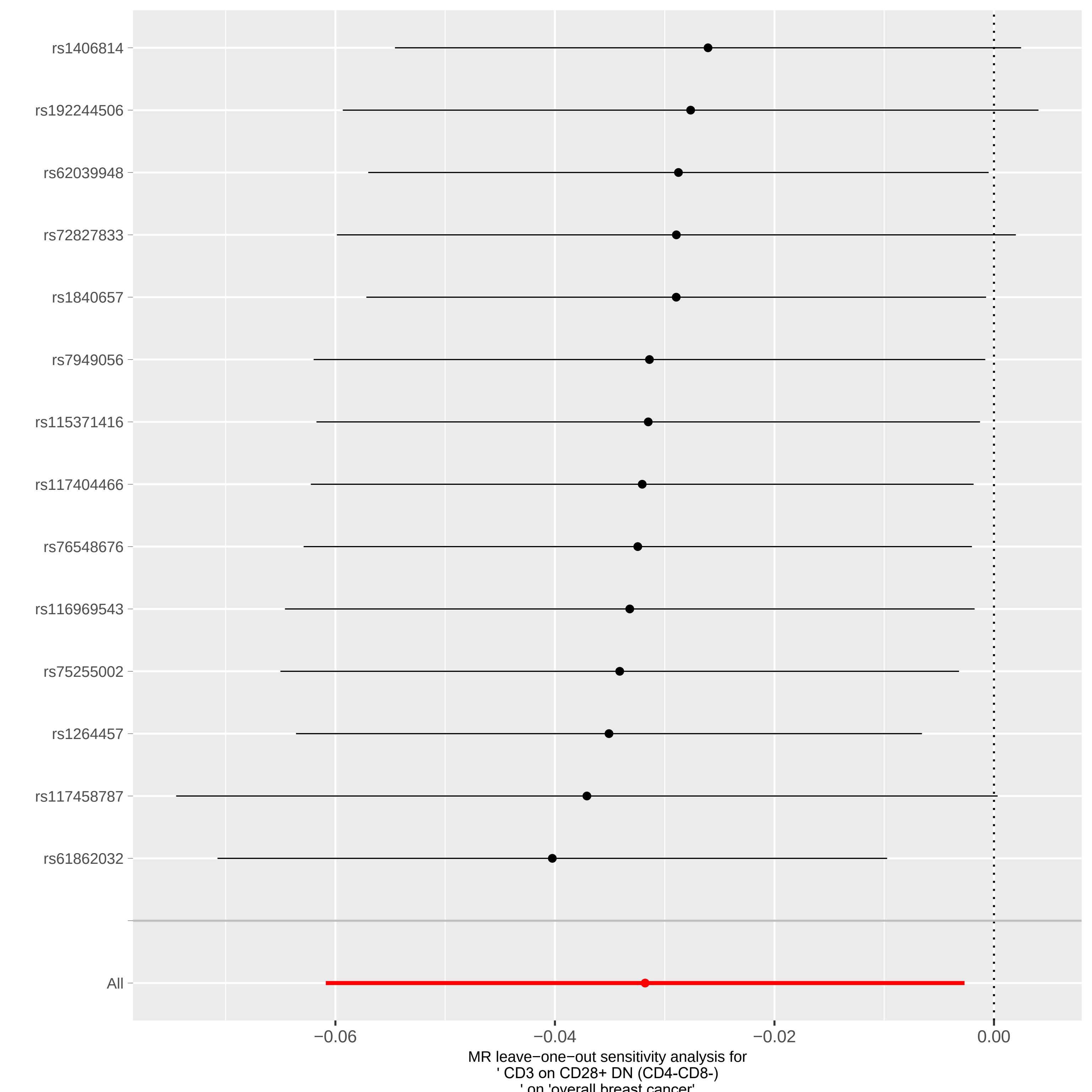
rs1406814
rs192244506
rs62039948
rs72827833
rs1840657
rs7949056
rs115371416
rs117404466
rs76548676
rs116969543
rs75255002
rs1264457
rs117458787
rs61862032
All
−0.06
−0.04
−0.02
0.00
MR leave−one−out sensitivity analysis for
' CD3 on CD28+ DN (CD4-CD8-)
' on 'overall breast cancer'

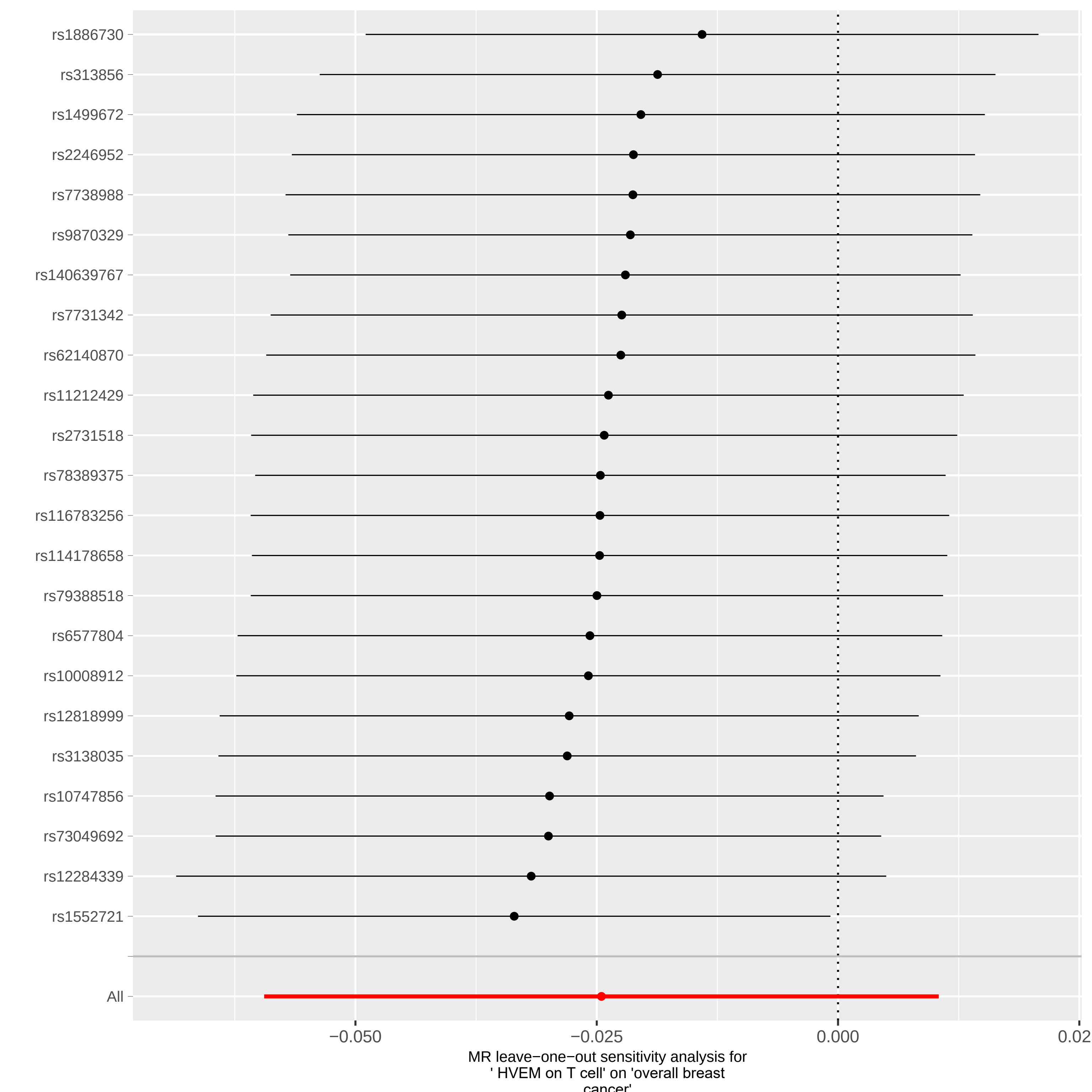
rs1886730
rs313856
rs1499672
rs2246952
rs7738988
rs9870329
rs140639767
rs7731342
rs62140870
rs11212429
rs2731518
rs78389375
rs116783256
rs114178658
rs79388518
rs6577804
rs10008912
rs12818999
rs3138035
rs10747856
rs73049692
rs12284339
rs1552721
All
−0.050
−0.025
0.000
0.02
MR leave−one−out sensitivity analysis for
' HVEM on T cell' on 'overall breast
cancer'

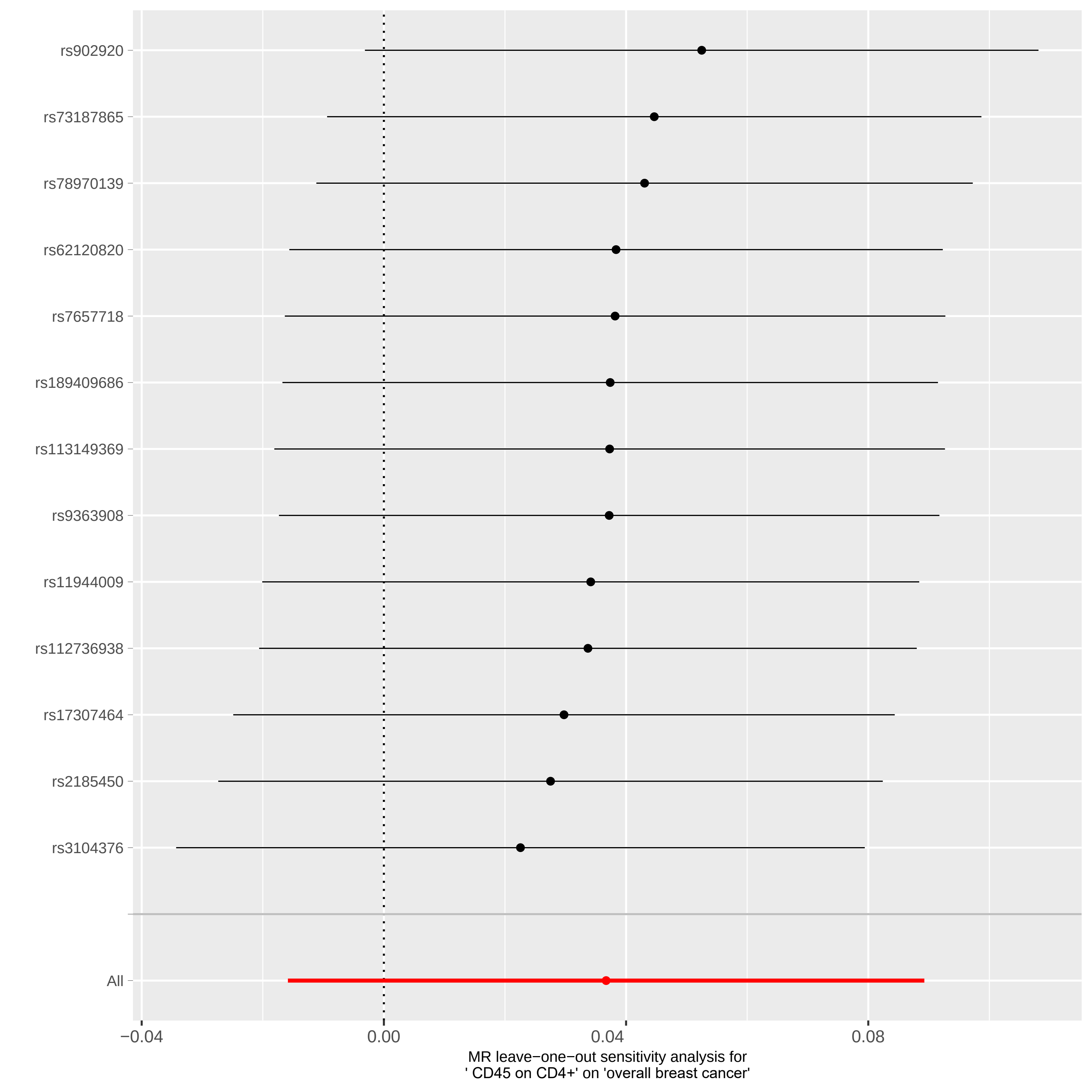

rs902920
rs73187865
rs78970139
rs62120820
rs7657718
rs189409686
rs113149369
rs9363908
rs11944009
rs112736938
rs17307464
rs2185450
rs3104376
All
−0.04
0.00
0.04
0.08
MR leave−one−out sensitivity analysis for
' CD45 on CD4+' on 'overall breast cancer'

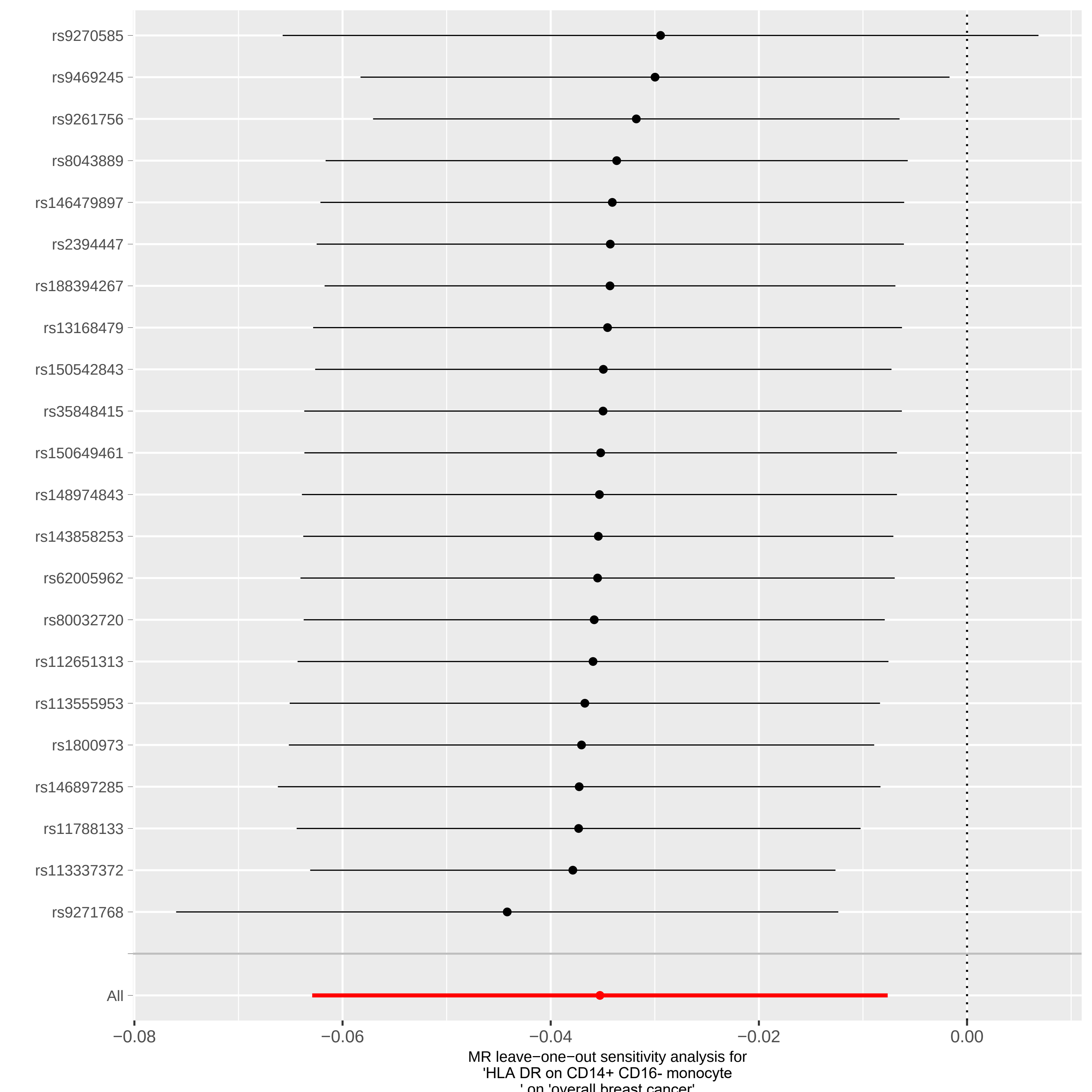
rs9270585
rs9469245
rs9261756
rs8043889
rs146479897
rs2394447
rs188394267
rs13168479
rs150542843
rs35848415
rs150649461
rs148974843
rs143858253
rs62005962
rs80032720
rs112651313
rs113555953
rs1800973
rs146897285
rs11788133
rs113337372
rs9271768
All
−0.08
−0.06
−0.04
−0.02
0.00
MR leave−one−out sensitivity analysis for
'HLA DR on CD14+ CD16- monocyte
' on 'overall breast cancer'

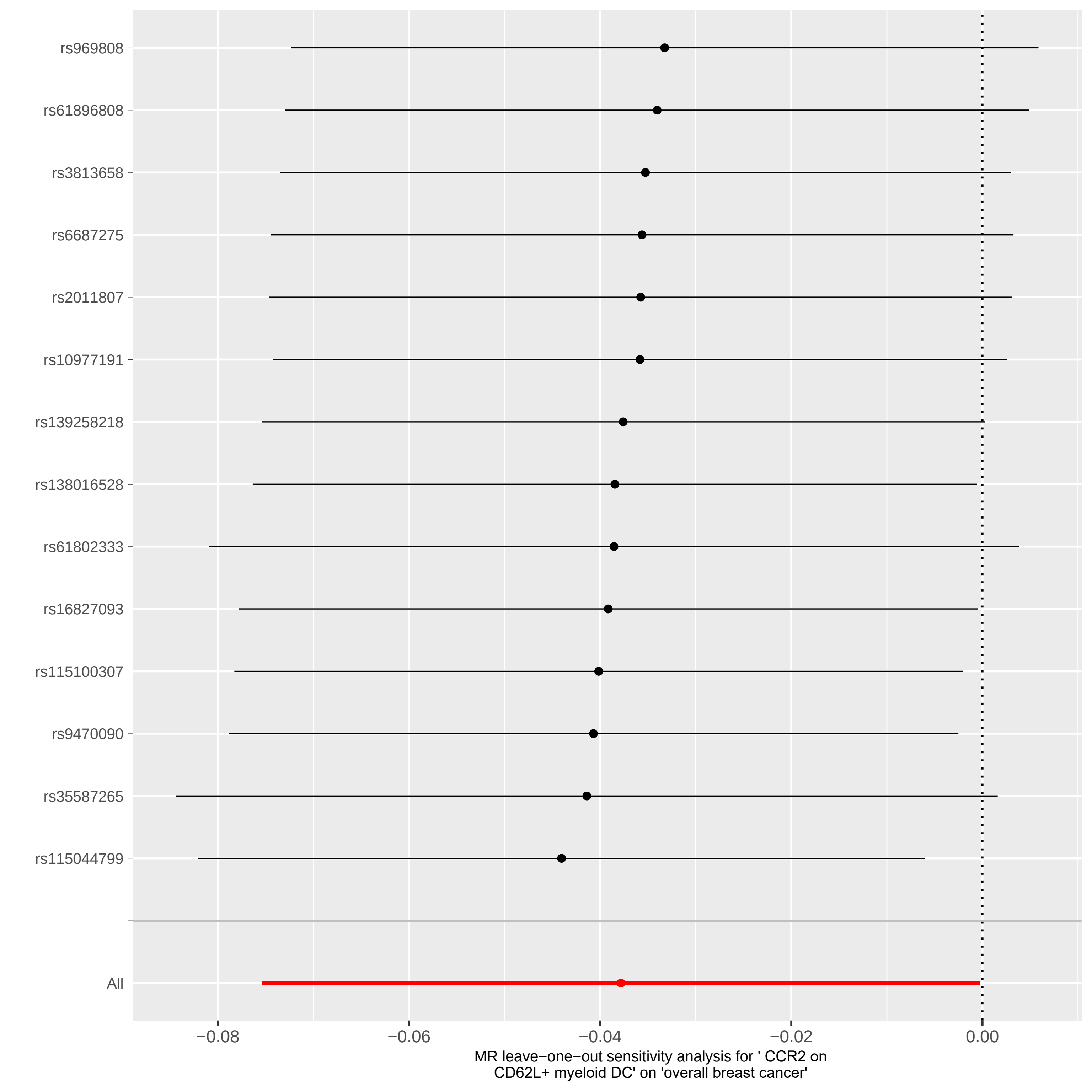
rs969808
rs61896808
rs3813658
rs6687275
rs2011807
rs10977191
rs139258218
rs138016528
rs61802333
rs16827093
rs115100307
rs9470090
rs35587265
rs115044799
All
−0.08
−0.06
−0.04
−0.02
0.00
MR leave−one−out sensitivity analysis for ' CCR2 on
CD62L+ myeloid DC' on 'overall breast cancer'

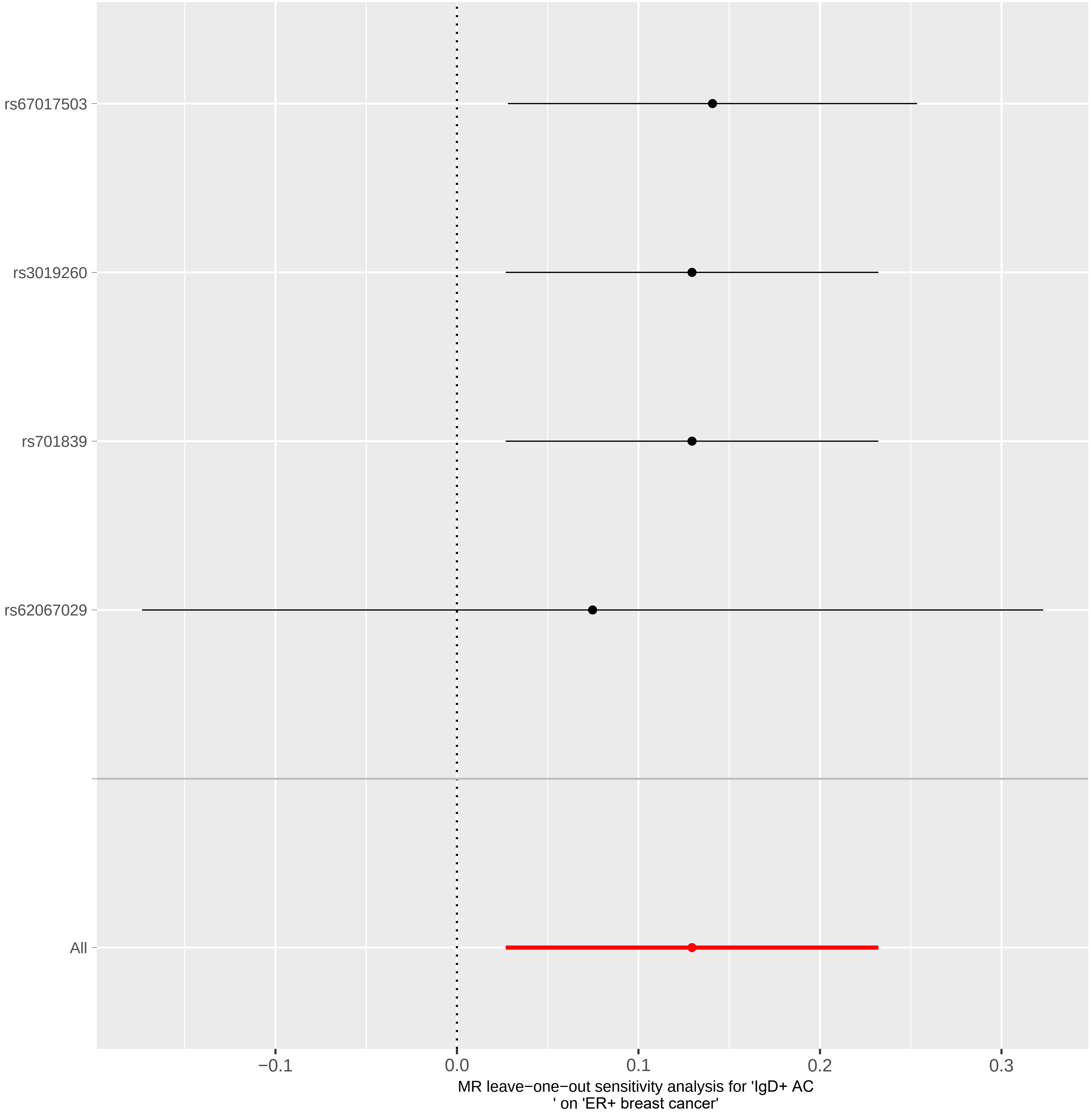

rs67017503
rs3019260
rs701839
rs62067029
All
−0.1
0.0
0.1
0.2
0.3
MR leave−one−out sensitivity analysis for 'IgD+ AC
' on 'ER+ breast cancer'

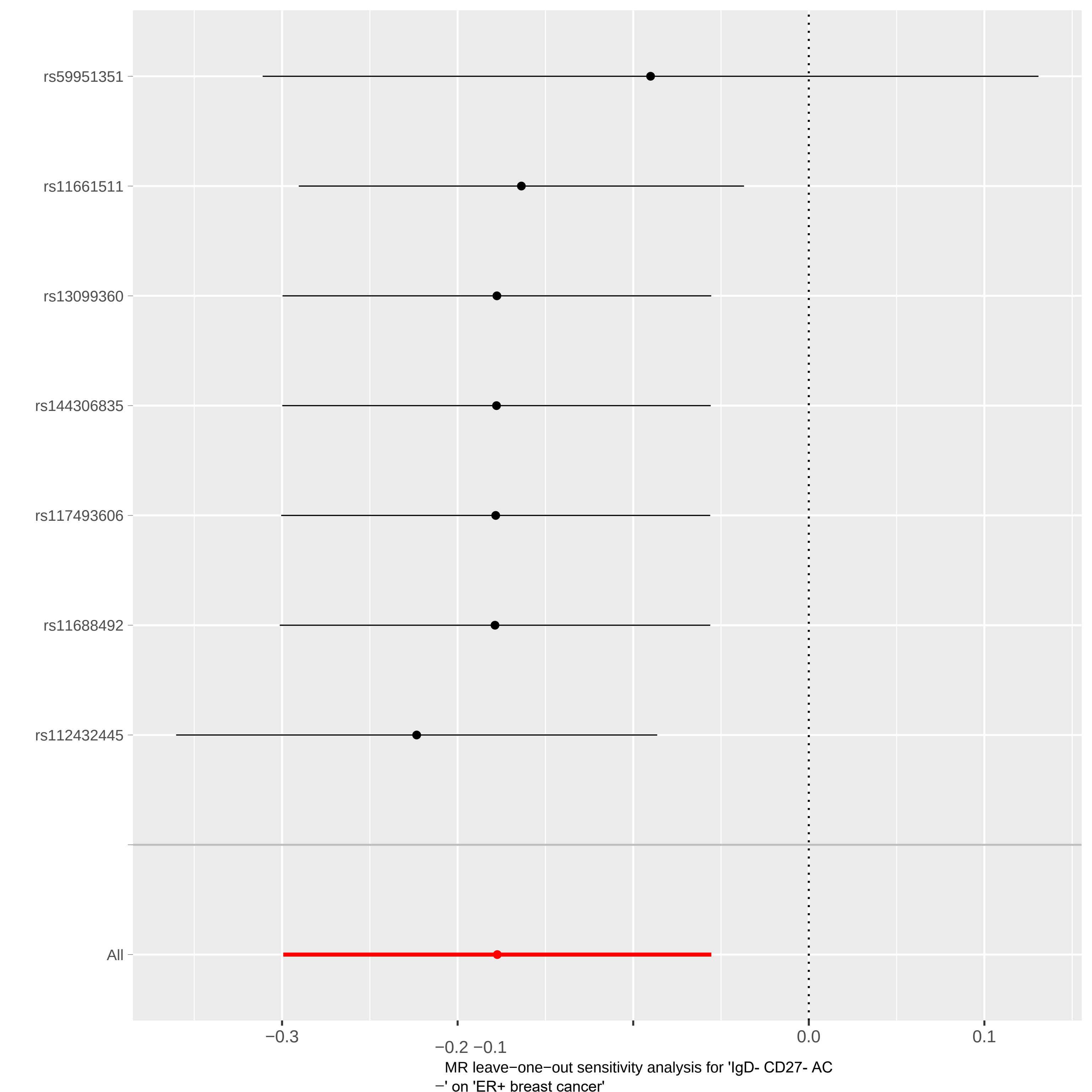
rs59951351
rs11661511
rs13099360
rs144306835
rs117493606
rs11688492
rs112432445
All
−0.3
−0.2 −0.1
0.0
0.1
MR leave−one−out sensitivity analysis for 'IgD- CD27- AC
−' on 'ER+ breast cancer'

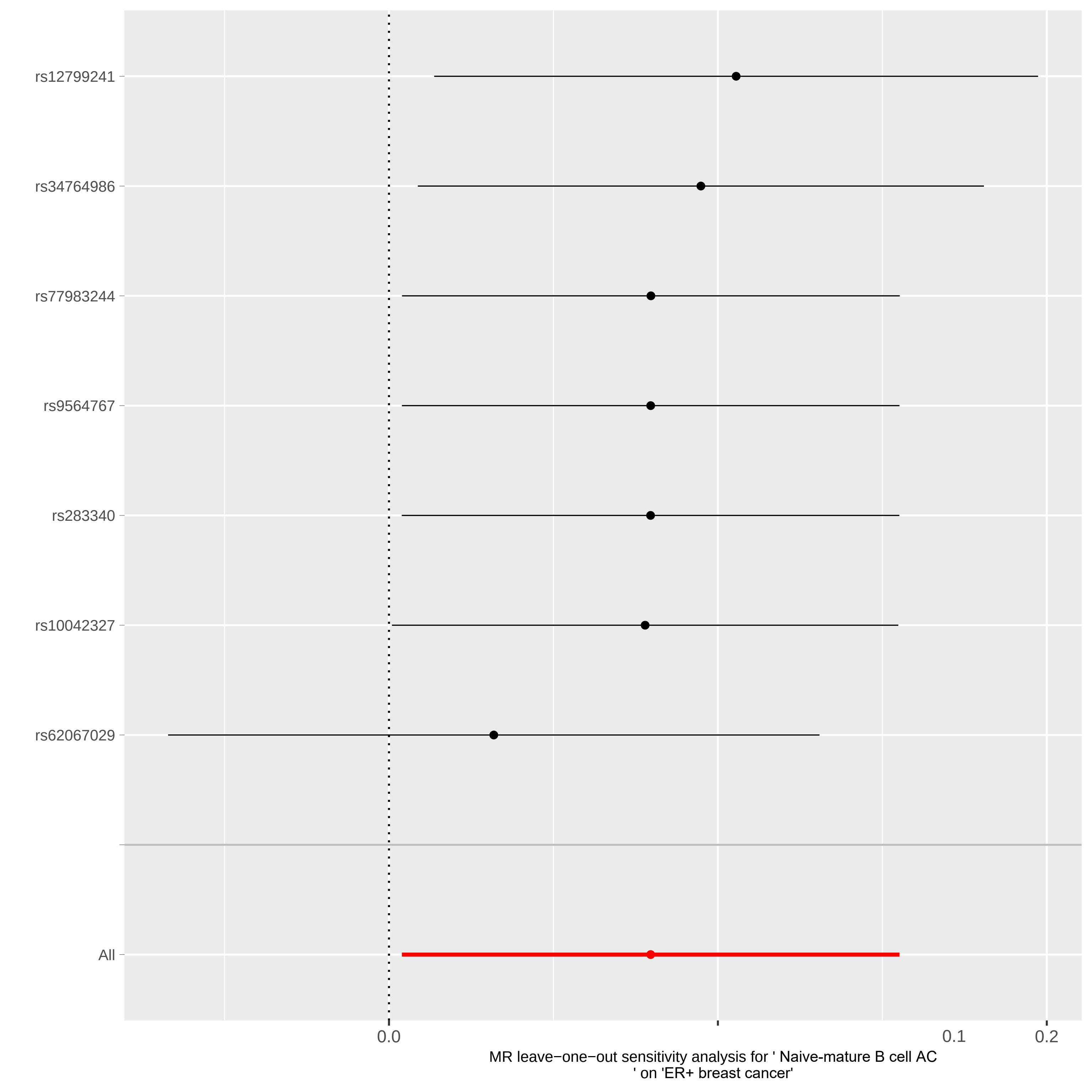
rs12799241
rs34764986
rs77983244
rs9564767
rs283340
rs10042327
rs62067029
All
0.0
0.1
0.2
MR leave−one−out sensitivity analysis for ' Naive-mature B cell AC
' on 'ER+ breast cancer'

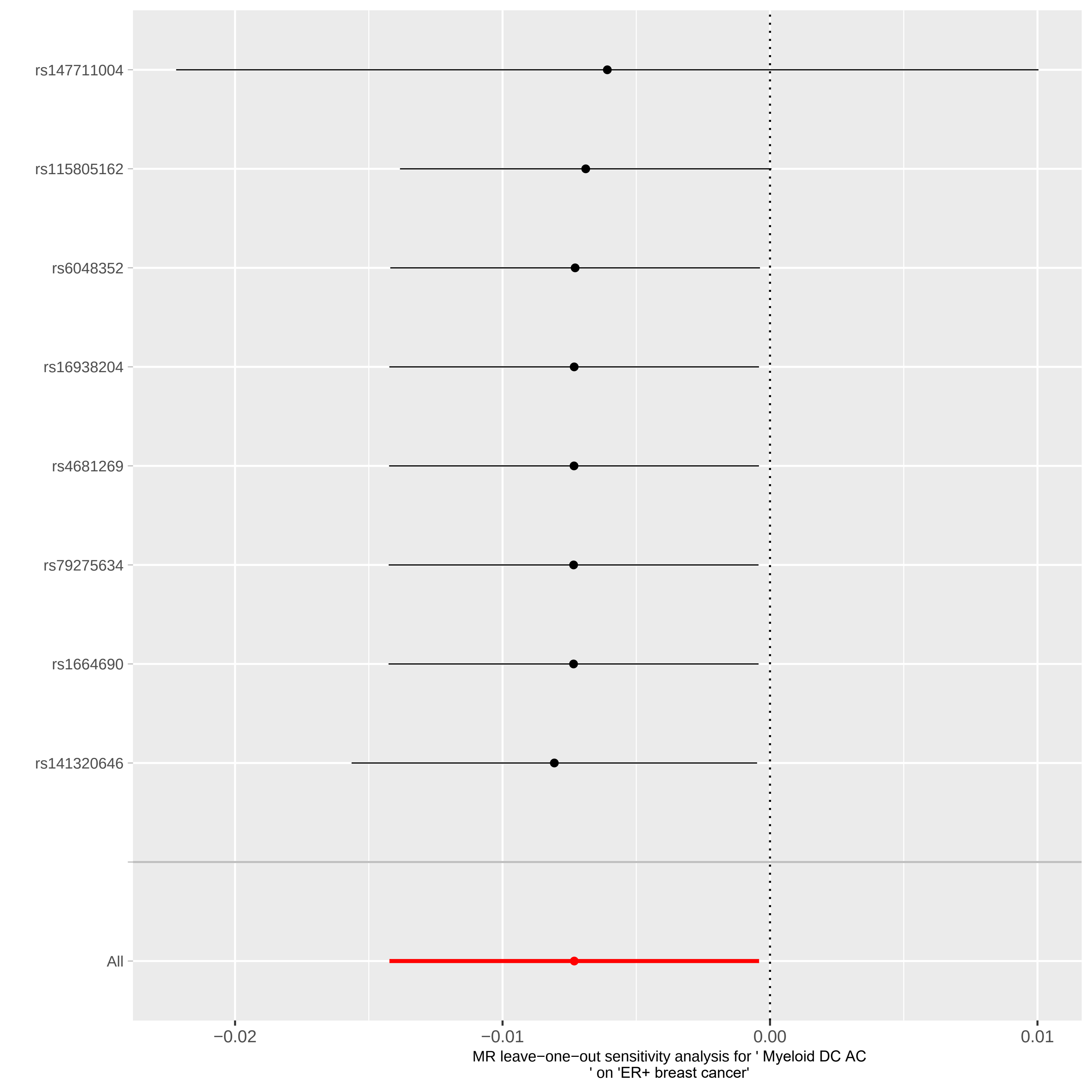
rs147711004
rs115805162
rs6048352
rs16938204
rs4681269
rs79275634
rs1664690
rs141320646
All
−0.02
−0.01
0.00
0.01
MR leave−one−out sensitivity analysis for ' Myeloid DC AC
' on 'ER+ breast cancer'

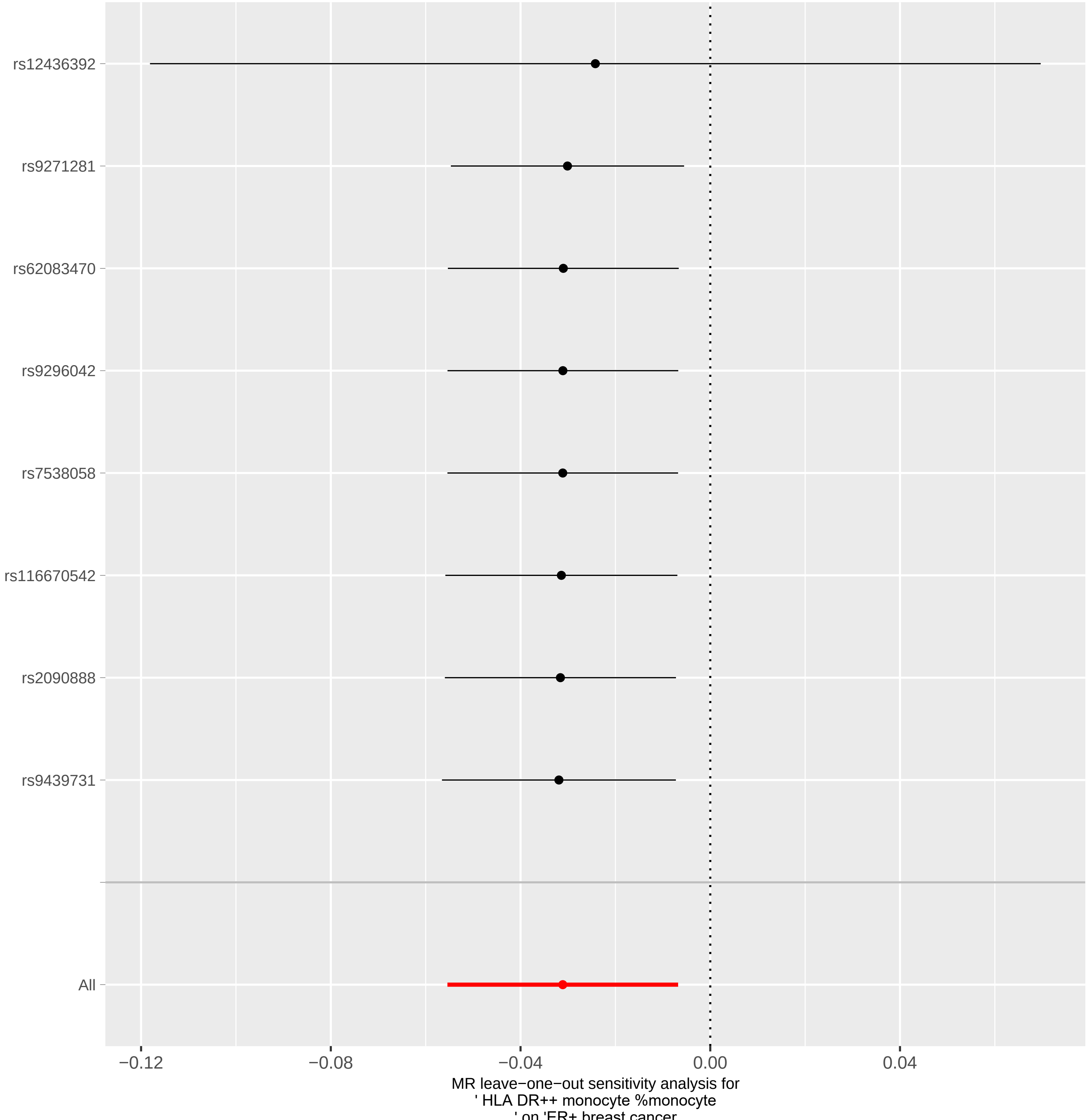
rs12436392
rs9271281
rs62083470
rs9296042
rs7538058
rs116670542
rs2090888
rs9439731
All
−0.12
−0.08
−0.04
0.00
0.04
MR leave−one−out sensitivity analysis for
' HLA DR++ monocyte %monocyte
' on 'ER+ breast cancer

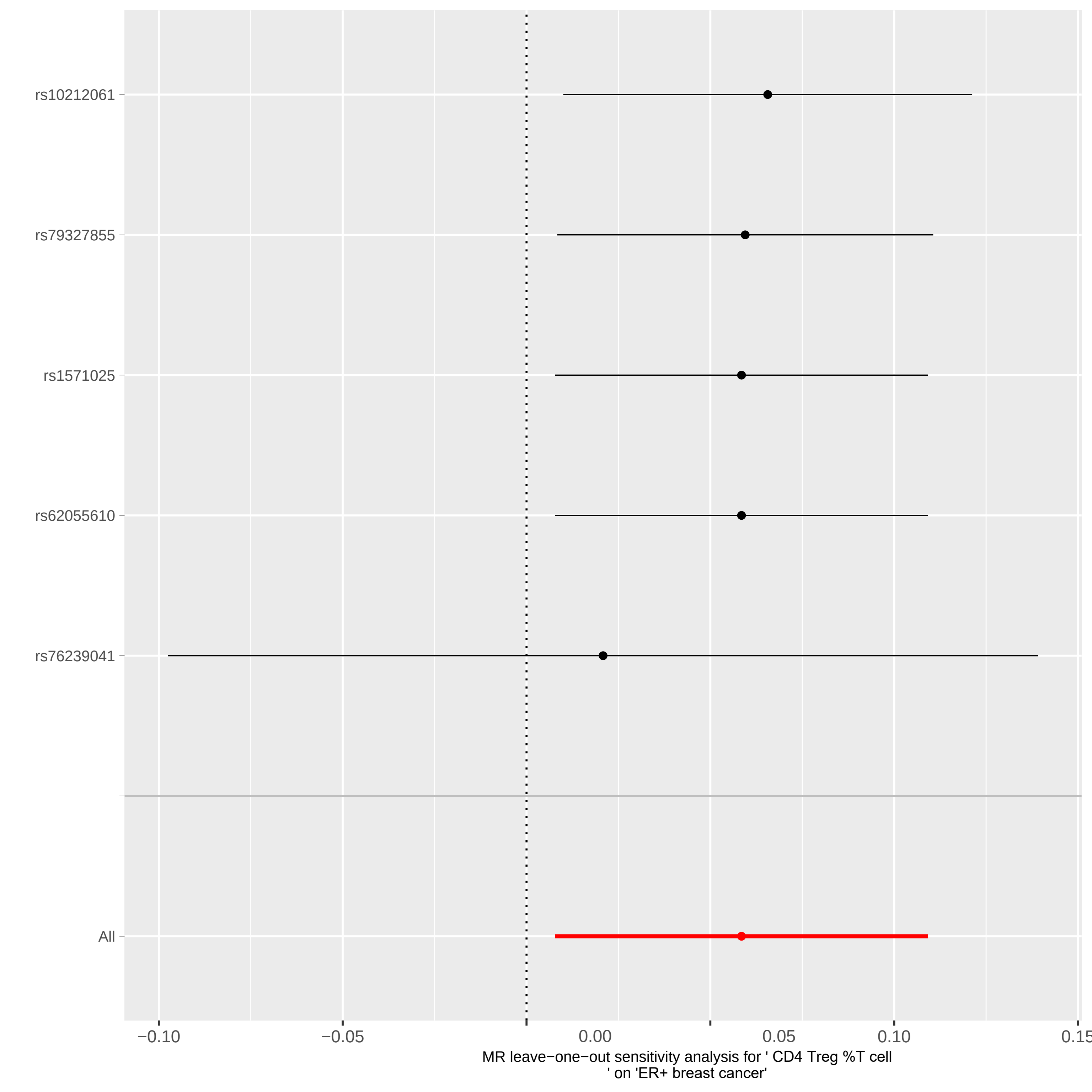
rs10212061
rs79327855
rs1571025
rs62055610
rs76239041
All
−0.10
−0.05
0.00
0.05
0.10
0.15
MR leave−one−out sensitivity analysis for ' CD4 Treg %T cell
' on 'ER+ breast cancer'

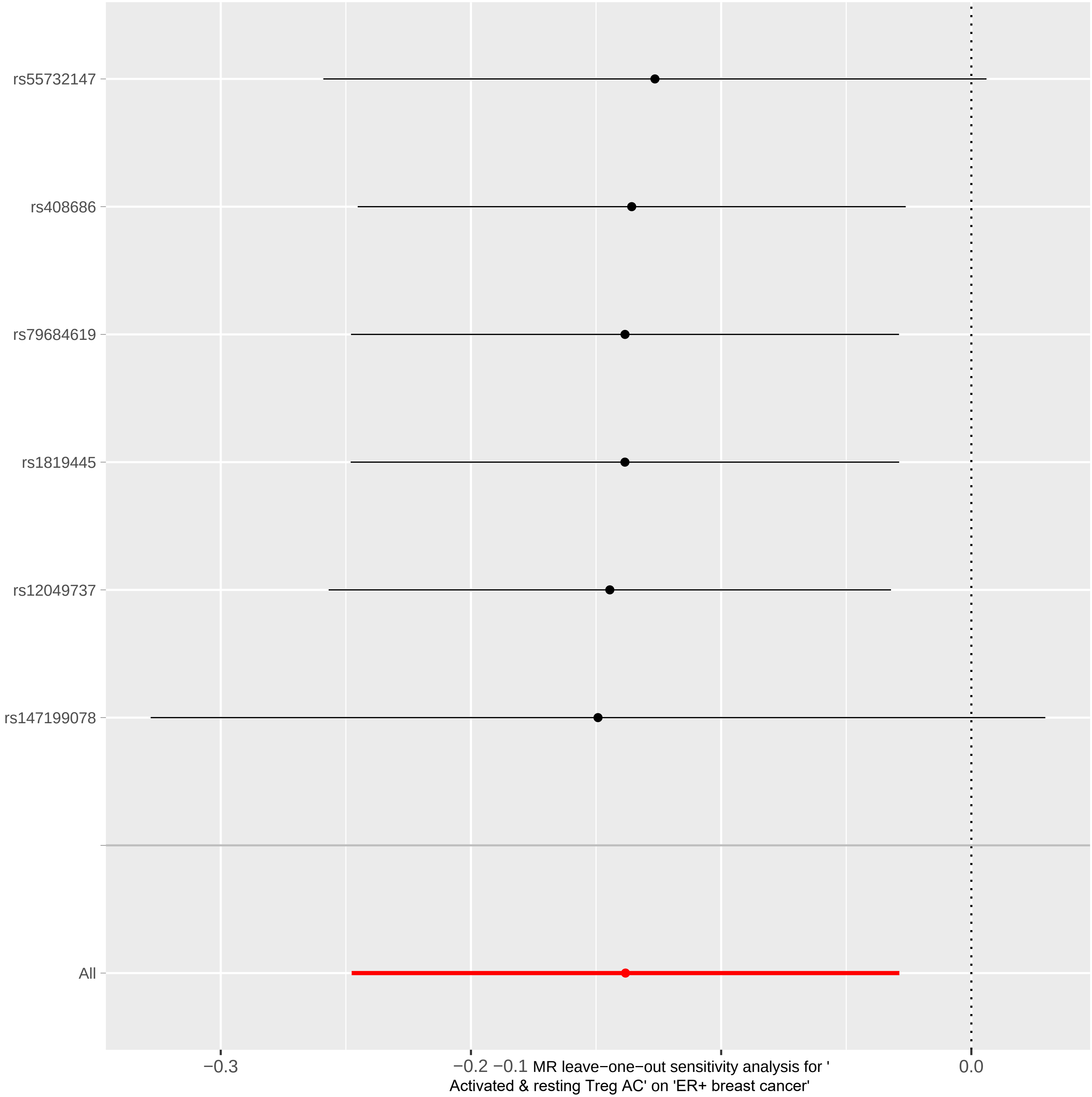
rs55732147
rs408686
rs79684619
rs1819445
rs12049737
rs147199078
All
−0.3
−0.2
−0.1
0.0
MR leave−one−out sensitivity analysis for '
Activated & resting Treg AC' on 'ER+ breast cancer'

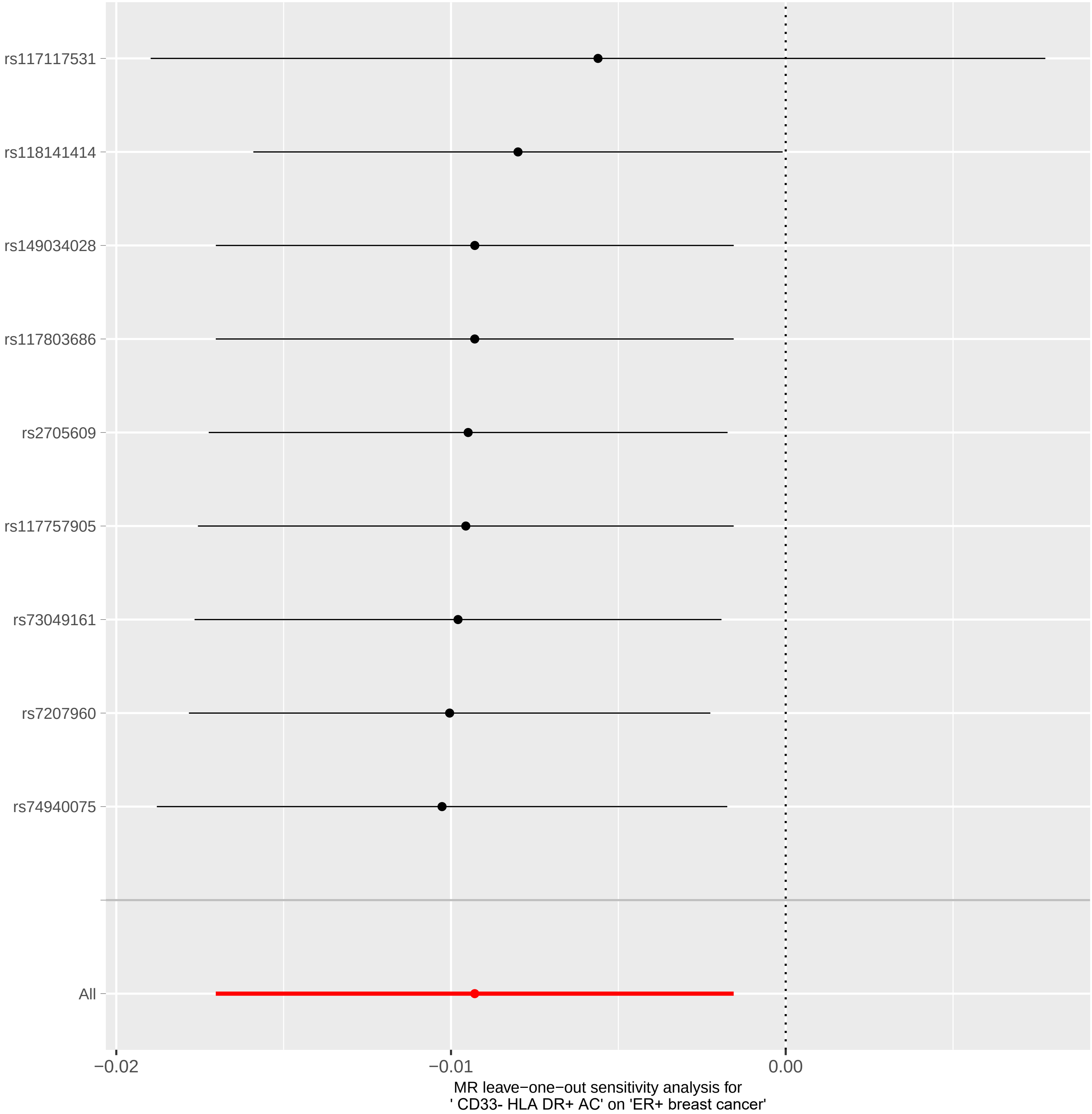

rs117117531
rs118141414
rs149034028
rs117803686
rs2705609
rs117757905
rs73049161
rs7207960
rs74940075
All
−0.02
−0.01
0.00
MR leave−one−out sensitivity analysis for
' CD33- HLA DR+ AC' on 'ER+ breast cancer'

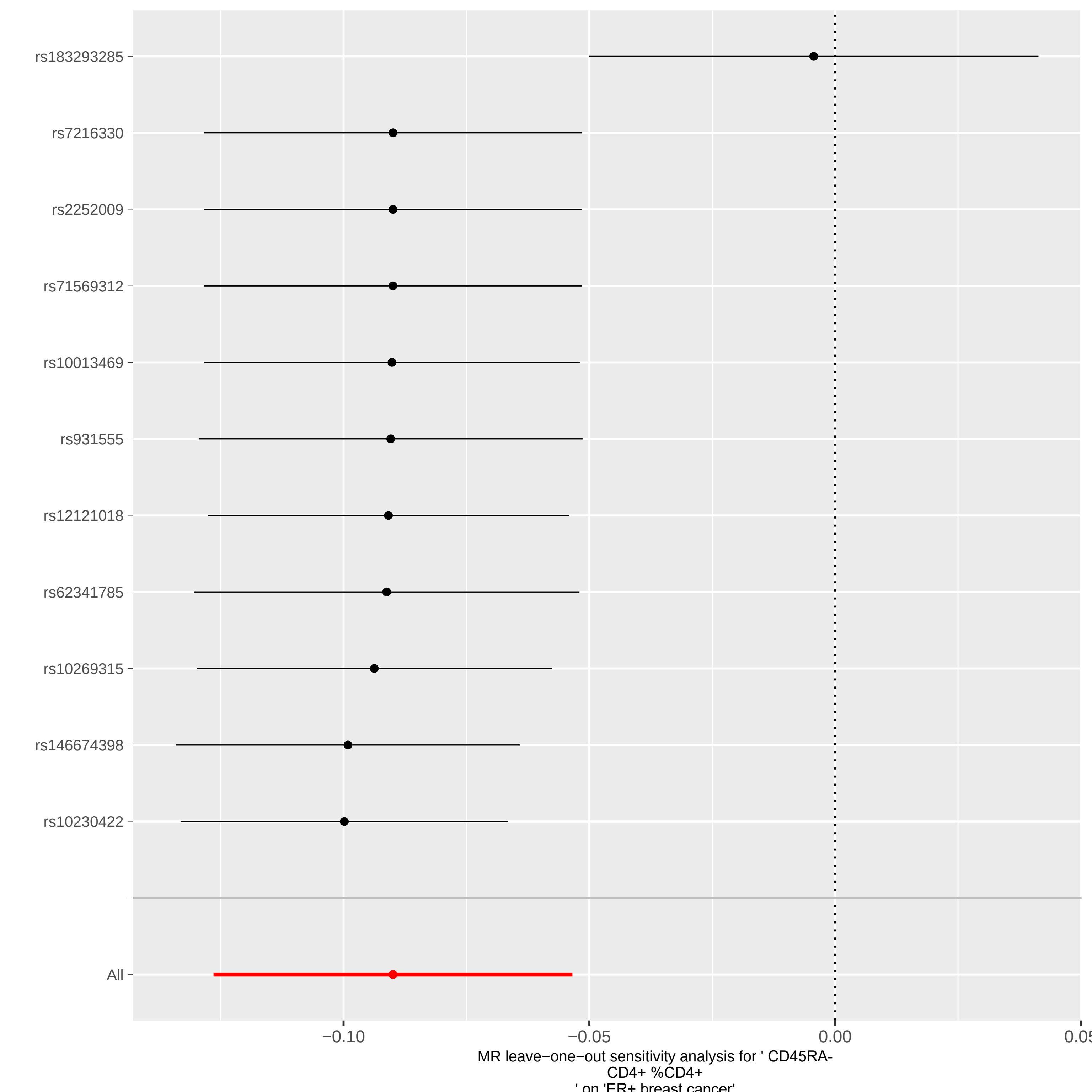

rs183293285
rs7216330
rs2252009
rs71569312
rs10013469
rs931555
rs12121018
rs62341785
rs10269315
rs146674398
rs10230422
All
−0.10
−0.05
0.00
MR leave−one−out sensitivity analysis for ' CD45RA- CD4+ %CD4+ ' on 'ER+ breast cancer'

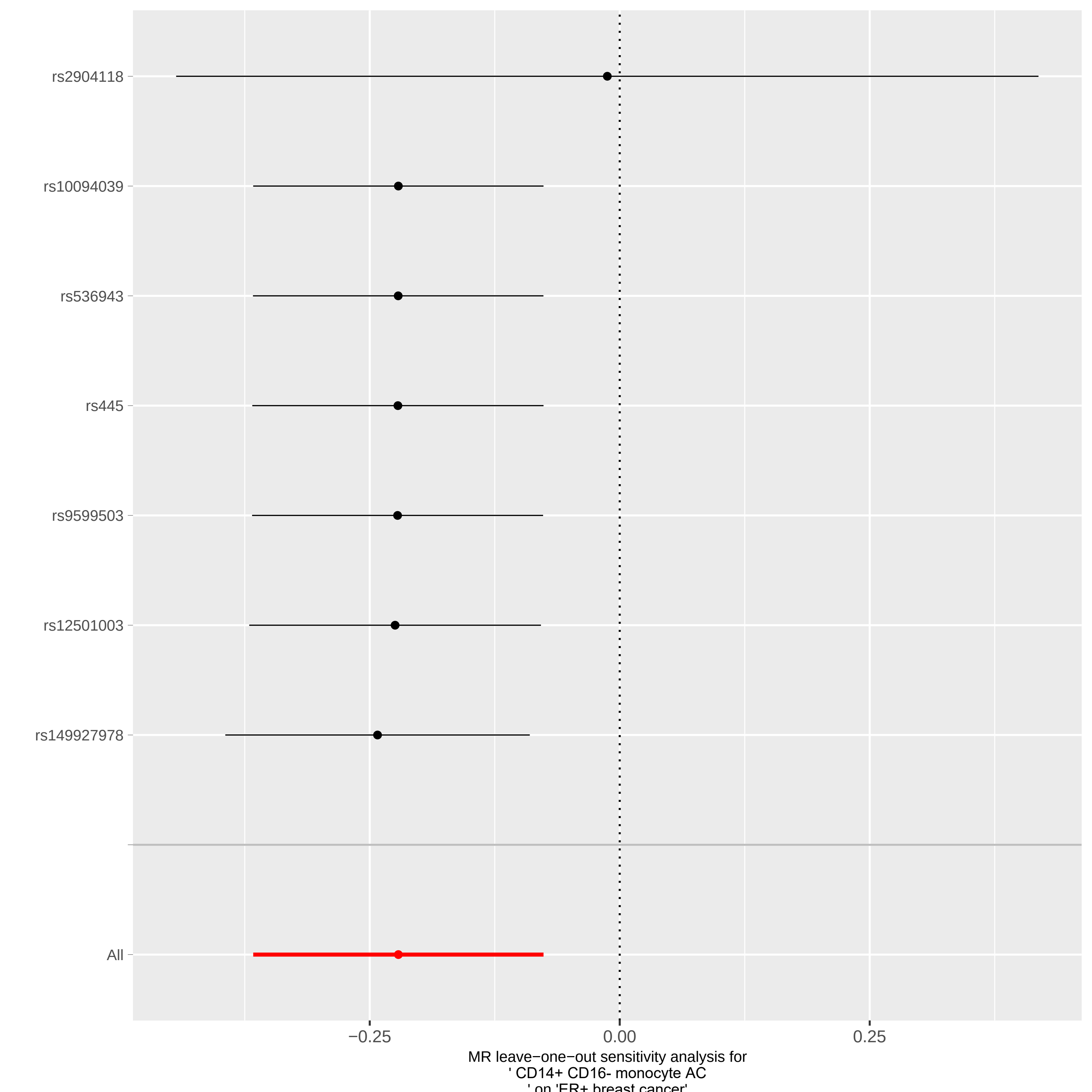
rs2904118
rs10094039
rs536943
rs445
rs9599503
rs12501003
rs149927978
All
−0.25
0.00
0.25
MR leave−one−out sensitivity analysis for
' CD14+ CD16- monocyte AC
' on 'ER+ breast cancer'

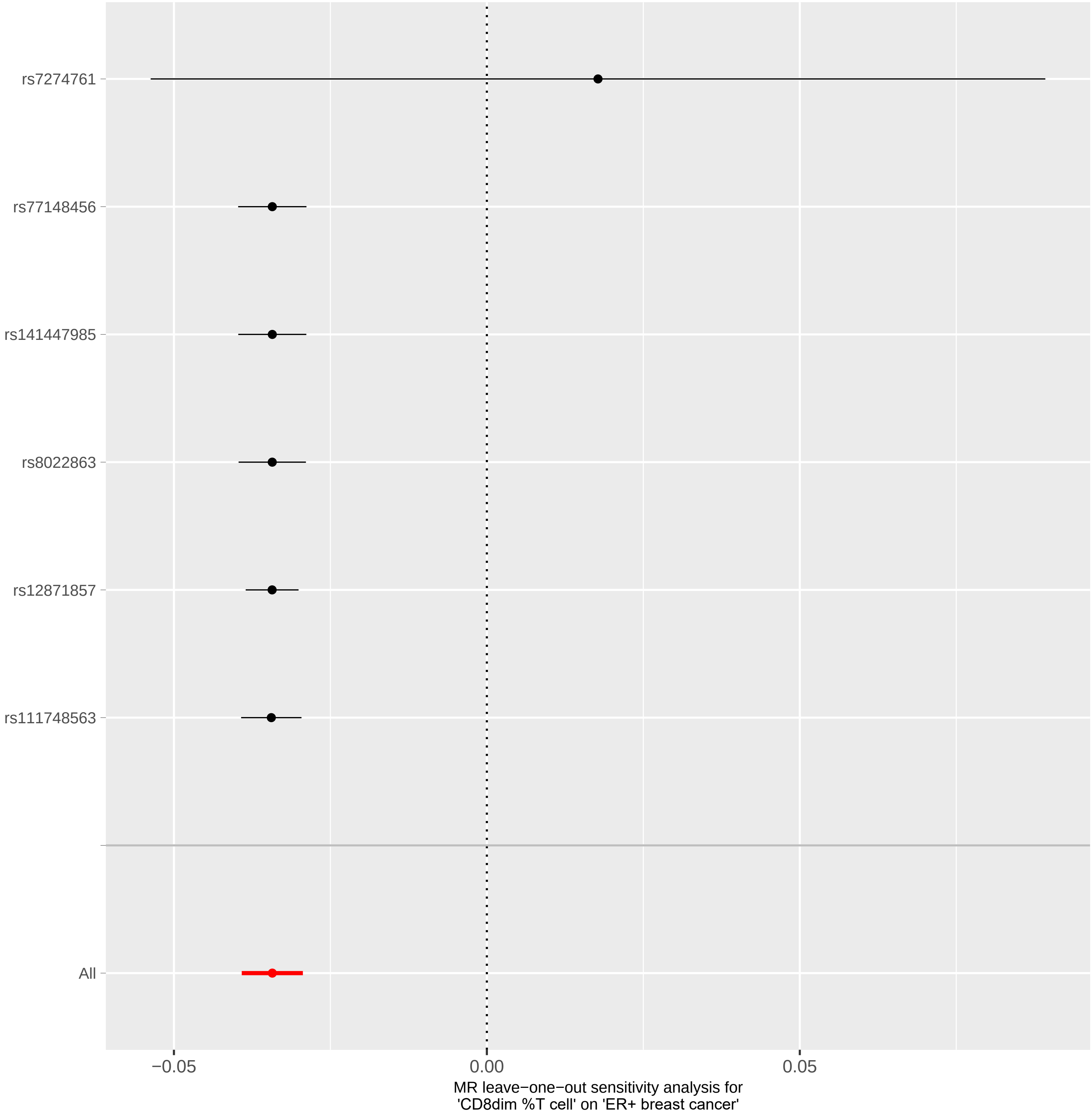
rs7274761
rs77148456
rs141447985
rs8022863
rs12871857
rs111748563
All
−0.05
0.00
0.05
MR leave−one−out sensitivity analysis for
'CD8dim %T cell' on 'ER+ breast cancer'

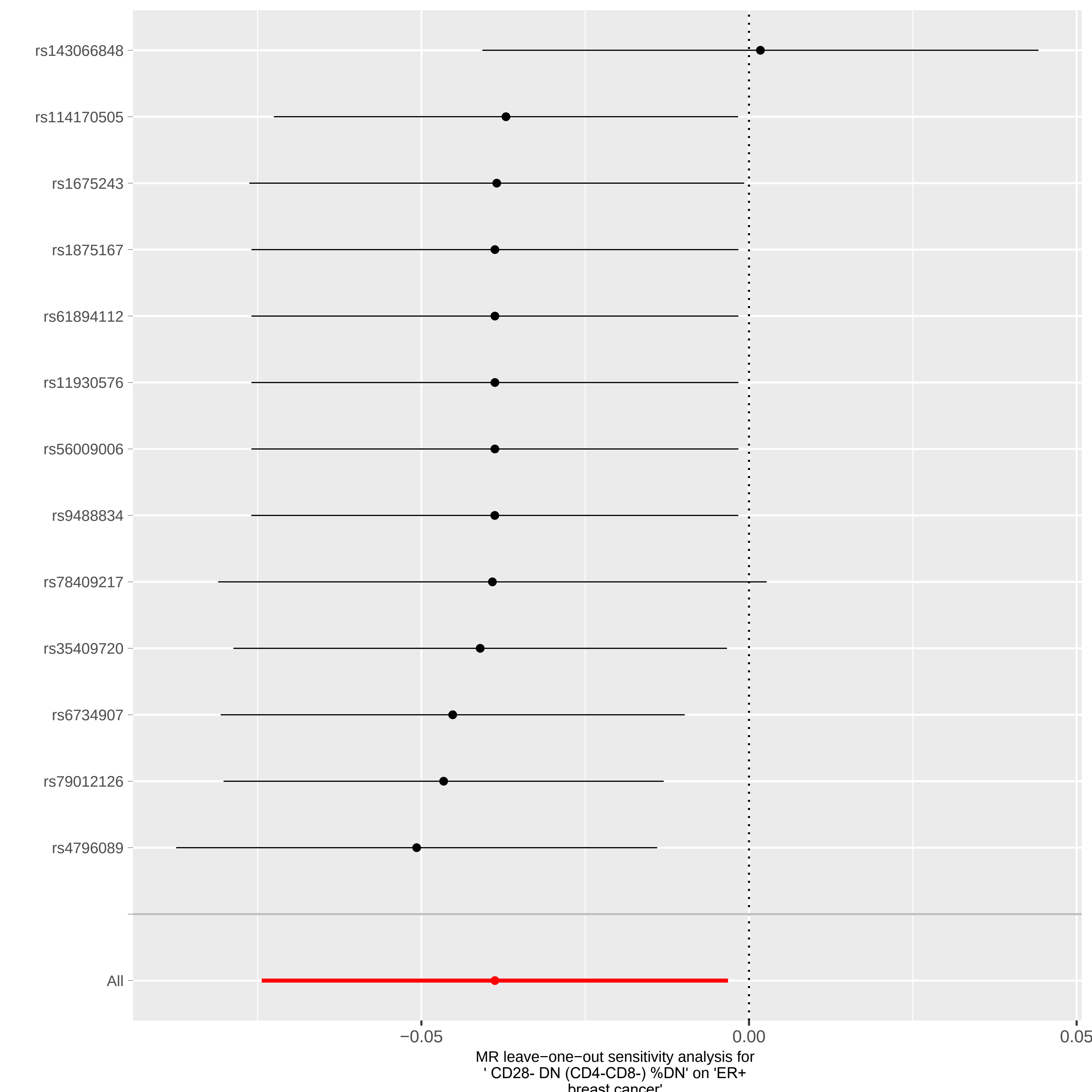
rs143066848
rs114170505
rs1675243
rs1875167
rs61894112
rs11930576
rs56009006
rs9488834
rs78409217
rs35409720
rs6734907
rs79012126
rs4796089
All
−0.05
0.00
0.05
MR leave−one−out sensitivity analysis for
' CD28- DN (CD4-CD8-) %DN' on 'ER+
breast cancer'

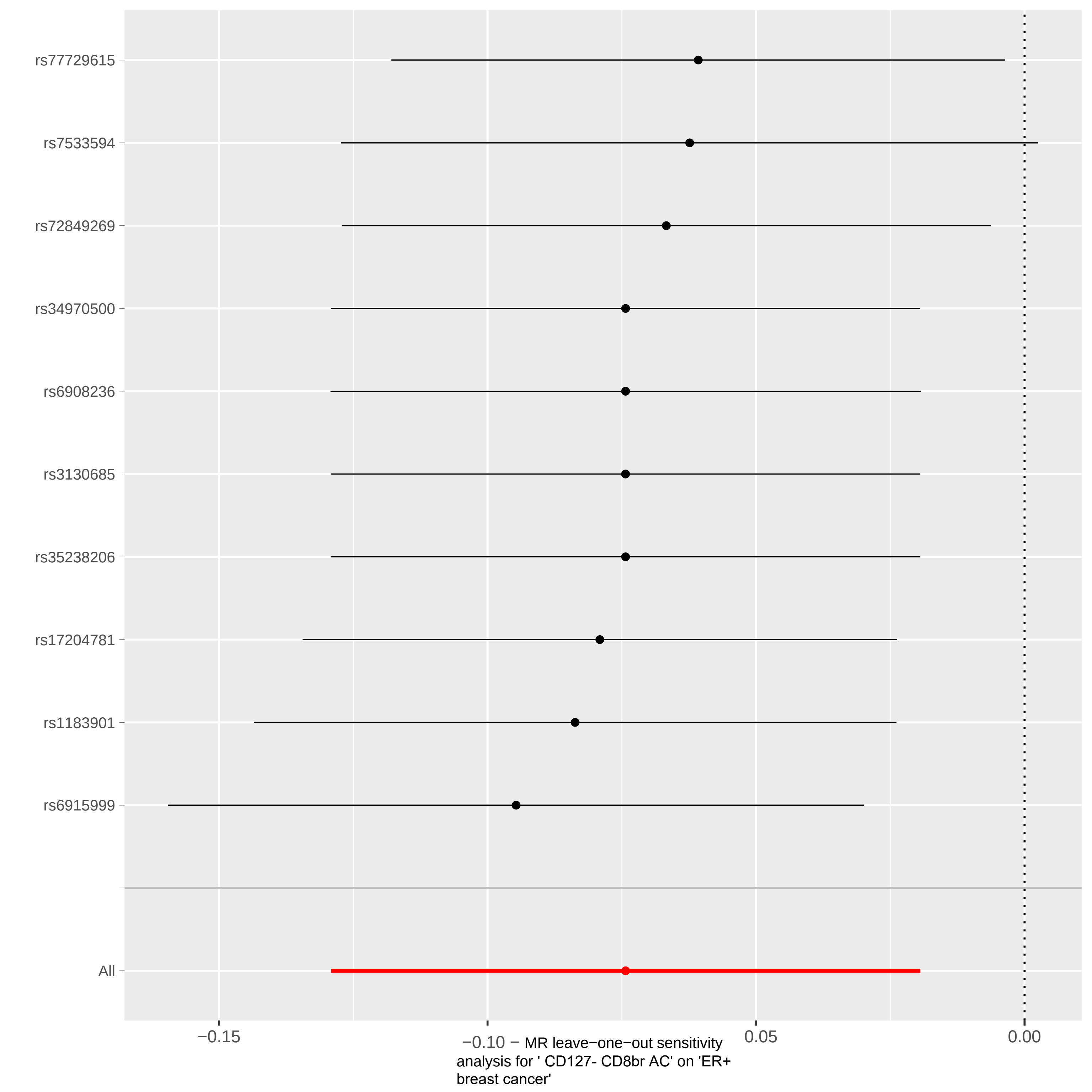
rs77729615
rs7533594
rs72849269
rs34970500
rs6908236
rs3130685
rs35238206
rs17204781
rs1183901
rs6915999
All
−0.15
−0.10
0.05
0.00
MR leave−one−out sensitivity analysis for ' CD127- CD8br AC' on 'ER+ breast cancer'

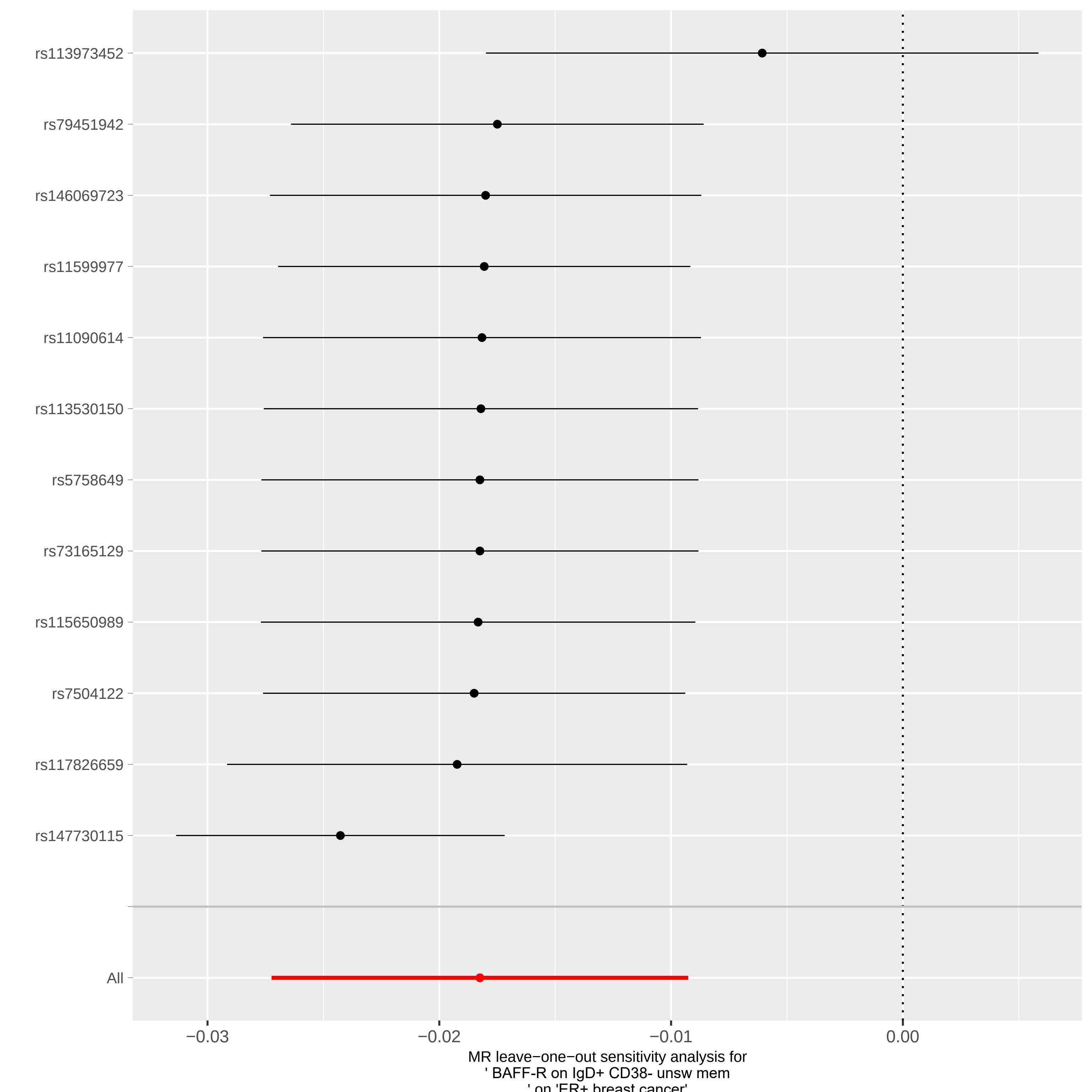
rs113973452
rs79451942
rs146069723
rs11599977
rs11090614
rs113530150
rs5758649
rs73165129
rs115650989
rs7504122
rs117826659
rs147730115
All
−0.03
−0.02
−0.01
0.00
MR leave−one−out sensitivity analysis for
' BAFF-R on IgD+ CD38- unsw mem
' on 'ER+ breast cancer'

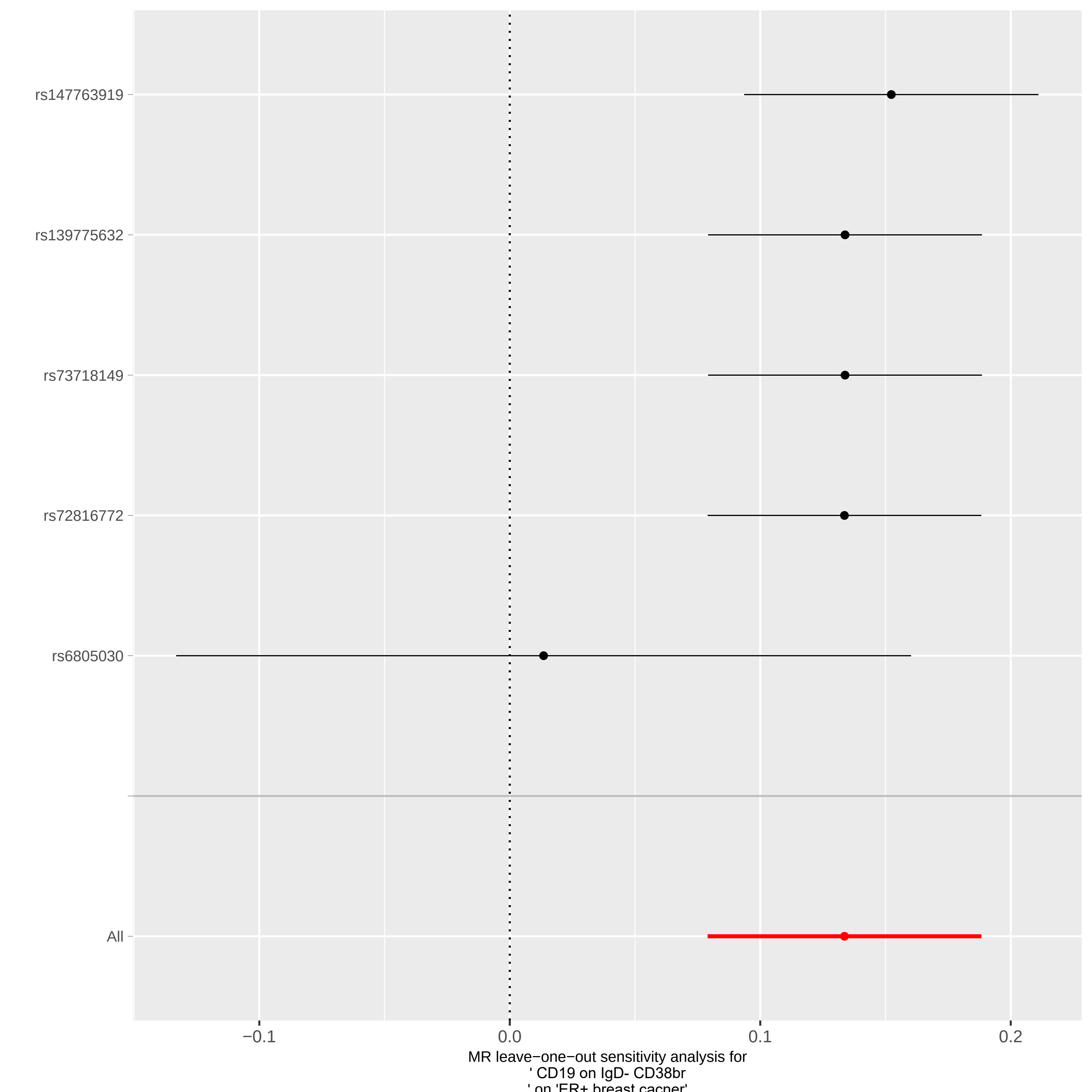
rs147763919
rs139775632
rs73718149
rs72816772
rs6805030
All
−0.1
0.0
0.1
0.2
MR leave−one−out sensitivity analysis for
' CD19 on IgD- CD38br
' on 'ER+ breast cacner'

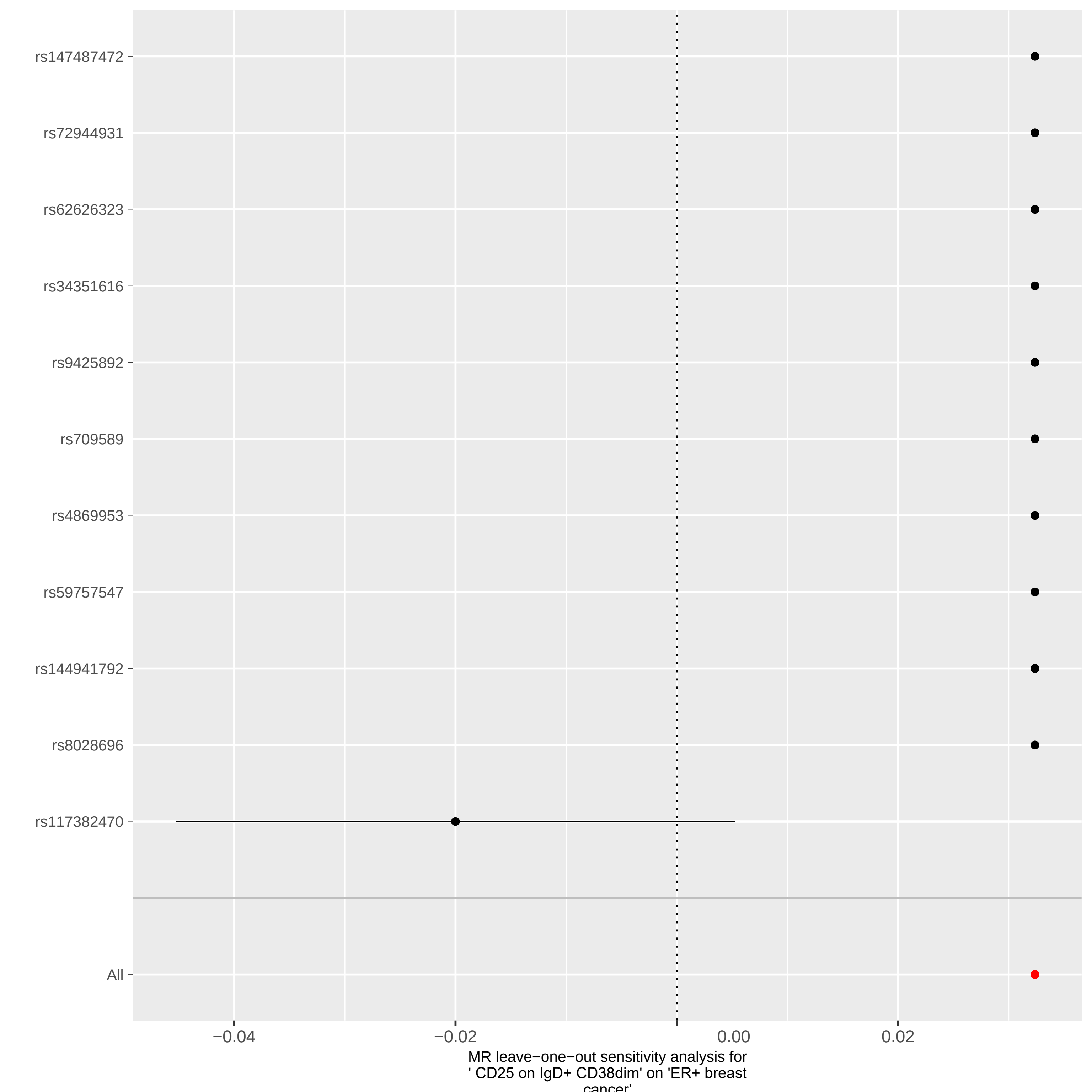

rs147487472
rs72944931
rs62626323
rs34351616
rs9425892
rs709589
rs4869953
rs59757547
rs144941792
rs8028696
rs117382470
All
−0.04
−0.02
0.00
0.02
MR leave−one−out sensitivity analysis for
' CD25 on IgD+ CD38dim' on 'ER+ breast
cancer'

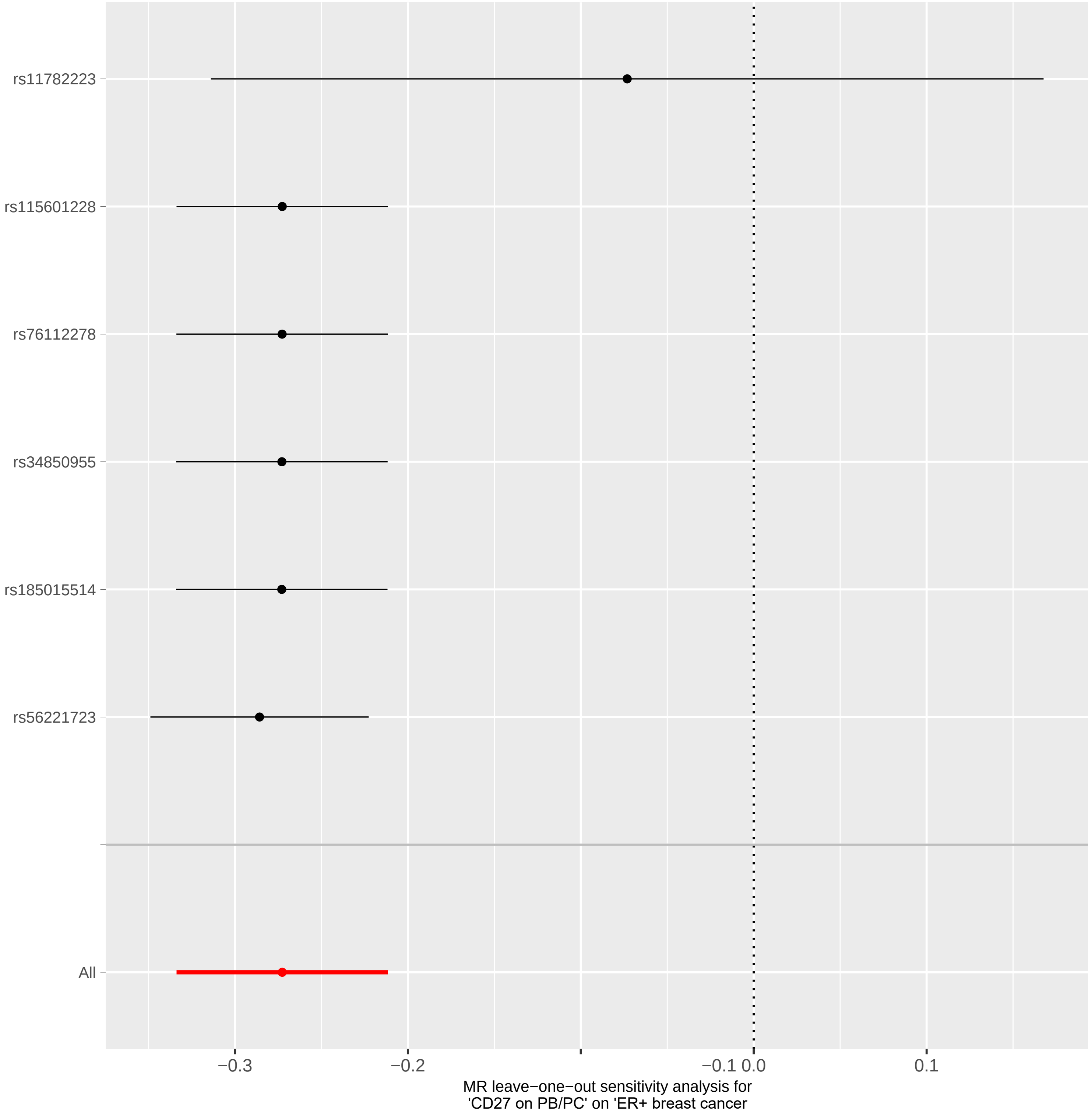

rs11782223
rs115601228
rs76112278
rs34850955
rs185015514
rs56221723
All
−0.3
−0.2
−0.1 0.0
0.1
MR leave−one−out sensitivity analysis for
'CD27 on PB/PC' on 'ER+ breast cancer

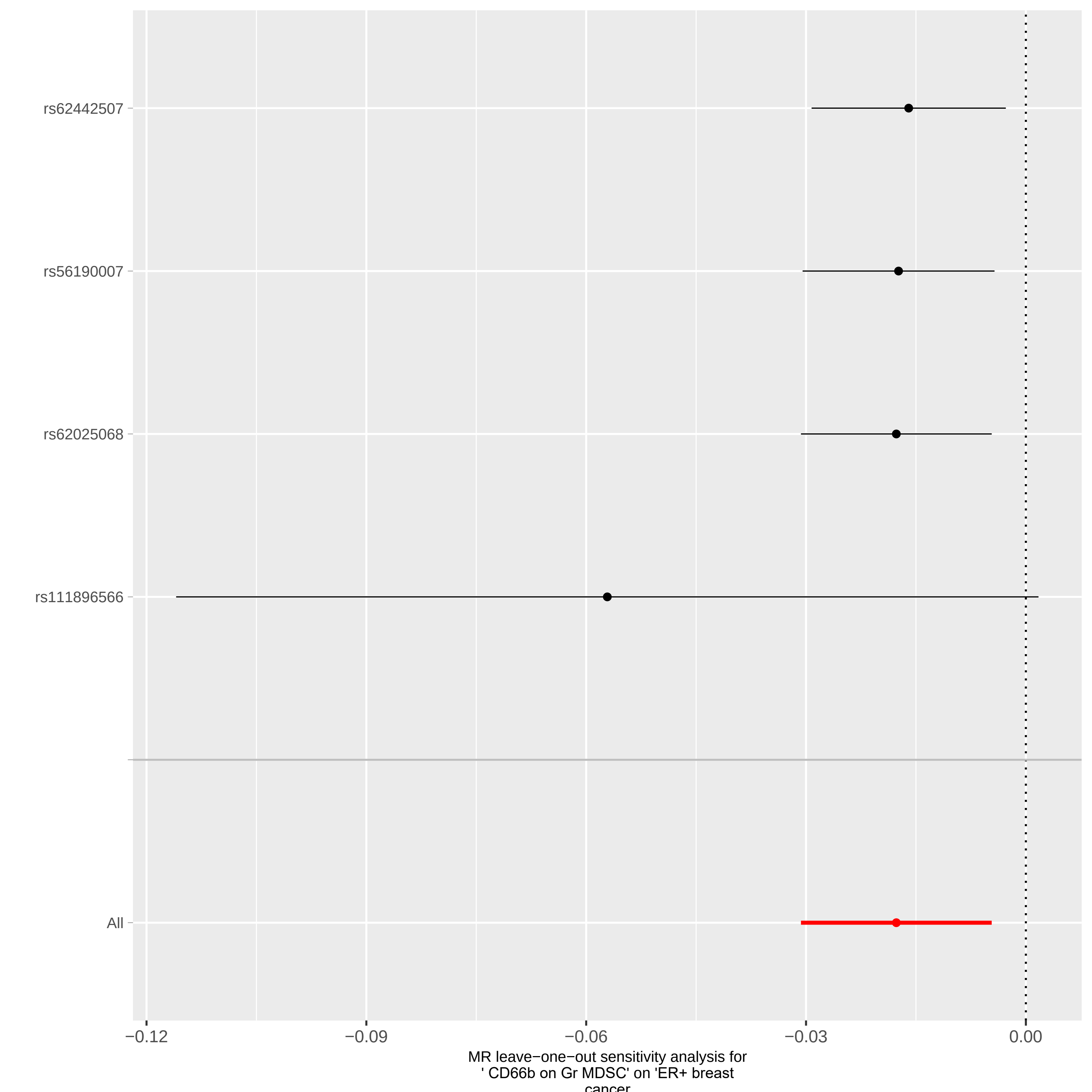
rs62442507
rs56190007
rs62025068
rs111896566
All
−0.12
−0.09
−0.06
−0.03
0.00
MR leave−one−out sensitivity analysis for
' CD66b on Gr MDSC' on 'ER+ breast
cancer

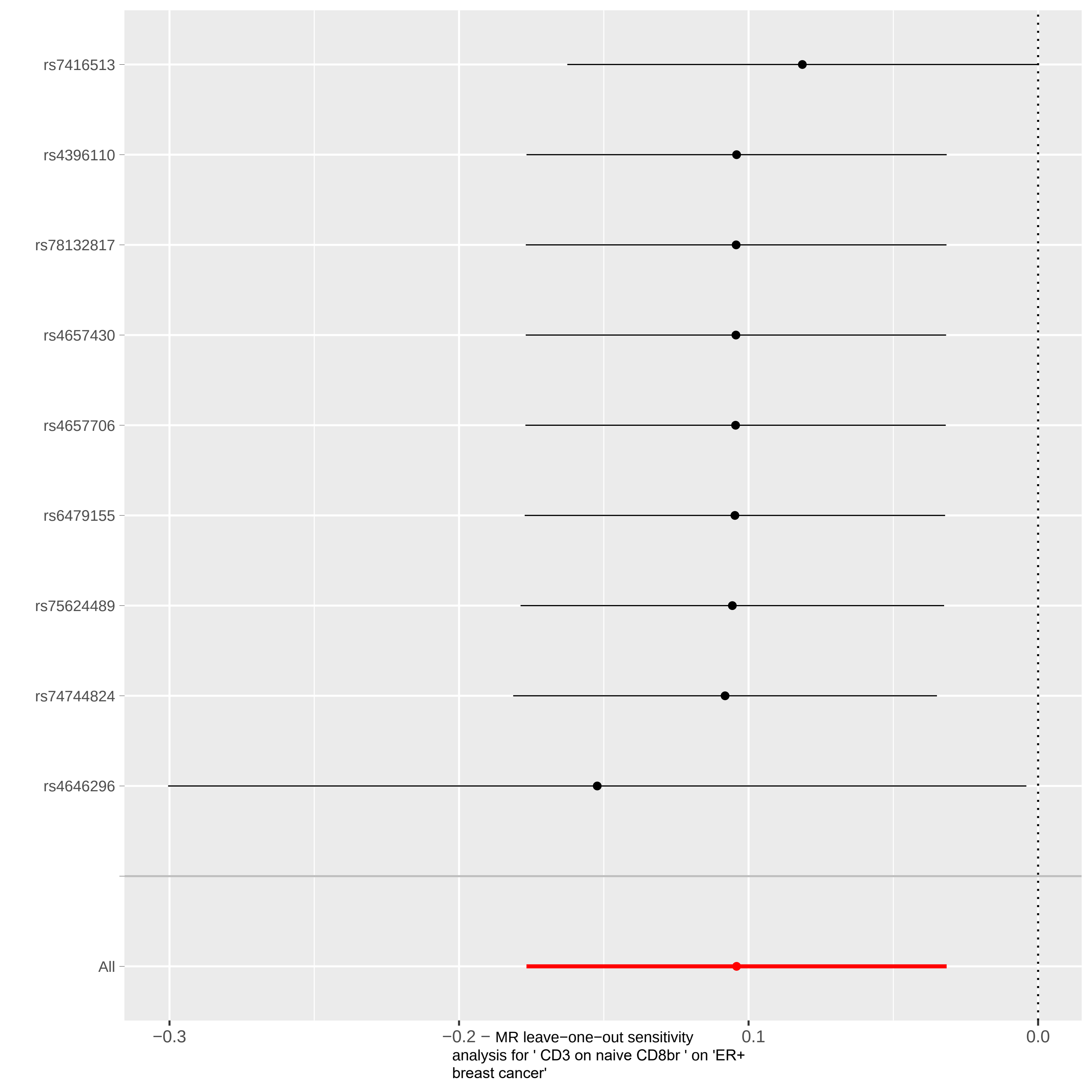

rs7416513
rs4396110
rs78132817
rs4657430
rs4657706
rs6479155
rs75624489
rs74744824
rs4646296
All
−0.3
−0.2
0.1
0.0
MR leave−one−out sensitivity analysis for ' CD3 on naive CD8br ' on 'ER+ breast cancer'

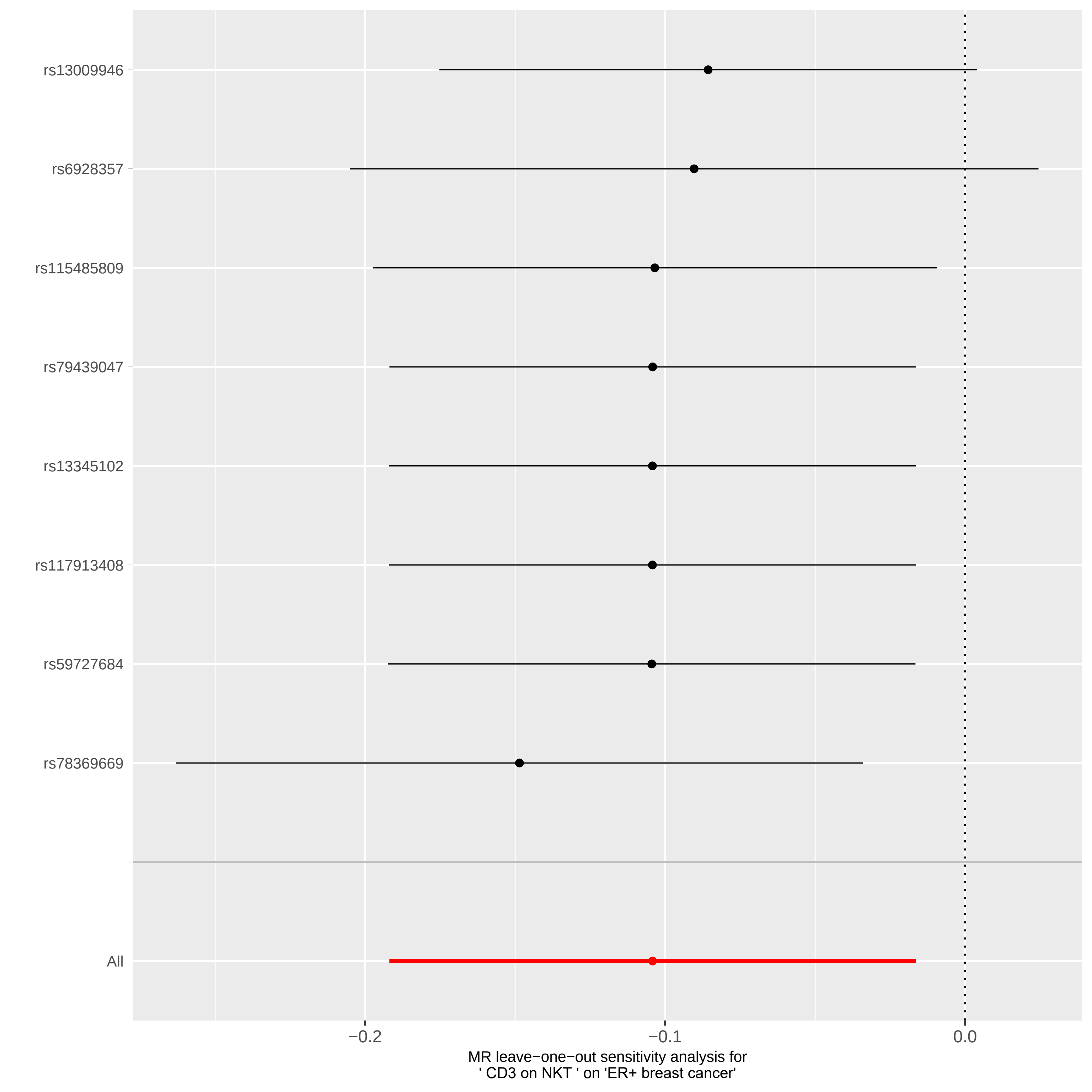

rs13009946
rs6928357
rs115485809
rs79439047
rs13345102
rs117913408
rs59727684
rs78369669
All
−0.2
−0.1
0.0
MR leave−one−out sensitivity analysis for
' CD3 on NKT ' on 'ER+ breast cancer'

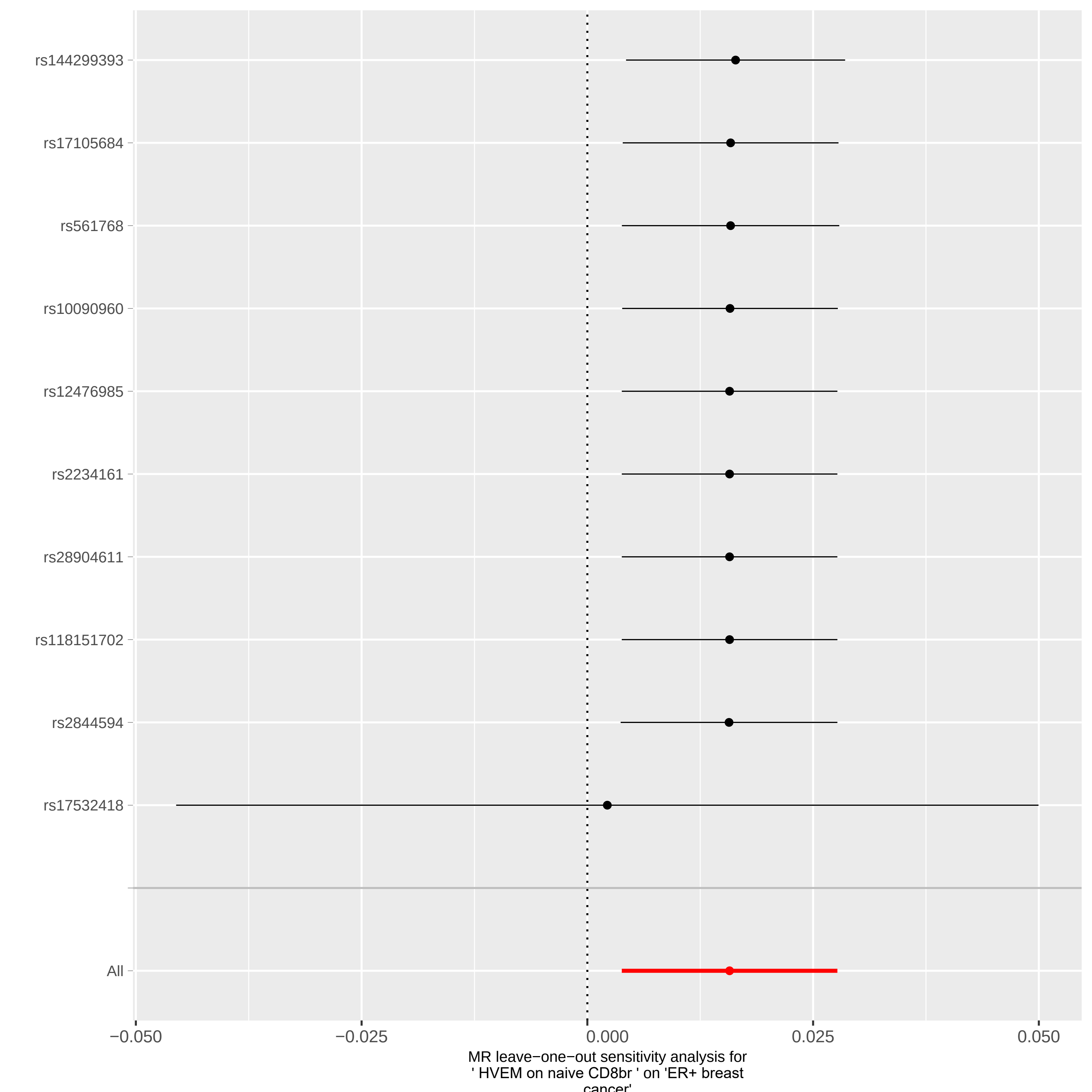
rs144299393
rs17105684
rs561768
rs10090960
rs12476985
rs2234161
rs28904611
rs118151702
rs2844594
rs17532418
All
−0.050
−0.025
0.000
0.025
0.050
MR leave−one−out sensitivity analysis for
' HVEM on naive CD8br ' on 'ER+ breast
cancer'

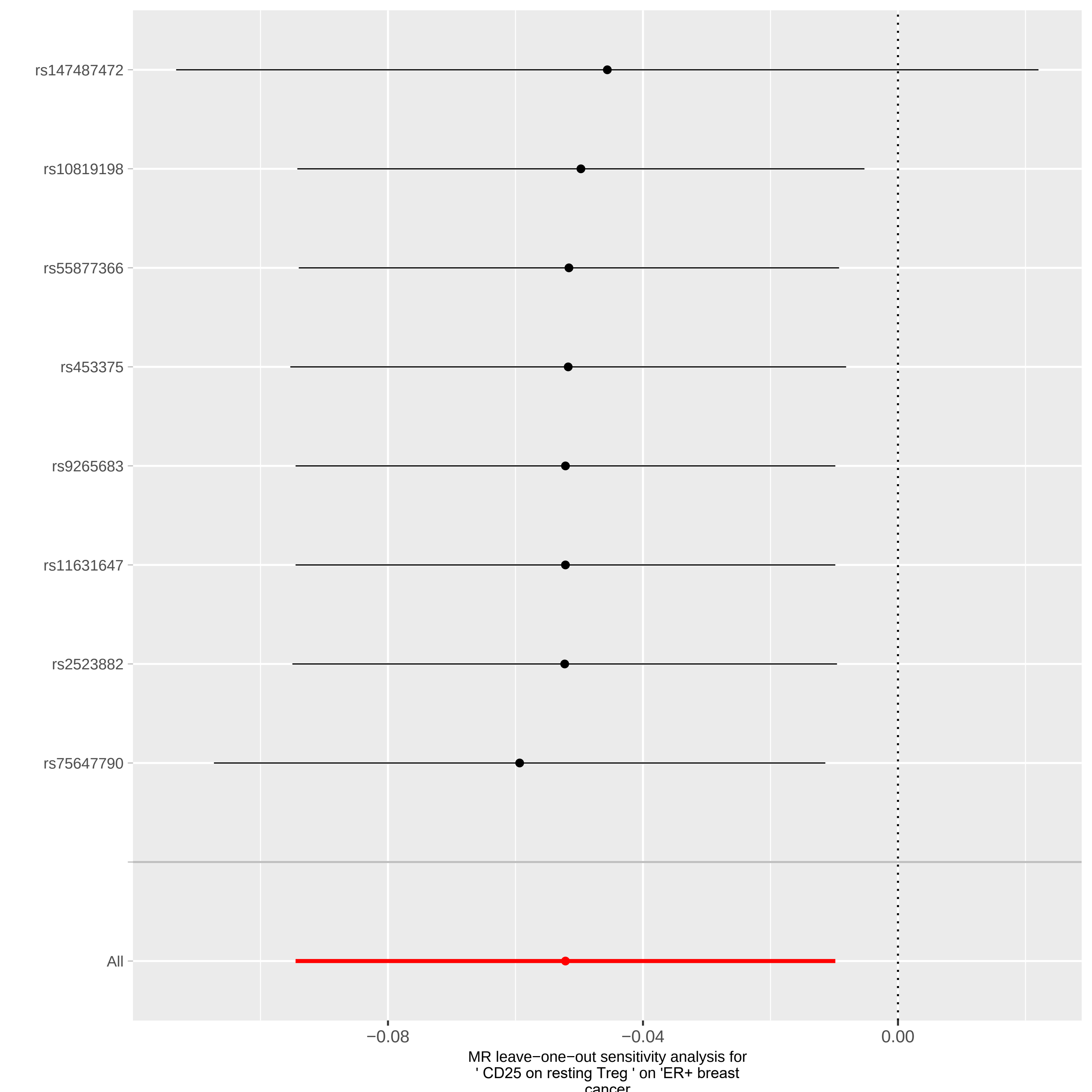
rs147487472
rs10819198
rs55877366
rs453375
rs9265683
rs11631647
rs2523882
rs75647790
All
−0.08
−0.04
0.00
MR leave−one−out sensitivity analysis for
' CD25 on resting Treg ' on 'ER+ breast
cancer

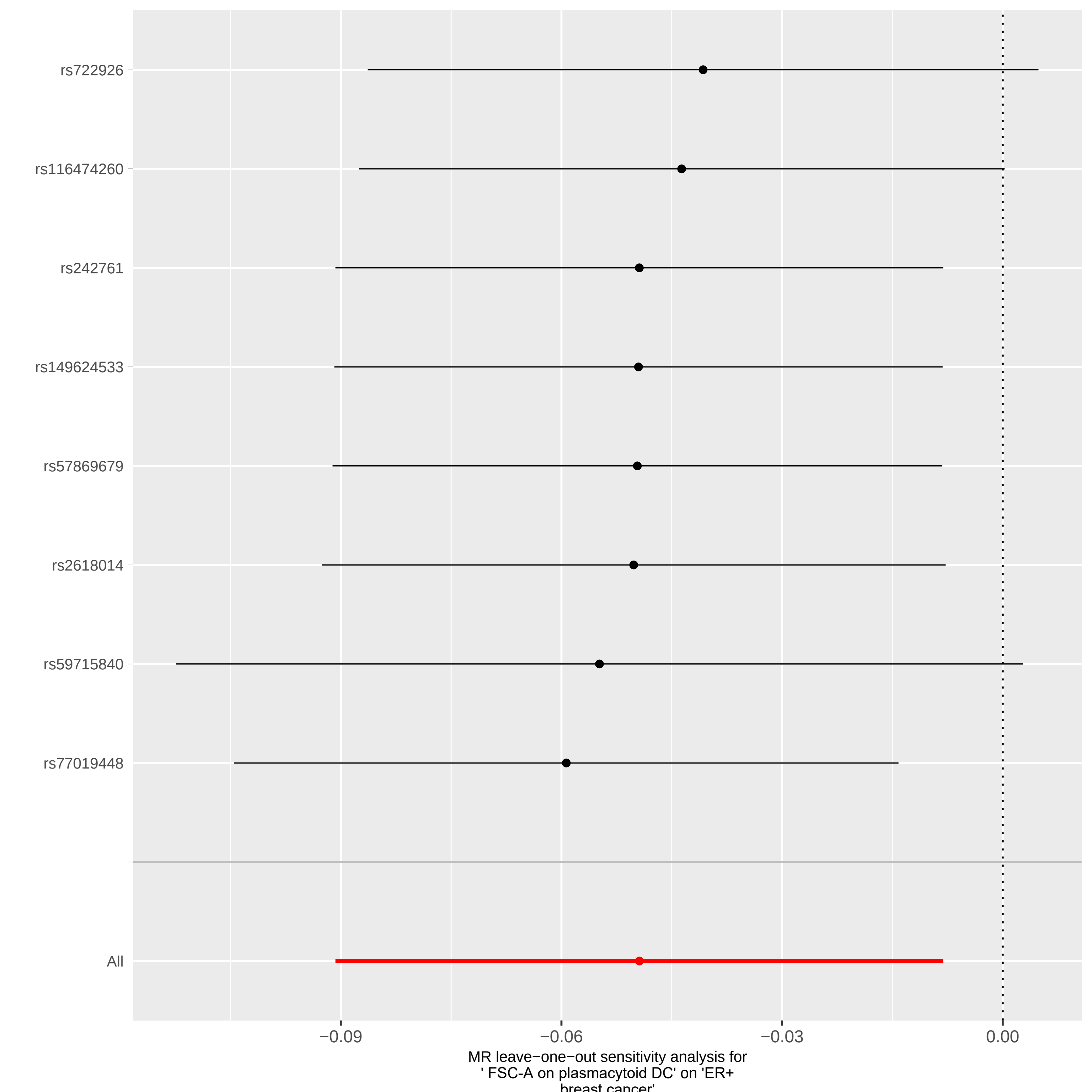

rs722926
rs116474260
rs242761
rs149624533
rs57869679
rs2618014
rs59715840
rs77019448
All
−0.09
−0.06
−0.03
0.00
MR leave−one−out sensitivity analysis for
' FSC-A on plasmacytoid DC' on 'ER+
breast cancer'

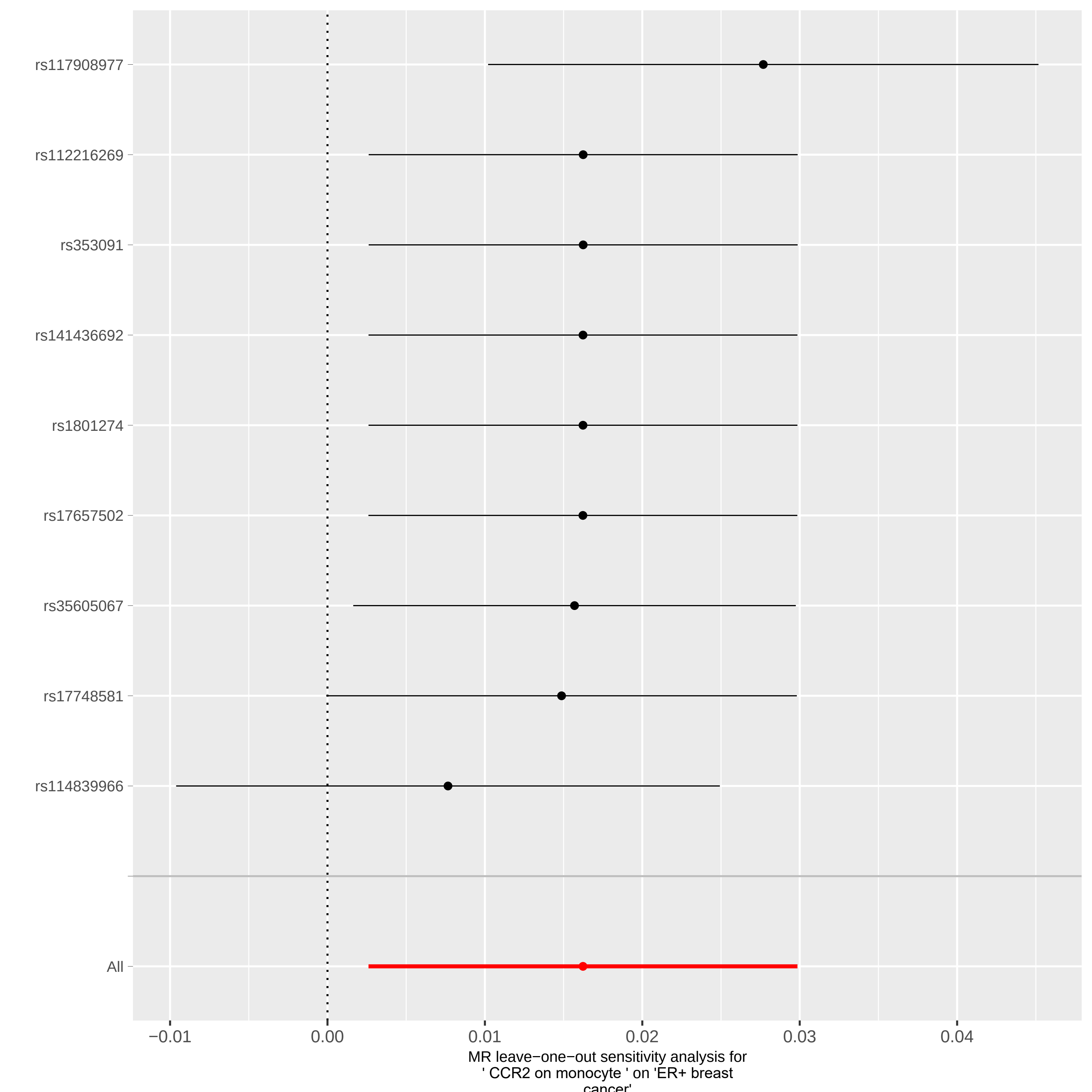
rs117908977
rs112216269
rs353091
rs141436692
rs1801274
rs17657502
rs35605067
rs17748581
rs114839966
All
−0.01
0.00
0.01
0.02
0.03
0.04
MR leave−one−out sensitivity analysis for
' CCR2 on monocyte ' on 'ER+ breast
cancer'

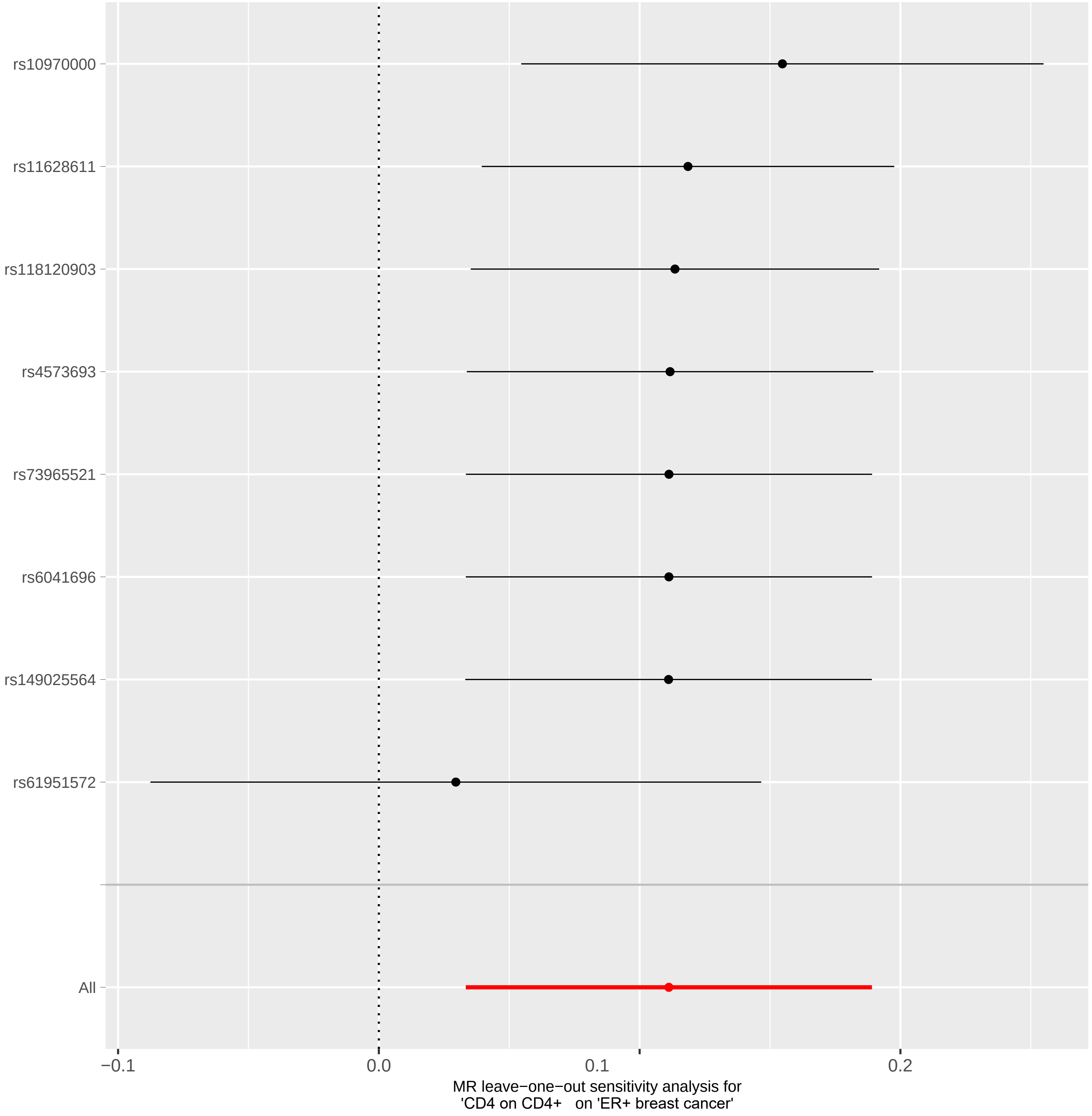
rs10970000
rs11628611
rs118120903
rs4573693
rs73965521
rs6041696
rs149025564
rs61951572
All
−0.1
0.0
0.1
0.2
MR leave−one−out sensitivity analysis for
'CD4 on CD4+ on 'ER+ breast cancer'

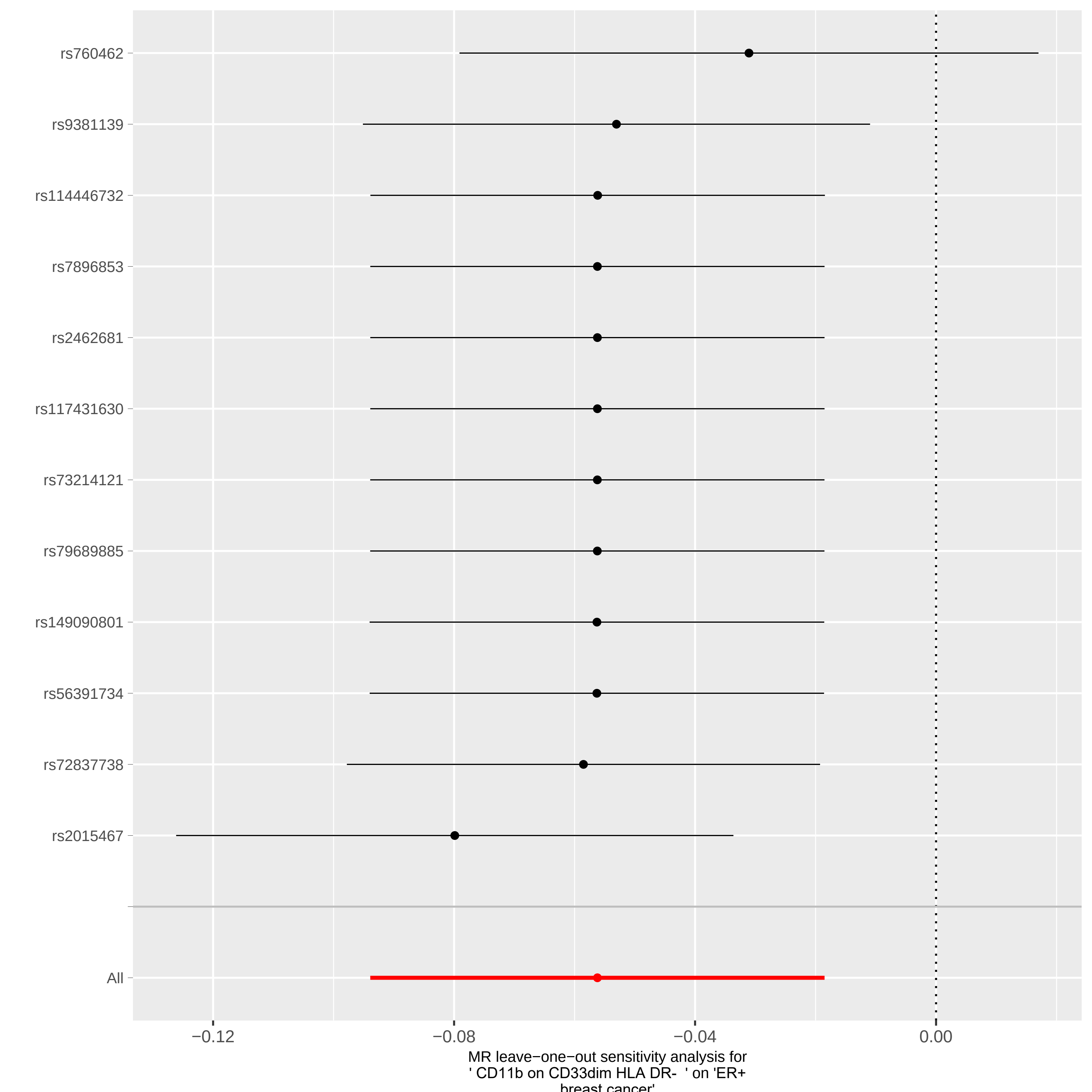
rs760462
rs9381139
rs114446732
rs7896853
rs2462681
rs117431630
rs73214121
rs79689885
rs149090801
rs56391734
rs72837738
rs2015467
All
−0.12
−0.08
−0.04
0.00
MR leave−one−out sensitivity analysis for
' CD11b on CD33dim HLA DR- ' on 'ER+
breast cancer'

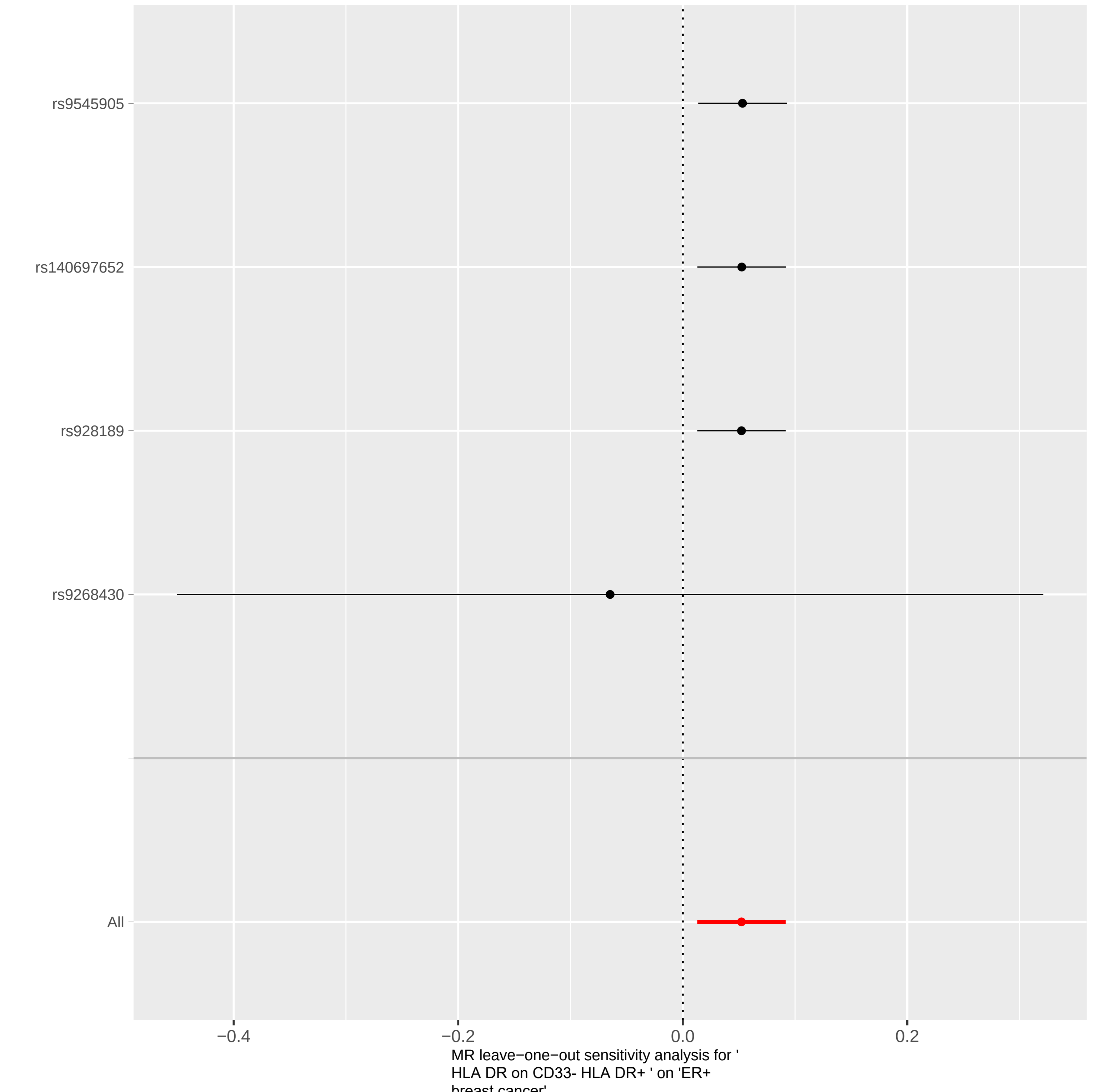

rs9545905
rs140697652
rs928189
rs9268430
All
−0.4
−0.2
0.0
0.2
MR leave−one−out sensitivity analysis for '
HLA DR on CD33- HLA DR+ ' on 'ER+
breast cancer'

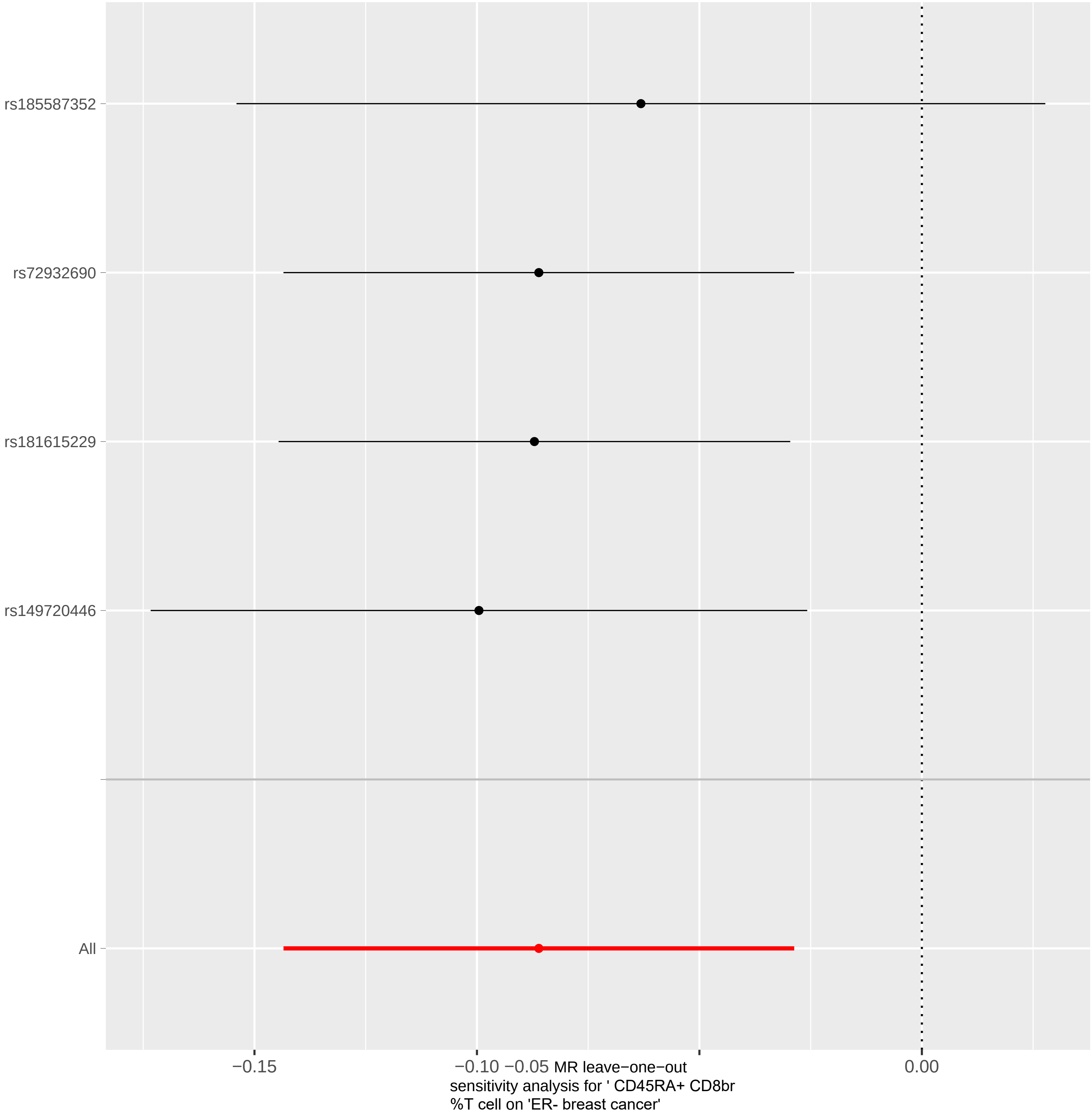

rs185587352
rs72932690
rs181615229
rs149720446
All
−0.15
−0.10
−0.05
0.00
MR leave−one−out
sensitivity analysis for ' CD45RA+ CD8br
%T cell on 'ER- breast cancer'

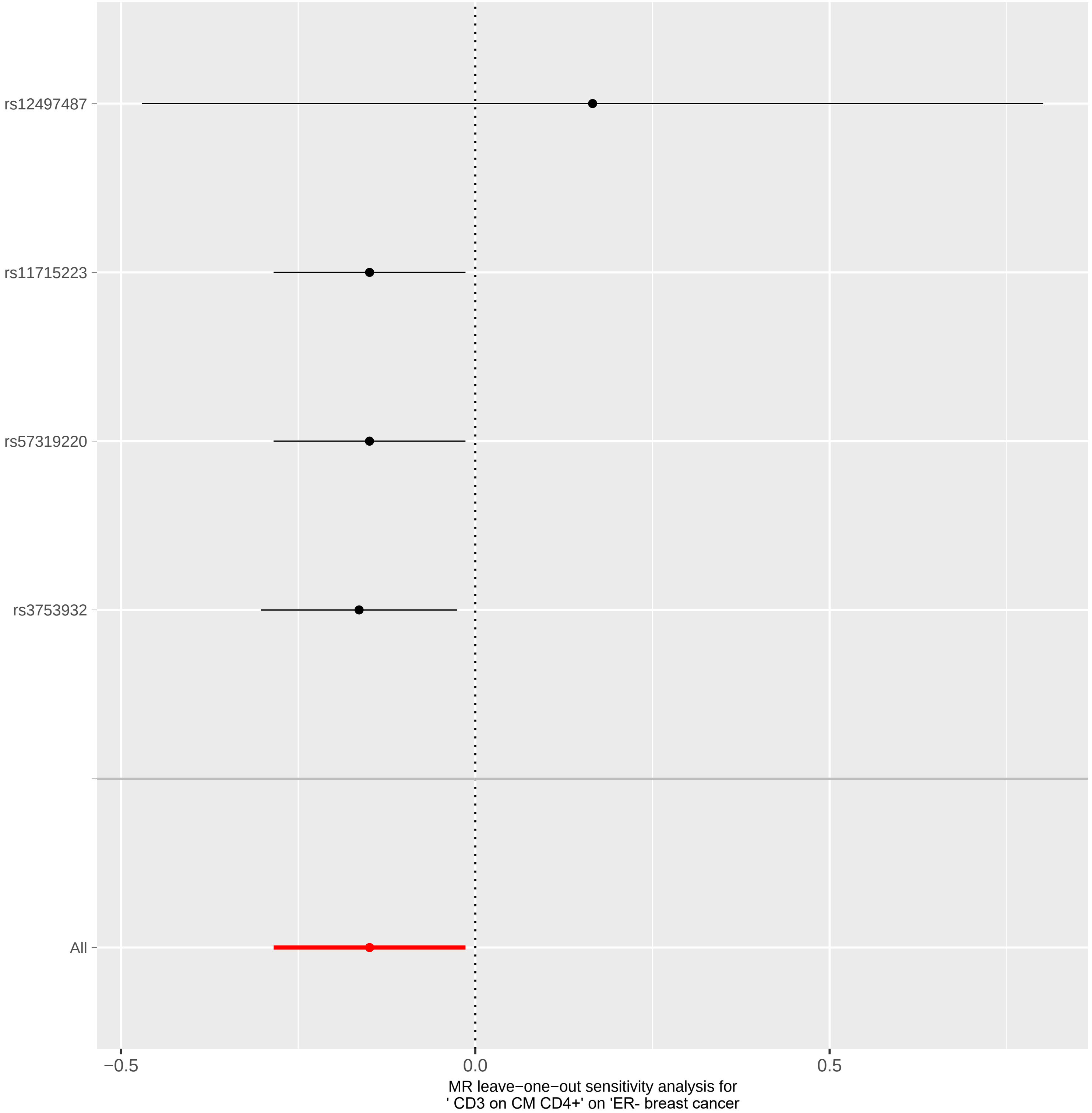
rs12497487
rs11715223
rs57319220
rs3753932
All
−0.5
0.0
0.5
MR leave−one−out sensitivity analysis for
' CD3 on CM CD4+' on 'ER- breast cancer

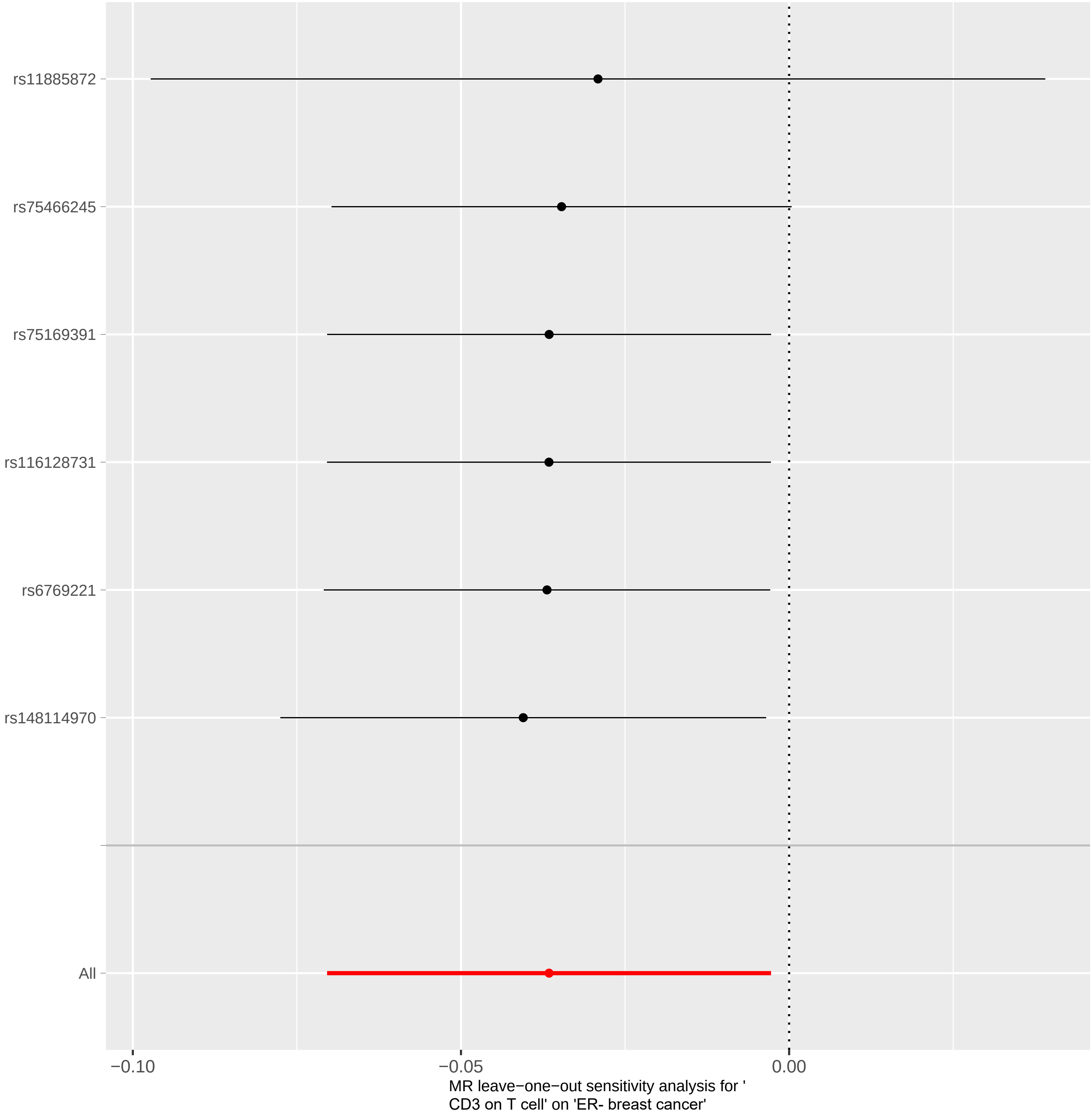
rs11885872
rs75466245
rs75169391
rs116128731
rs6769221
rs148114970
All
−0.10
−0.05
0.00
MR leave−one−out sensitivity analysis for '
CD3 on T cell' on 'ER- breast cancer'

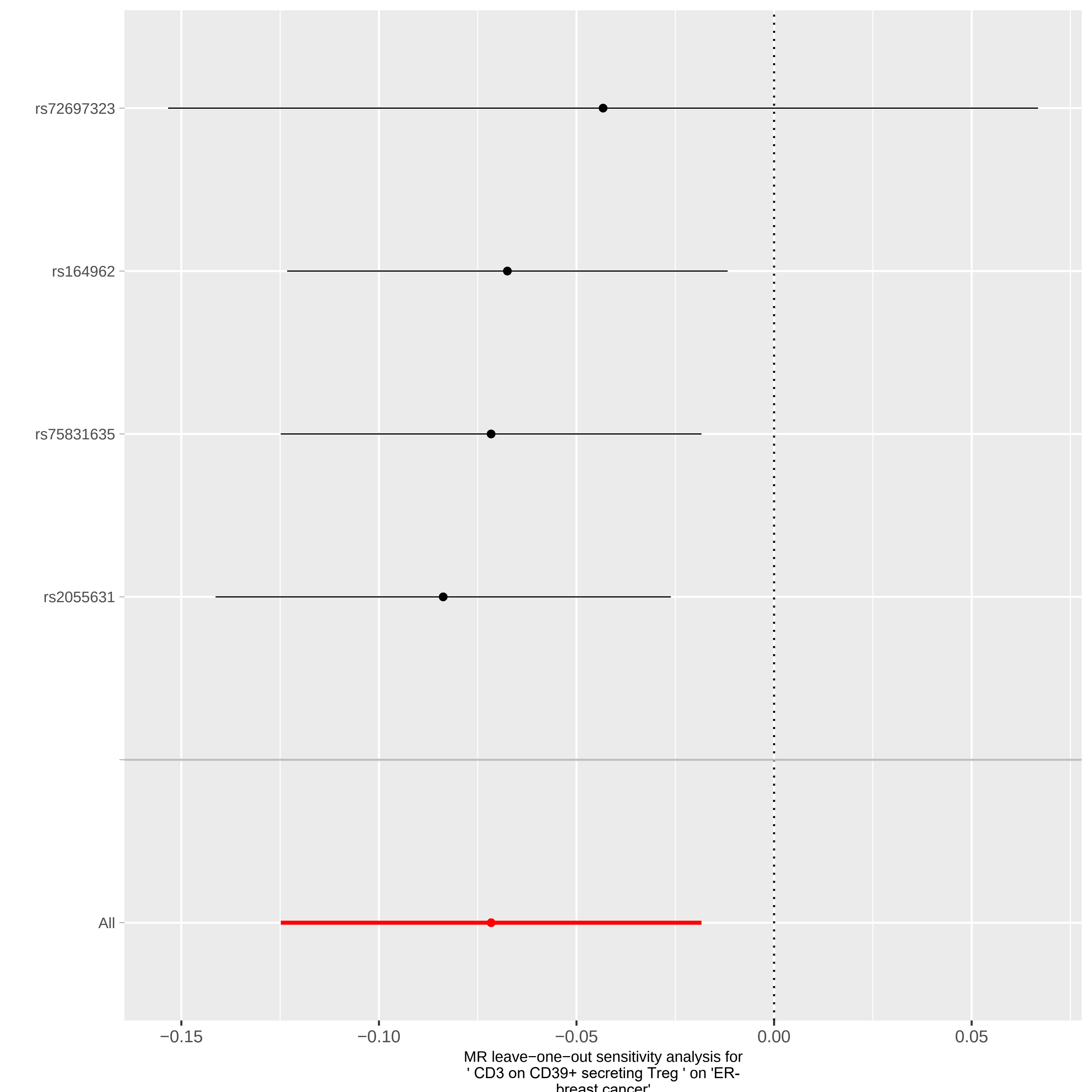
rs72697323
rs164962
rs75831635
rs2055631
All
−0.15
−0.10
−0.05
0.00
0.05
MR leave−one−out sensitivity analysis for
' CD3 on CD39+ secreting Treg ' on 'ER-
breast cancer'

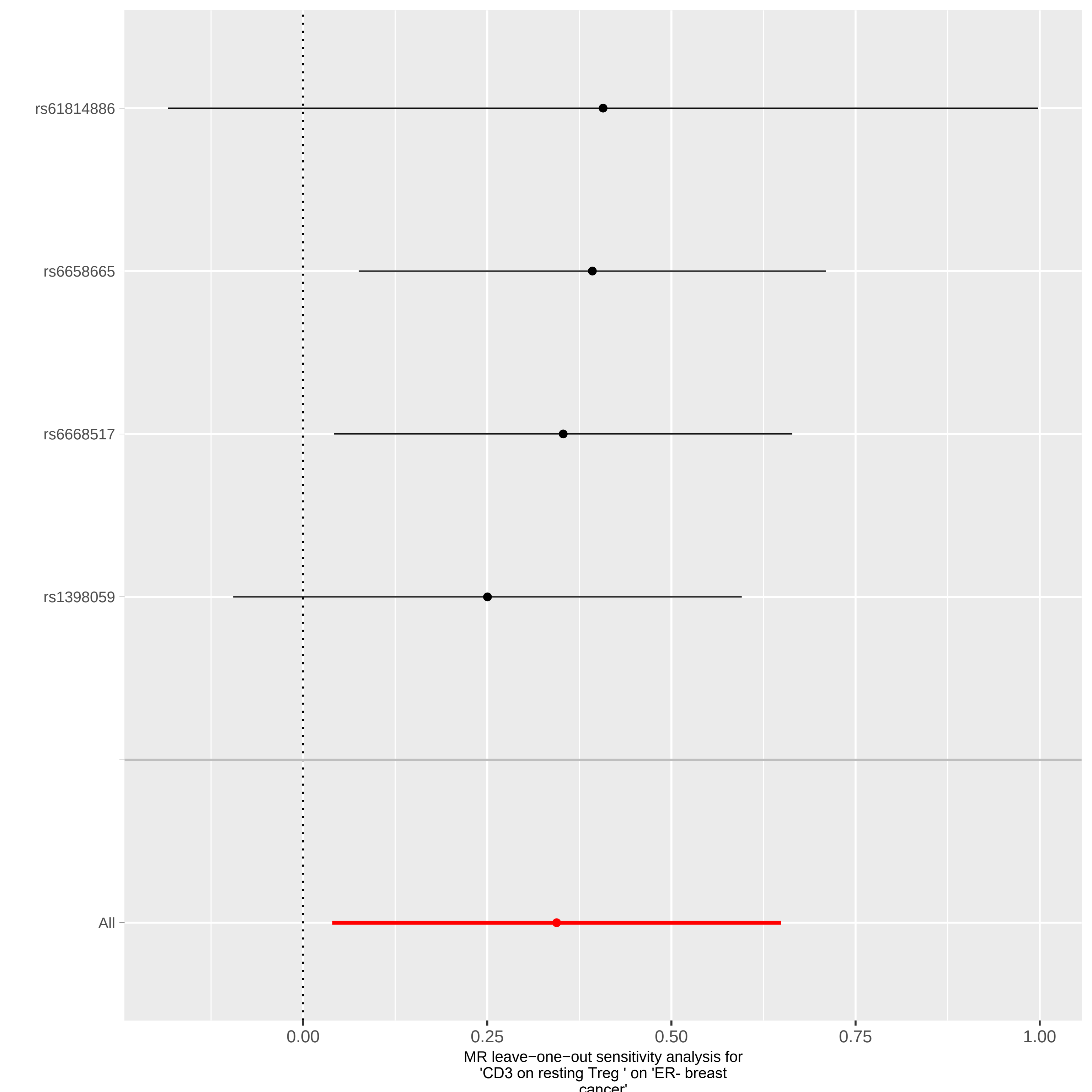
rs61814886
rs6658665
rs6668517
rs1398059
All
0.00
0.25
0.50
0.75
1.00
MR leave−one−out sensitivity analysis for
'CD3 on resting Treg ' on 'ER- breast
cancer'

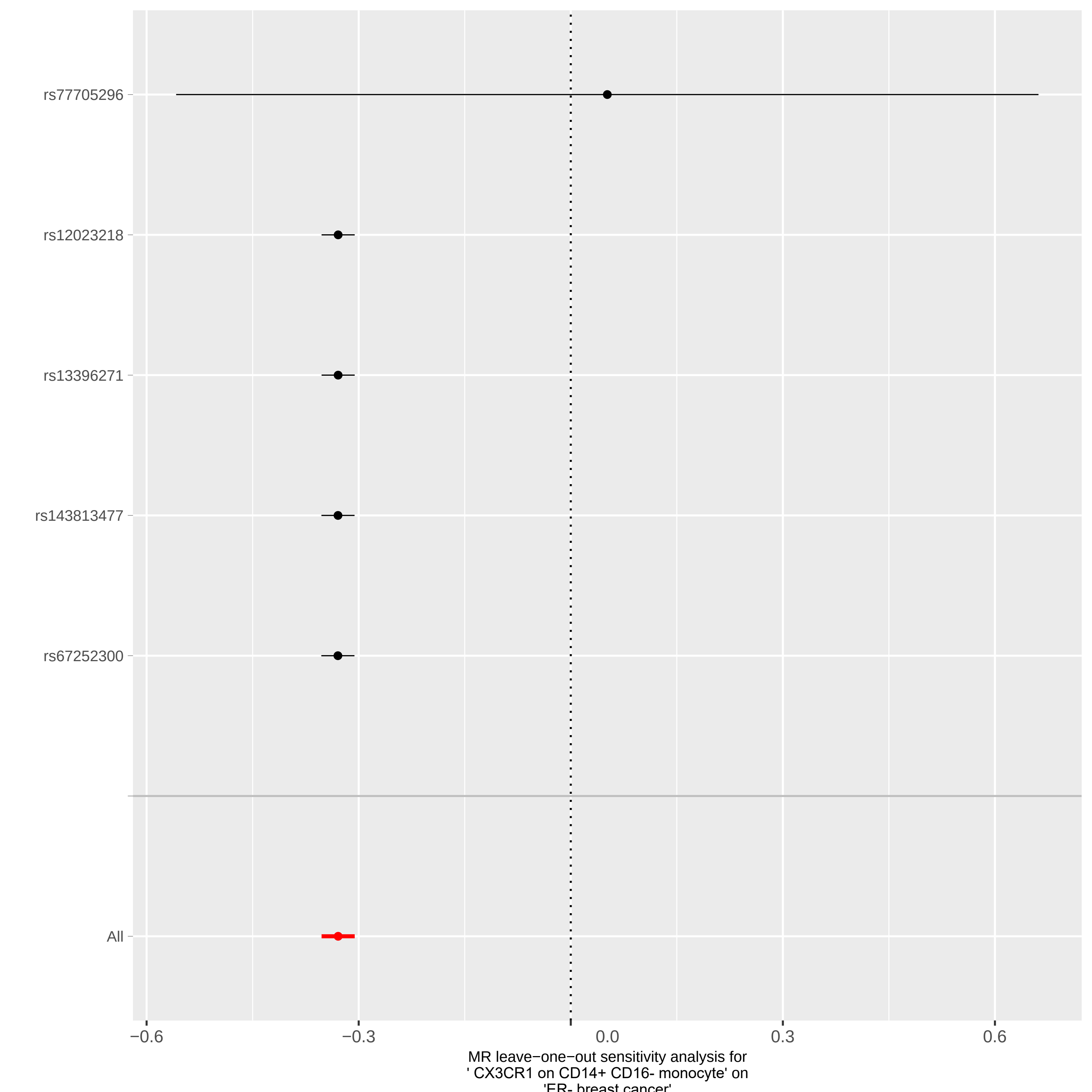
rs77705296
rs12023218
rs13396271
rs143813477
rs67252300
All
−0.6
−0.3
0.0
0.3
0.6
MR leave−one−out sensitivity analysis for
' CX3CR1 on CD14+ CD16- monocyte' on
'ER- breast cancer'

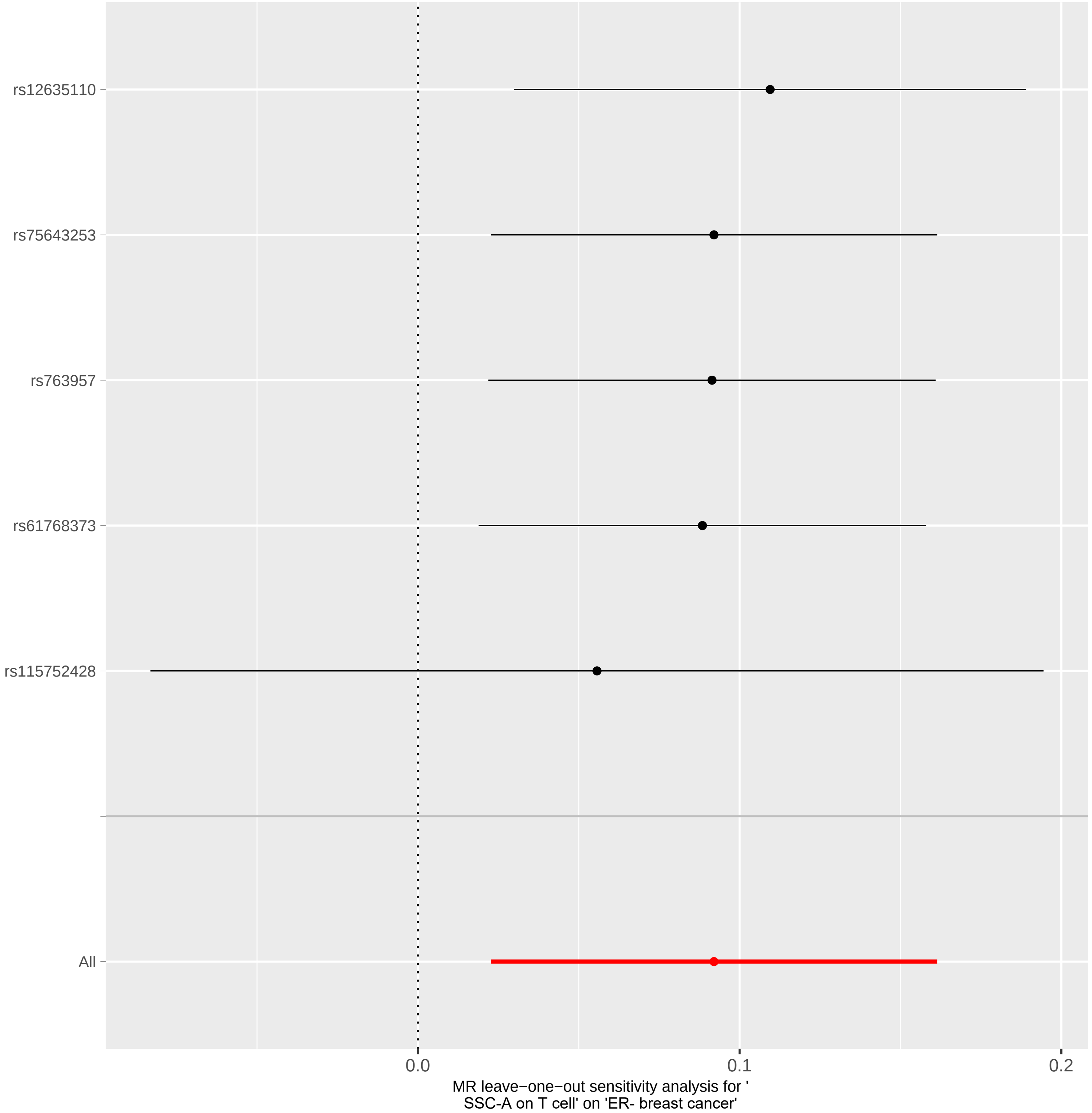
rs12635110
rs75643253
rs763957
rs61768373
rs115752428
All
0.0
0.1
0.2
MR leave−one−out sensitivity analysis for '
SSC-A on T cell' on 'ER- breast cancer'
