# Supplementary material for: A Mendelian analysis of the relationships between immune cells and breast cancer
Source: Front Oncol. 2024 Jan 24;14:1341292. doi: 10.3389/fonc.2024.1341292 (PMC10847340; doi:10.3389/fonc.2024.1341292)
Supplement: Supplementary Figure 2 — The scatter plots of the associations between immune cells on overall breast cancer, ER+ breast cancer and ER- breast cancer. [file DataSheet_2.pdf]

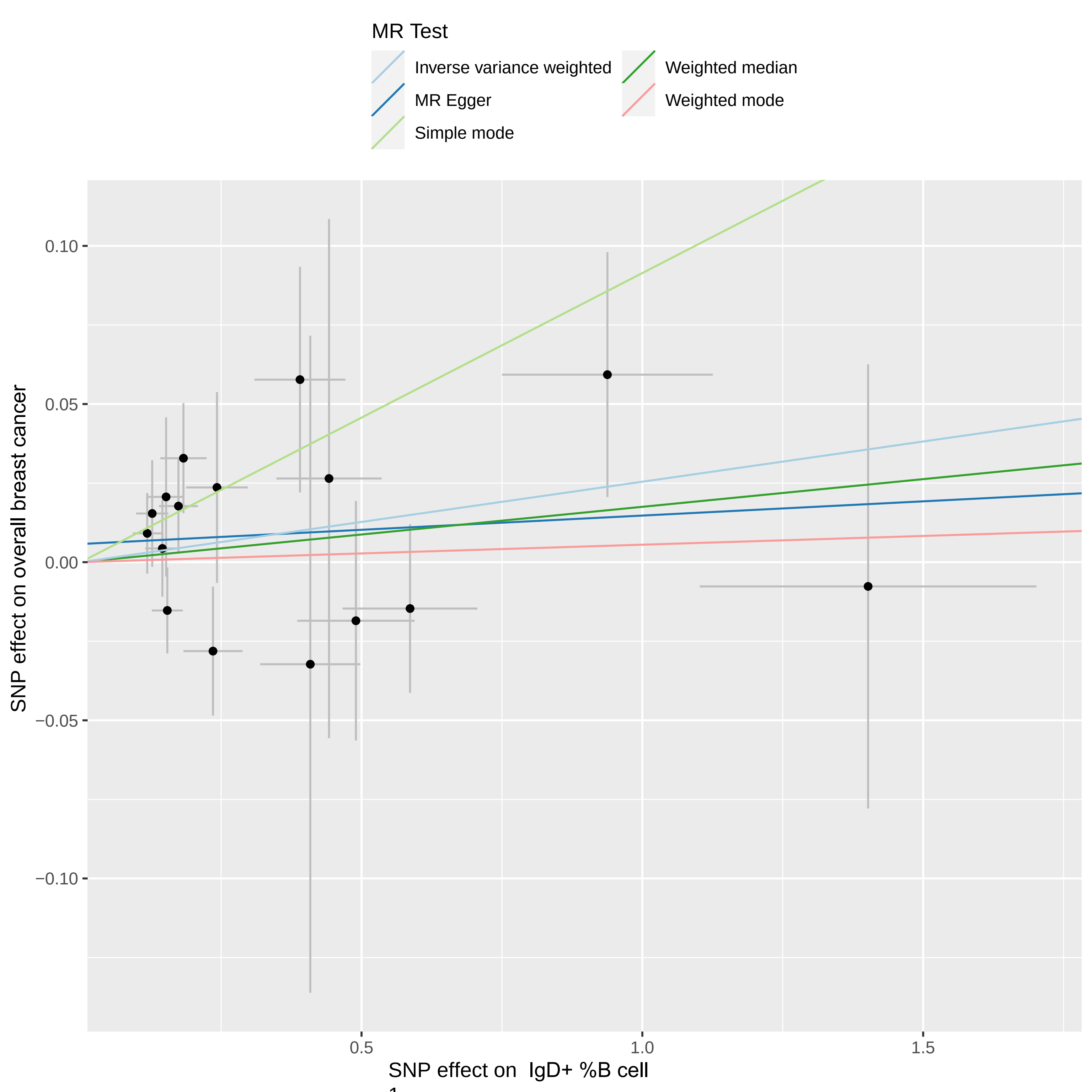
MR Test
Inverse variance weighted
MR Egger
Simple mode
Weighted median
Weighted mode
0.10
0.05
0.00
−0.05
−0.10
0.5
1.0
1.5
SNP effect on overall breast cancer
SNP effect on IgD+ %B cell

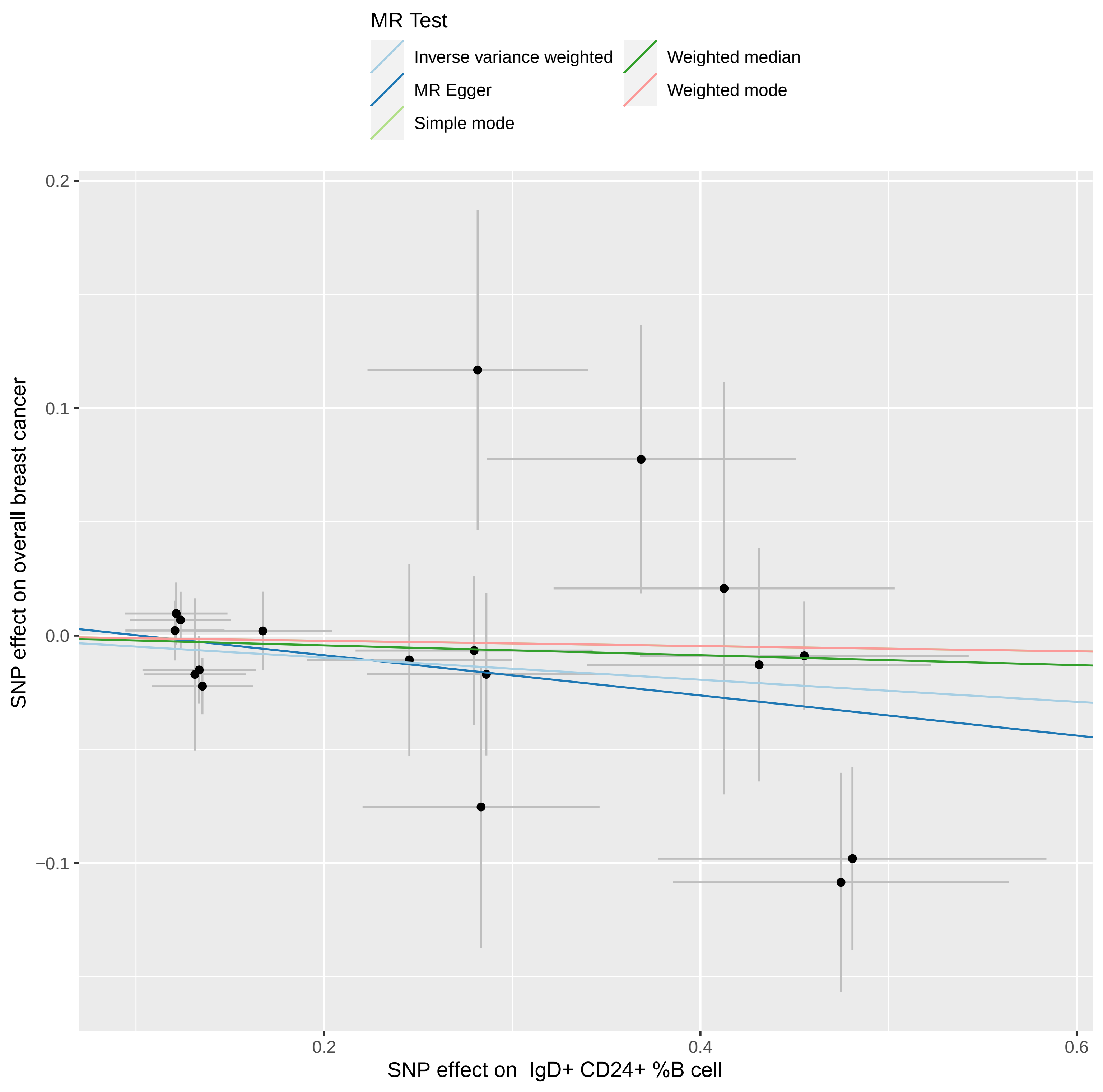

MR Test
Inverse variance weighted
MR Egger
Simple mode
Weighted median
Weighted mode
0.2
0.1
0.0
−0.1
SNP effect on overall breast cancer
0.2
0.4
0.6
SNP effect on IgD+ CD24+ %B cell

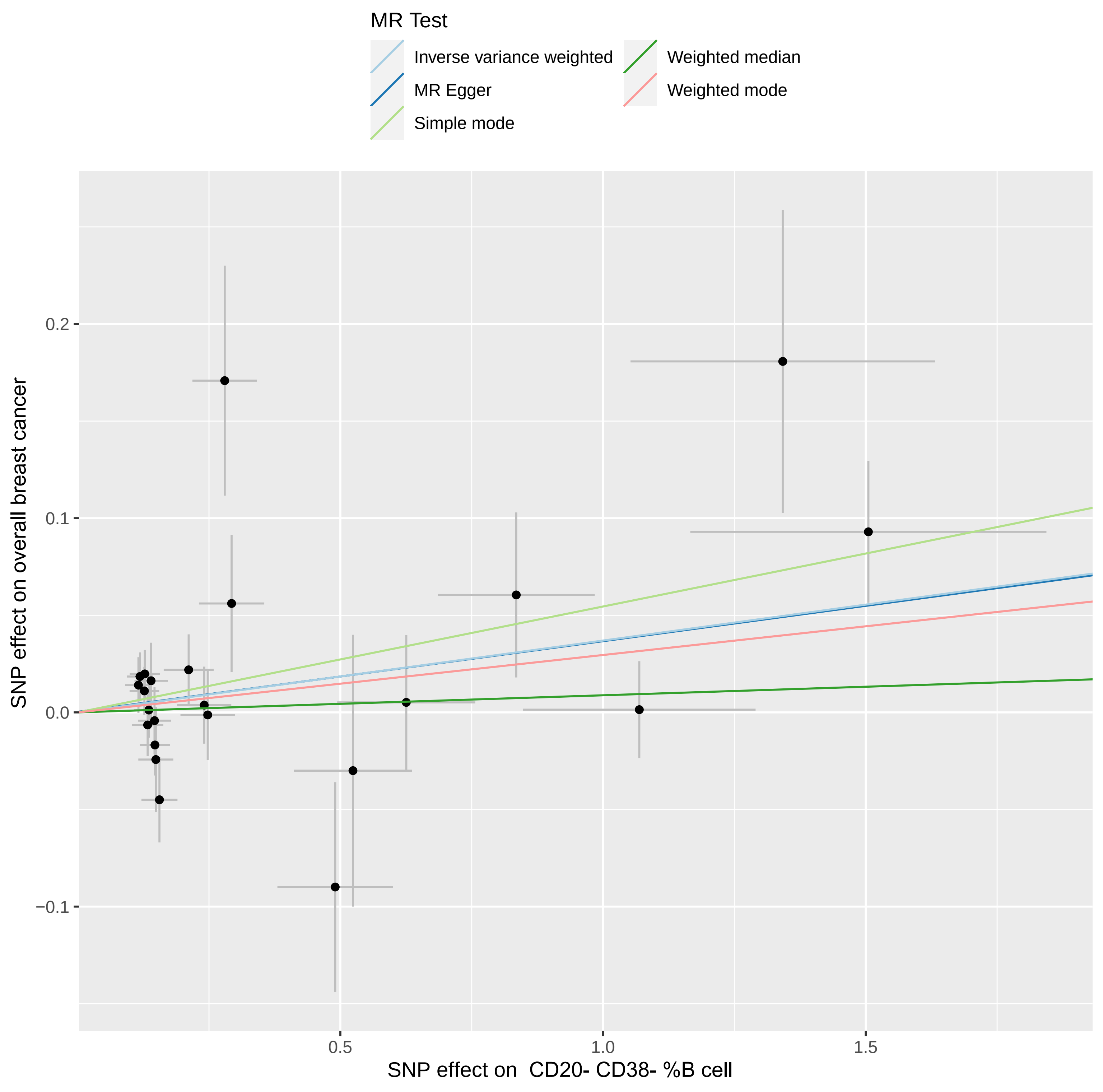

MR Test
Inverse variance weighted
MR Egger
Simple mode
Weighted median
Weighted mode
0.2
0.1
0.0
−0.1
0.5
1.0
1.5
SNP effect on overall breast cancer
SNP effect on CD20- CD38- %B cell

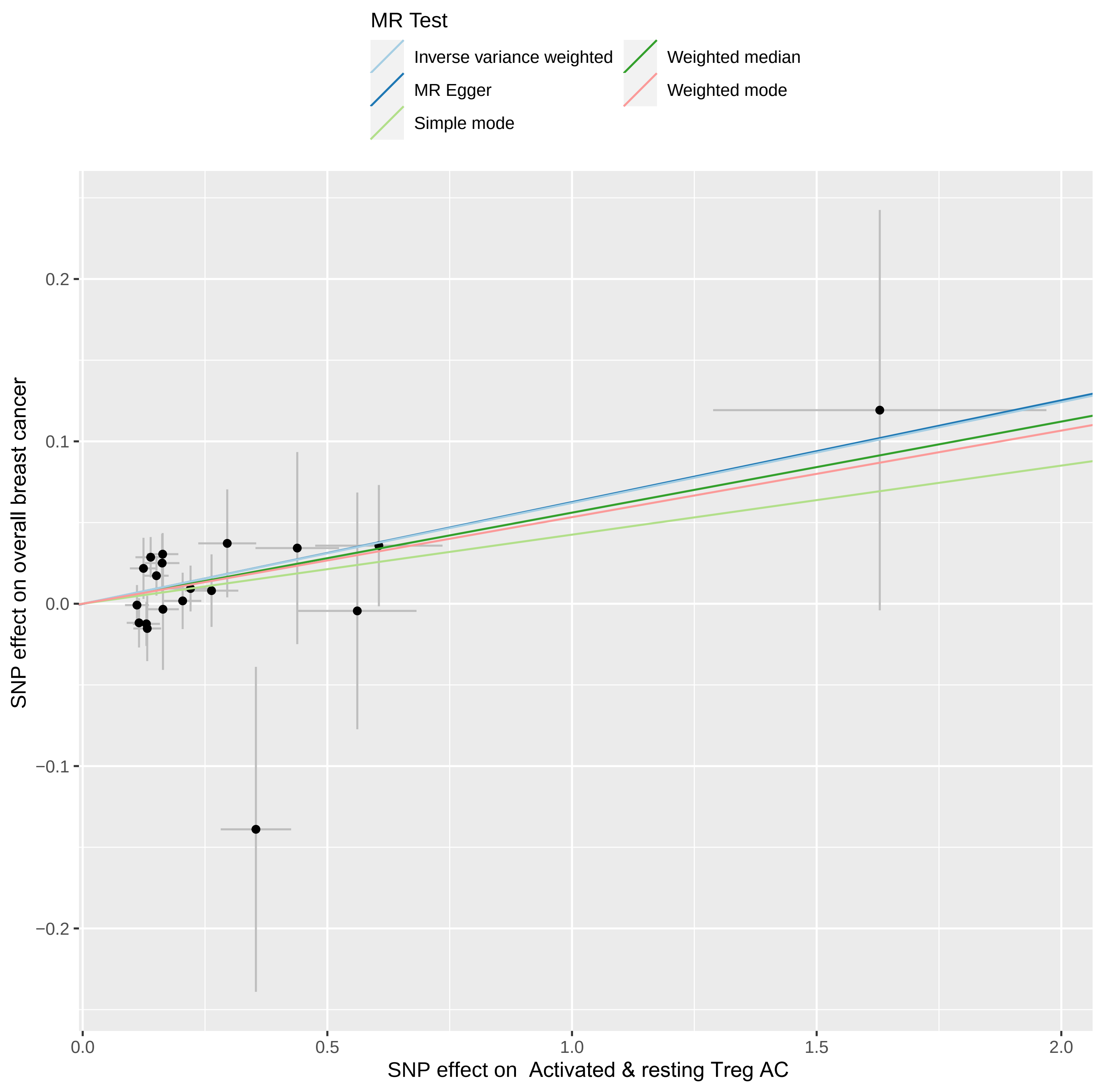
MR Test
Inverse variance weighted
MR Egger
Simple mode
Weighted median
Weighted mode
0.2
0.1
0.0
−0.1
−0.2
SNP effect on overall breast cancer
0.0
0.5
1.0
1.5
2.0
SNP effect on Activated & resting Treg AC

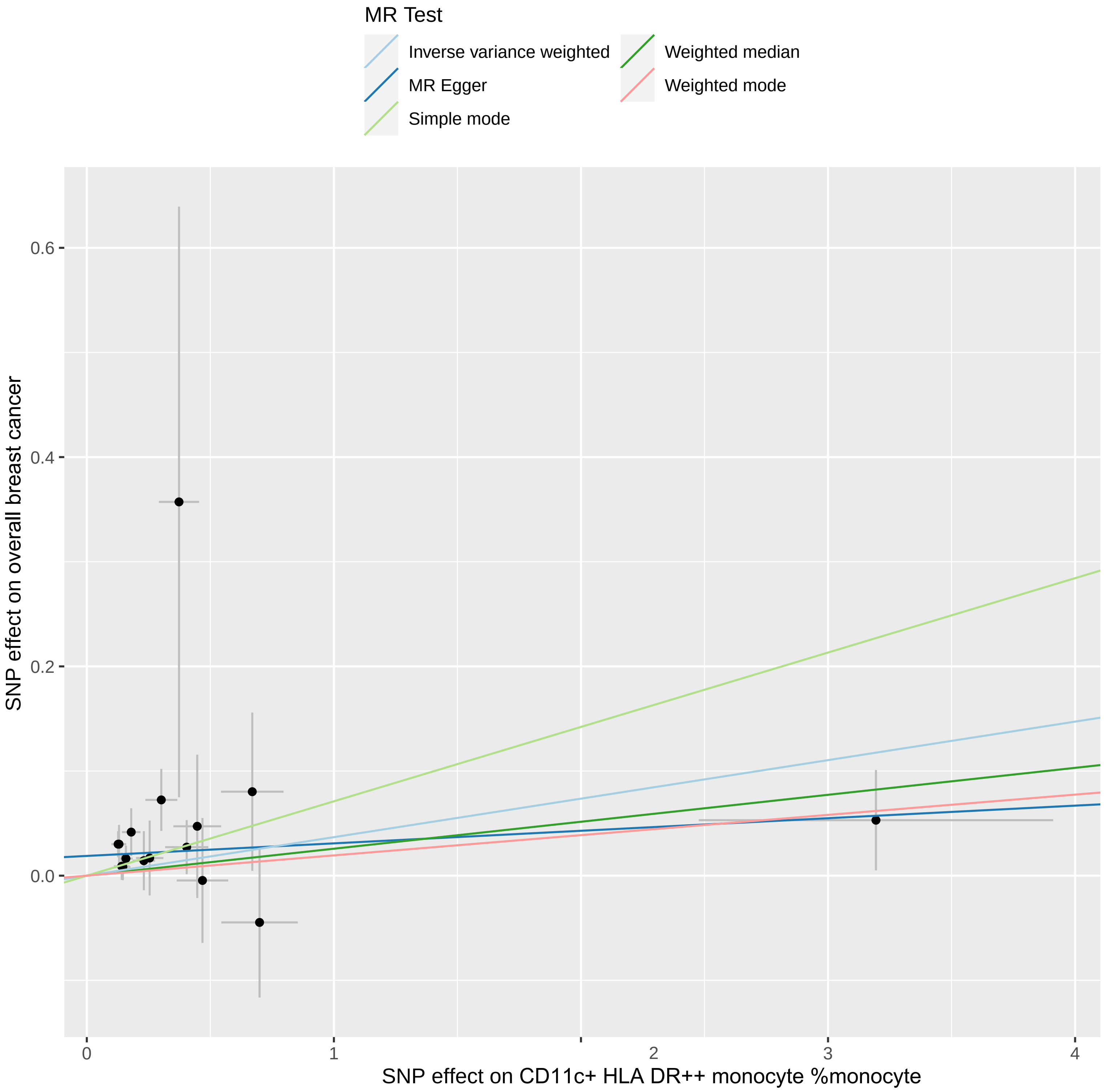

MR Test
Inverse variance weighted
MR Egger
Simple mode
Weighted median
Weighted mode
0.6
0.4
0.2
0.0
0
1
2
3
4
SNP effect on overall breast cancer
SNP effect on CD11c+ HLA DR++ monocyte %monocyte

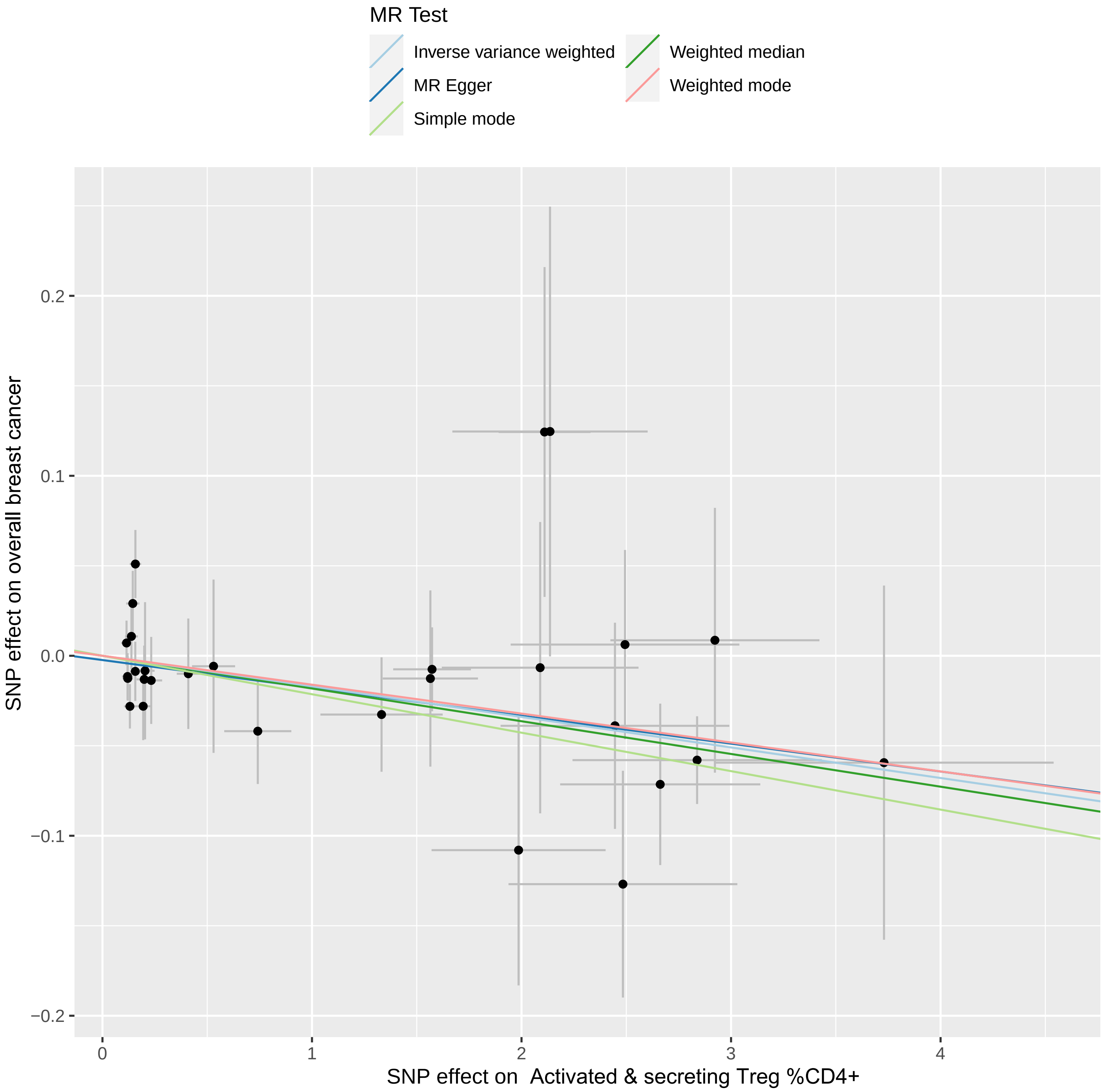
MR Test
Inverse variance weighted
MR Egger
Simple mode
Weighted median
Weighted mode
SNP effect on overall breast cancer
0.2
0.1
0.0
−0.1
−0.2
0
1
2
3
4
SNP effect on Activated & secreting Treg %CD4+

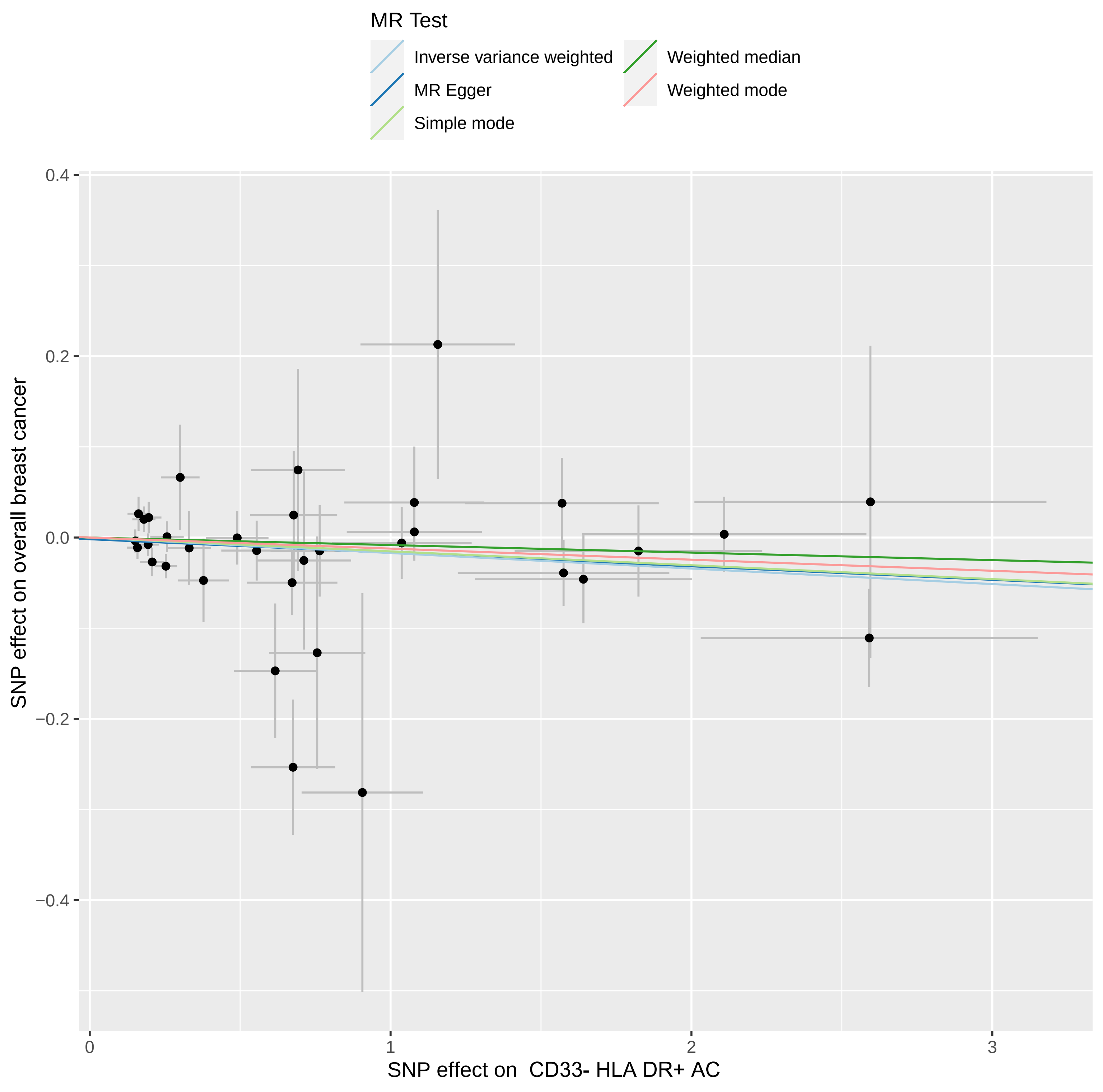

MR Test
Inverse variance weighted
MR Egger
Simple mode
Weighted median
Weighted mode
0.4
0.2
0.0
−0.2
−0.4
SNP effect on overall breast cancer
0
1
2
3
SNP effect on CD33- HLA DR+ AC

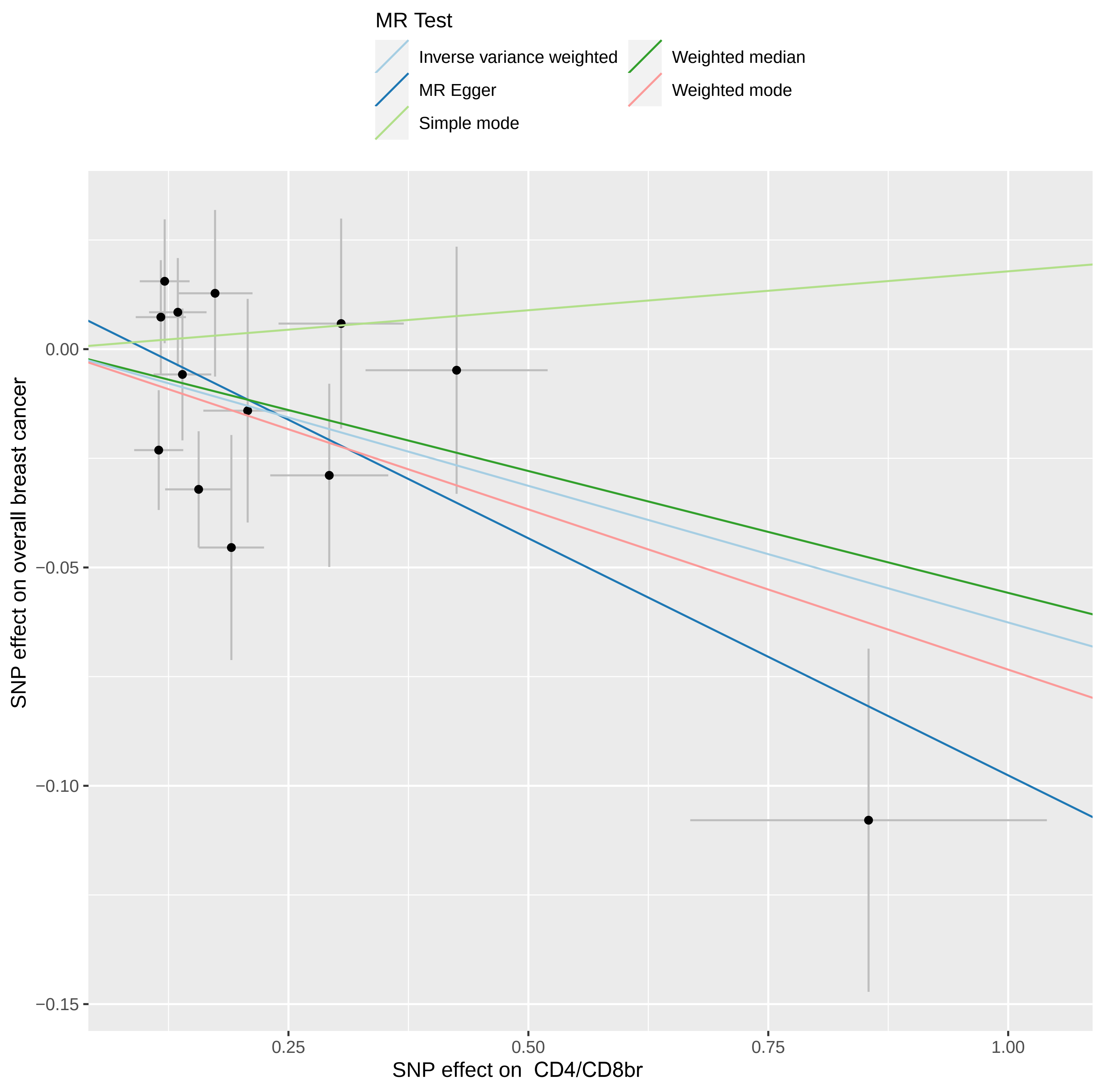

MR Test
Inverse variance weighted
MR Egger
Simple mode
Weighted median
Weighted mode
0.00
−0.05
−0.10
−0.15
0.25
0.50
0.75
1.00
SNP effect on overall breast cancer
SNP effect on CD4/CD8br

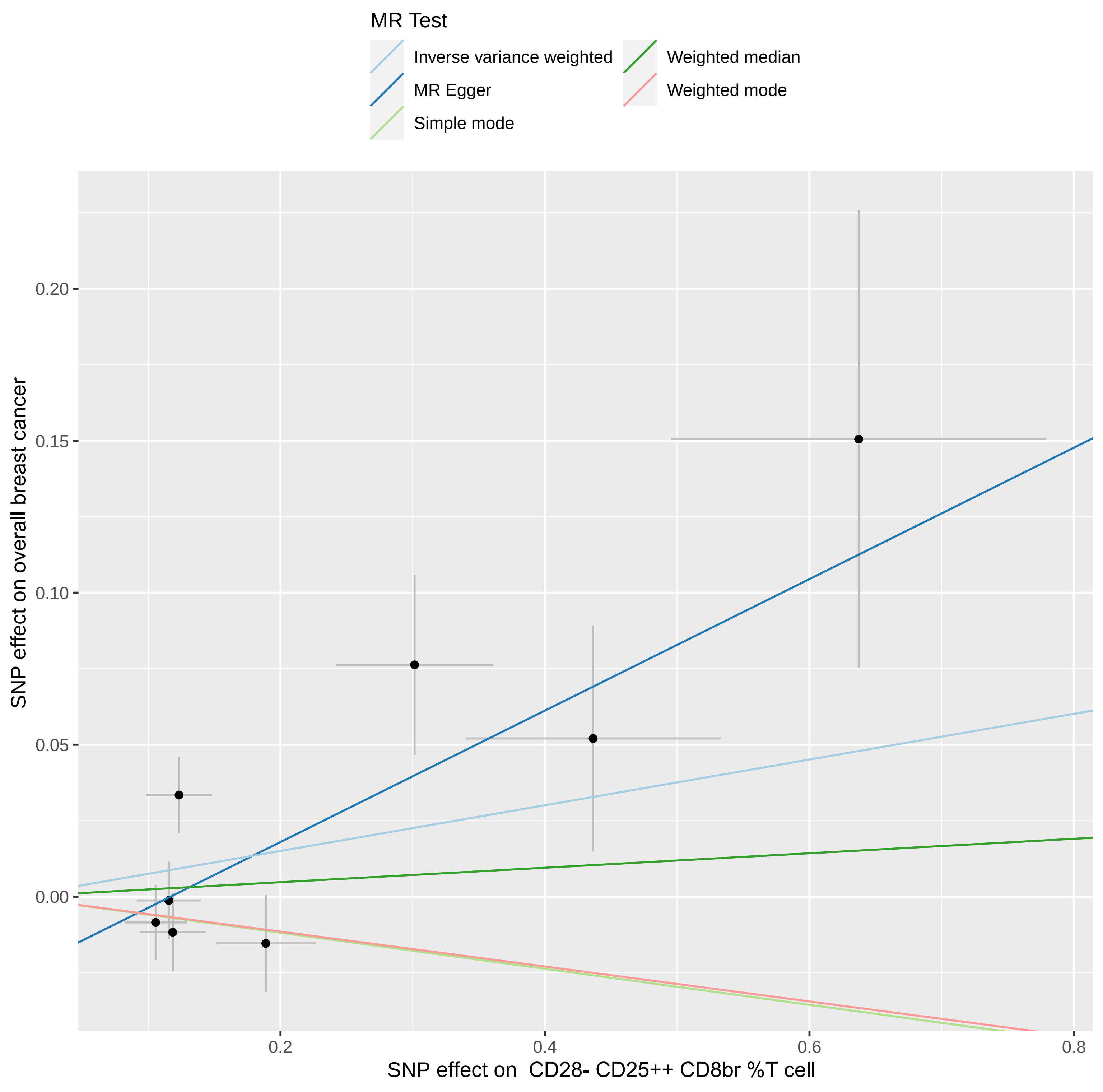

MR Test
Inverse variance weighted
MR Egger
Simple mode
Weighted median
Weighted mode
SNP effect on overall breast cancer
0.20
0.15
0.10
0.05
0.00
0.2
0.4
0.6
0.8
SNP effect on CD28- CD25++ CD8br %T cell

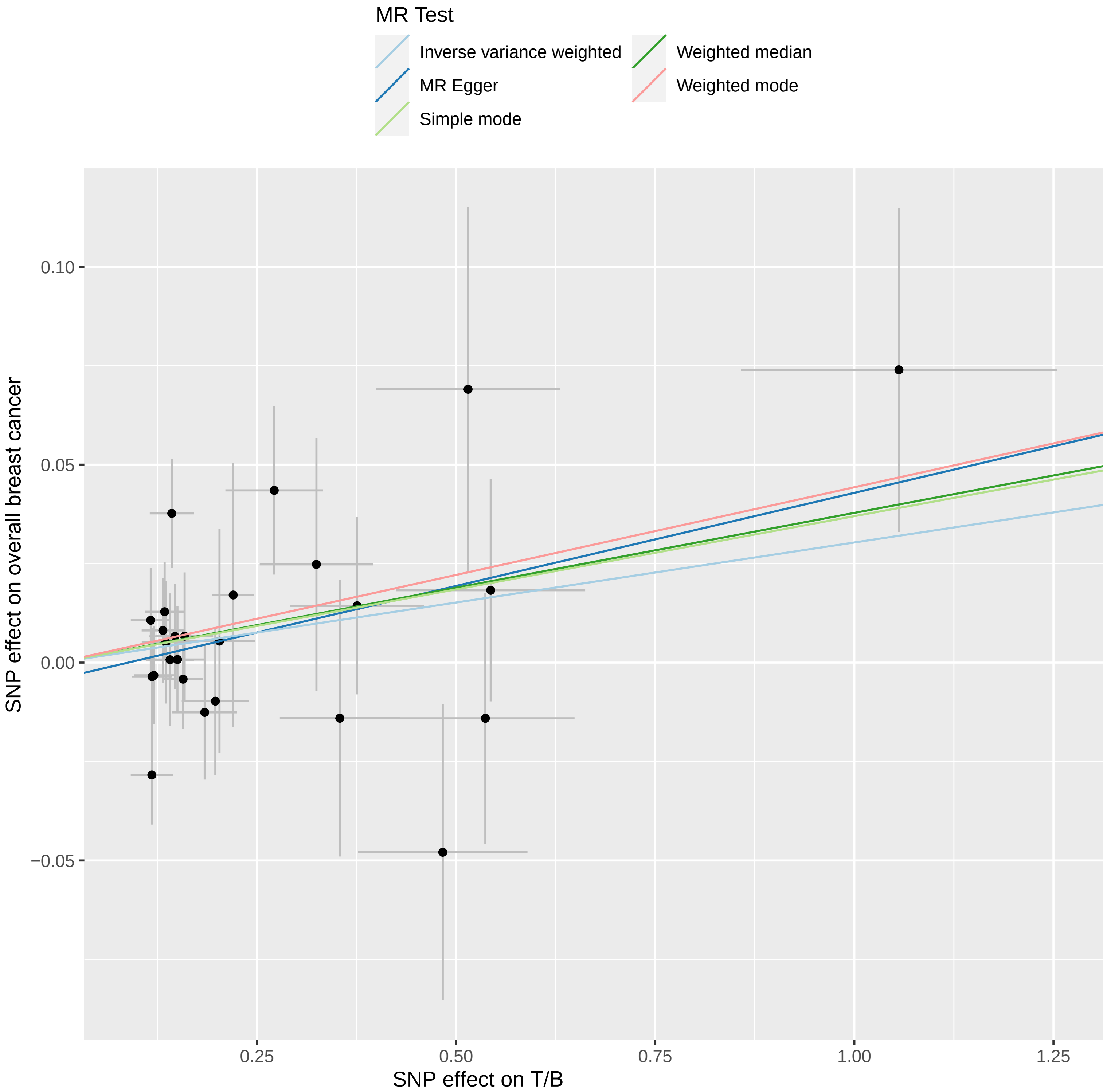

MR Test
Inverse variance weighted
MR Egger
Simple mode
Weighted median
Weighted mode
SNP effect on overall breast cancer
0.10
0.05
0.00
−0.05
0.25
0.50
0.75
1.00
1.25
SNP effect on T/B

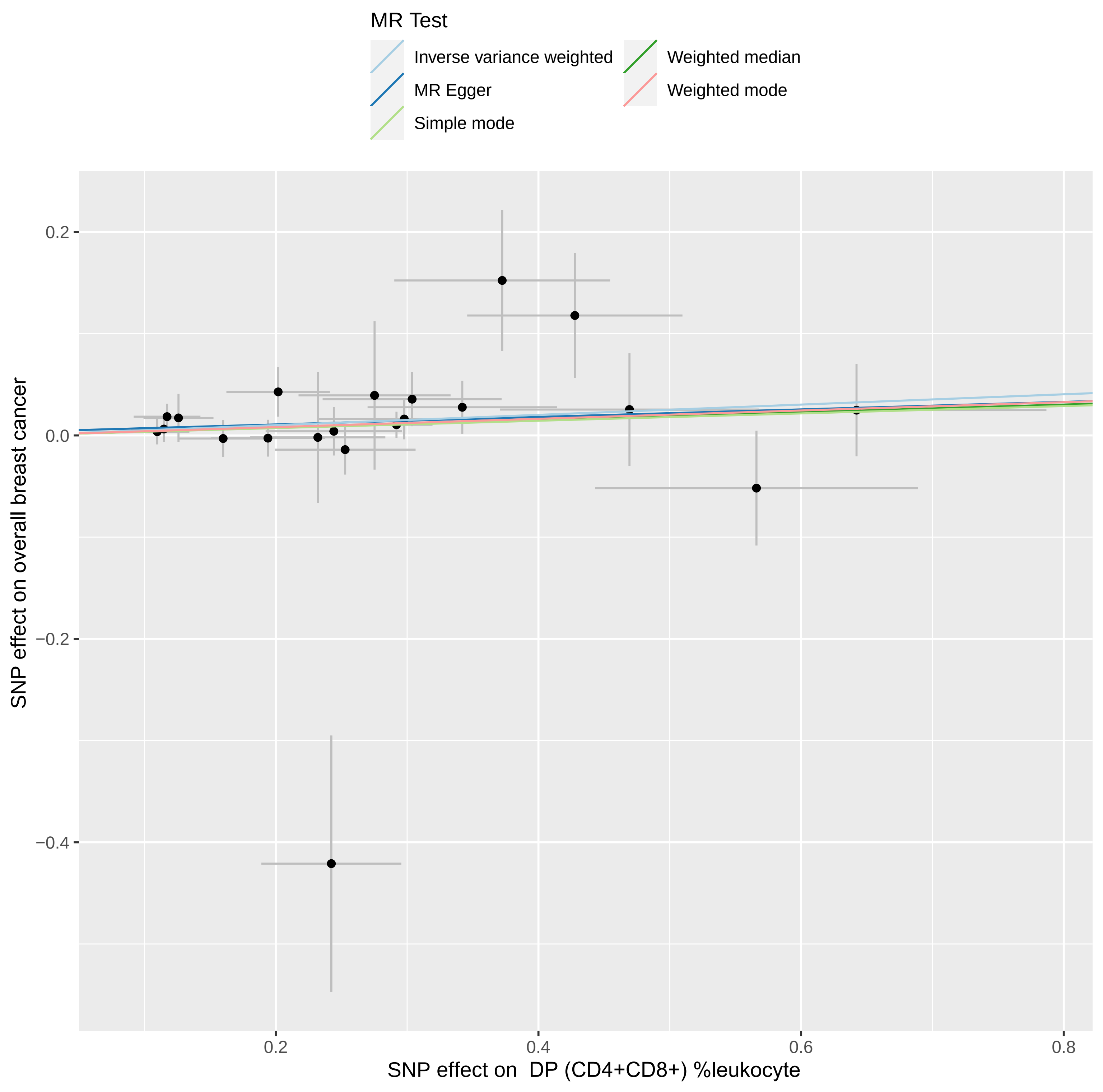
MR Test
Inverse variance weighted
MR Egger
Simple mode
Weighted median
Weighted mode
SNP effect on overall breast cancer
0.2
0.0
−0.2
−0.4
SNP effect on DP (CD4+CD8+) %leukocyte
0.2
0.4
0.6
0.8

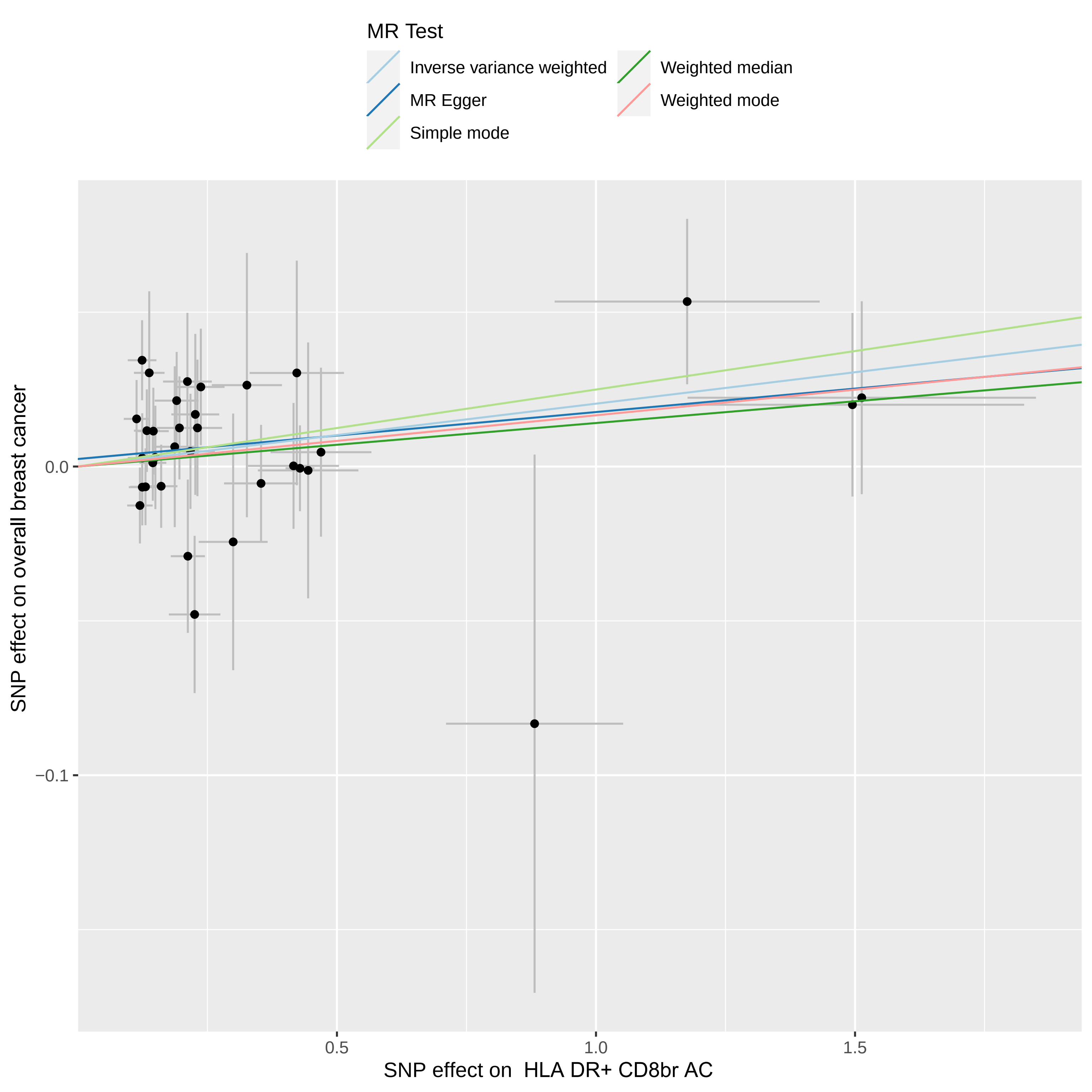
MR Test
Inverse variance weighted
MR Egger
Simple mode
Weighted median
Weighted mode
SNP effect on overall breast cancer
0.0
−0.1
0.5
1.0
1.5
SNP effect on HLA DR+ CD8br AC

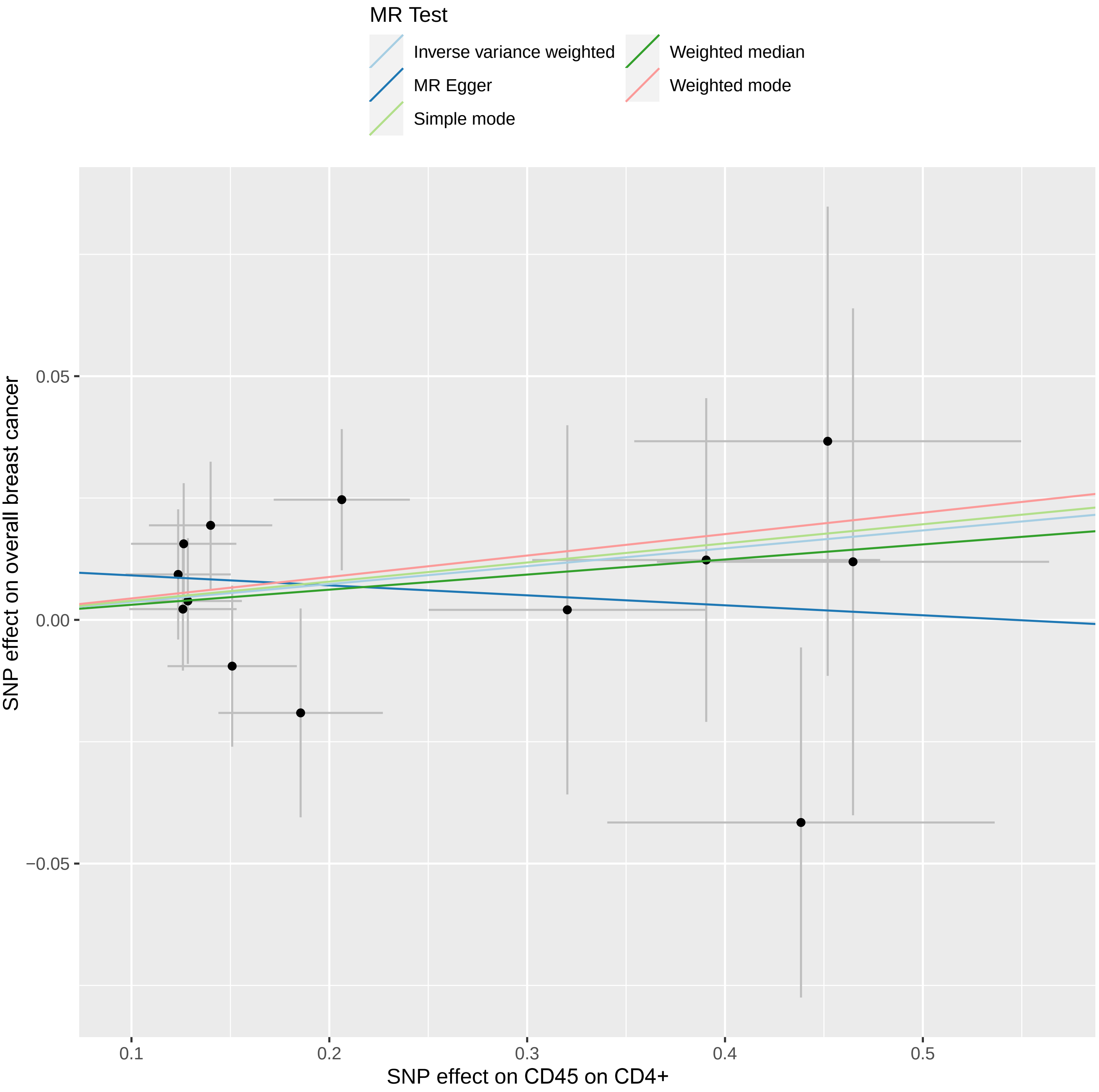
MR Test
Inverse variance weighted
MR Egger
Simple mode
Weighted median
Weighted mode
0.05
0.00
−0.05
0.1
0.2
0.3
0.4
0.5
SNP effect on overall breast cancer
SNP effect on CD45 on CD4+

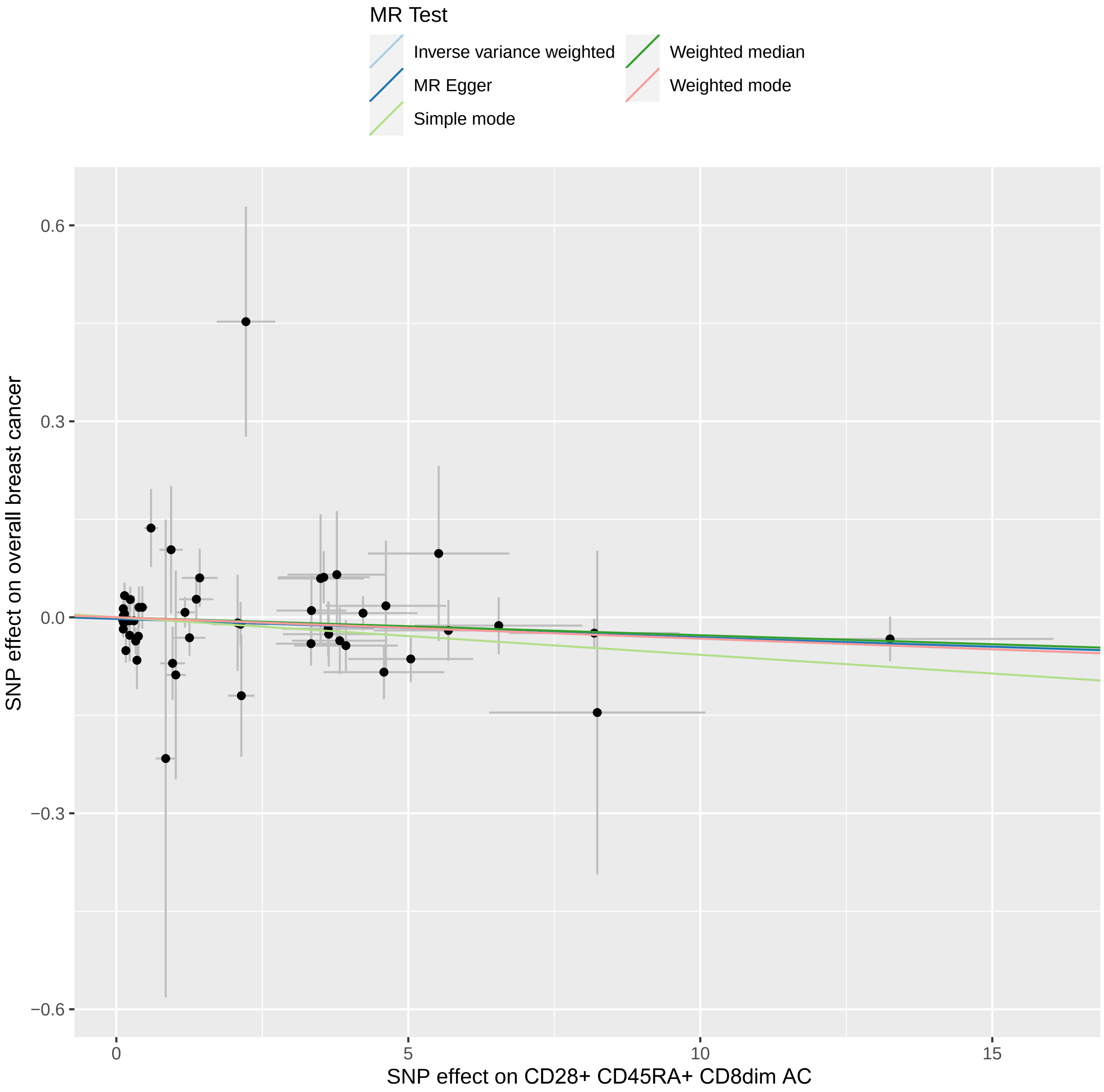
MR Test
Inverse variance weighted
MR Egger
Simple mode
Weighted median
Weighted mode
0.6
0.3
0.0
−0.3
−0.6
0
5
10
15
SNP effect on overall breast cancer
SNP effect on CD28+ CD45RA+ CD8dim AC

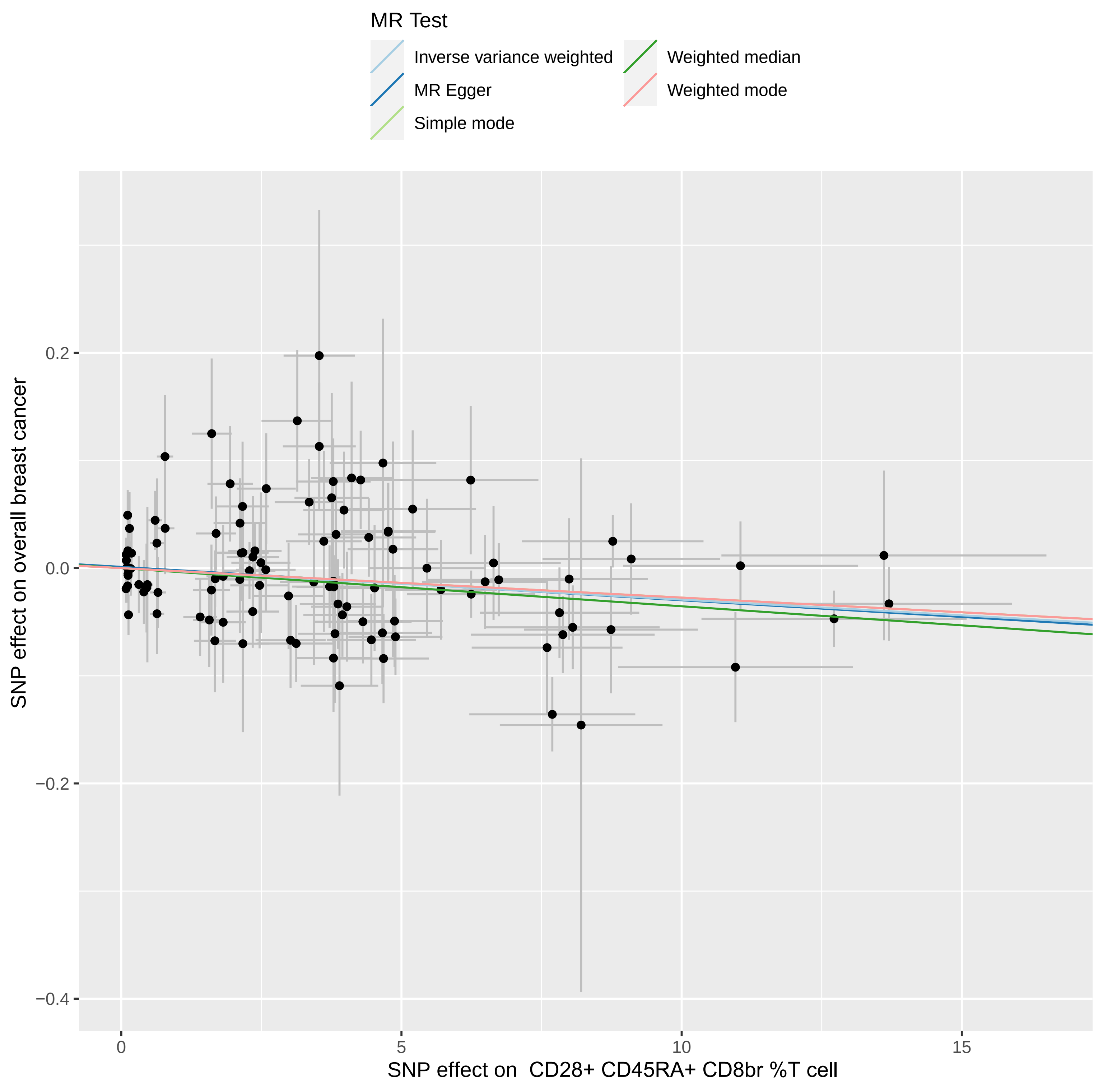

MR Test
Inverse variance weighted
MR Egger
Simple mode
Weighted median
Weighted mode
SNP effect on overall breast cancer
0.2
0.0
−0.2
−0.4
0
5
10
15
SNP effect on CD28+ CD45RA+ CD8br %T cell

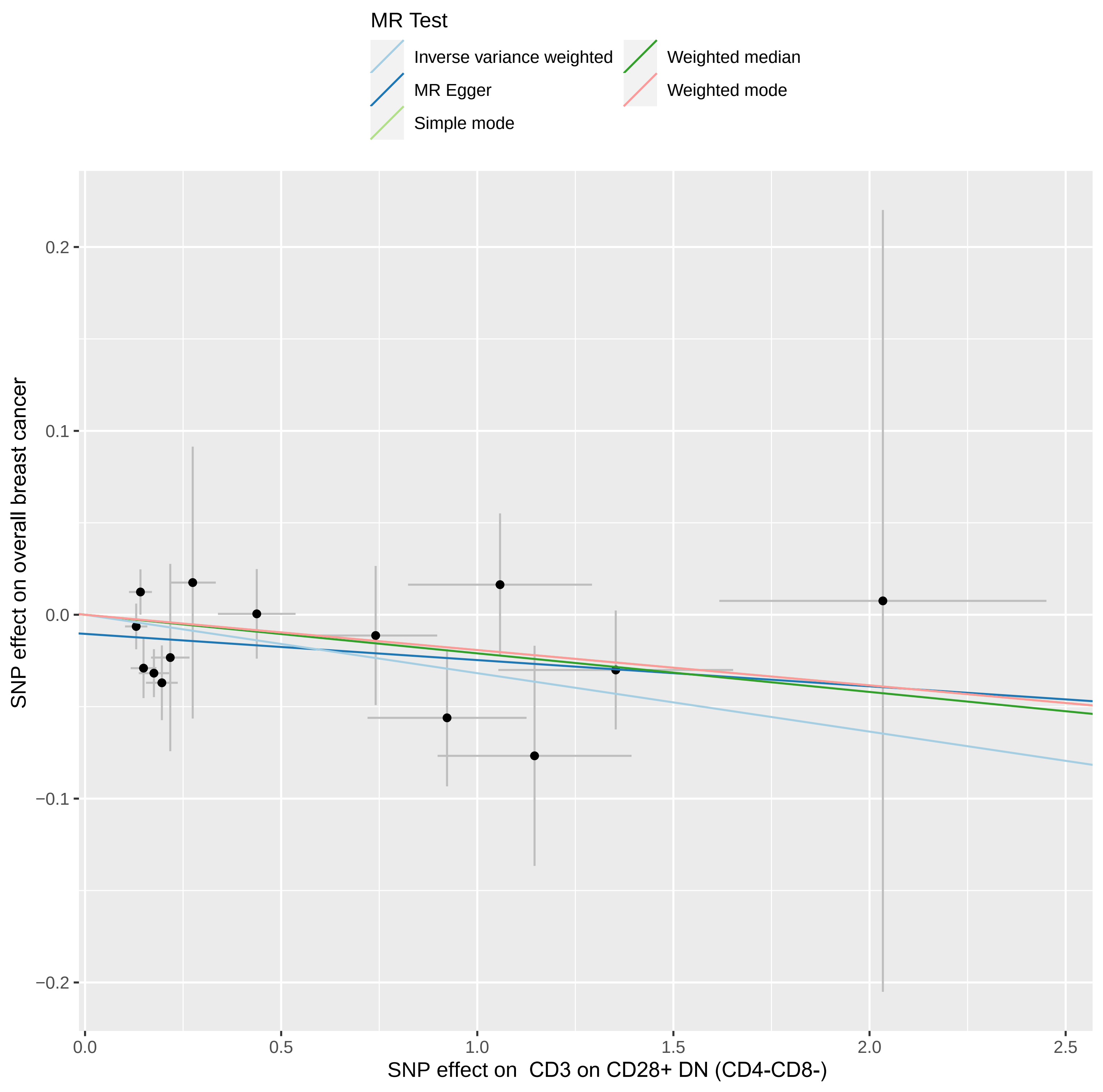

MR Test
Inverse variance weighted
MR Egger
Simple mode
Weighted median
Weighted mode
0.2
0.1
0.0
−0.1
−0.2
SNP effect on overall breast cancer
0.0
0.5
1.0
1.5
2.0
2.5
SNP effect on CD3 on CD28+ DN (CD4-CD8-)

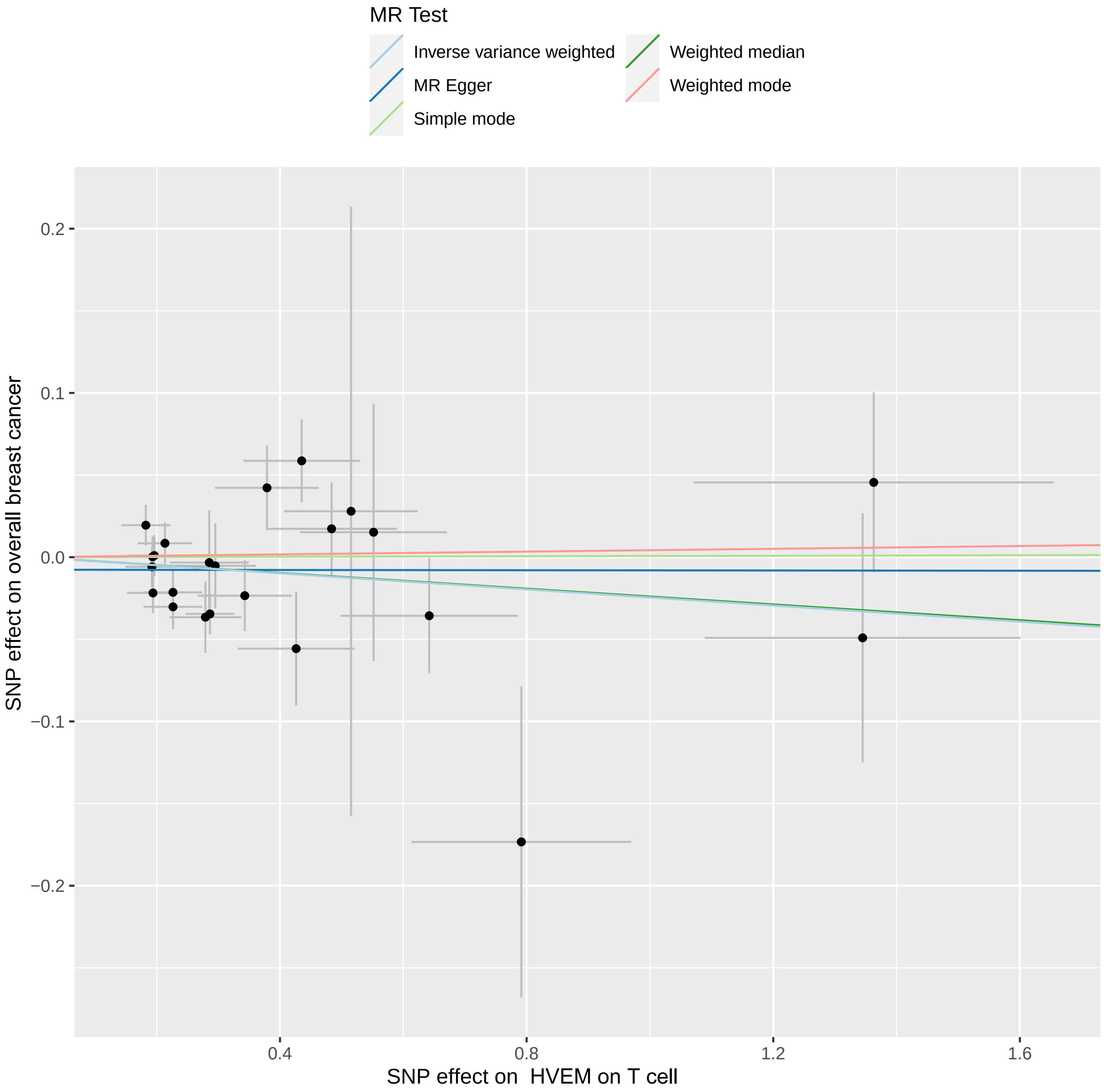

MR Test
Inverse variance weighted
MR Egger
Simple mode
Weighted median
Weighted mode
0.2
0.1
0.0
−0.1
−0.2
0.4
0.8
1.2
1.6
SNP effect on overall breast cancer
SNP effect on HVEM on T cell

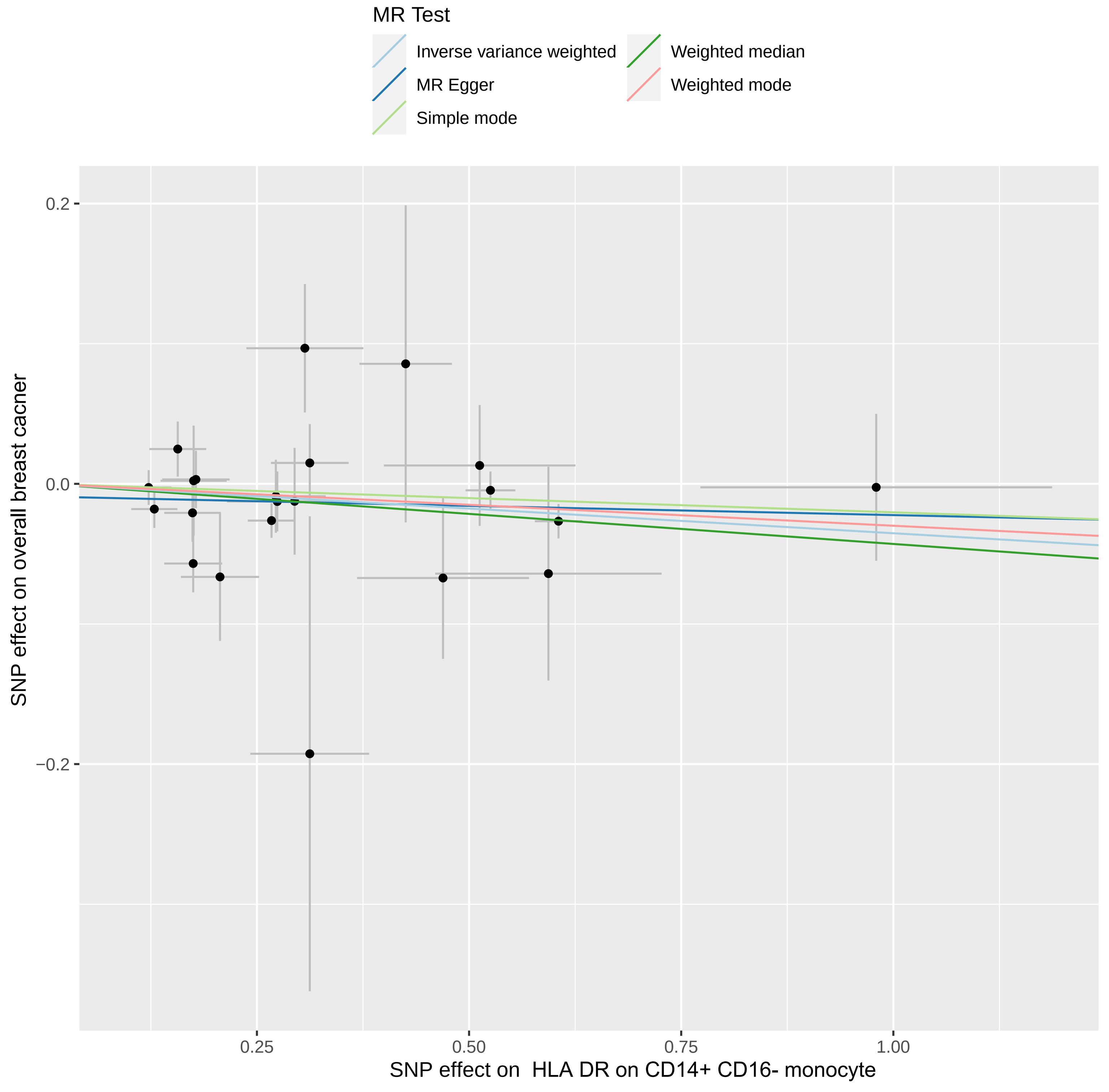

MR Test
Inverse variance weighted
MR Egger
Simple mode
Weighted median
Weighted mode
0.2
0.0
−0.2
0.25
0.50
0.75
1.00
SNP effect on overall breast cacner
SNP effect on HLA DR on CD14+ CD16- monocyte

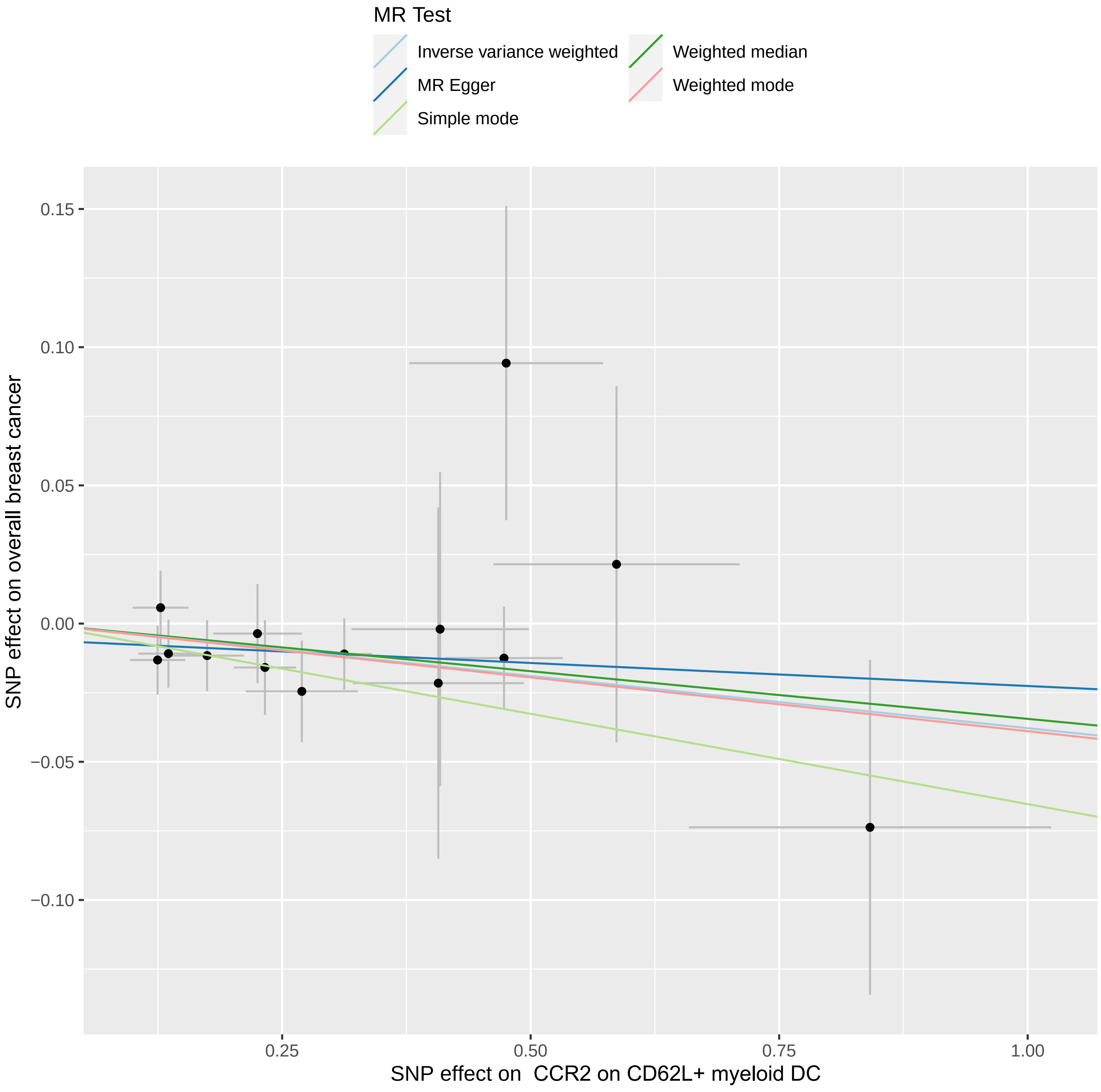

MR Test
Inverse variance weighted
MR Egger
Simple mode
Weighted median
Weighted mode
0.15
0.10
0.05
0.00
−0.05
−0.10
0.25
0.50
0.75
1.00
SNP effect on overall breast cancer
SNP effect on CCR2 on CD62L+ myeloid DC

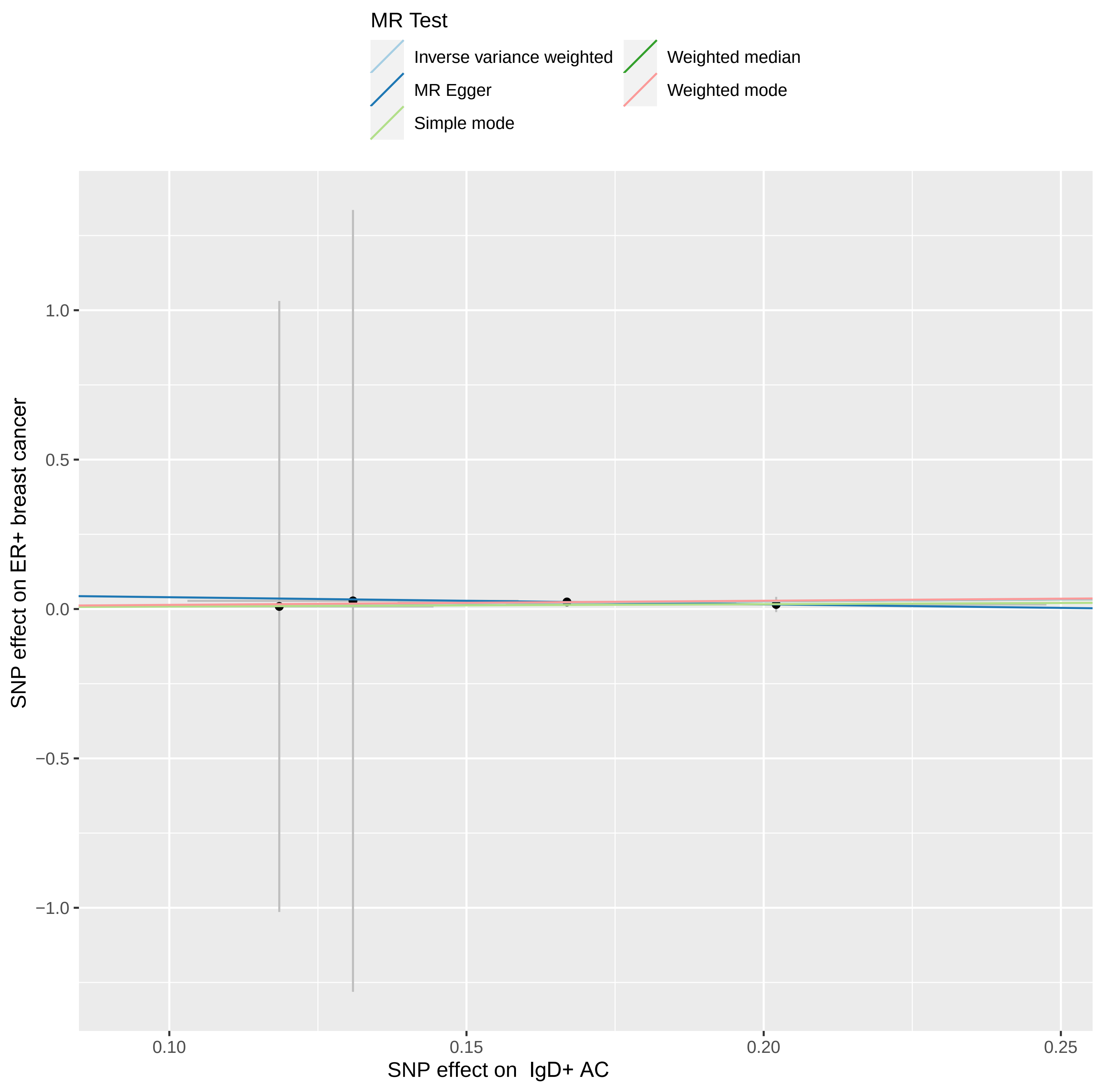

MR Test
Inverse variance weighted
MR Egger
Simple mode
Weighted median
Weighted mode
1.0
0.5
0.0
−0.5
−1.0
0.10
0.15
0.20
0.25
SNP effect on ER+ breast cancer
SNP effect on IgD+ AC

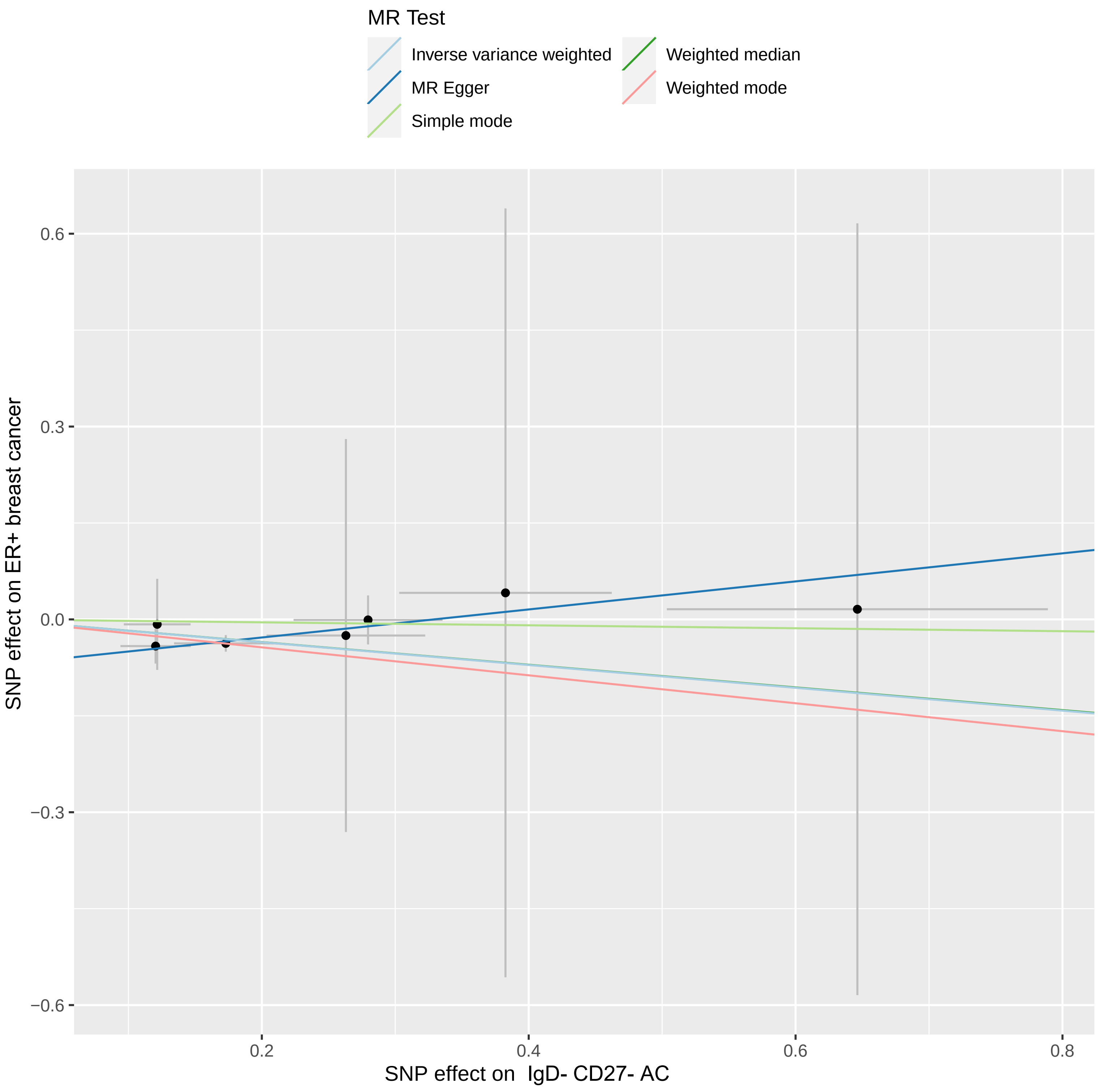
MR Test
Inverse variance weighted
MR Egger
Simple mode
Weighted median
Weighted mode
SNP effect on ER+ breast cancer
0.6
0.3
0.0
−0.3
−0.6
0.2
0.4
0.6
0.8
SNP effect on IgD- CD27- AC

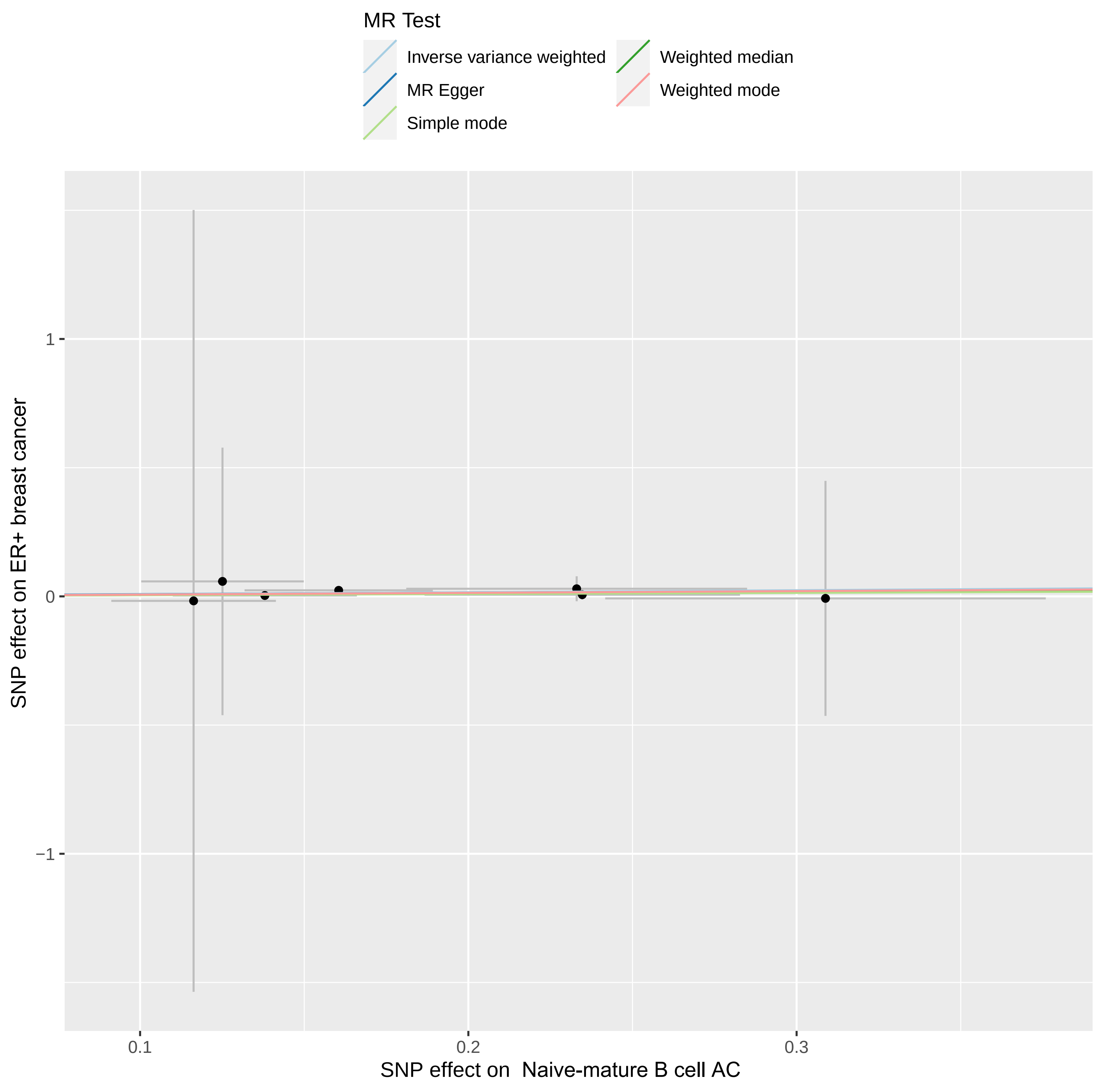
MR Test
Inverse variance weighted
MR Egger
Simple mode
Weighted median
Weighted mode
1
0
−1
0.1
0.2
0.3
SNP effect on ER+ breast cancer
SNP effect on Naive-mature B cell AC

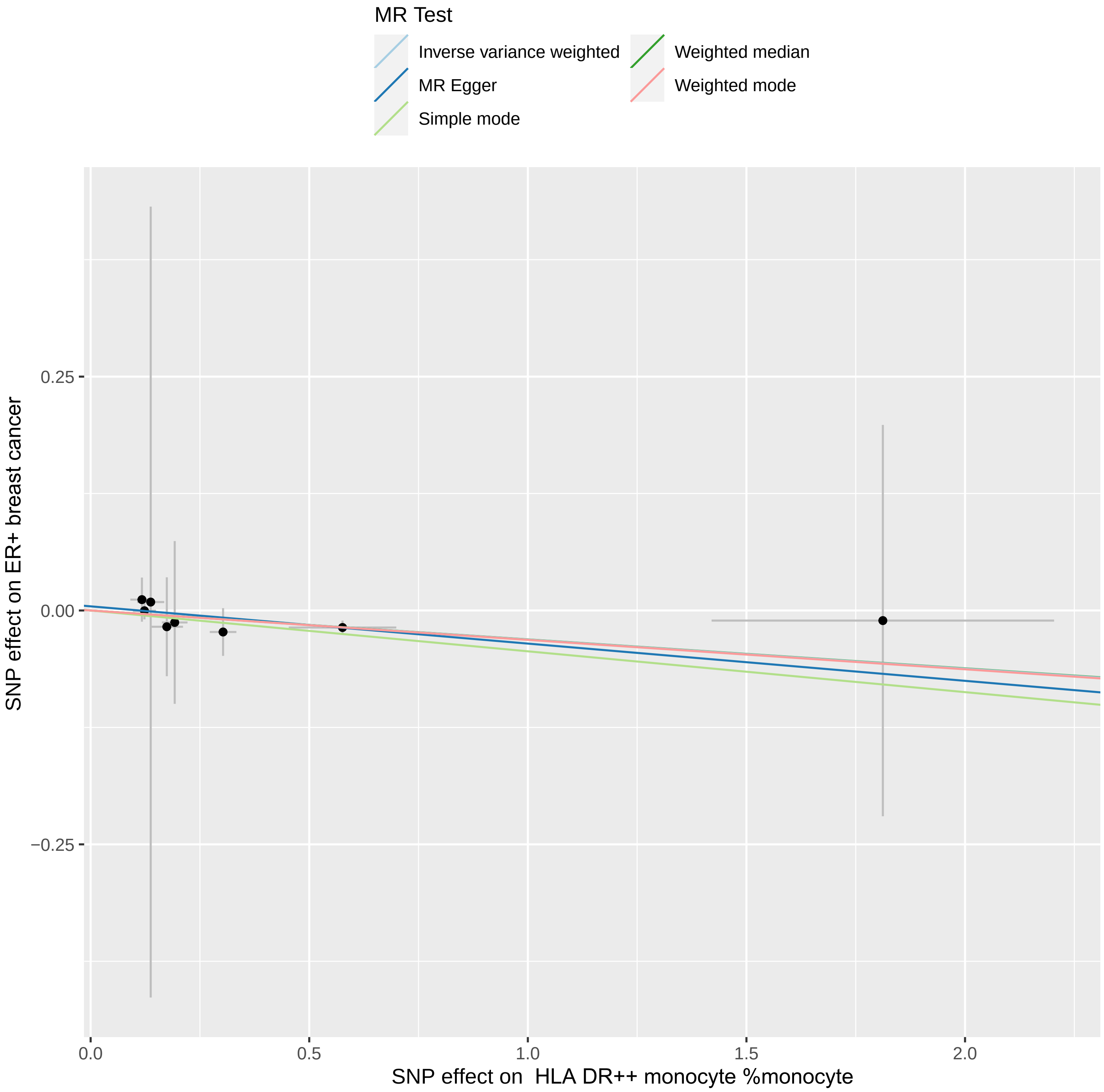

MR Test
Inverse variance weighted
MR Egger
Simple mode
Weighted median
Weighted mode
0.25
0.00
−0.25
0.0
0.5
1.0
1.5
2.0
SNP effect on ER+ breast cancer
SNP effect on HLA DR++ monocyte %monocyte

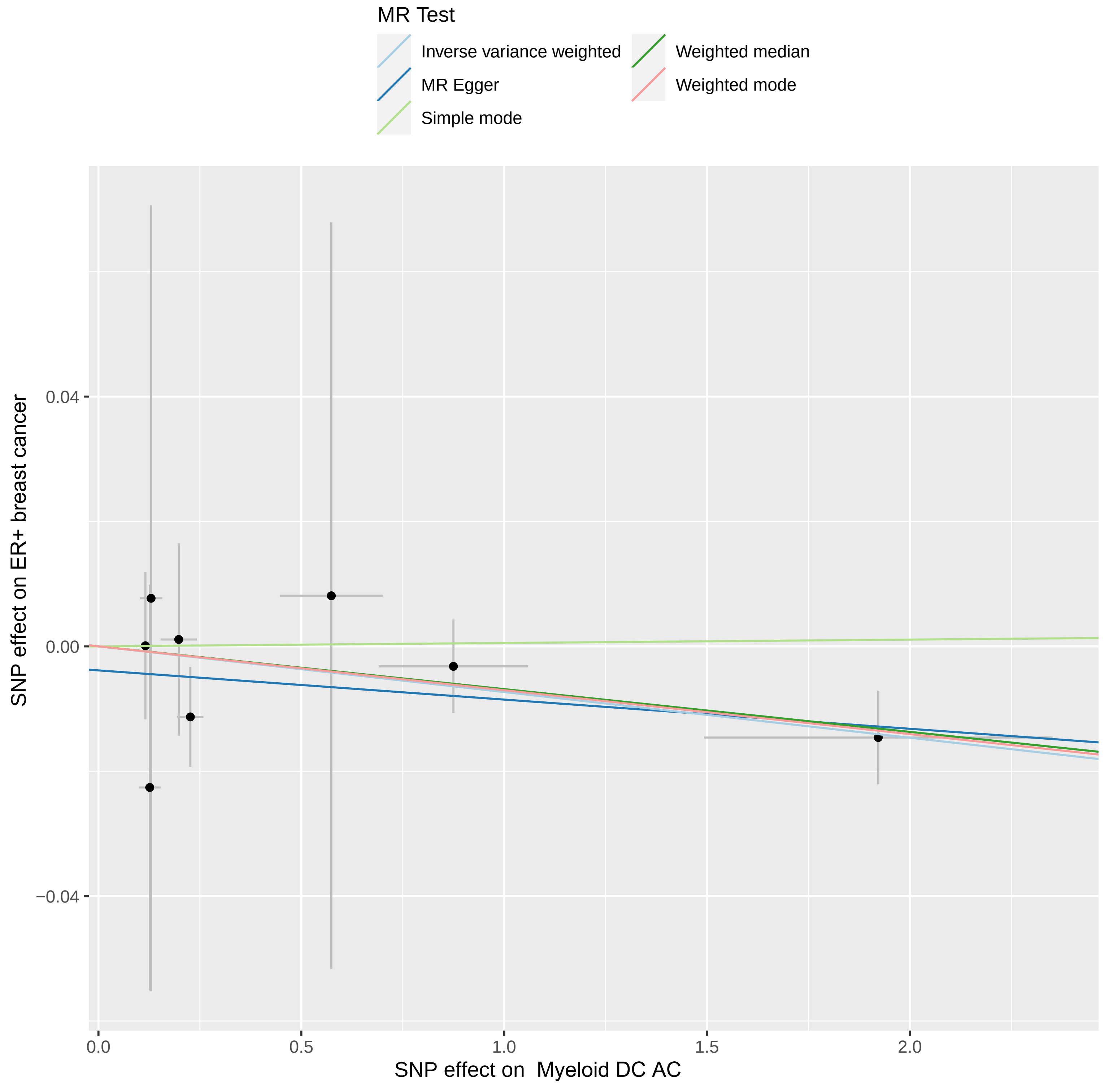

MR Test
Inverse variance weighted
MR Egger
Simple mode
Weighted median
Weighted mode
0.04
0.00
−0.04
0.0
0.5
1.0
1.5
2.0
SNP effect on ER+ breast cancer
SNP effect on Myeloid DC AC

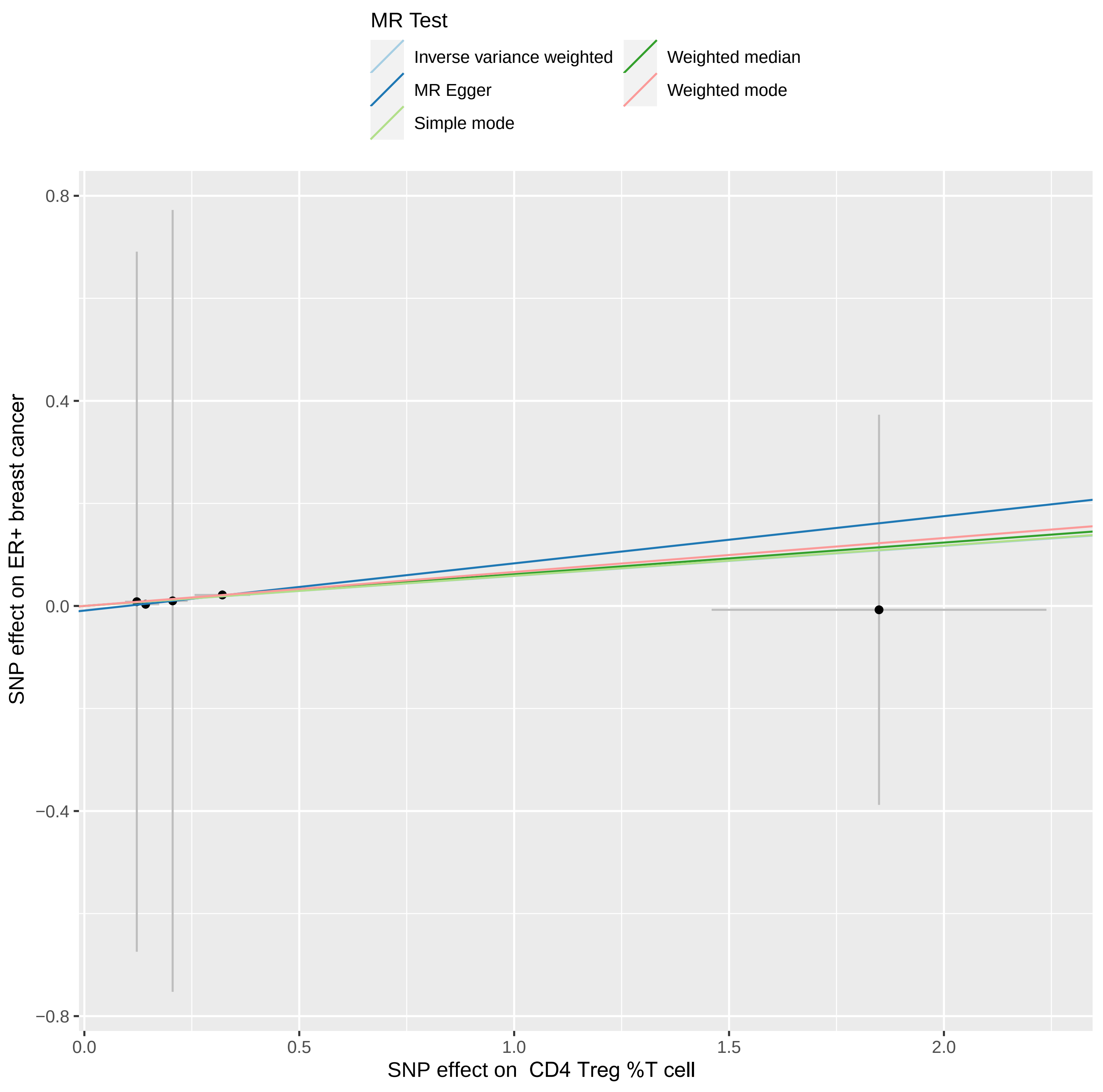

MR Test
Inverse variance weighted
MR Egger
Simple mode
Weighted median
Weighted mode
0.8
0.4
0.0
−0.4
−0.8
0.0
0.5
1.0
1.5
2.0
SNP effect on ER+ breast cancer
SNP effect on CD4 Treg %T cell

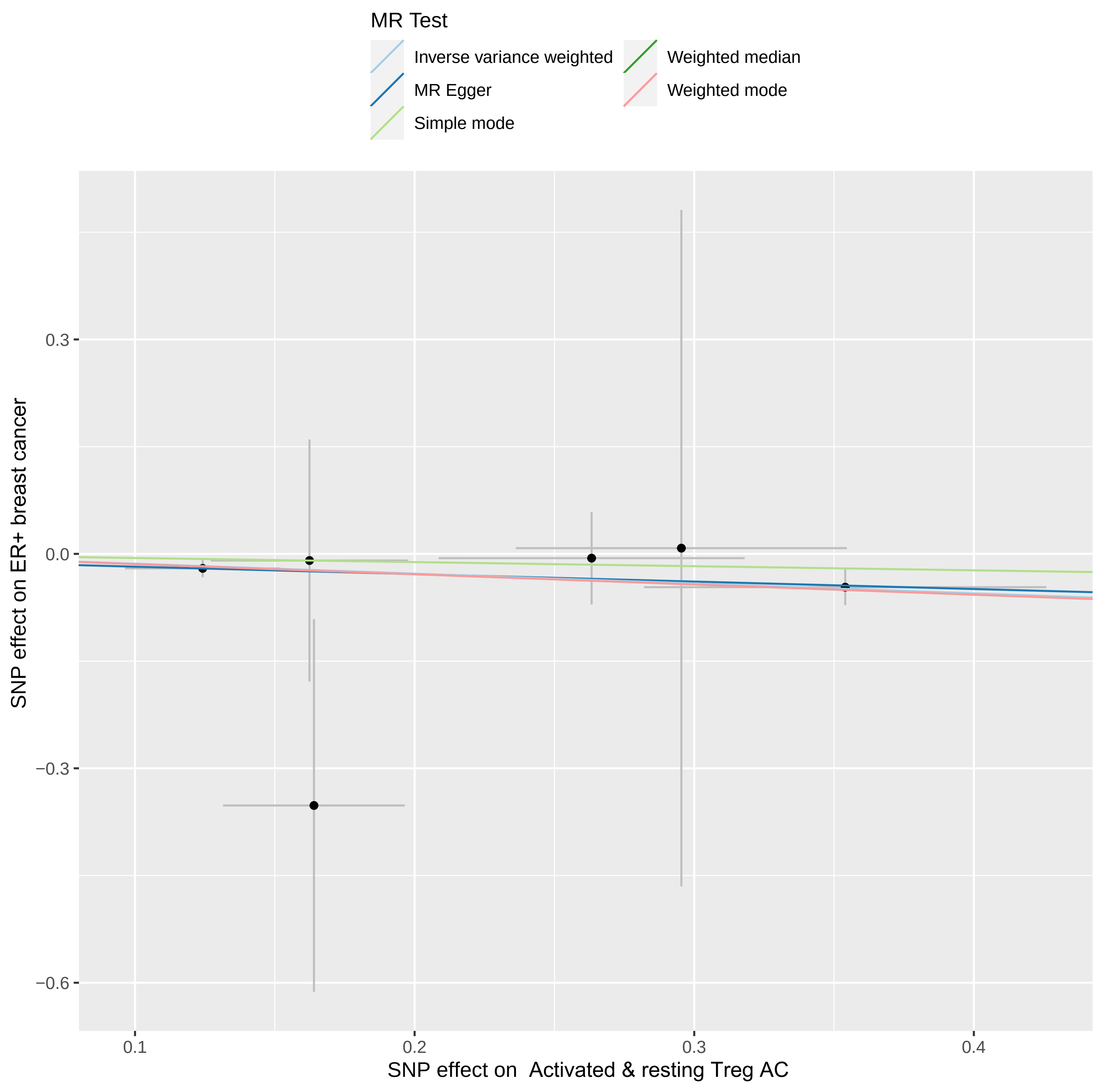

MR Test
Inverse variance weighted
MR Egger
Simple mode
Weighted median
Weighted mode
0.3
0.0
−0.3
−0.6
0.1
0.2
0.3
0.4
SNP effect on ER+ breast cancer
SNP effect on Activated & resting Treg AC

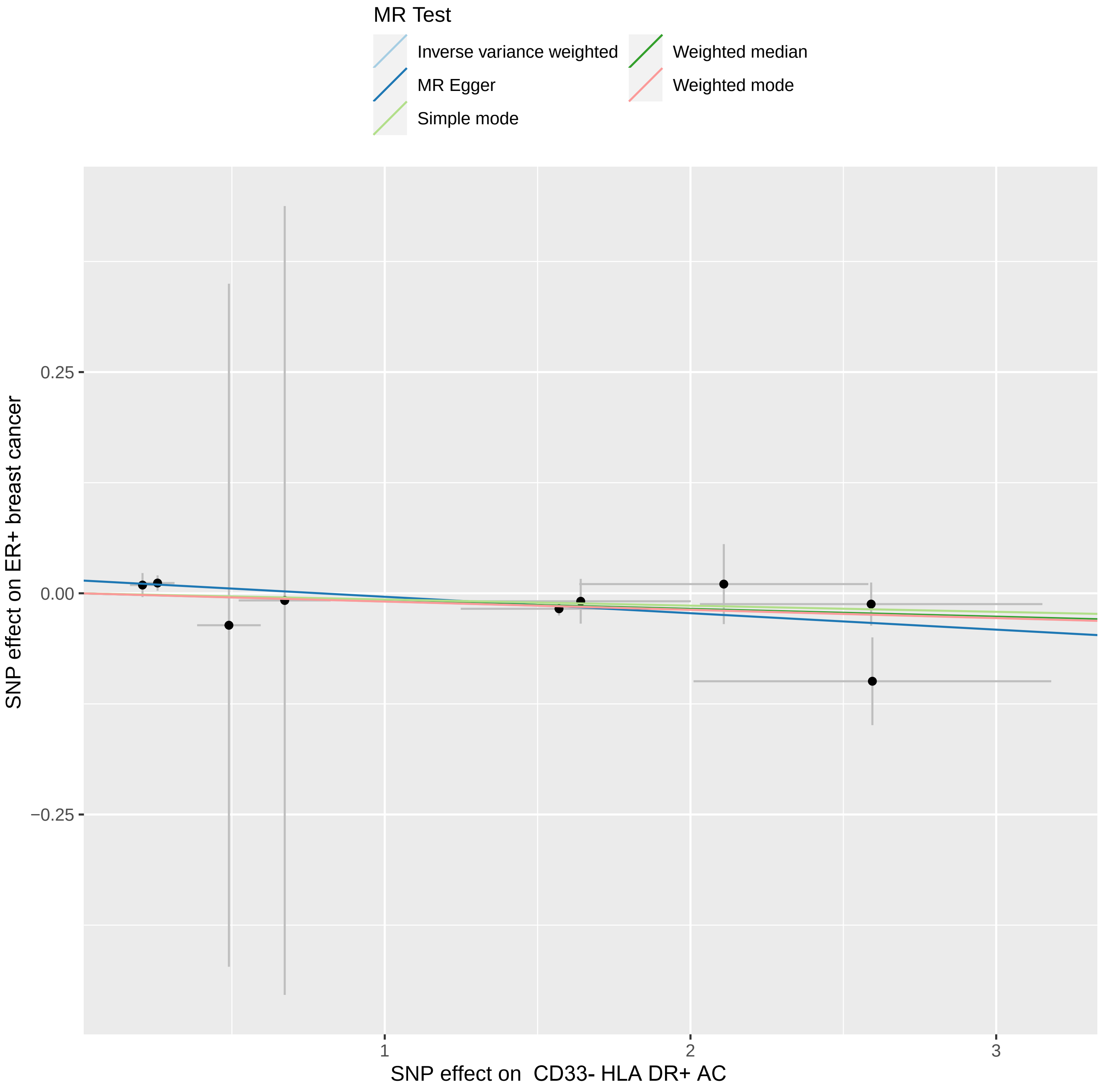
MR Test
Inverse variance weighted
MR Egger
Simple mode
Weighted median
Weighted mode
0.25
0.00
−0.25
1
2
3
SNP effect on ER+ breast cancer
SNP effect on CD33- HLA DR+ AC

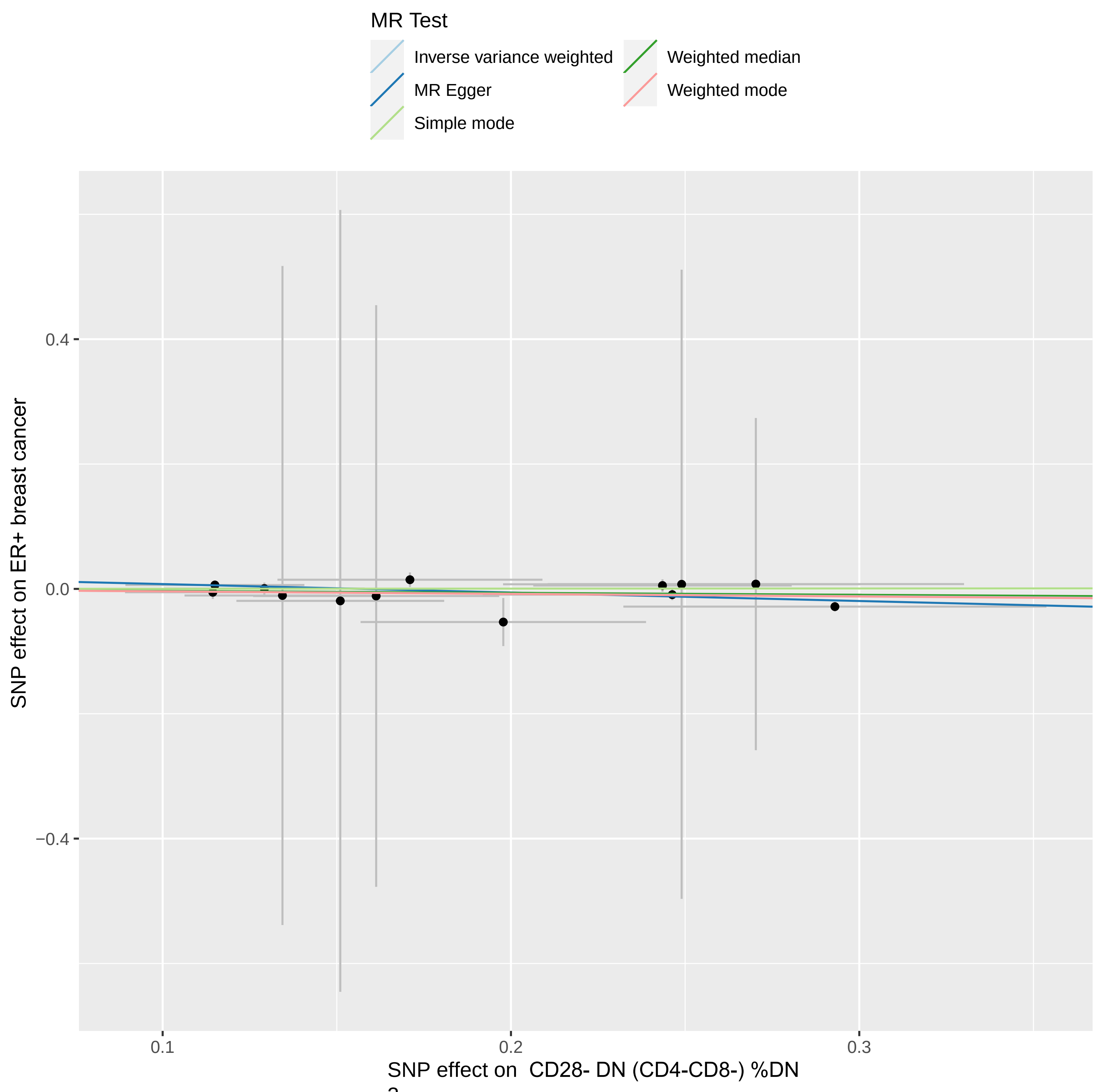
MR Test
Inverse variance weighted
MR Egger
Simple mode
Weighted median
Weighted mode
SNP effect on ER+ breast cancer
0.4
0.0
−0.4
0.1
0.2
0.3
SNP effect on CD28- DN (CD4-CD8-) %DN
2

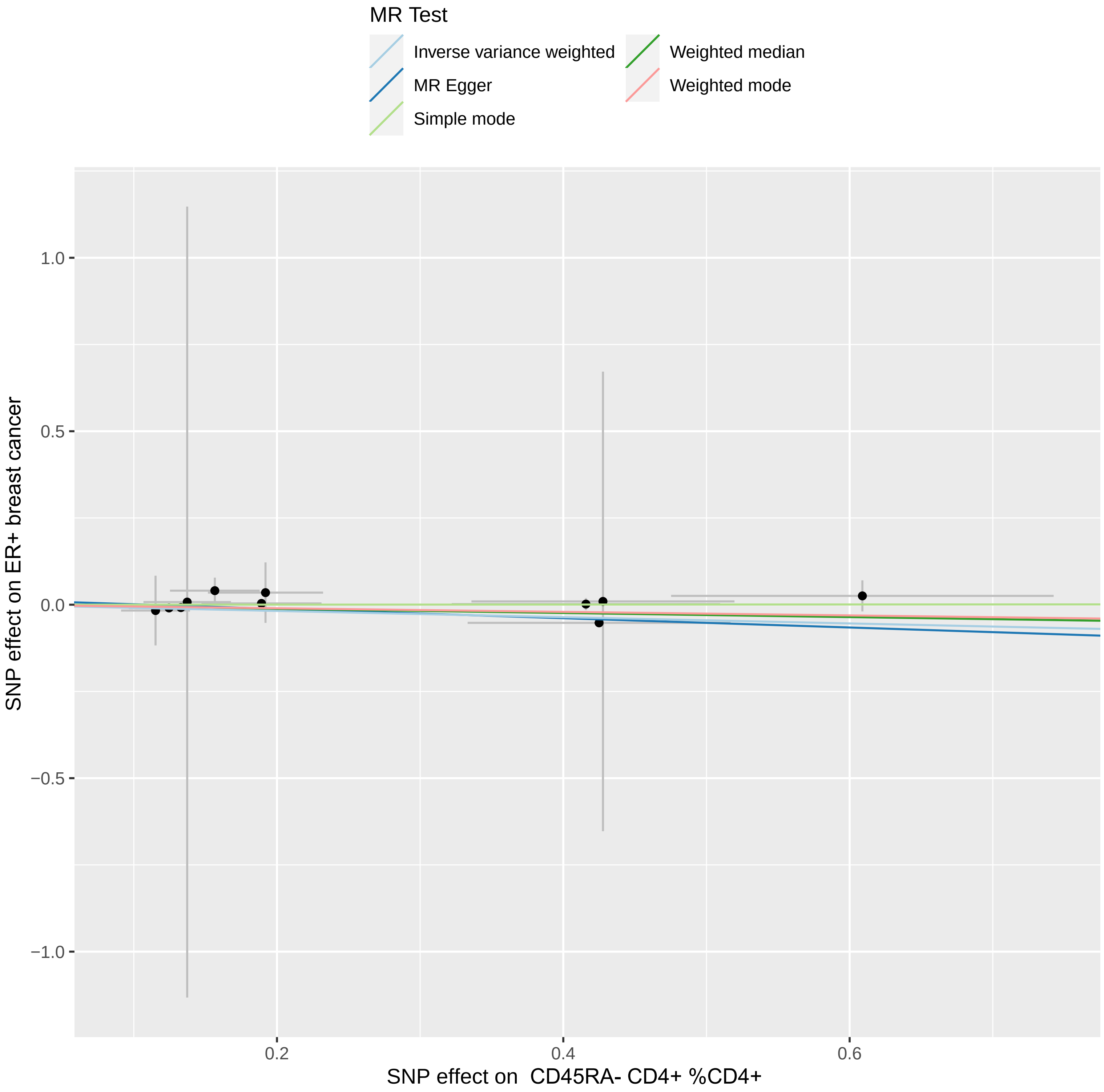
MR Test
Inverse variance weighted
MR Egger
Simple mode
Weighted median
Weighted mode
SNP effect on ER+ breast cancer
1.0
0.5
0.0
−0.5
−1.0
0.2
0.4
0.6
SNP effect on CD45RA- CD4+ %CD4+

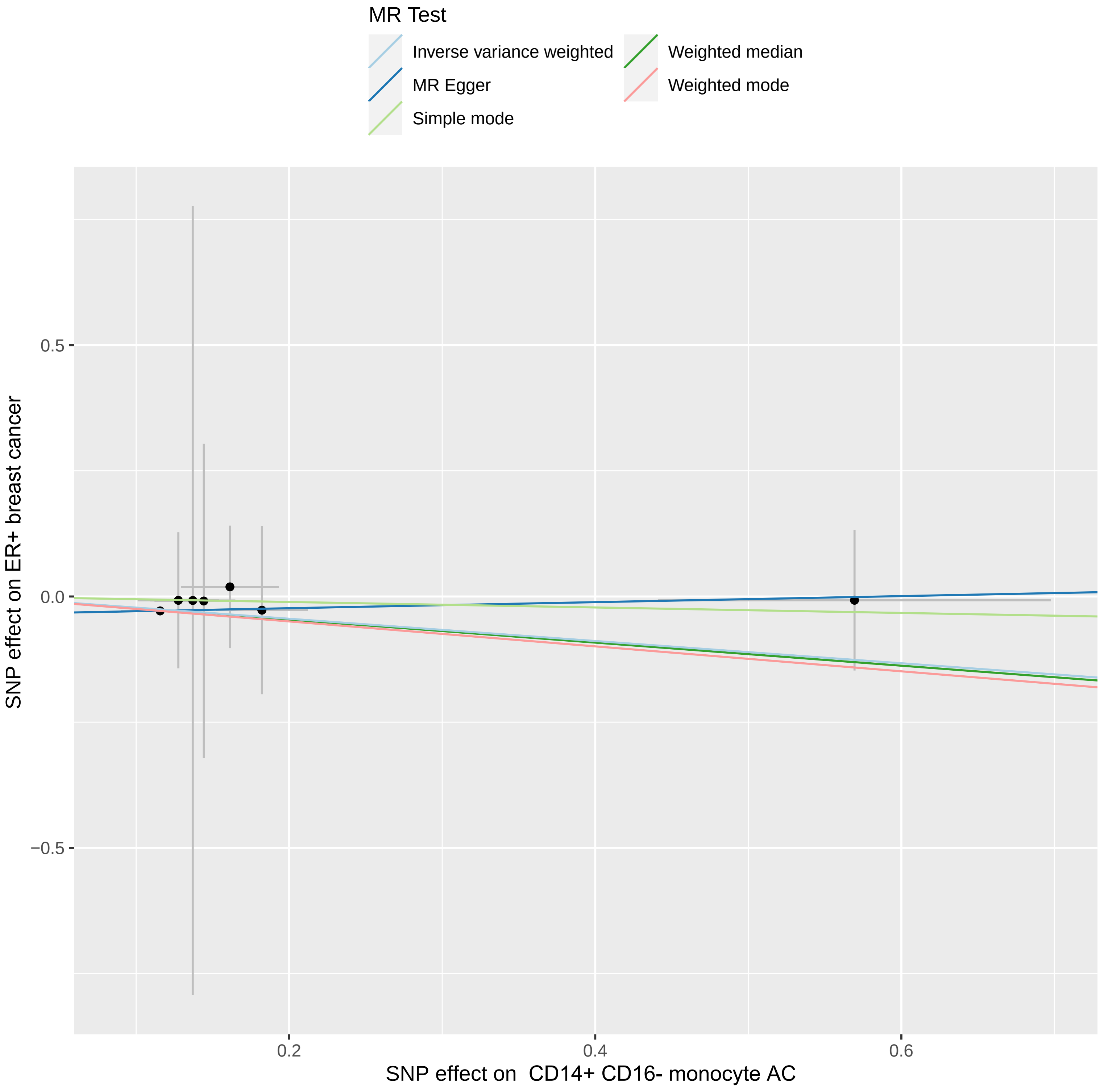

MR Test
Inverse variance weighted
MR Egger
Simple mode
Weighted median
Weighted mode
0.5
0.0
−0.5
0.2
0.4
0.6
SNP effect on ER+ breast cancer
SNP effect on CD14+ CD16- monocyte AC

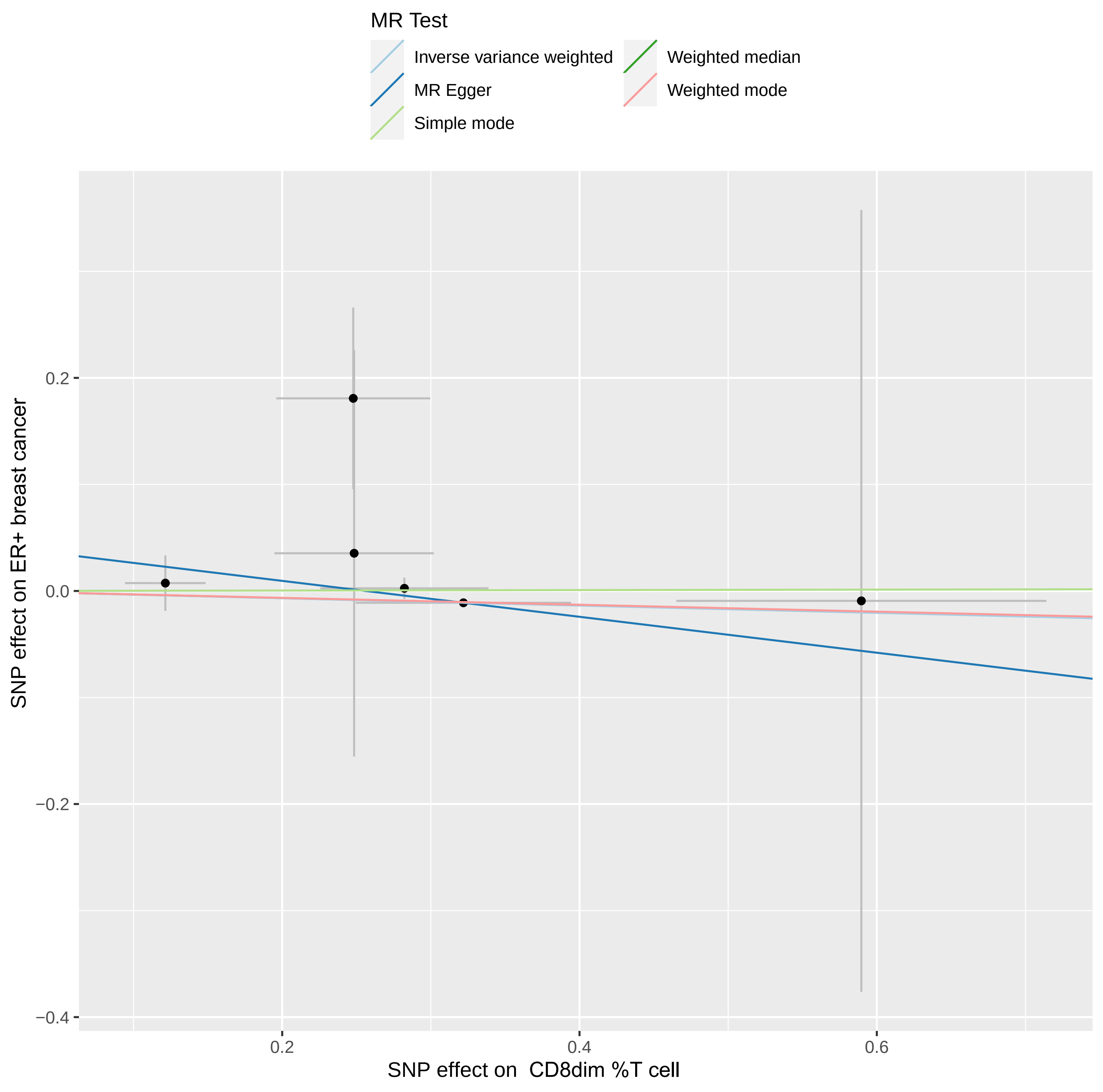
MR Test
Inverse variance weighted
MR Egger
Simple mode
Weighted median
Weighted mode
SNP effect on ER+ breast cancer
0.2
0.0
−0.2
−0.4
0.2
0.4
0.6
SNP effect on CD8dim %T cell

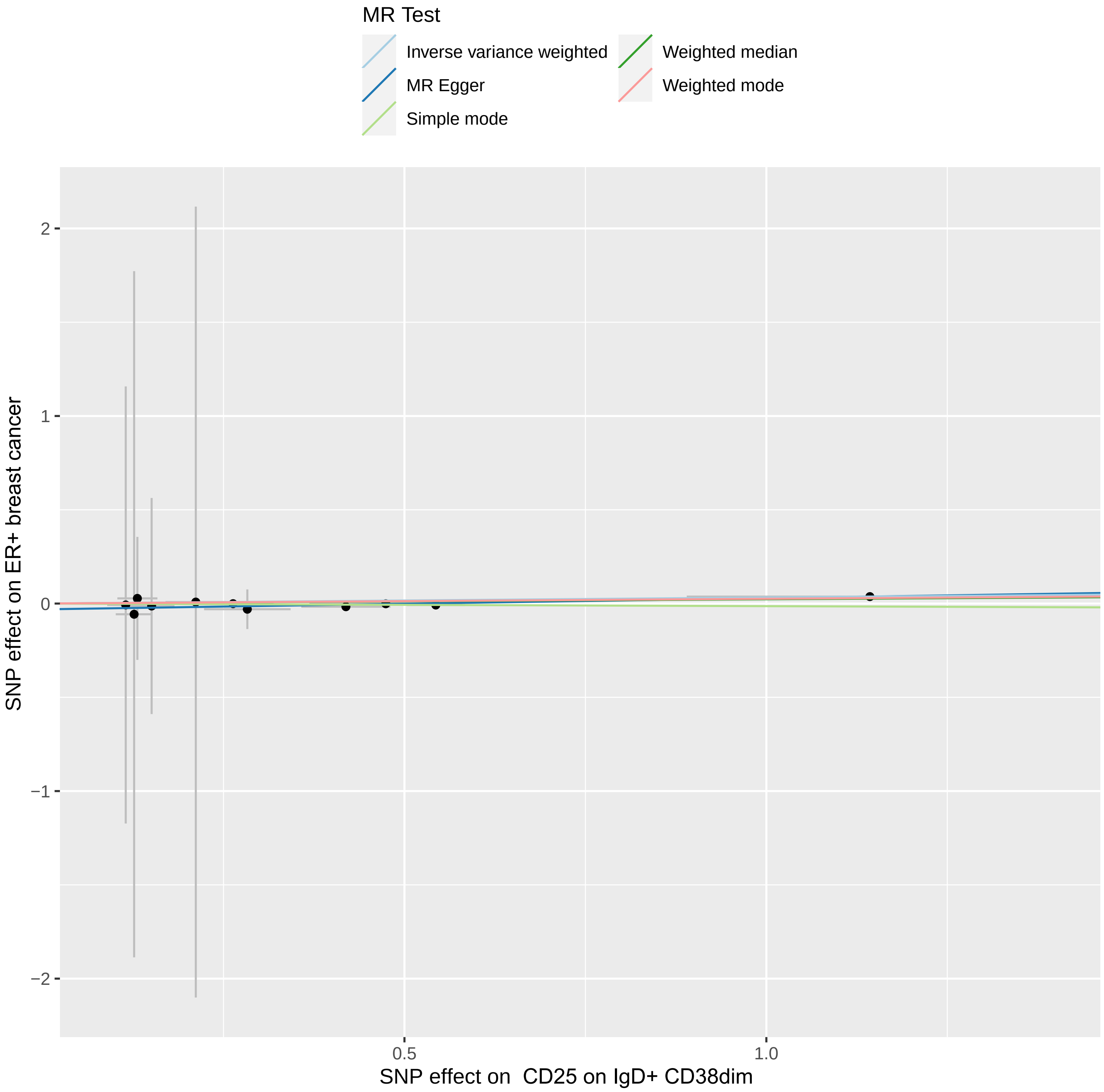

MR Test
Inverse variance weighted
MR Egger
Simple mode
Weighted median
Weighted mode
2
1
0
−1
−2
0.5
1.0
SNP effect on ER+ breast cancer
SNP effect on CD25 on IgD+ CD38dim

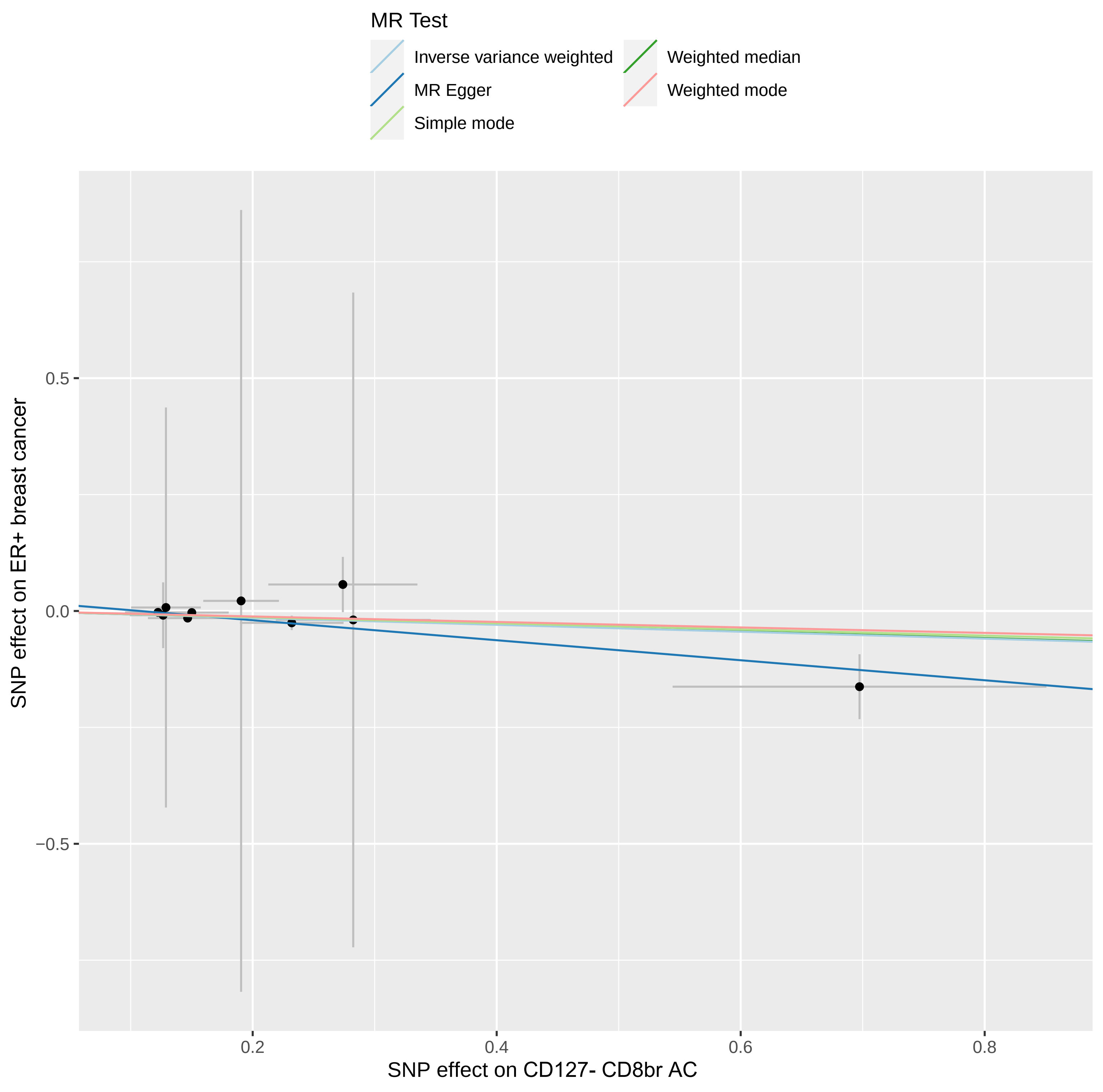
MR Test
Inverse variance weighted
MR Egger
Simple mode
Weighted median
Weighted mode
0.5
0.0
−0.5
0.2
0.4
0.6
0.8
SNP effect on ER+ breast cancer
SNP effect on CD127- CD8br AC

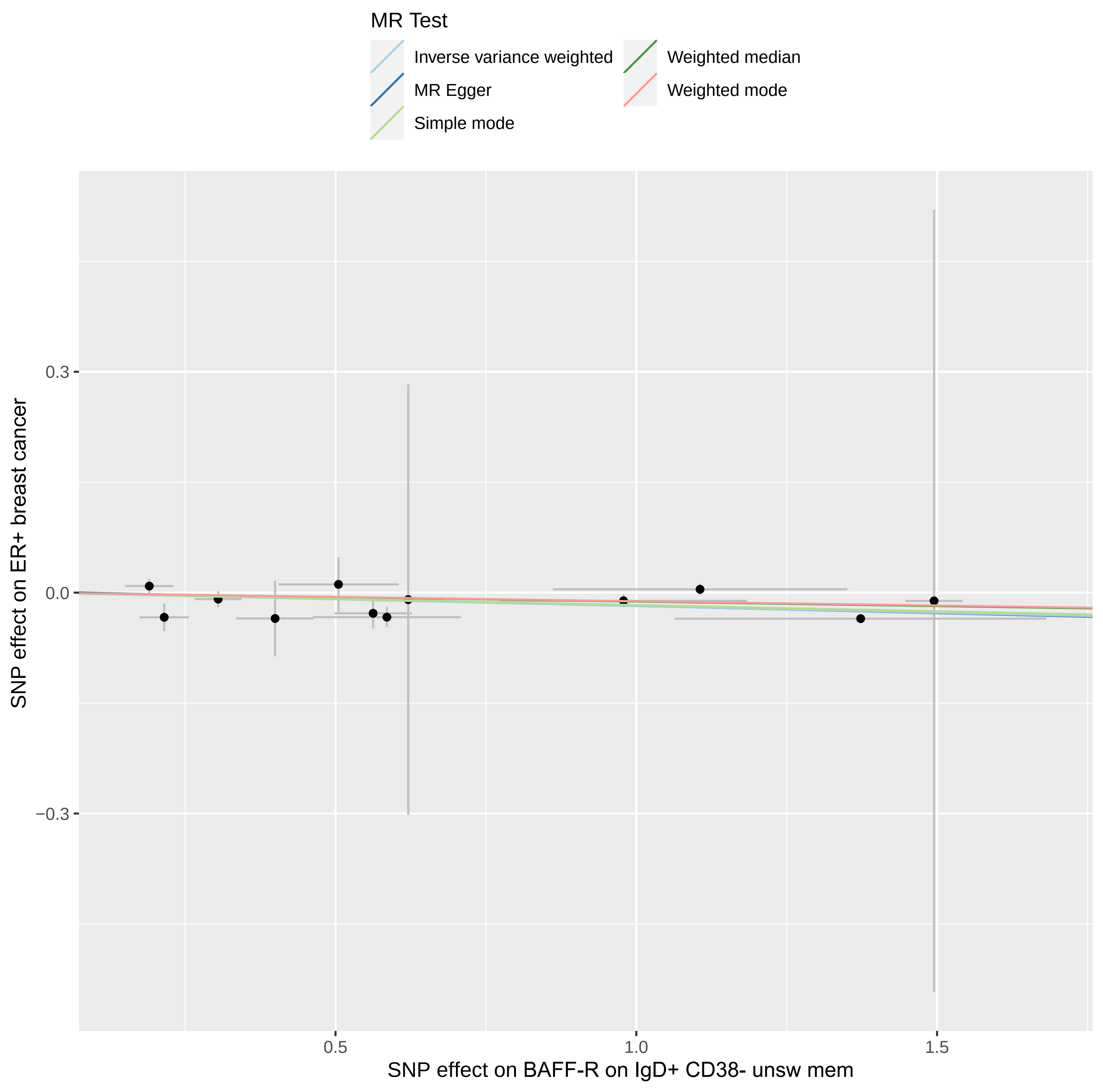
MR Test
Inverse variance weighted
MR Egger
Simple mode
Weighted median
Weighted mode
0.3
0.0
−0.3
0.5
1.0
1.5
SNP effect on ER+ breast cancer
SNP effect on BAFF-R on IgD+ CD38- unsw mem

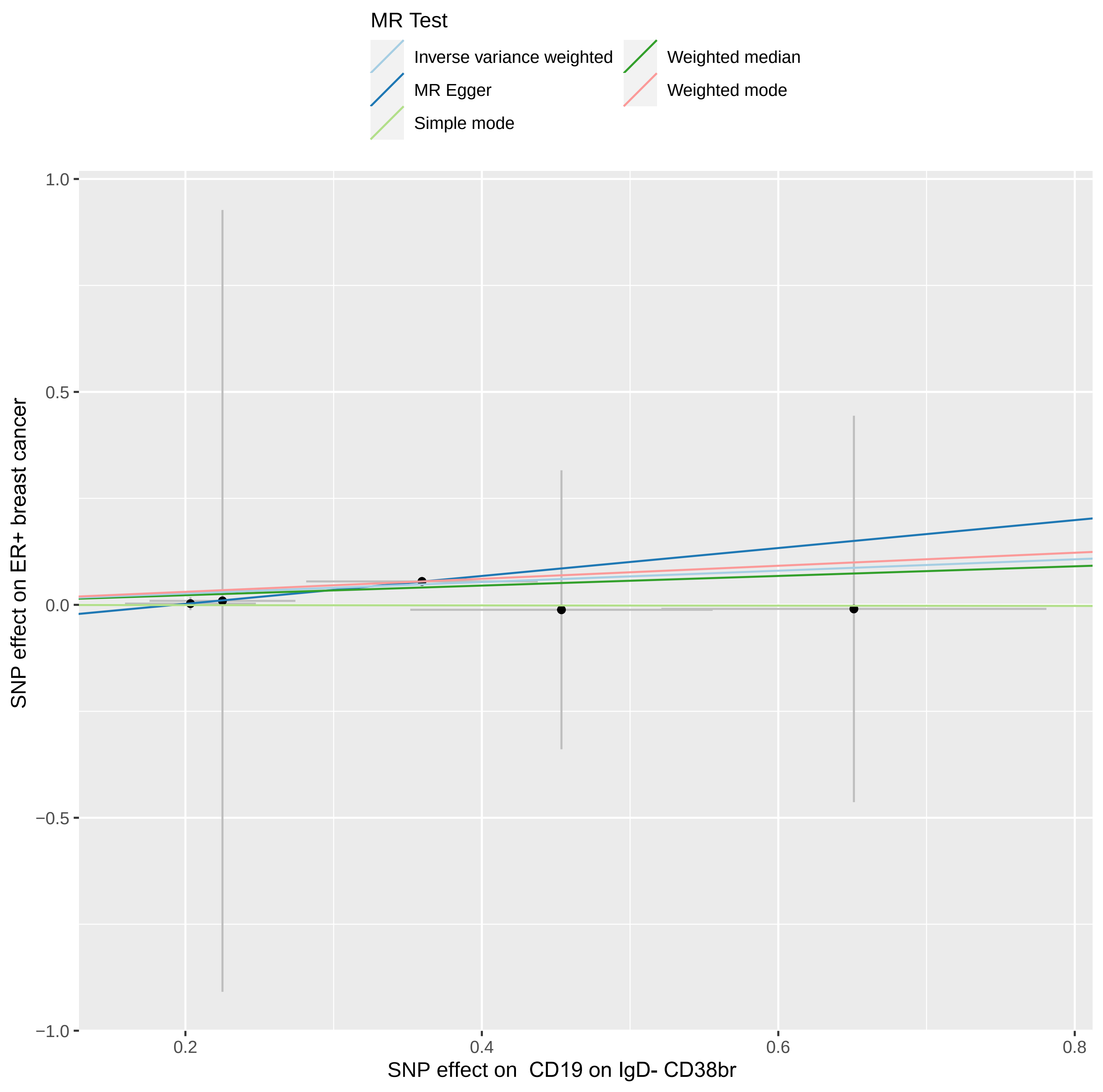

MR Test
Inverse variance weighted
MR Egger
Simple mode
Weighted median
Weighted mode
1.0
0.5
0.0
−0.5
−1.0
0.2
0.4
0.6
0.8
SNP effect on ER+ breast cancer
SNP effect on CD19 on IgD- CD38br

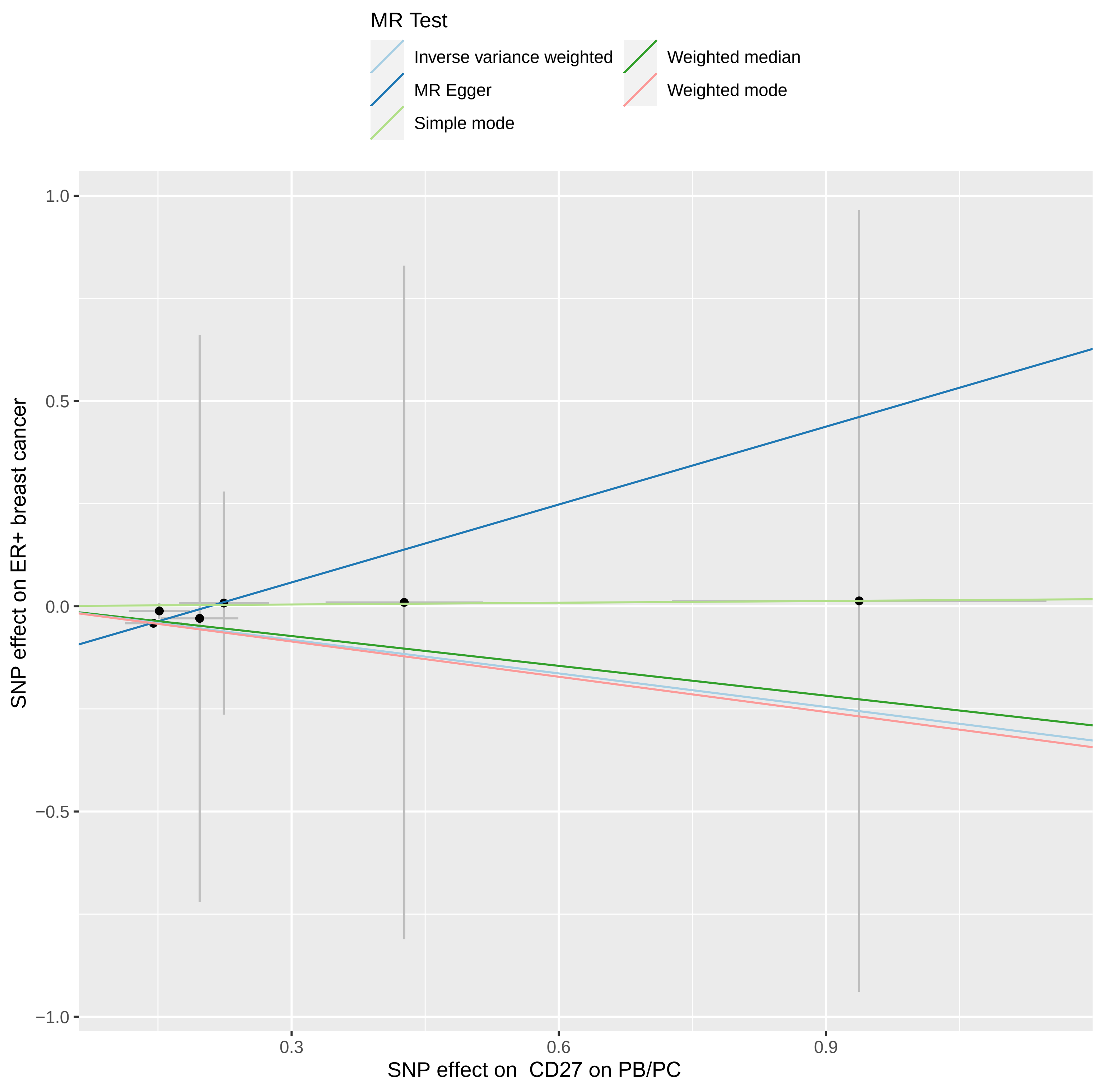

MR Test
Inverse variance weighted
MR Egger
Simple mode
Weighted median
Weighted mode
1.0
0.5
0.0
−0.5
−1.0
0.3
0.6
0.9
SNP effect on ER+ breast cancer
SNP effect on CD27 on PB/PC

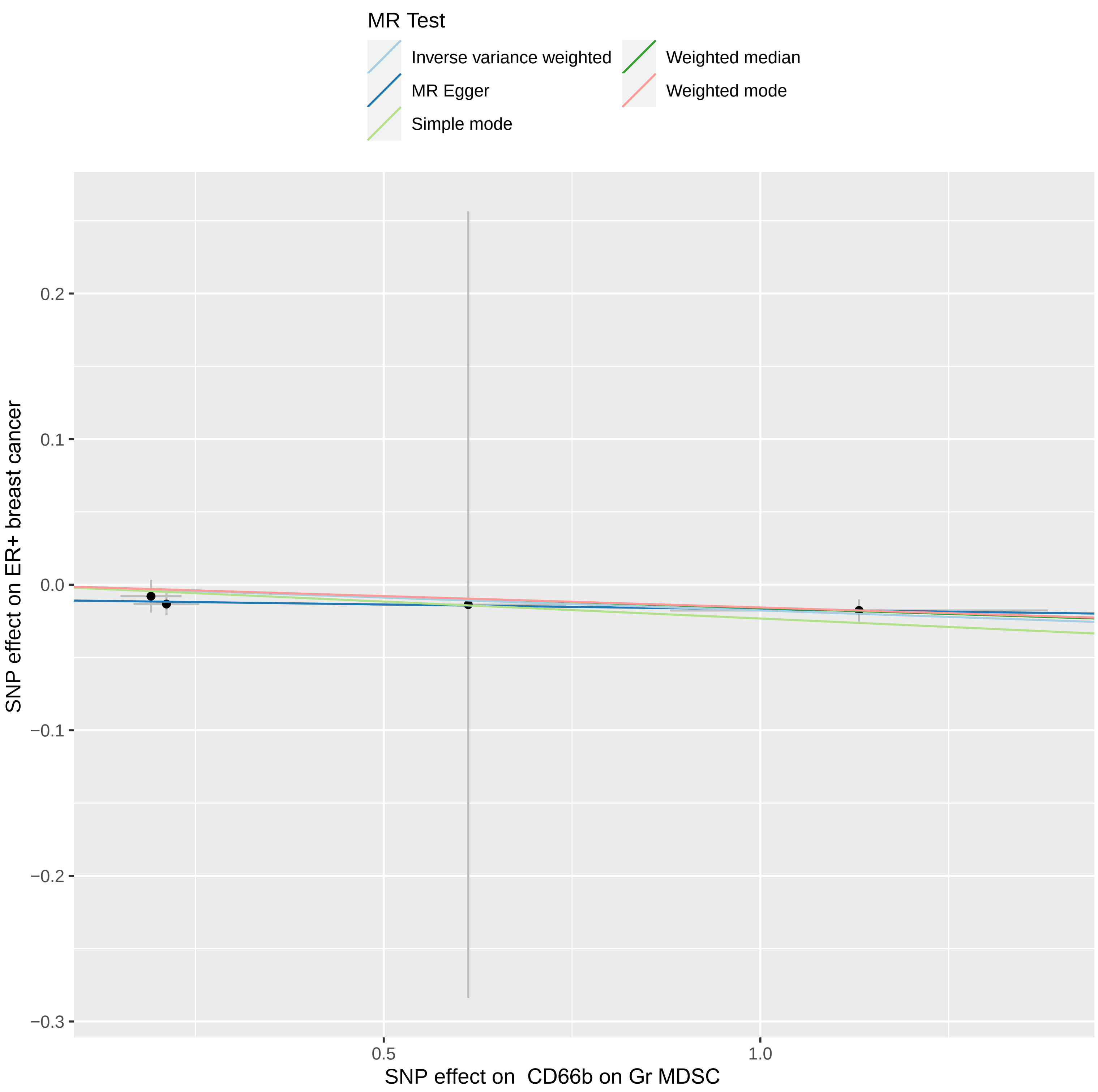

MR Test
Inverse variance weighted
MR Egger
Simple mode
Weighted median
Weighted mode
SNP effect on ER+ breast cancer
0.2
0.1
0.0
−0.1
−0.2
−0.3
0.5
1.0
SNP effect on CD66b on Gr MDSC

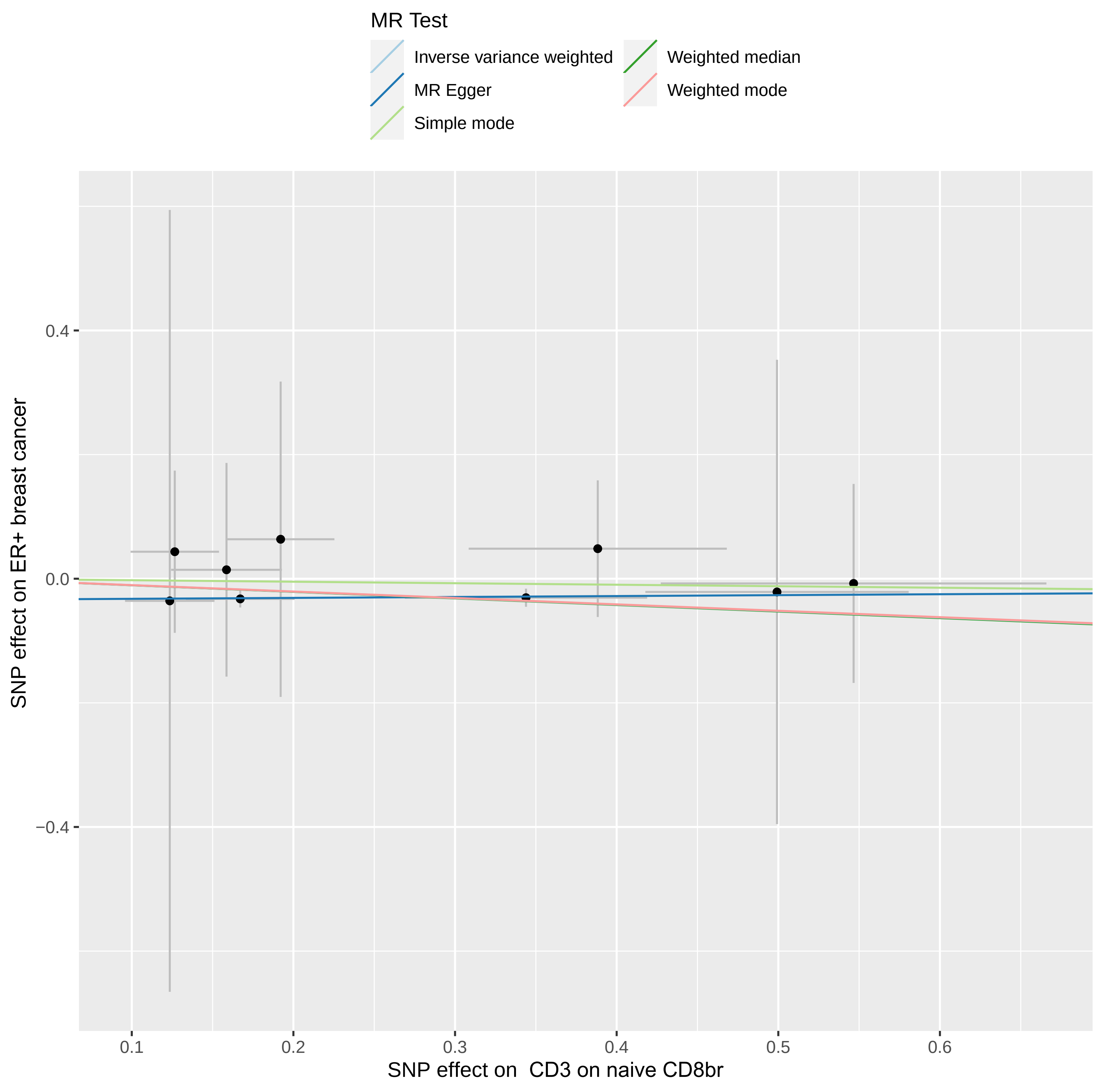
MR Test
Inverse variance weighted
MR Egger
Simple mode
Weighted median
Weighted mode
0.4
0.0
−0.4
0.1
0.2
0.3
0.4
0.5
0.6
SNP effect on ER+ breast cancer
SNP effect on CD3 on naive CD8br

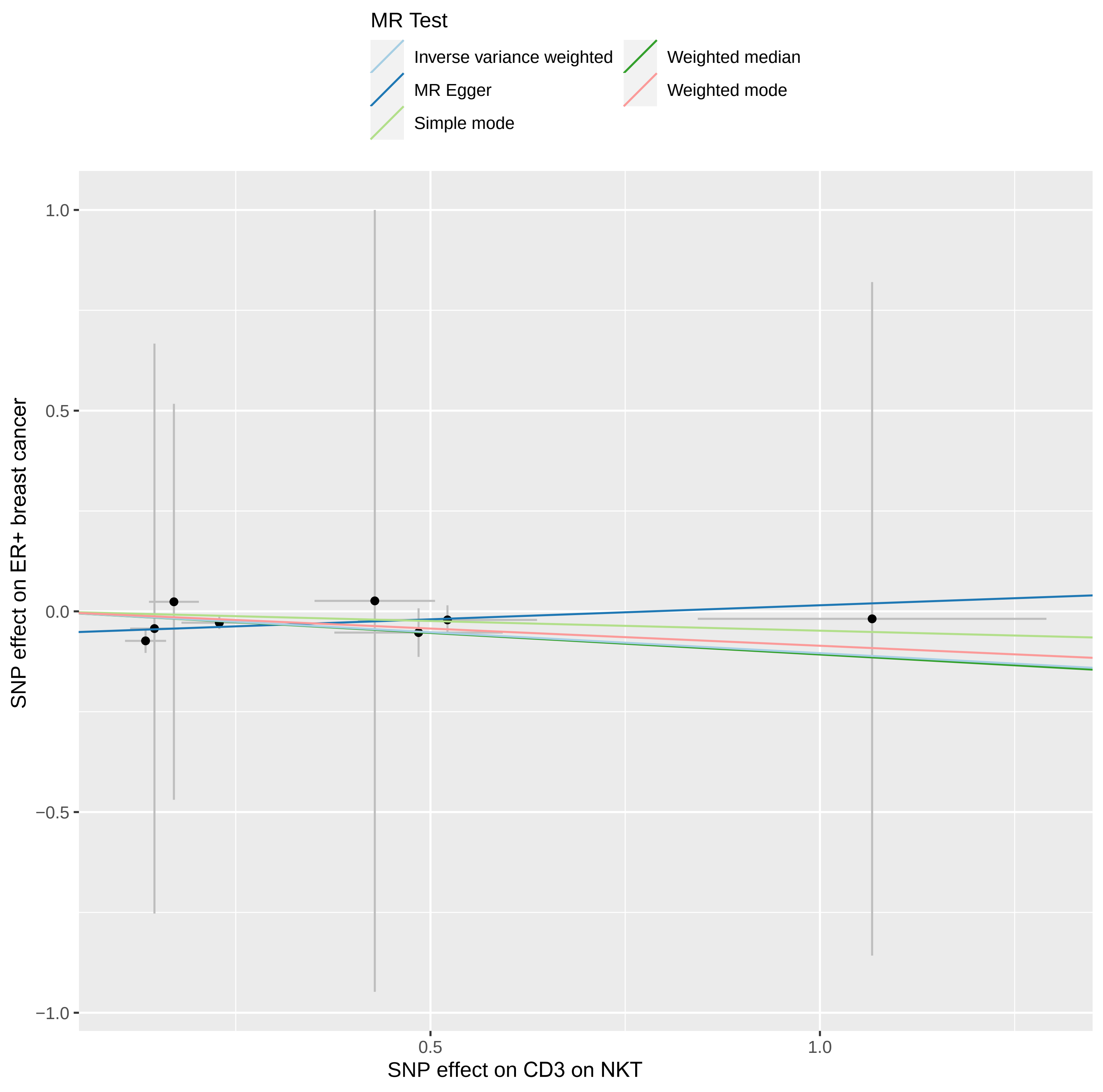

MR Test
Inverse variance weighted
MR Egger
Simple mode
Weighted median
Weighted mode
1.0
0.5
0.0
−0.5
−1.0
0.5
1.0
SNP effect on ER+ breast cancer
SNP effect on CD3 on NKT

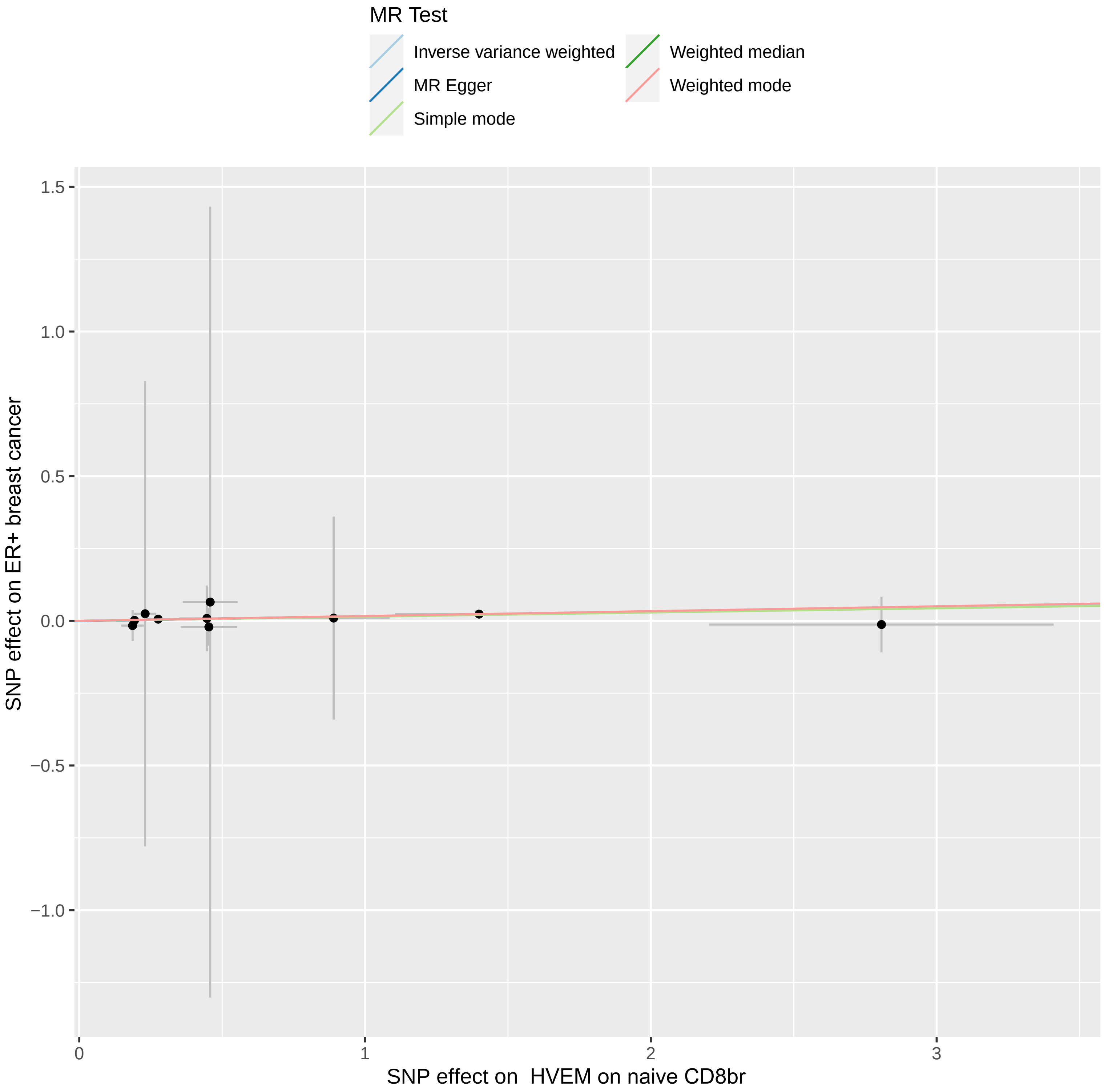
MR Test
Inverse variance weighted
MR Egger
Simple mode
Weighted median
Weighted mode
1.5
1.0
0.5
0.0
−0.5
−1.0
0
1
2
3
SNP effect on ER+ breast cancer
SNP effect on HVEM on naive CD8br

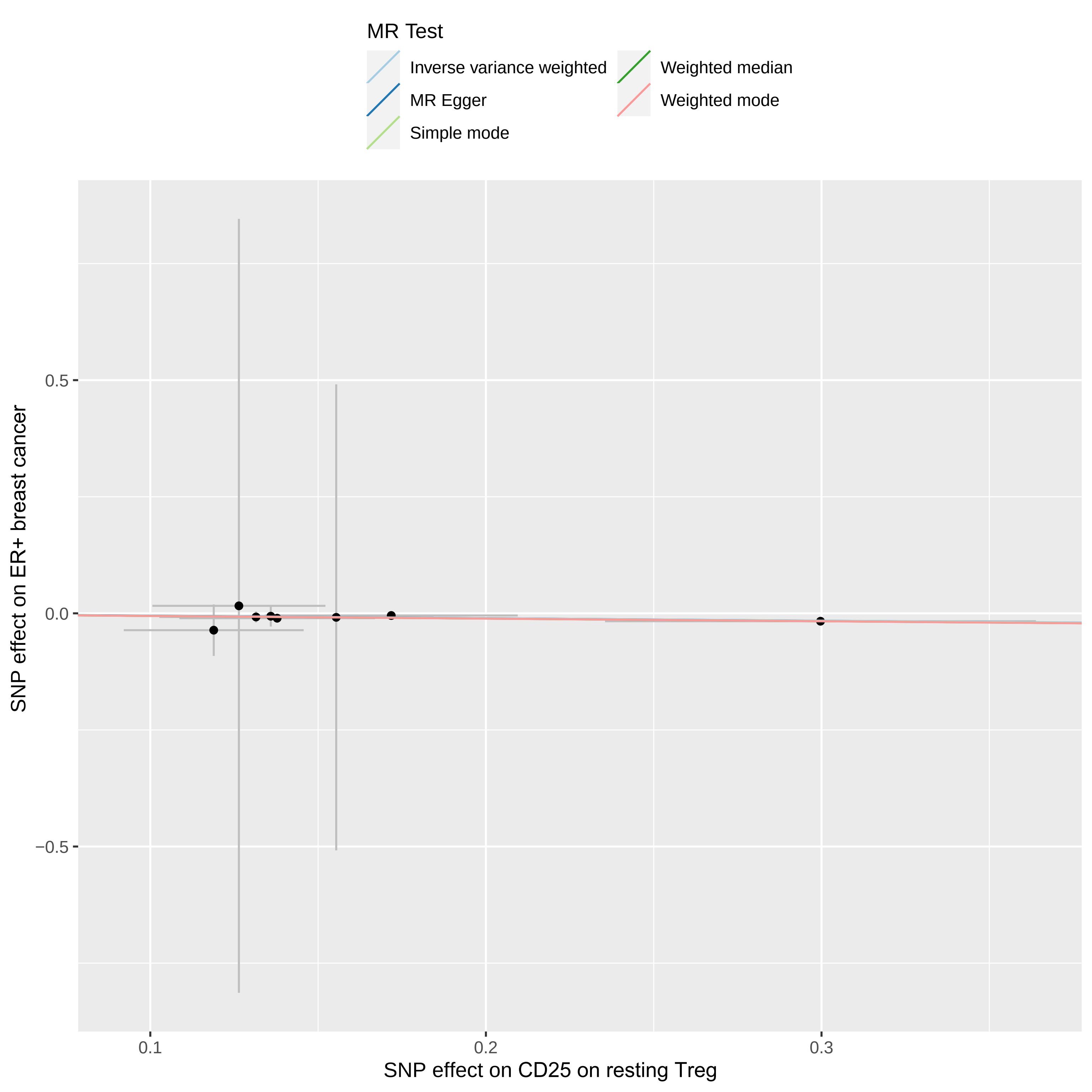
MR Test
Inverse variance weighted
MR Egger
Simple mode
Weighted median
Weighted mode
0.5
0.0
−0.5
0.1
0.2
0.3
SNP effect on ER+ breast cancer
SNP effect on CD25 on resting Treg

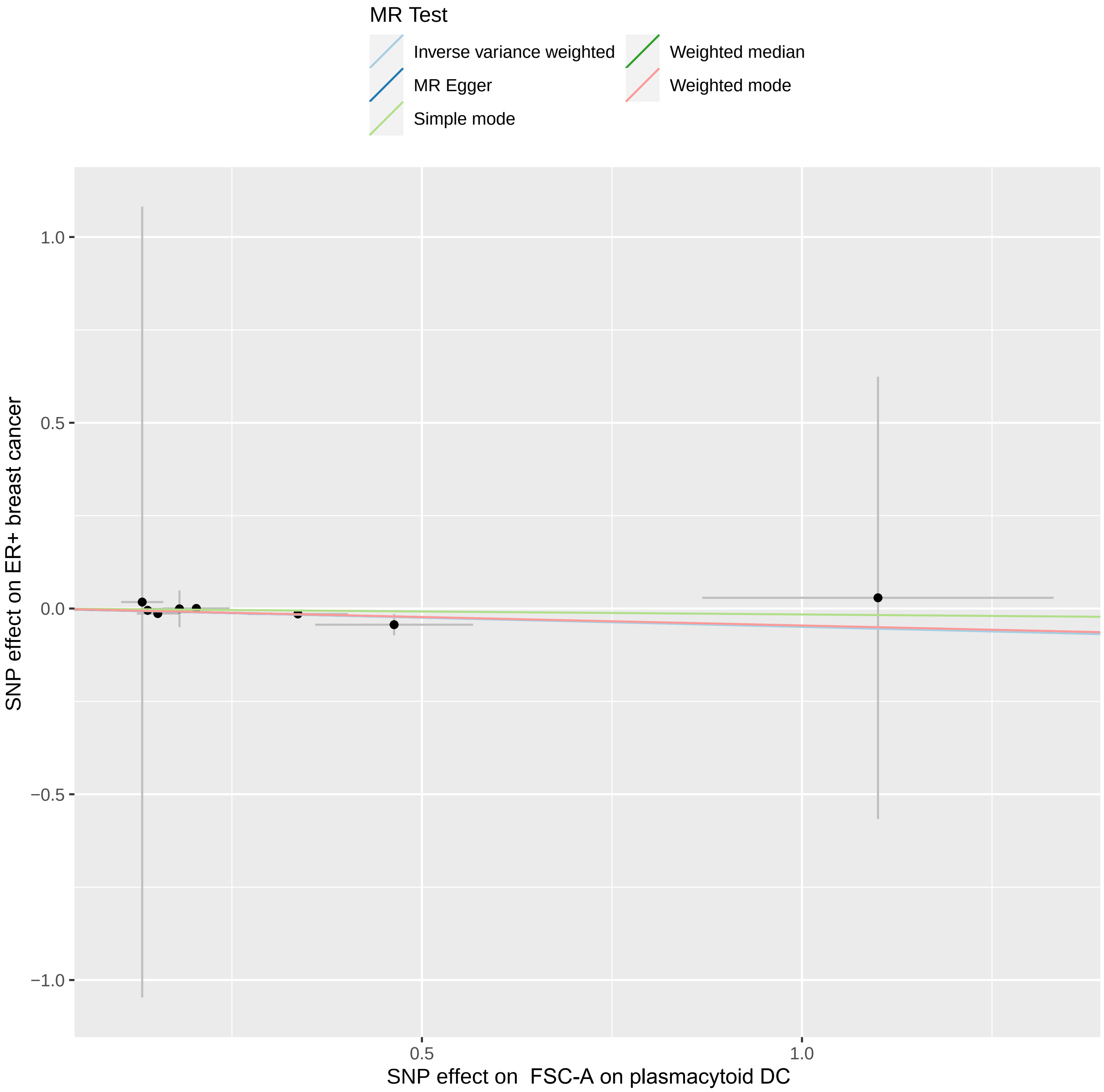
MR Test
Inverse variance weighted
MR Egger
Simple mode
Weighted median
Weighted mode
1.0
0.5
0.0
−0.5
−1.0
SNP effect on ER+ breast cancer
0.5
1.0
SNP effect on FSC-A on plasmacytoid DC

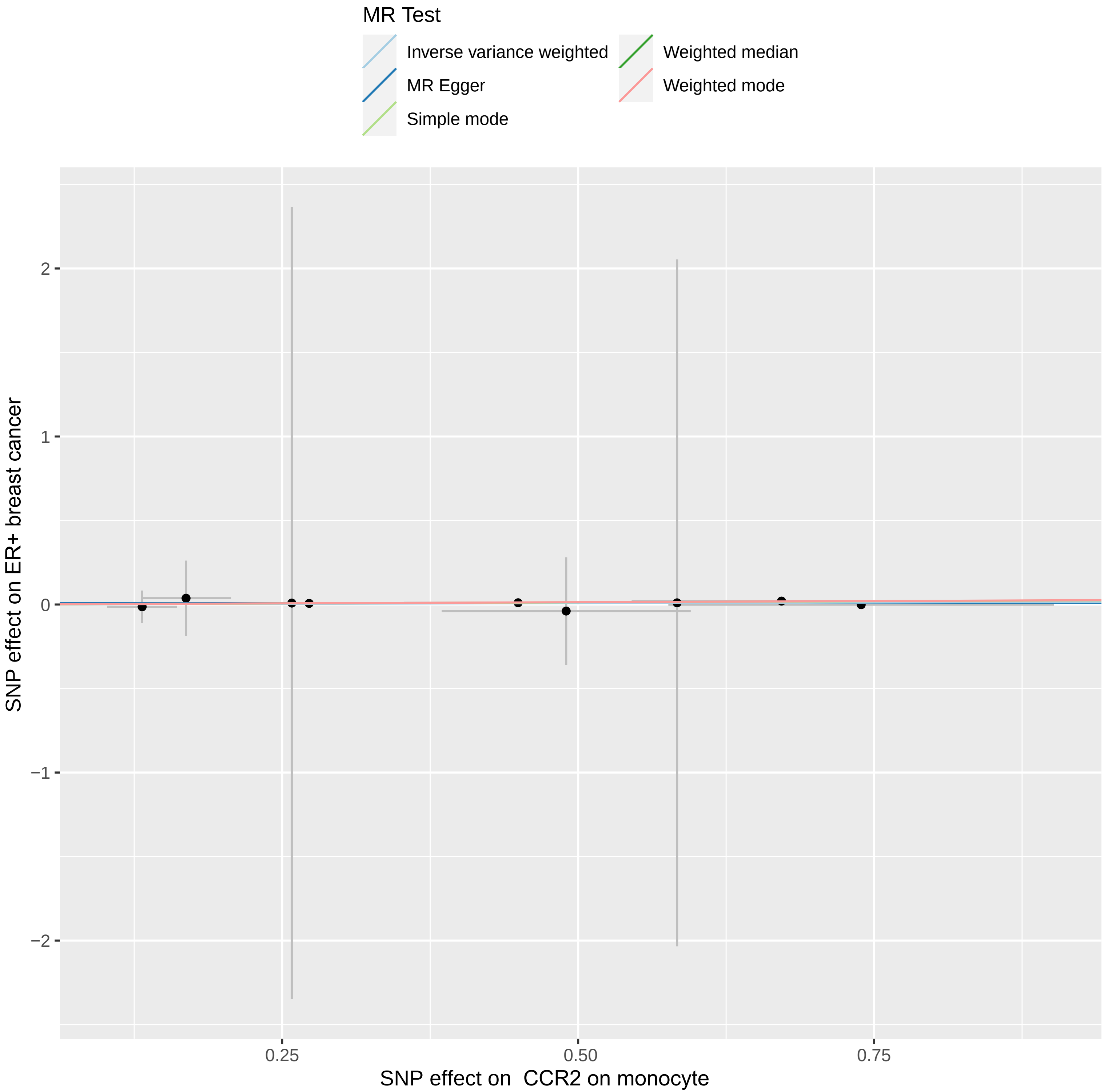
MR Test
Inverse variance weighted
MR Egger
Simple mode
Weighted median
Weighted mode
SNP effect on ER+ breast cancer
2
1
0
−1
−2
0.25
0.50
0.75
SNP effect on CCR2 on monocyte

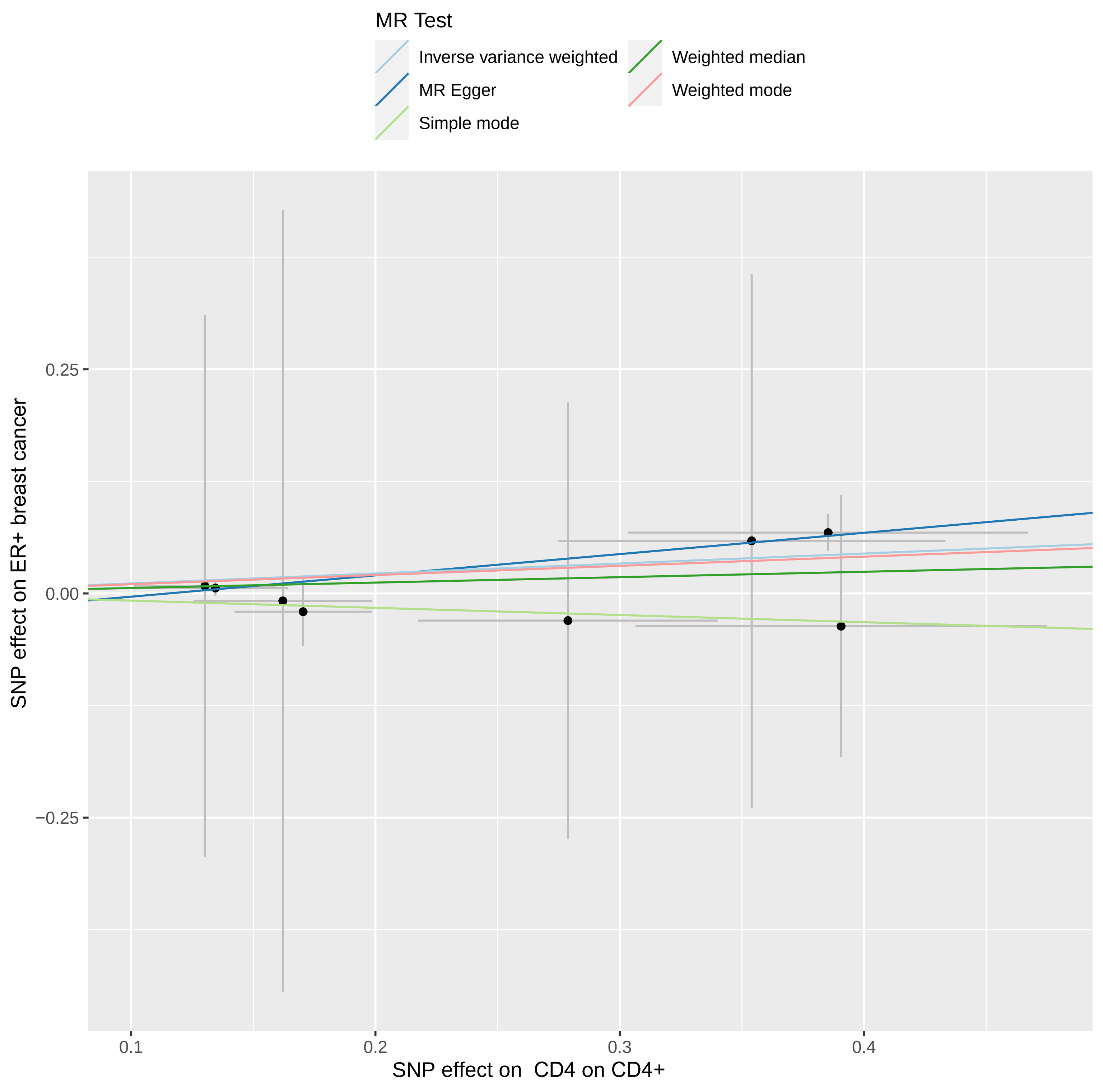

MR Test
Inverse variance weighted
MR Egger
Simple mode
Weighted median
Weighted mode
0.25
0.00
−0.25
0.1
0.2
0.3
0.4
SNP effect on ER+ breast cancer
SNP effect on CD4 on CD4+

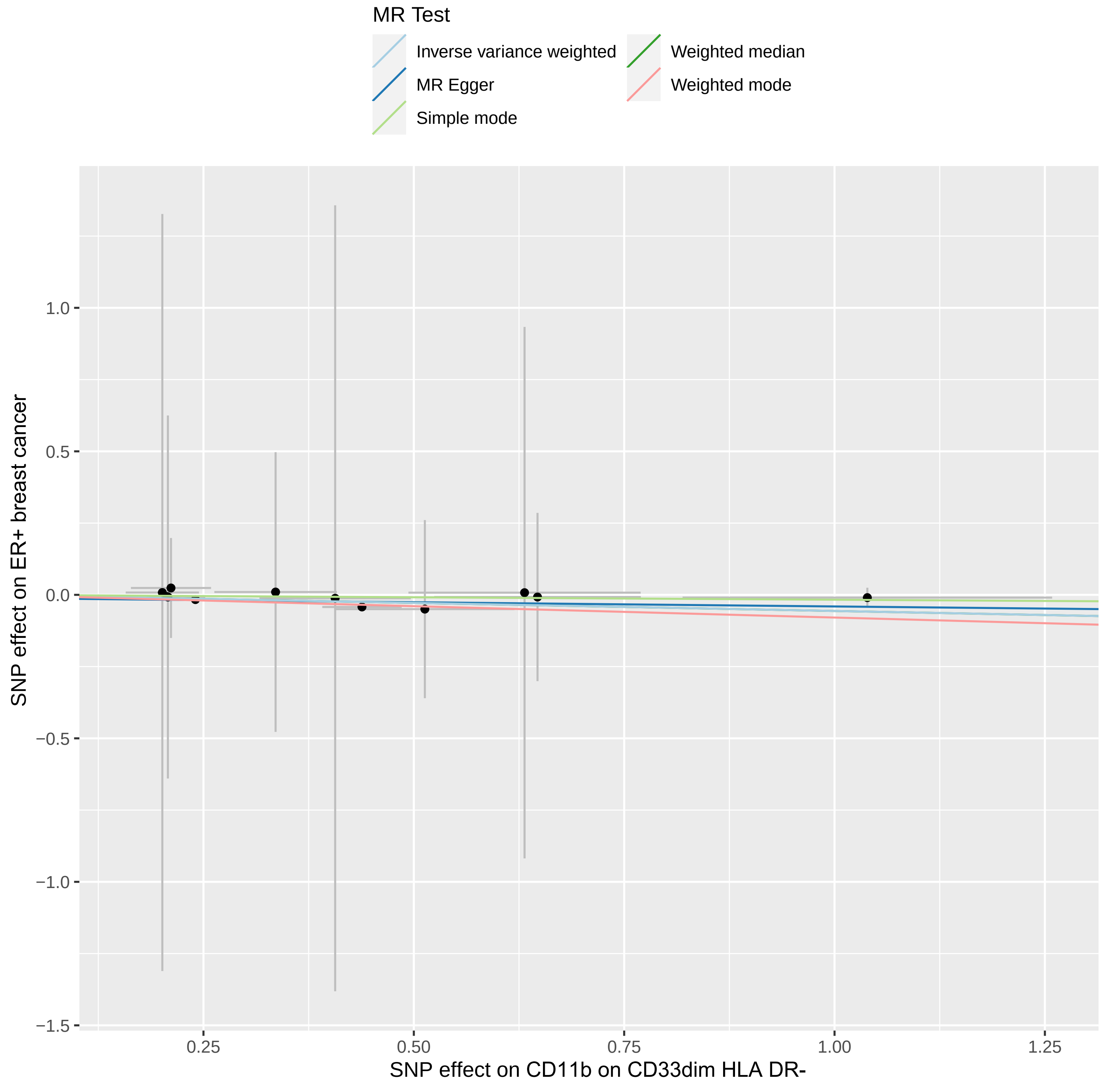
MR Test
Inverse variance weighted
MR Egger
Simple mode
Weighted median
Weighted mode
SNP effect on ER+ breast cancer
1.0
0.5
0.0
−0.5
−1.0
−1.5
0.25
0.50
0.75
1.00
1.25
SNP effect on CD11b on CD33dim HLA DR-

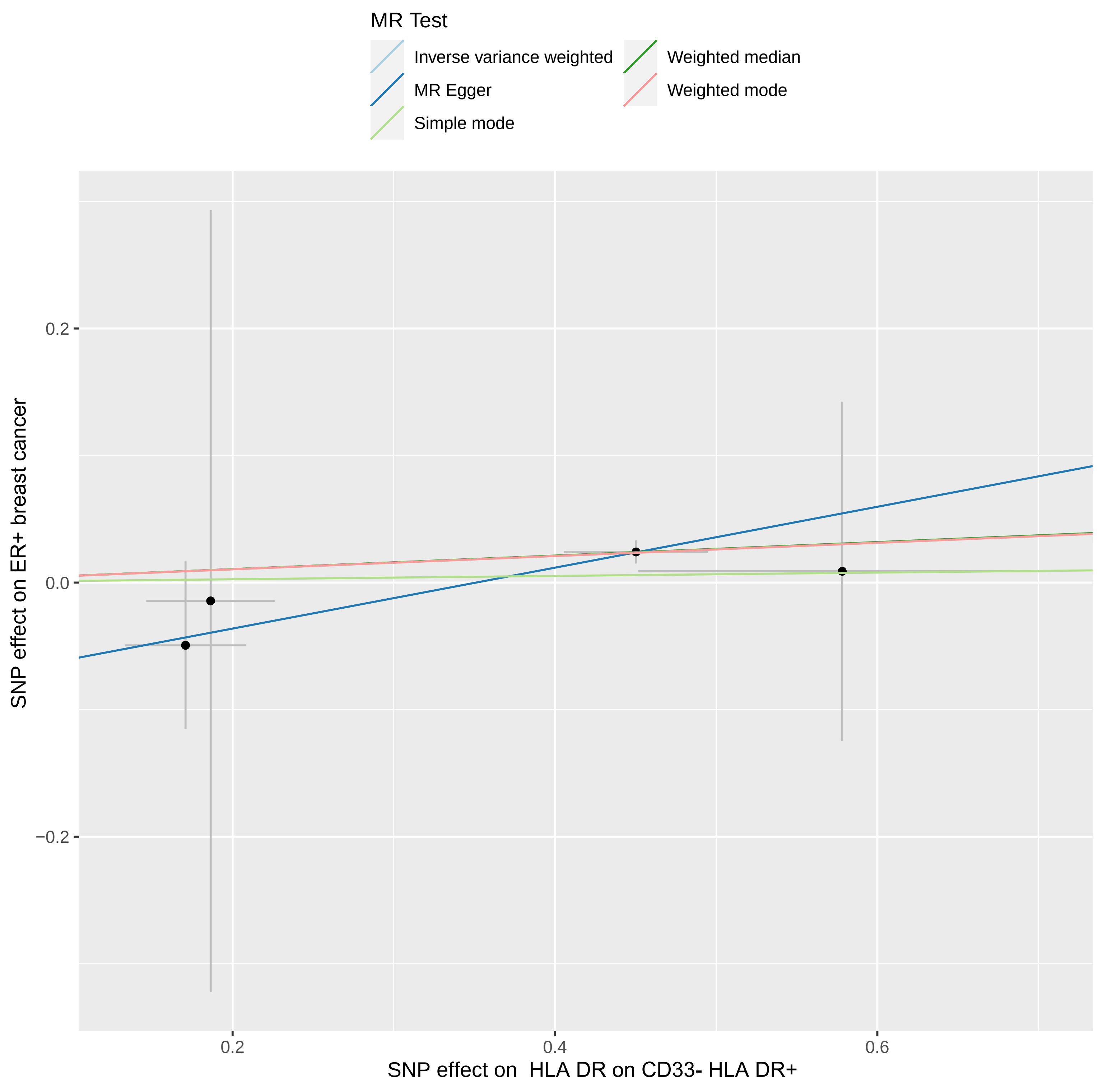

MR Test
Inverse variance weighted
MR Egger
Simple mode
Weighted median
Weighted mode
0.2
0.0
−0.2
0.2
0.4
0.6
SNP effect on ER+ breast cancer
SNP effect on HLA DR on CD33- HLA DR+

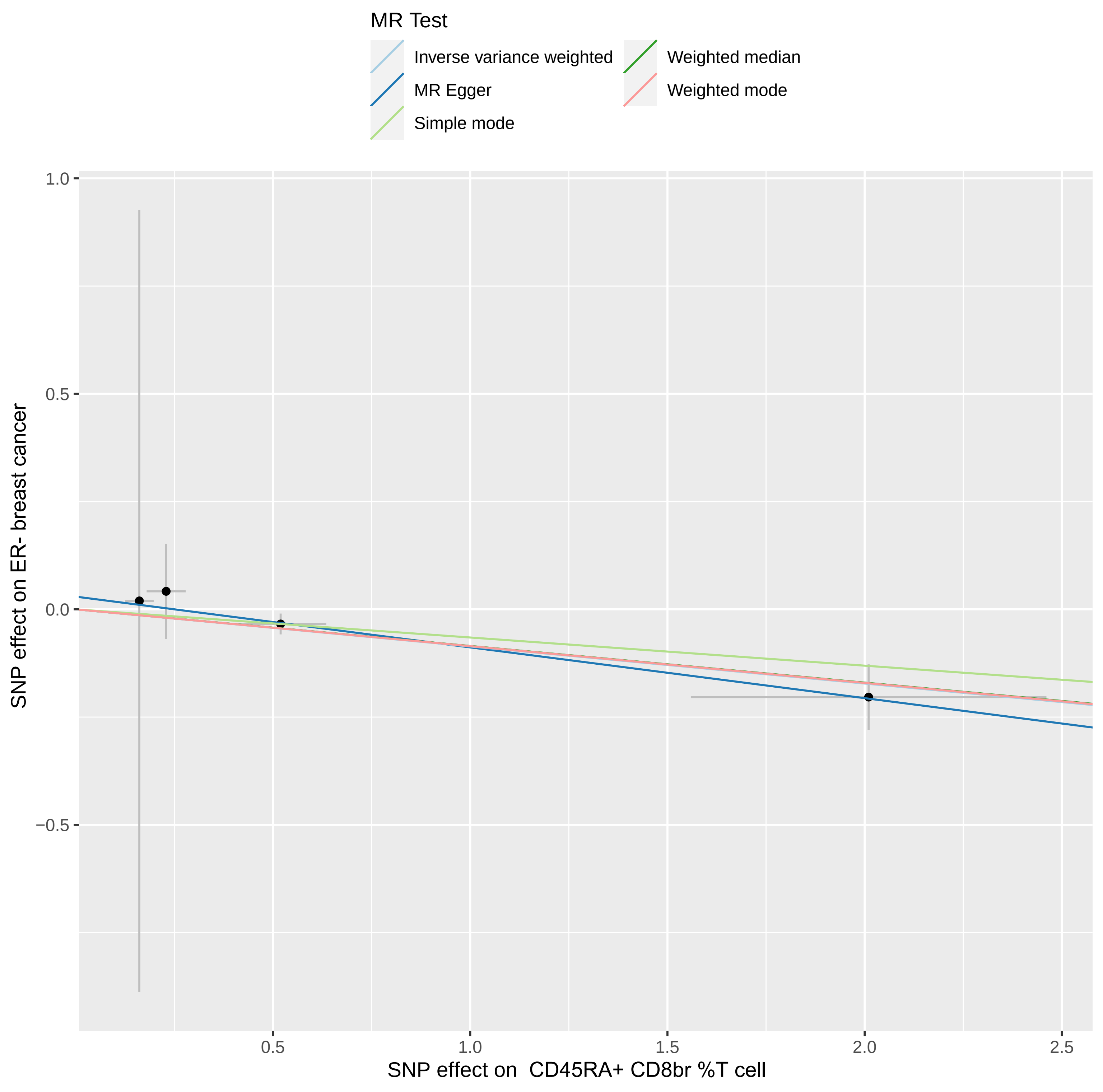

MR Test
Inverse variance weighted
MR Egger
Simple mode
Weighted median
Weighted mode
1.0
0.5
0.0
−0.5
SNP effect on ER- breast cancer
0.5
1.0
1.5
2.0
2.5
SNP effect on CD45RA+ CD8br %T cell

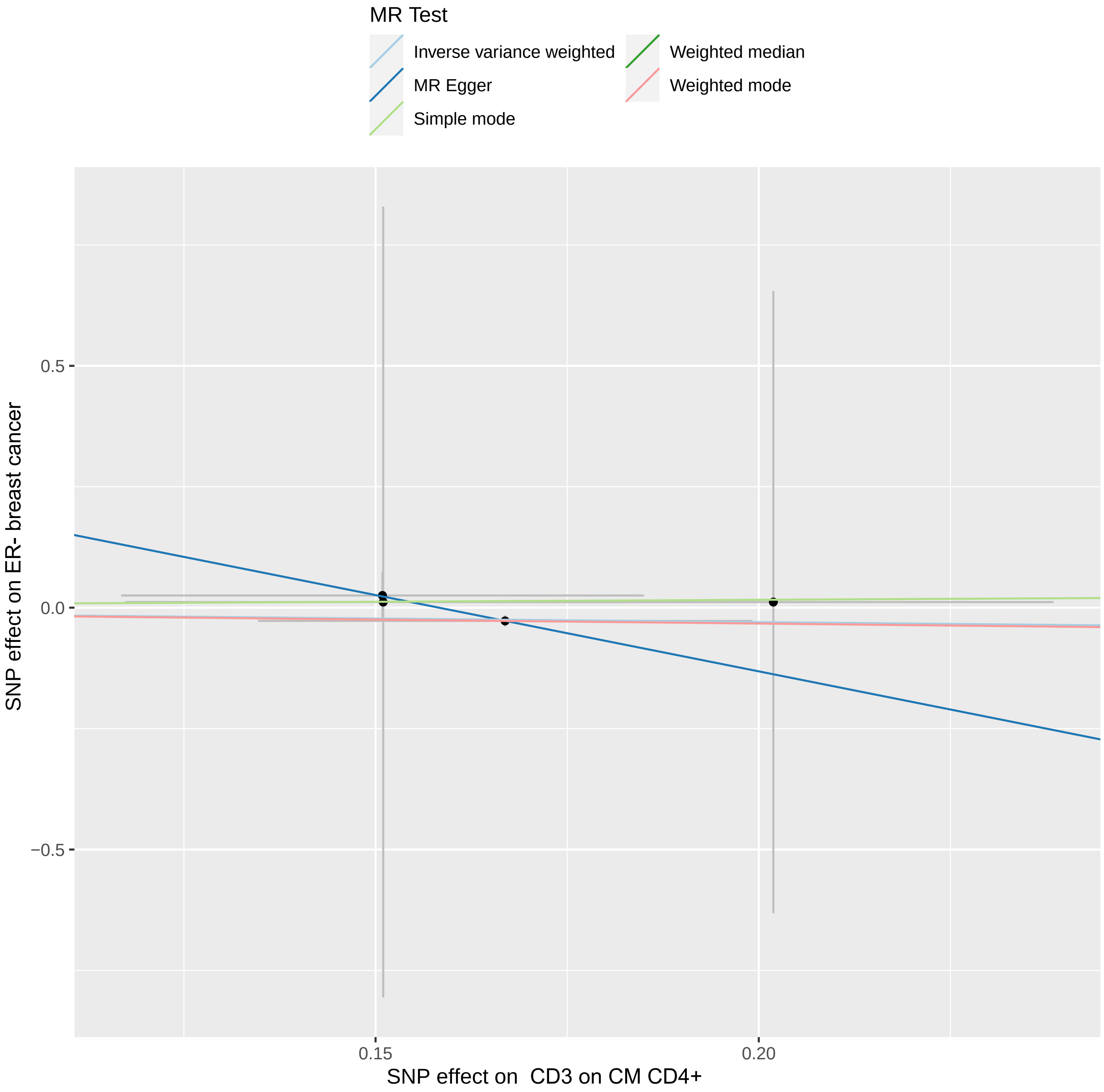
MR Test
Inverse variance weighted
MR Egger
Simple mode
Weighted median
Weighted mode
0.5
0.0
−0.5
0.15
0.20
SNP effect on ER- breast cancer
SNP effect on CD3 on CM CD4+

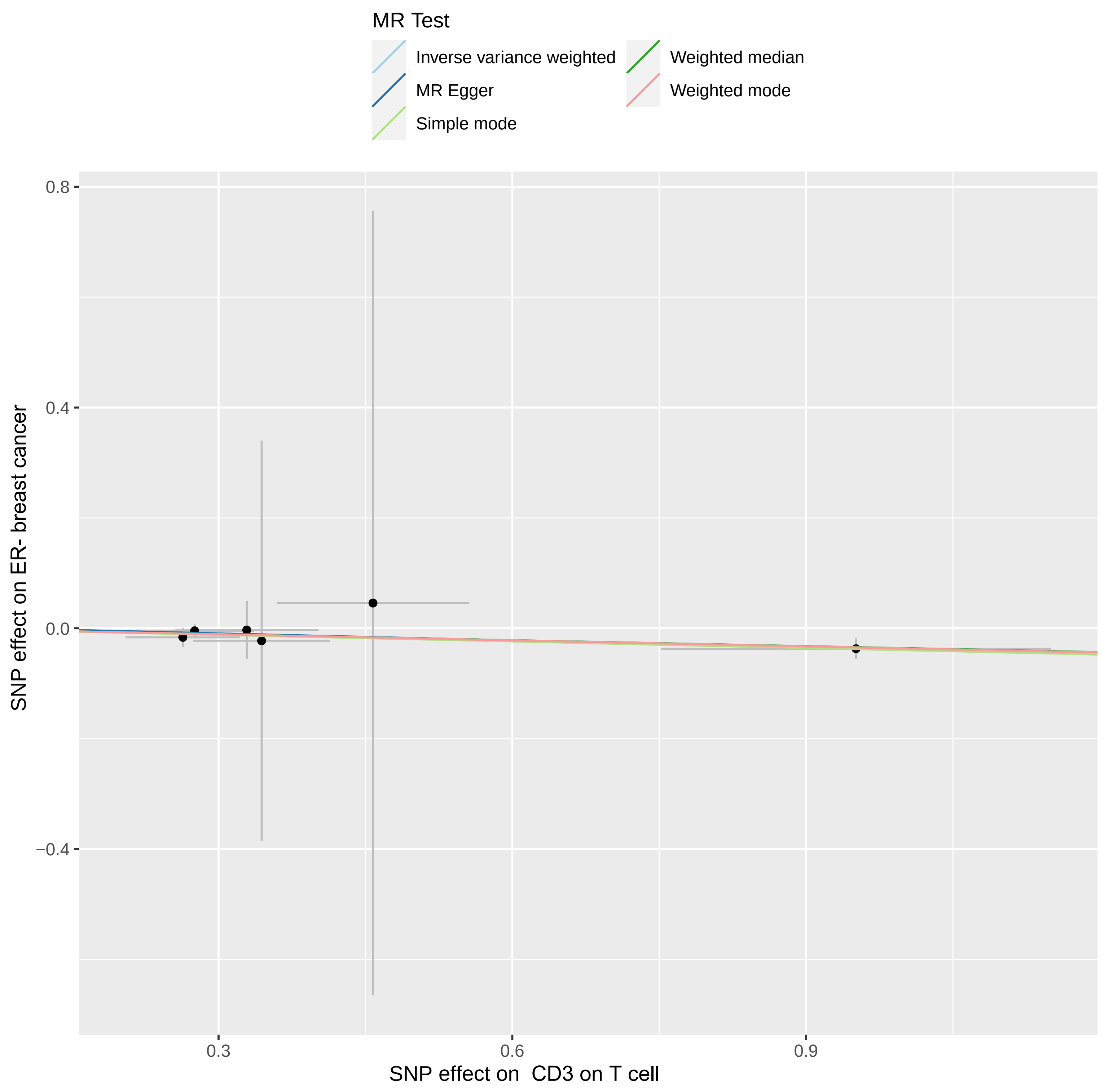

MR Test
Inverse variance weighted
MR Egger
Simple mode
Weighted median
Weighted mode
0.8
0.4
0.0
−0.4
0.3
0.6
0.9
SNP effect on ER- breast cancer
SNP effect on CD3 on T cell

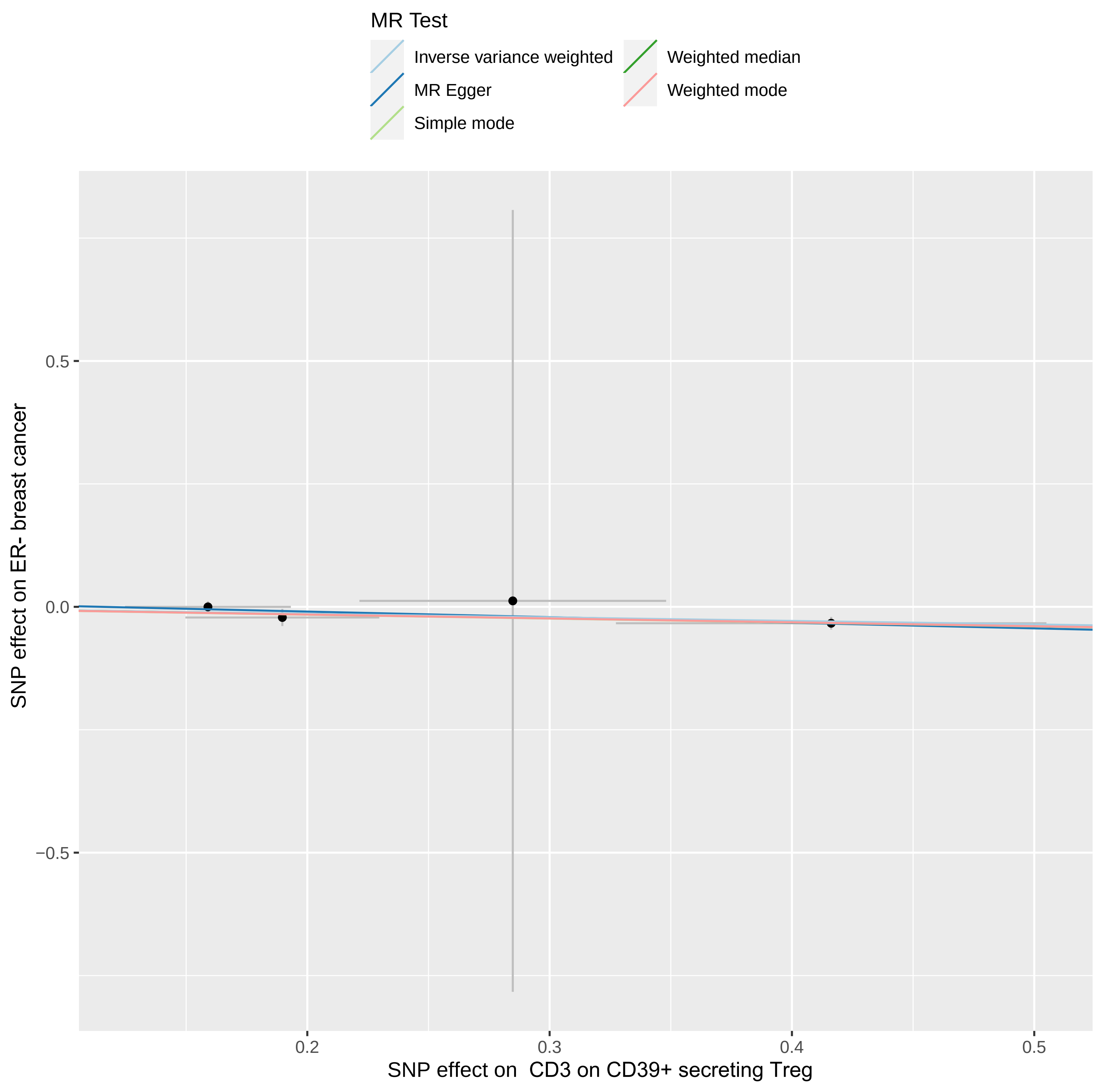

MR Test
Inverse variance weighted
MR Egger
Simple mode
Weighted median
Weighted mode
0.5
0.0
−0.5
0.2
0.3
0.4
0.5
SNP effect on ER- breast cancer
SNP effect on CD3 on CD39+ secreting Treg

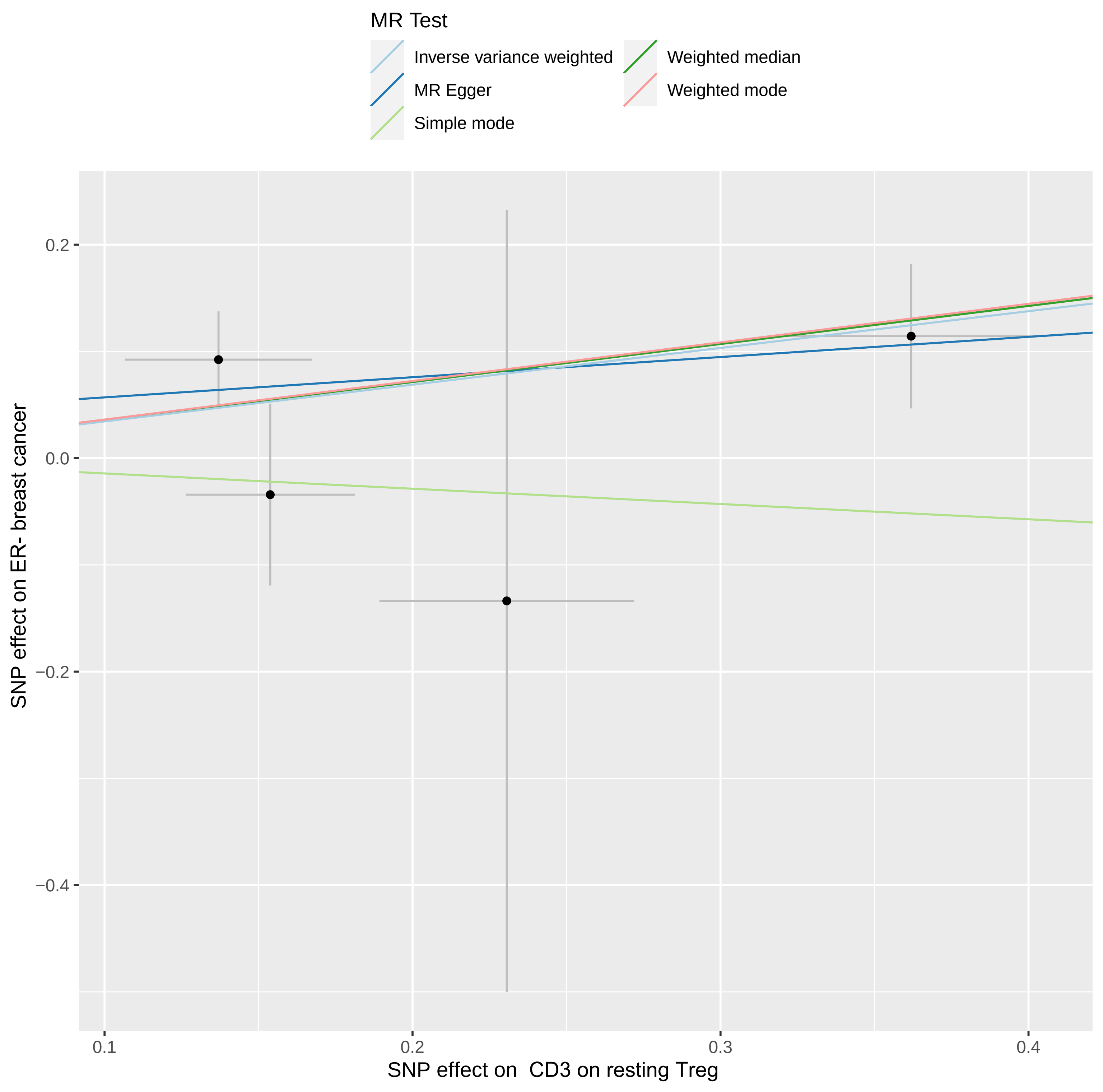

MR Test
Inverse variance weighted
MR Egger
Simple mode
Weighted median
Weighted mode
SNP effect on ER- breast cancer
0.2
0.0
−0.2
−0.4
0.1
0.2
0.3
0.4
SNP effect on CD3 on resting Treg

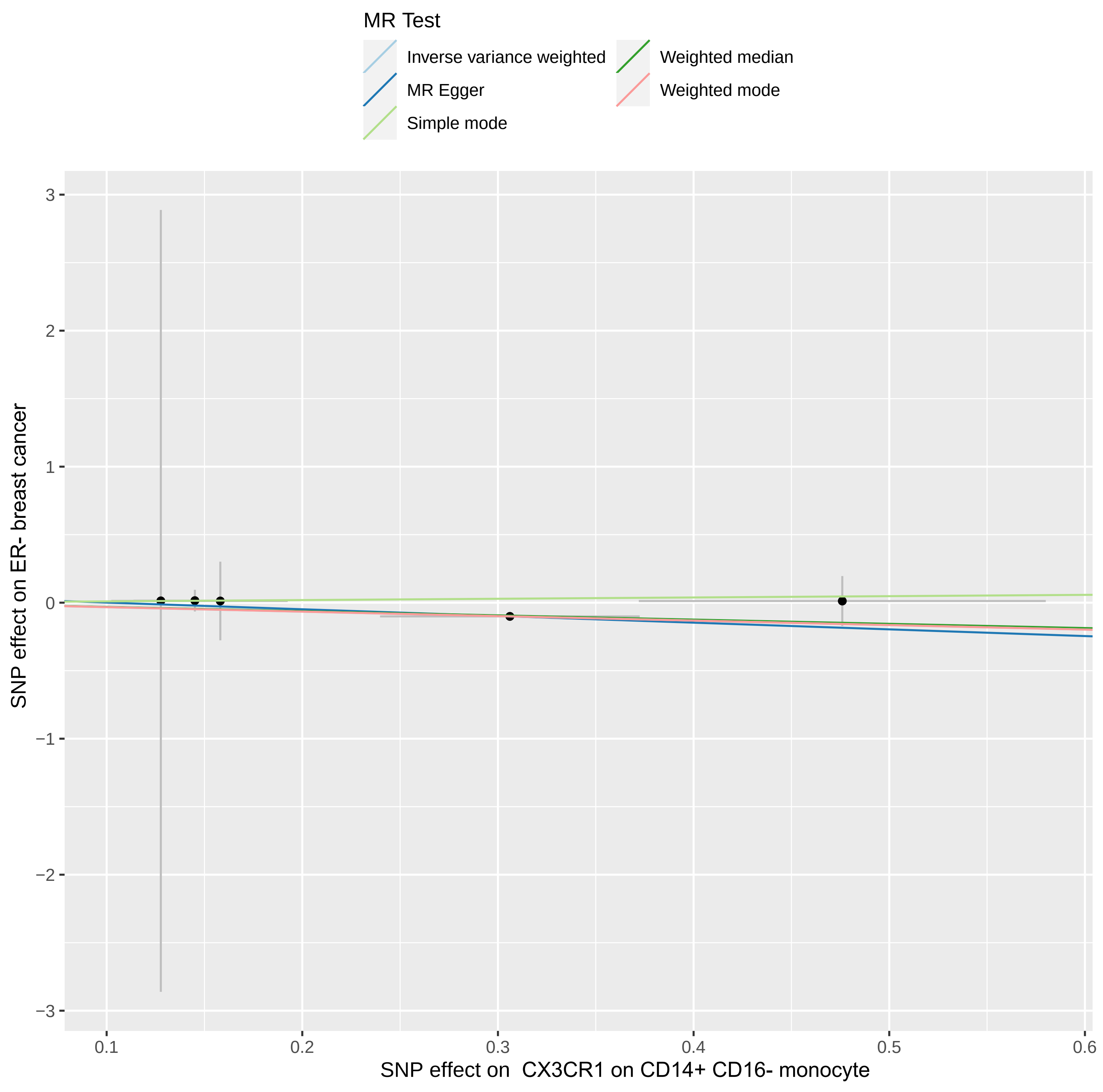

MR Test
Inverse variance weighted
MR Egger
Simple mode
Weighted median
Weighted mode
3
2
1
0
−1
−2
−3
SNP effect on ER- breast cancer
0.1
0.2
0.3
0.4
0.5
0.6
SNP effect on CX3CR1 on CD14+ CD16- monocyte

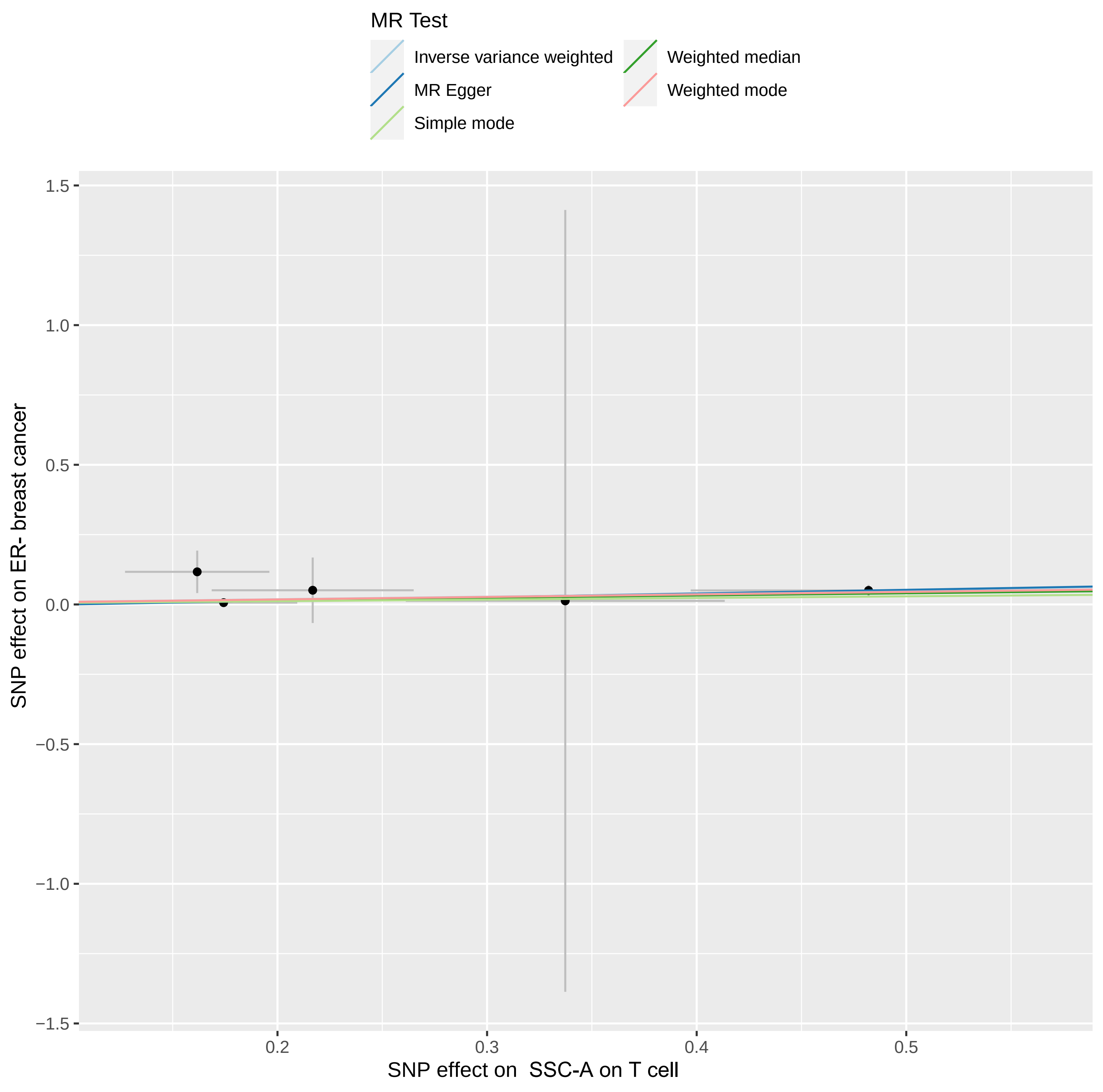

MR Test
Inverse variance weighted
MR Egger
Simple mode
Weighted median
Weighted mode
1.5
1.0
0.5
0.0
−0.5
−1.0
−1.5
SNP effect on ER- breast cancer
0.2
0.3
0.4
0.5
SNP effect on SSC-A on T cell
