# Supplementary material for: A Mendelian analysis of the relationships between immune cells and breast cancer
Source: Front Oncol. 2024 Jan 24;14:1341292. doi: 10.3389/fonc.2024.1341292 (PMC10847340; doi:10.3389/fonc.2024.1341292)
Supplement: Supplementary Figure 3 — Funnel plot to assess heterogeneity between immune cells on overall breast cancer, ER+ breast cancer and ER- breast cancer. [file DataSheet_3.pdf]

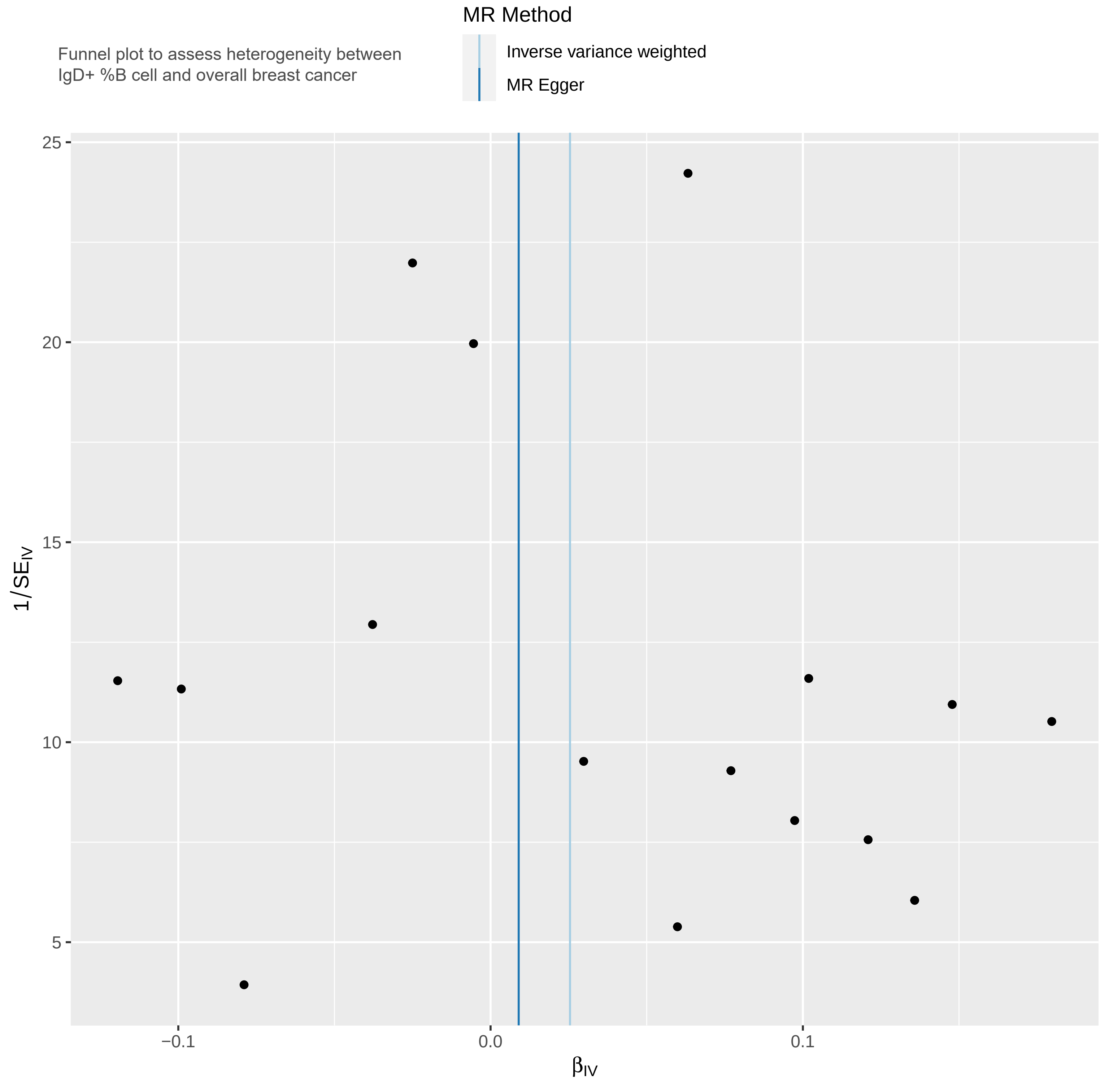

MR Method
Inverse variance weighted
MR Egger
Funnel plot to assess heterogeneity between
IgD+ %B cell and overall breast cancer
25
20
15
10
5
1/SE_IV
−0.1
0.0
0.1
β_IV

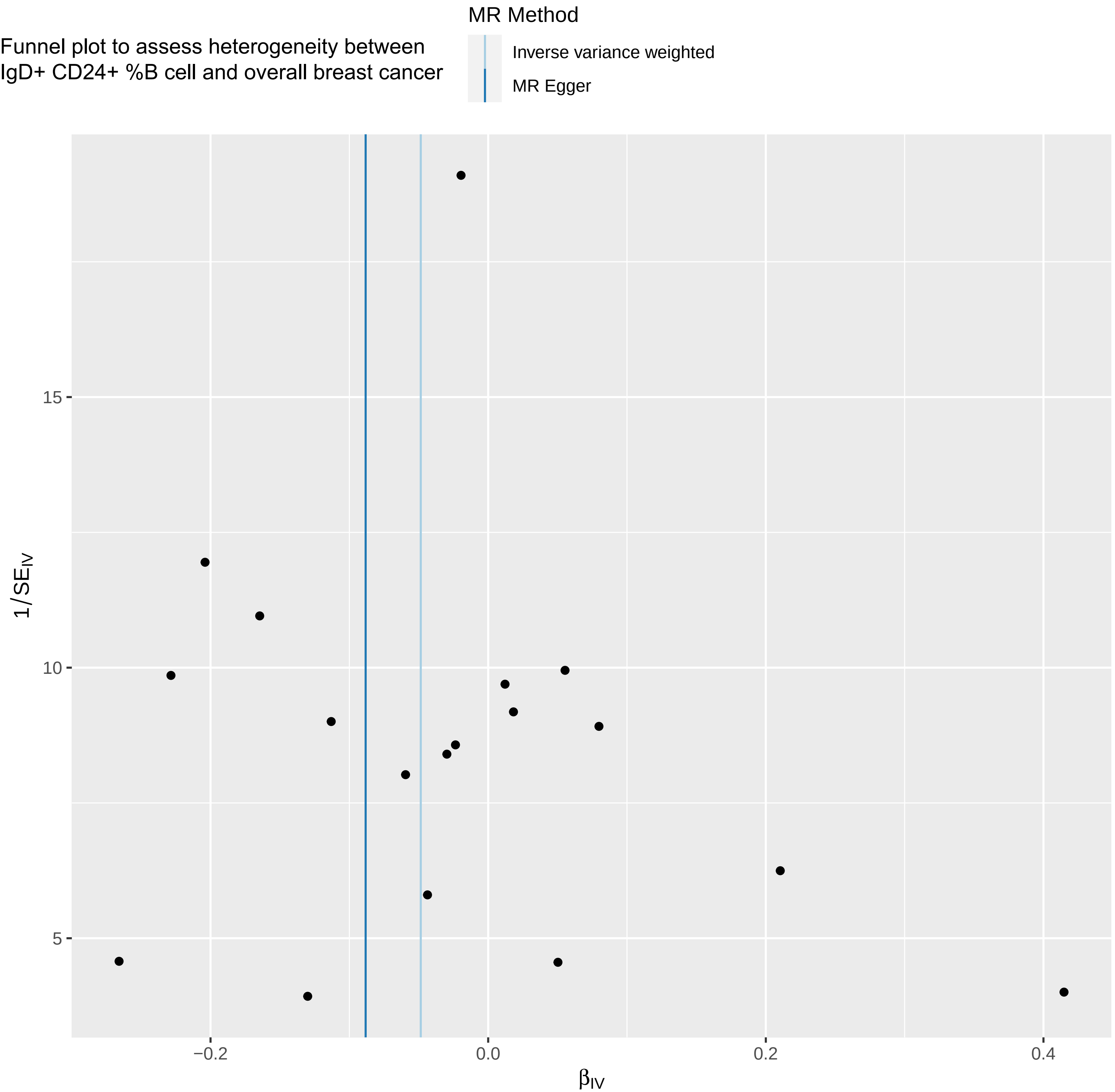

Funnel plot to assess heterogeneity between
IgD+ CD24+ %B cell and overall breast cancer
MR Method
Inverse variance weighted
MR Egger
15
10
5
1/SE$_{IV}$
−0.2
0.0
0.2
0.4
β$_{IV}$

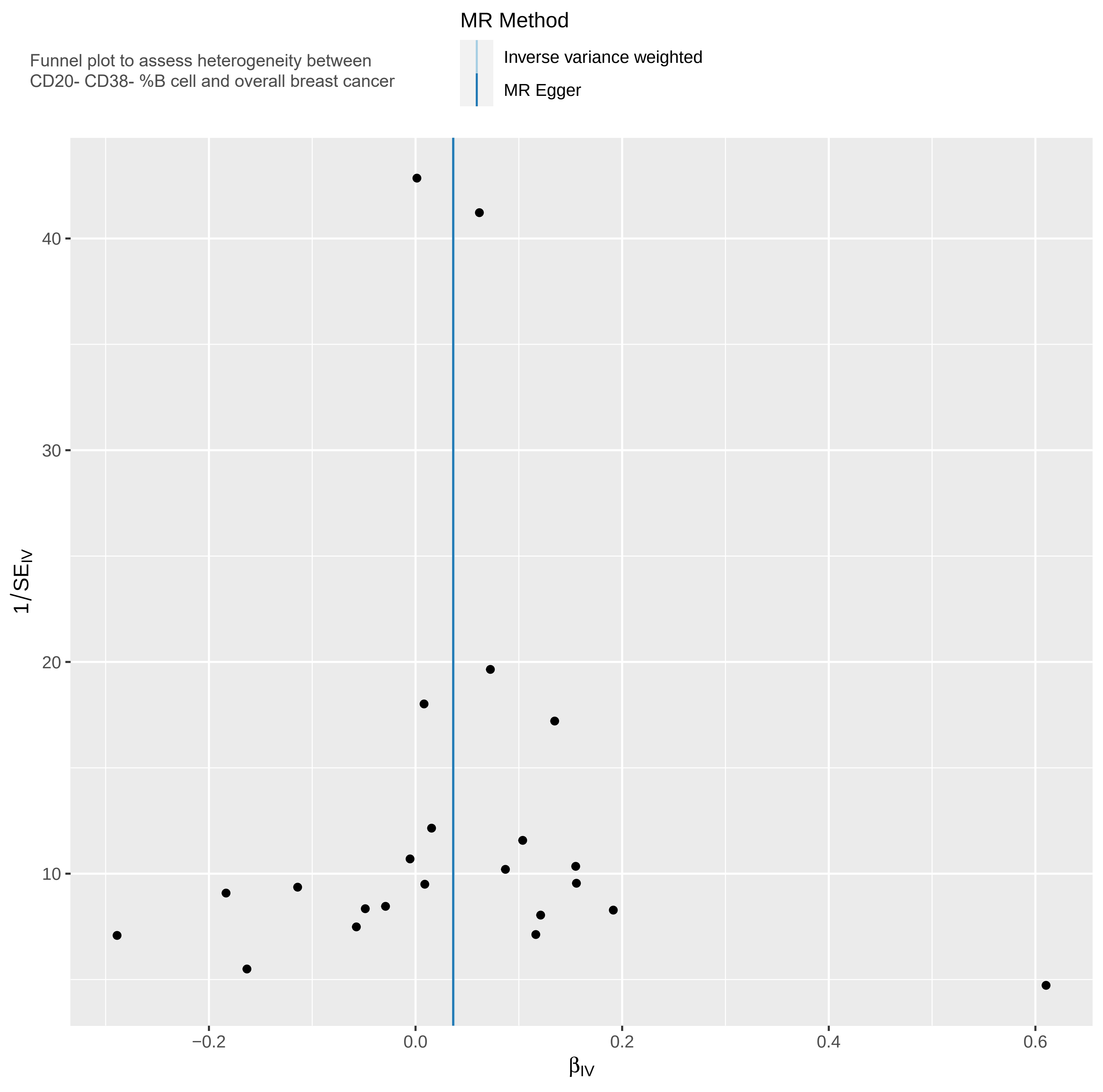
Funnel plot to assess heterogeneity between
CD20- CD38- %B cell and overall breast cancer
MR Method
Inverse variance weighted
MR Egger
1/SE_IV
β_IV
40
30
20
10
−0.2
0.0
0.2
0.4
0.6

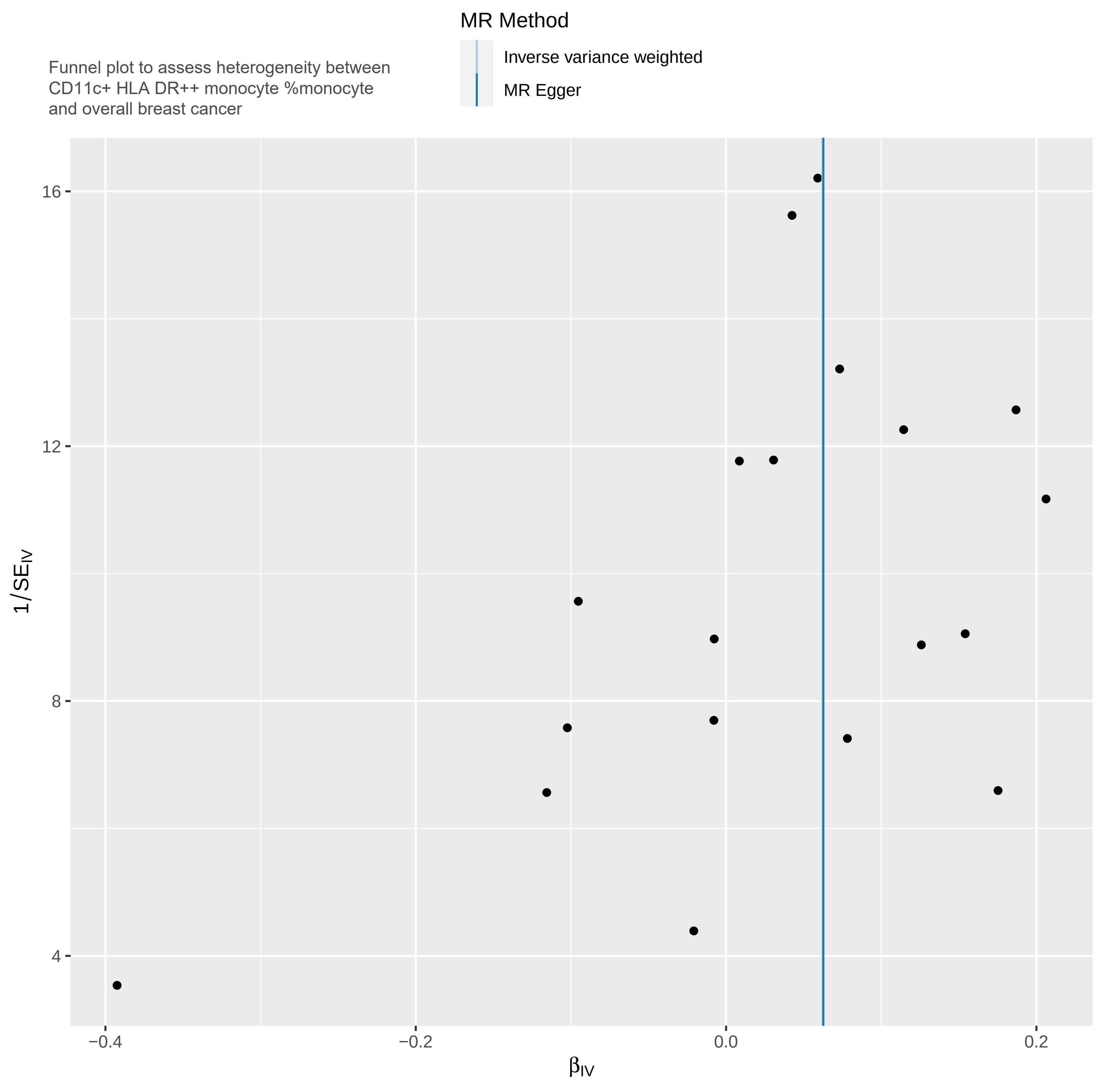

MR Method
Inverse variance weighted
MR Egger
Funnel plot to assess heterogeneity between
CD11c+ HLA DR++ monocyte %monocyte
and overall breast cancer
16
12
8
4
1/SE_IV
−0.4
−0.2
0.0
0.2
β_IV

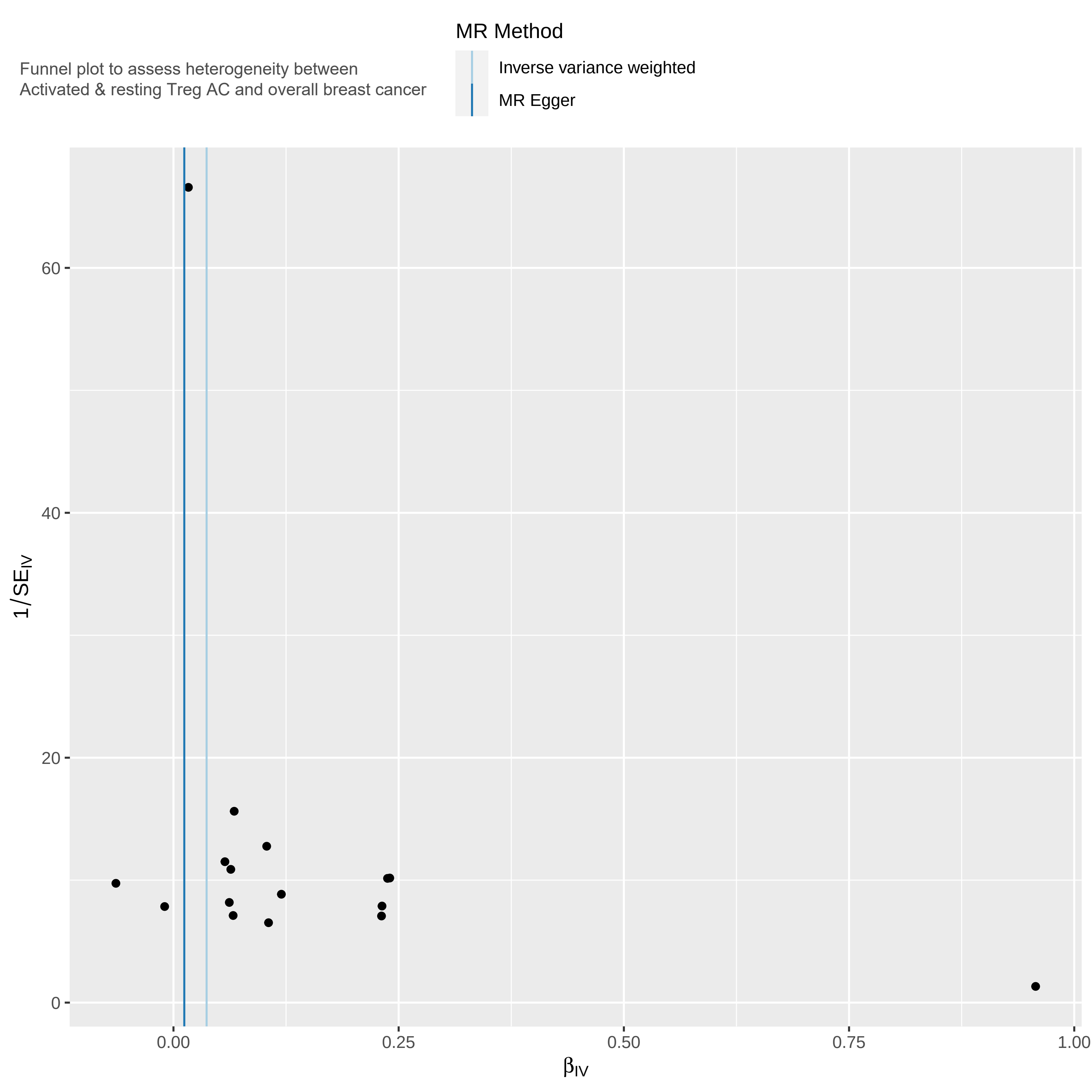

Funnel plot to assess heterogeneity between
Activated & resting Treg AC and overall breast cancer
MR Method
Inverse variance weighted
MR Egger
60
40
20
0
1/SE_IV
0.00
0.25
0.50
0.75
1.00
β_IV

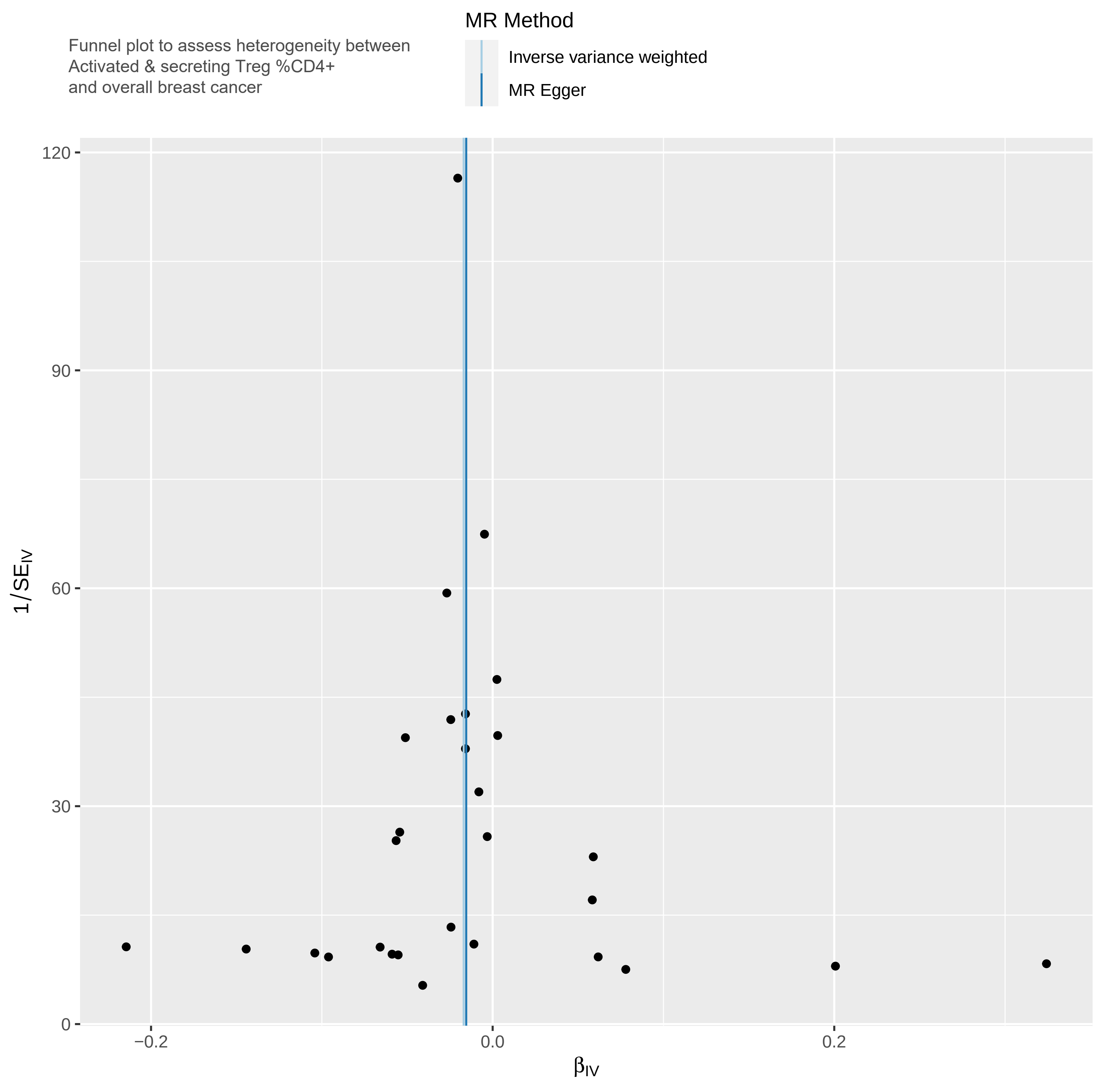

Funnel plot to assess heterogeneity between
Activated & secreting Treg %CD4+
and overall breast cancer
MR Method
Inverse variance weighted
MR Egger
120
90
60
30
0
−0.2
0.0
0.2
1/SE_IV
β_IV

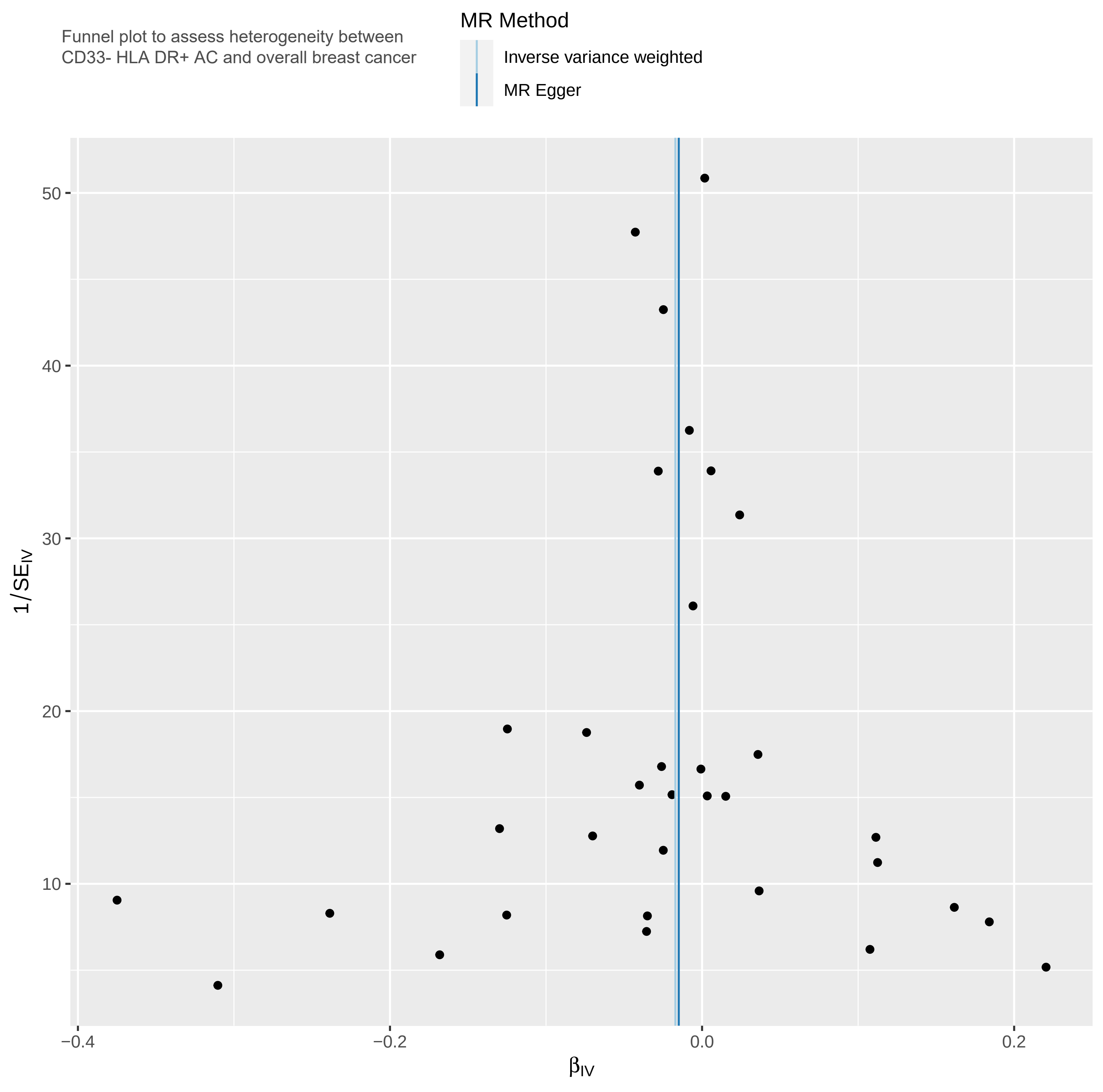

Funnel plot to assess heterogeneity between
CD33- HLA DR+ AC and overall breast cancer
MR Method
Inverse variance weighted
MR Egger
50
40
30
20
10
1/SE$_{IV}$
−0.4
−0.2
0.0
0.2
β_{IV}

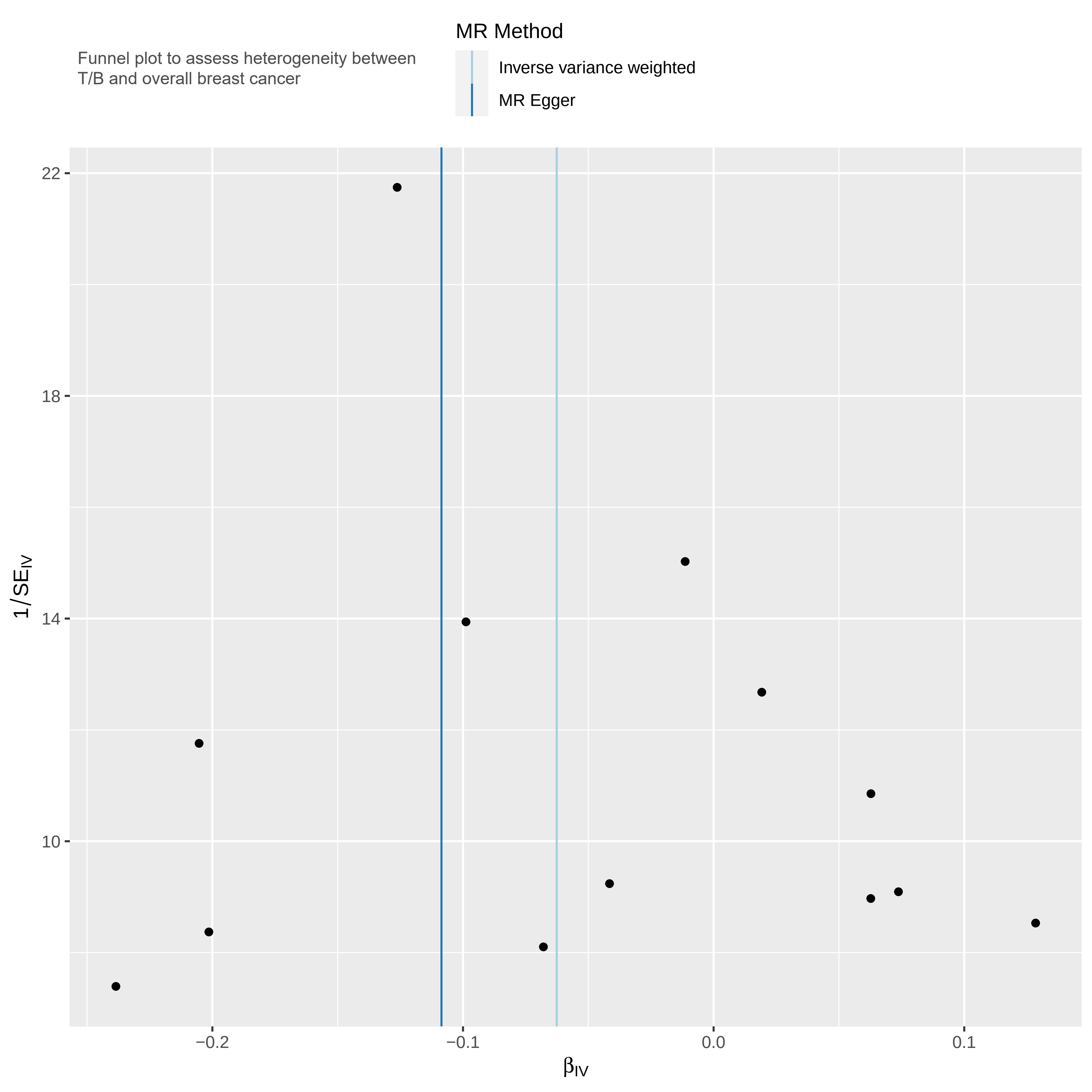
Funnel plot to assess heterogeneity between
T/B and overall breast cancer
MR Method
Inverse variance weighted
MR Egger
22
18
14
10
1/SE$_{IV}$
−0.2
−0.1
0.0
0.1
β$_{IV}$

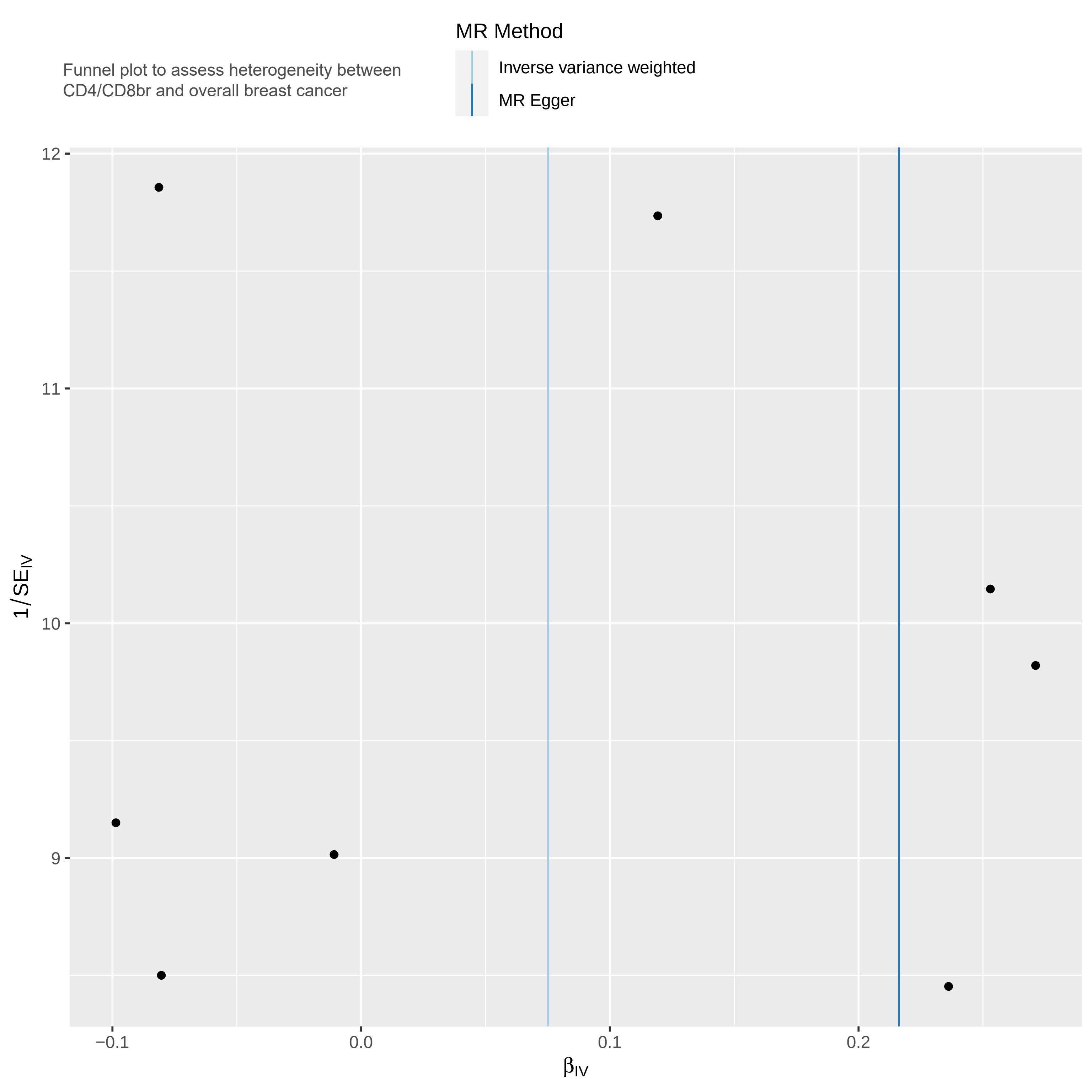

Funnel plot to assess heterogeneity between
CD4/CD8br and overall breast cancer
MR Method
Inverse variance weighted
MR Egger
12
11
10
9
−0.1
0.0
0.1
0.2
1/SE_IV
β_IV

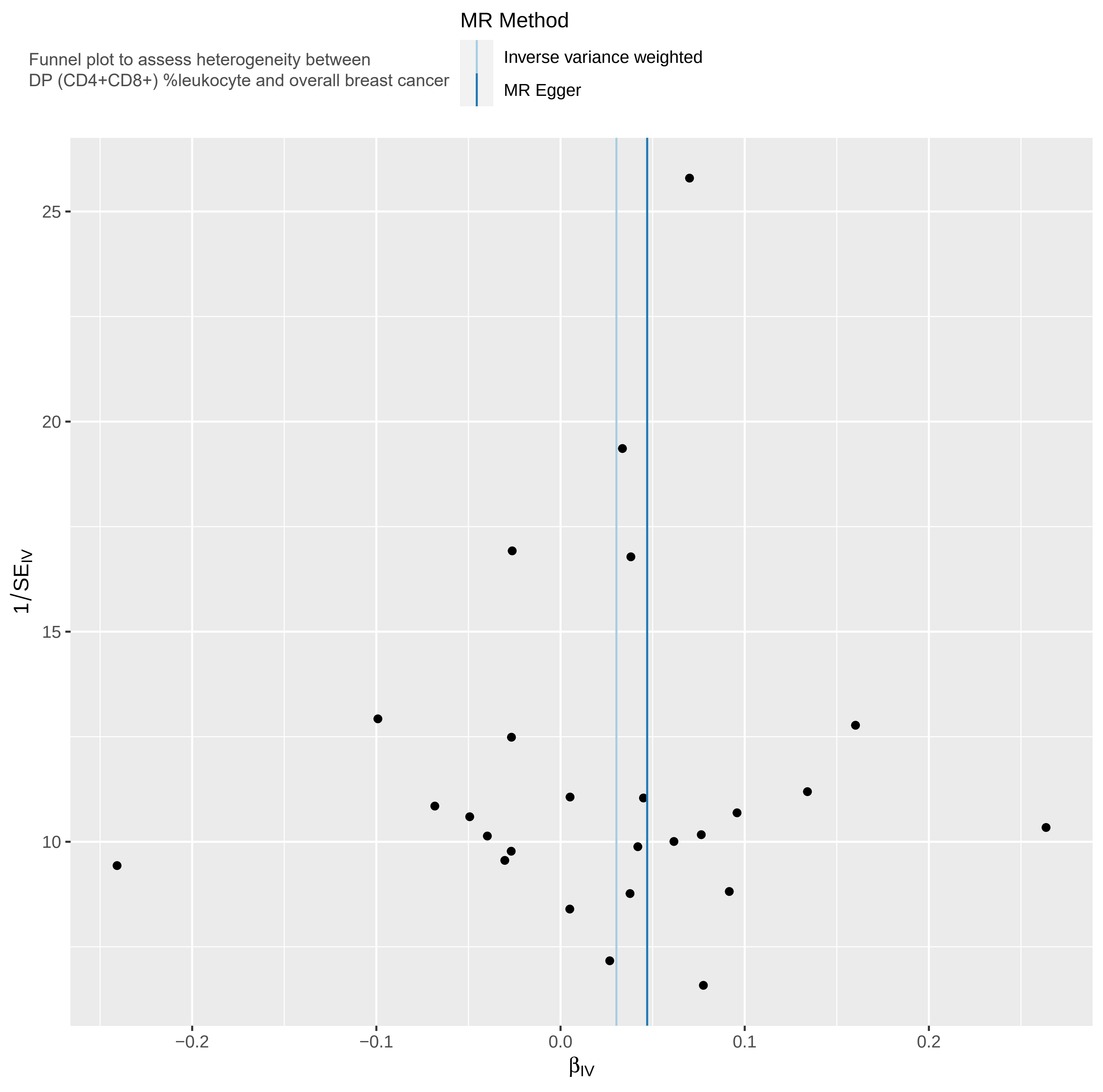
Funnel plot to assess heterogeneity between
DP (CD4+CD8+) %leukocyte and overall breast cancer
MR Method
Inverse variance weighted
MR Egger
1/SE_IV
25
20
15
10
−0.2
−0.1
0.0
0.1
0.2
β_IV

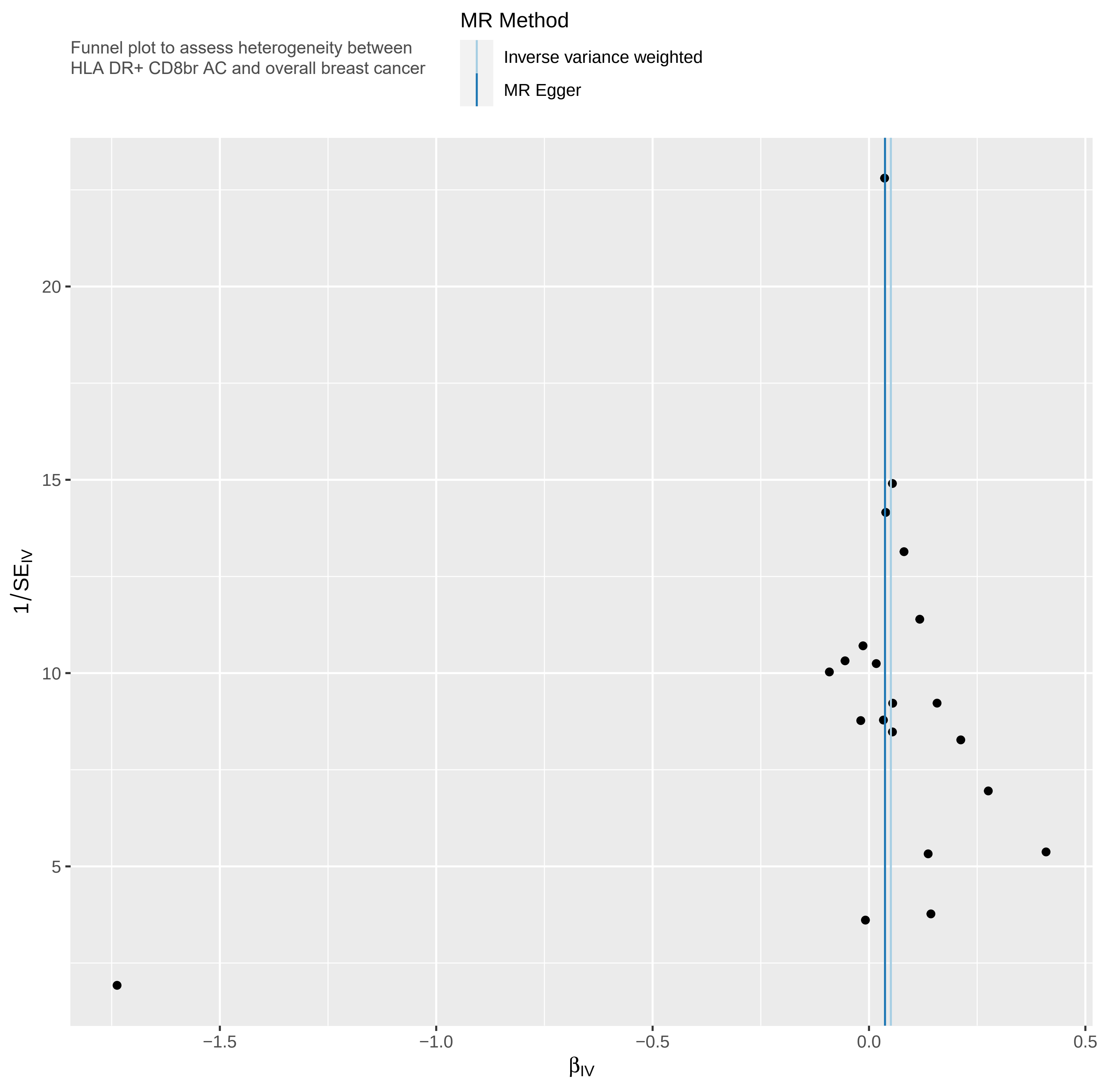

Funnel plot to assess heterogeneity between
HLA DR+ CD8br AC and overall breast cancer
MR Method
Inverse variance weighted
MR Egger
20
15
10
5
1/SE_IV
−1.5
−1.0
−0.5
0.0
0.5
β_IV

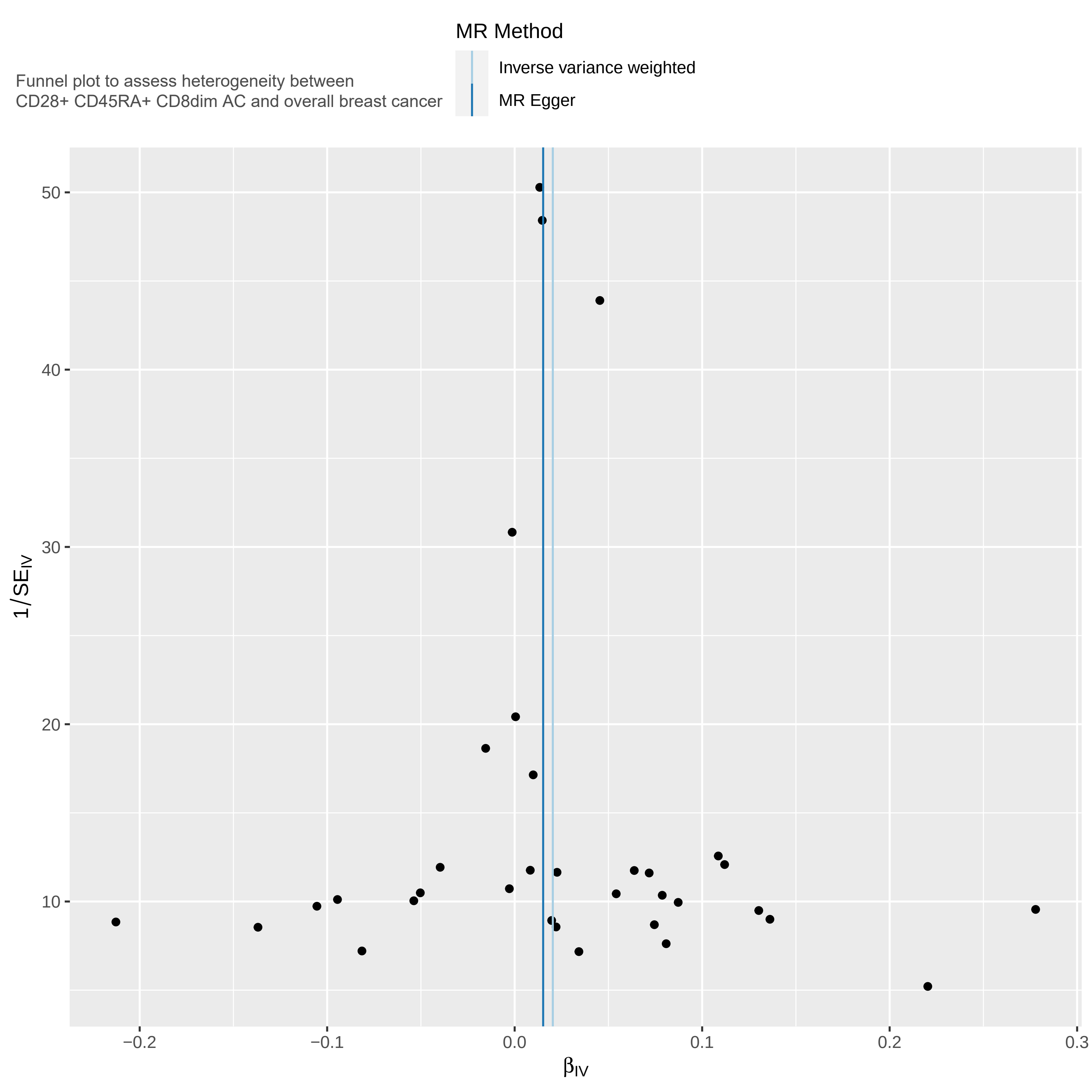

Funnel plot to assess heterogeneity between
CD28+ CD45RA+ CD8dim AC and overall breast cancer
MR Method
Inverse variance weighted
MR Egger
1/SE_IV
β_IV
50
40
30
20
10
−0.2
−0.1
0.0
0.1
0.2
0.3

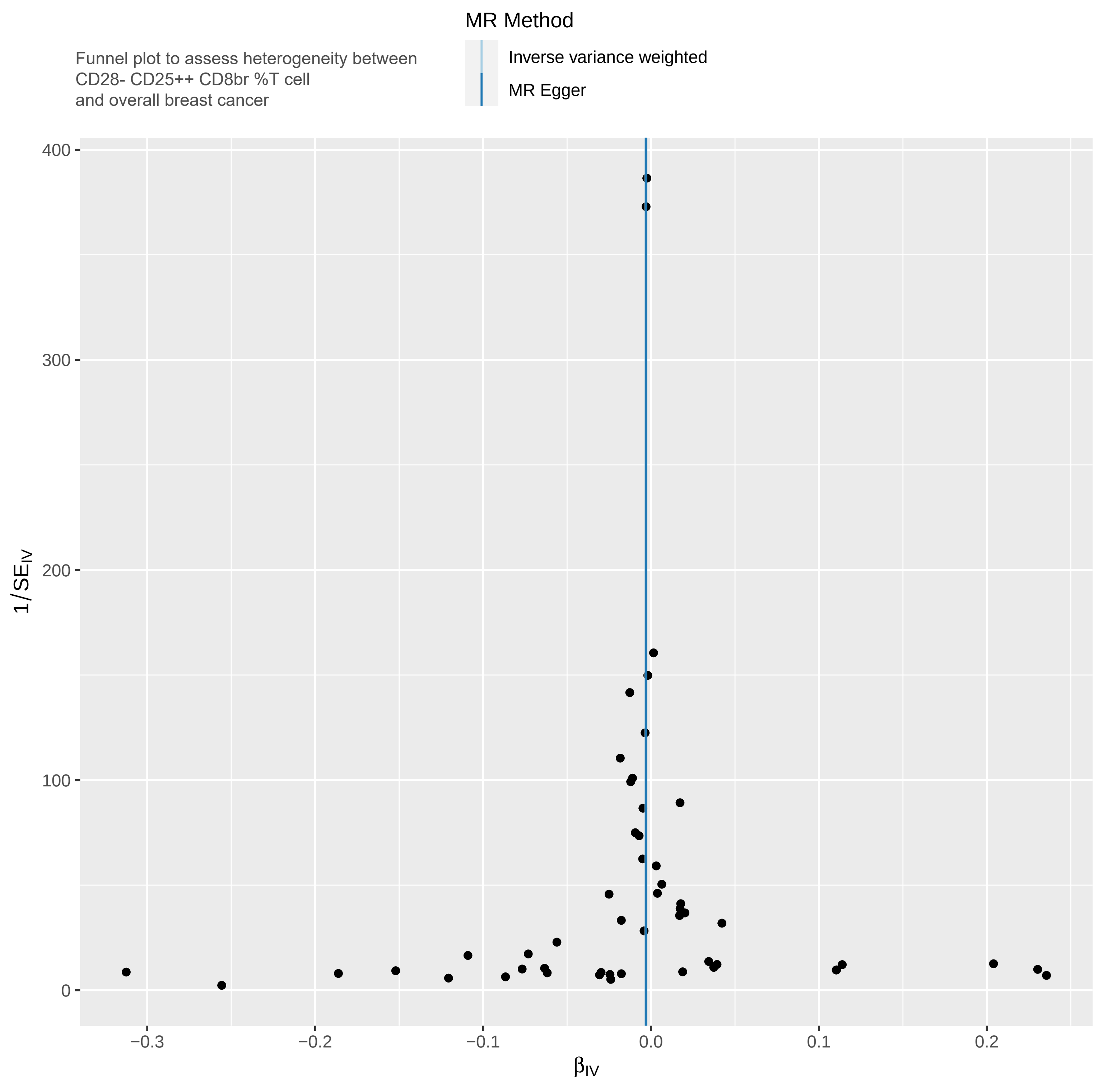
Funnel plot to assess heterogeneity between
CD28- CD25++ CD8br %T cell
and overall breast cancer
MR Method
Inverse variance weighted
MR Egger
400
300
200
100
0
1/SE_IV
−0.3
−0.2
−0.1
0.0
0.1
0.2
β_IV

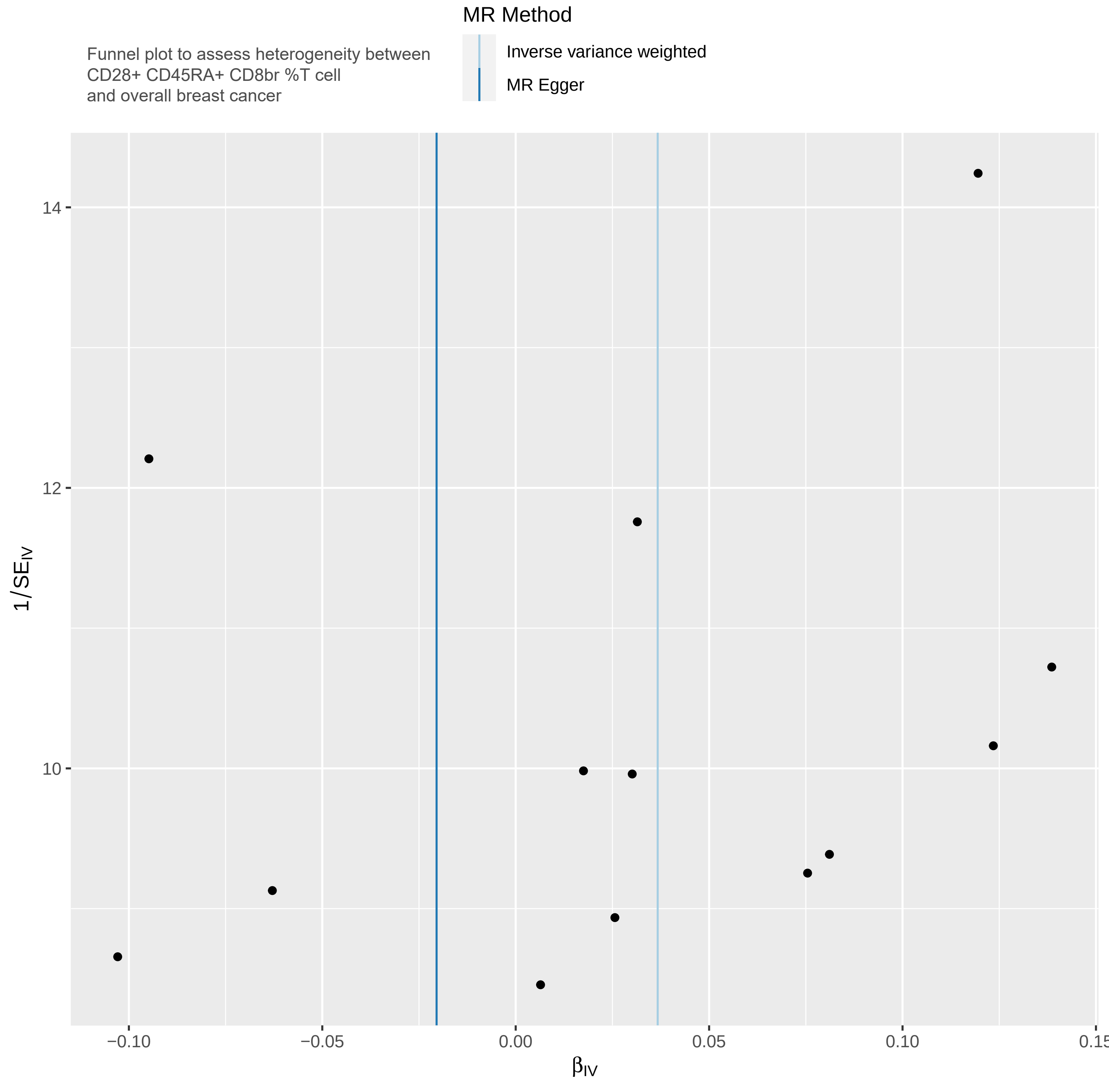
Funnel plot to assess heterogeneity between
CD28+ CD45RA+ CD8br %T cell
and overall breast cancer
MR Method
Inverse variance weighted
MR Egger
1/SE_IV
β_IV
14
12
10
−0.10
−0.05
0.00
0.05
0.10
0.15

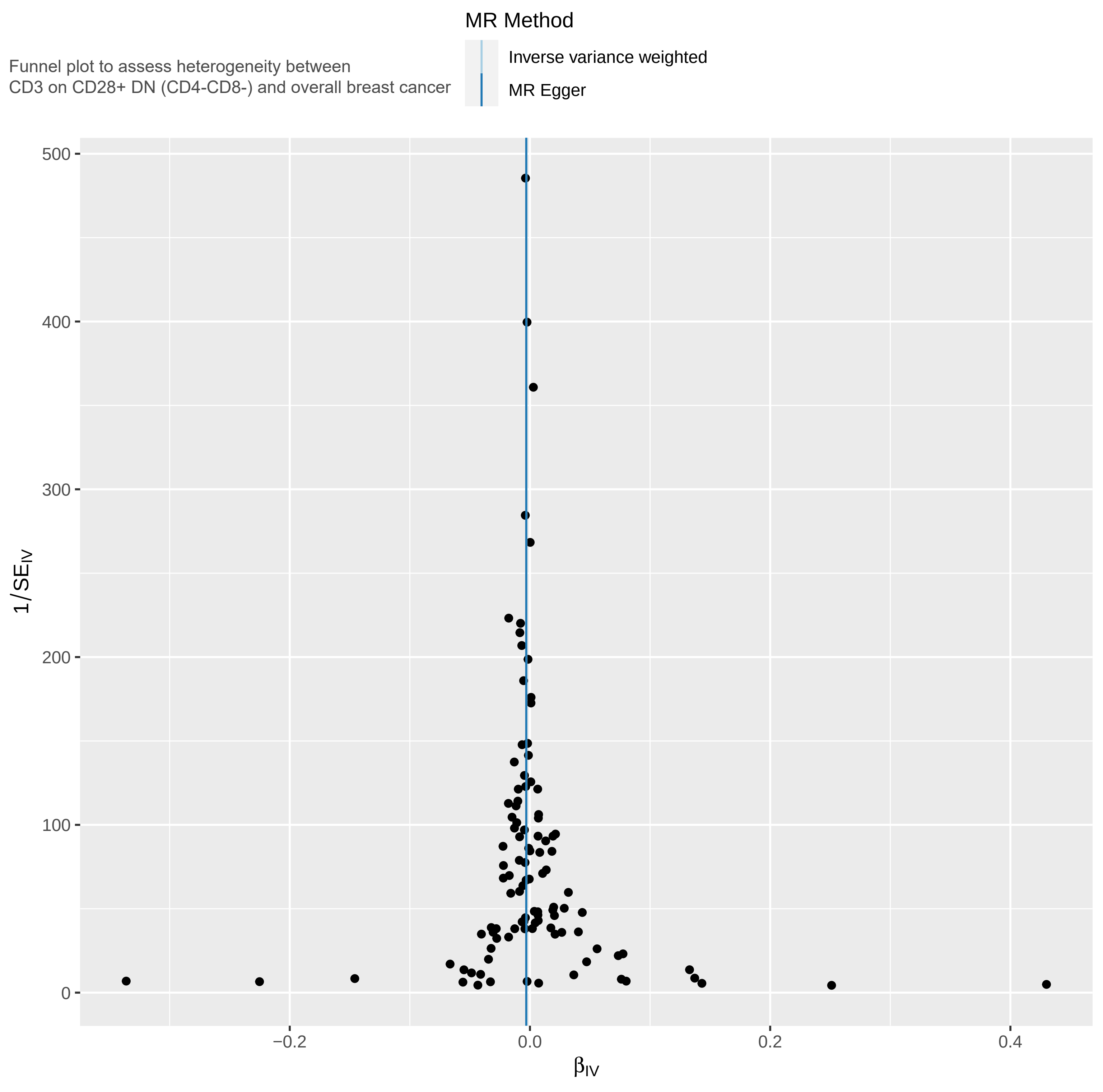

Funnel plot to assess heterogeneity between
CD3 on CD28+ DN (CD4-CD8-) and overall breast cancer
MR Method
Inverse variance weighted
MR Egger
500
400
300
200
100
0
1/SE_IV
−0.2
0.0
0.2
0.4
β_IV

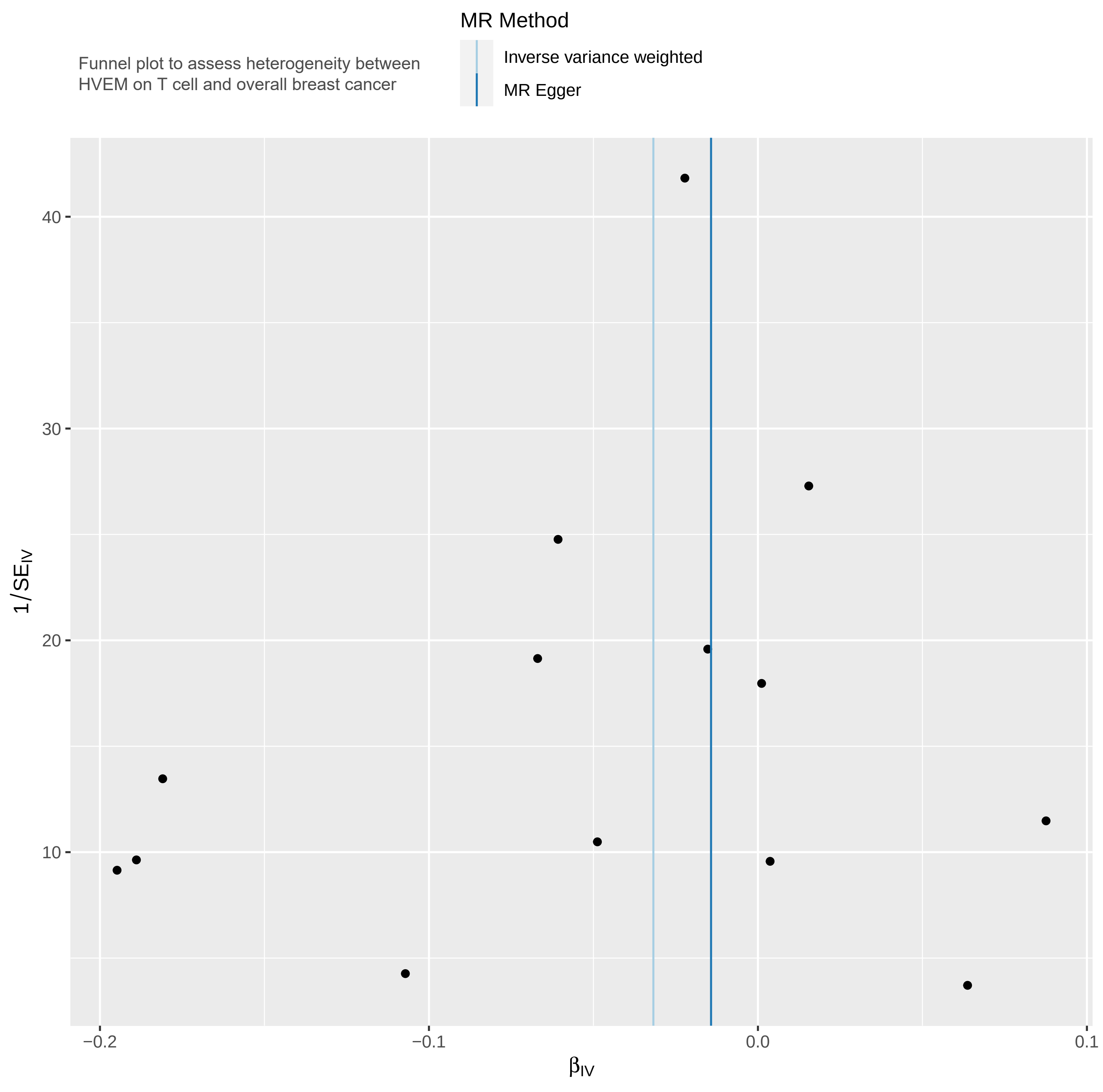
Funnel plot to assess heterogeneity between
HVEM on T cell and overall breast cancer
MR Method
Inverse variance weighted
MR Egger
40
30
20
10
1/SE$_{IV}$
−0.2
−0.1
0.0
0.1
β_{IV}

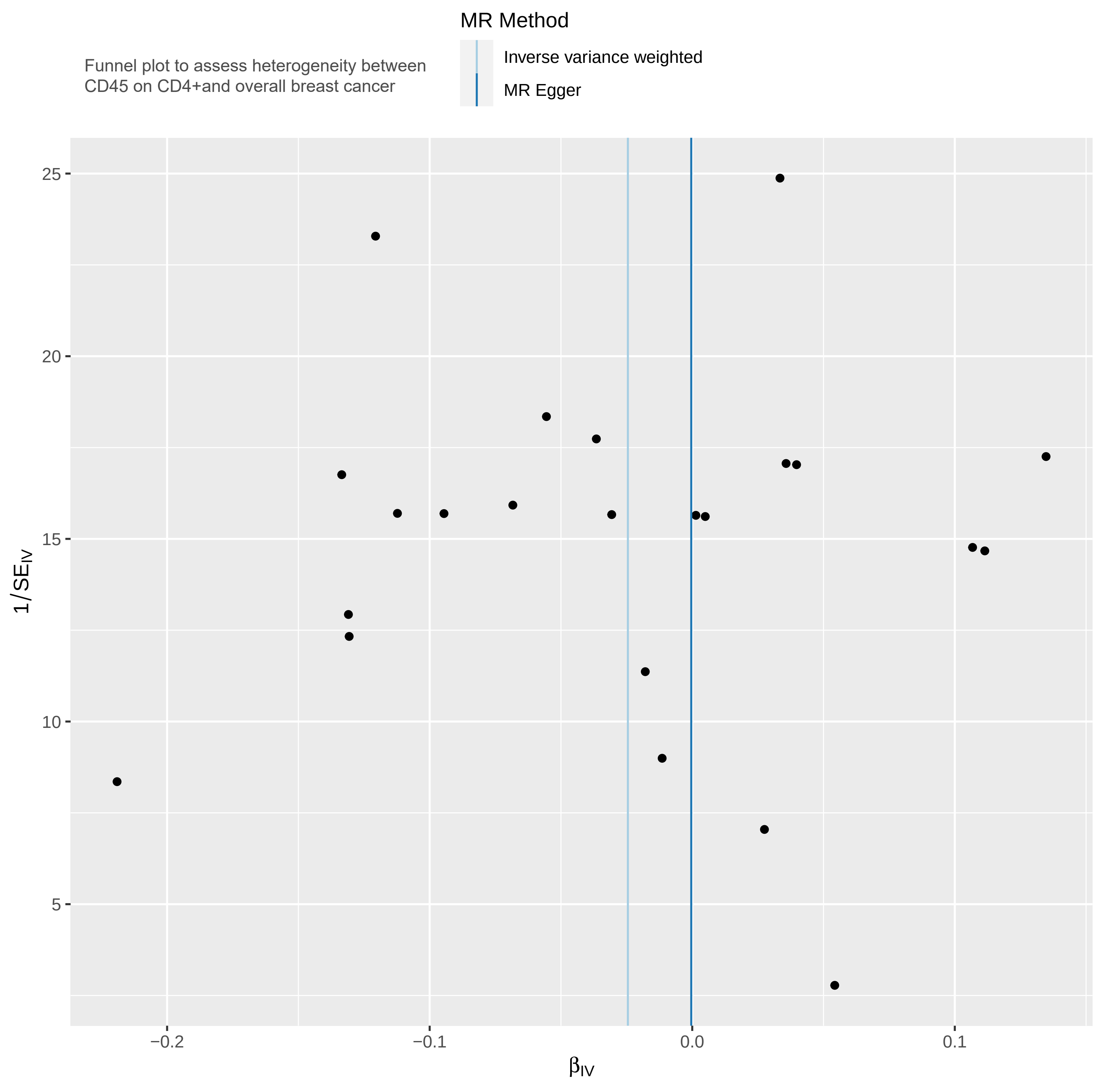

MR Method
Inverse variance weighted
MR Egger
Funnel plot to assess heterogeneity between
CD45 on CD4+and overall breast cancer
25
20
15
10
5
1/SEIV
−0.2
−0.1
0.0
0.1
βIV

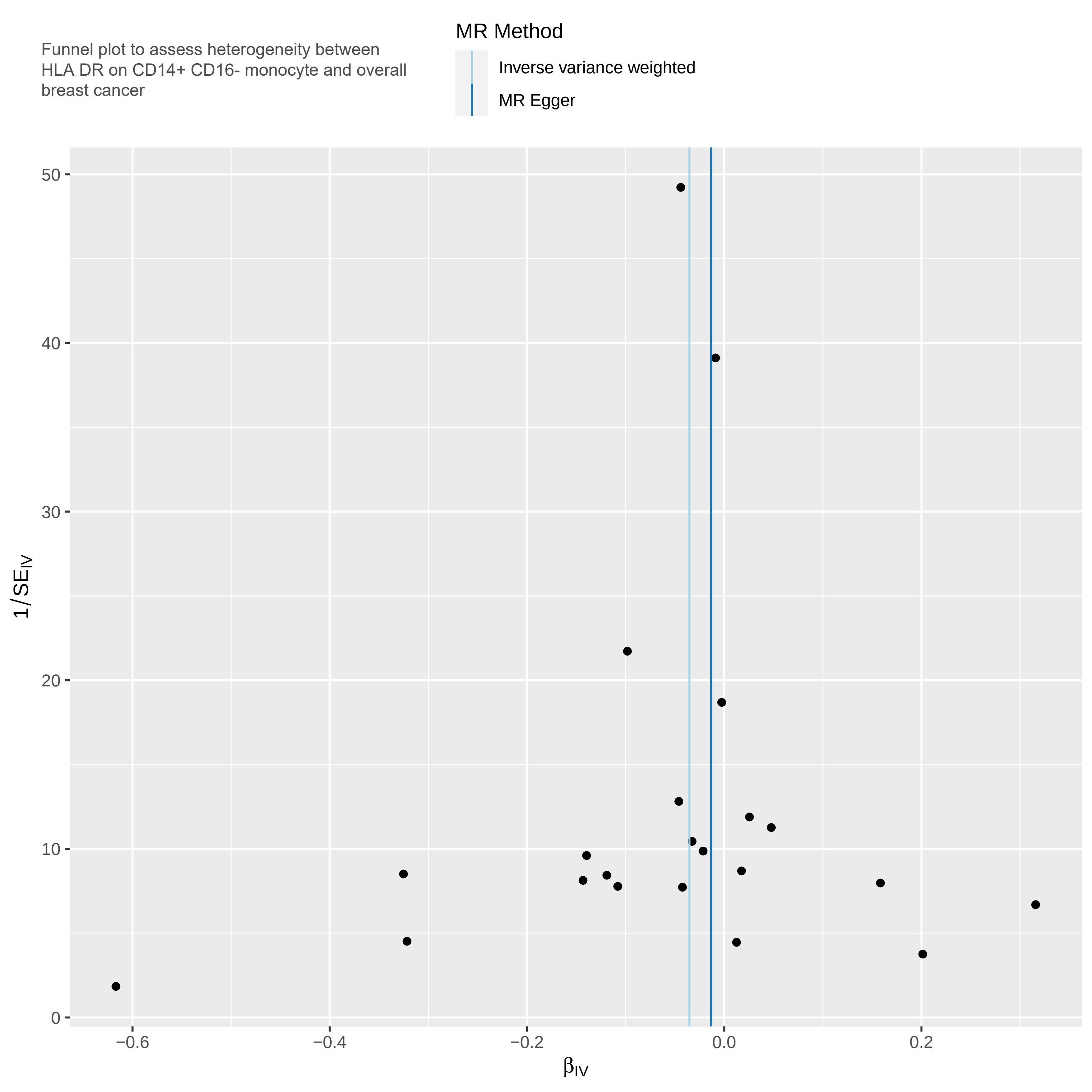
Funnel plot to assess heterogeneity between HLA DR on CD14+ CD16- monocyte and overall breast cancer
MR Method
Inverse variance weighted
MR Egger
1/SE_IV
β_IV
50
40
30
20
10
0
−0.6
−0.4
−0.2
0.0
0.2

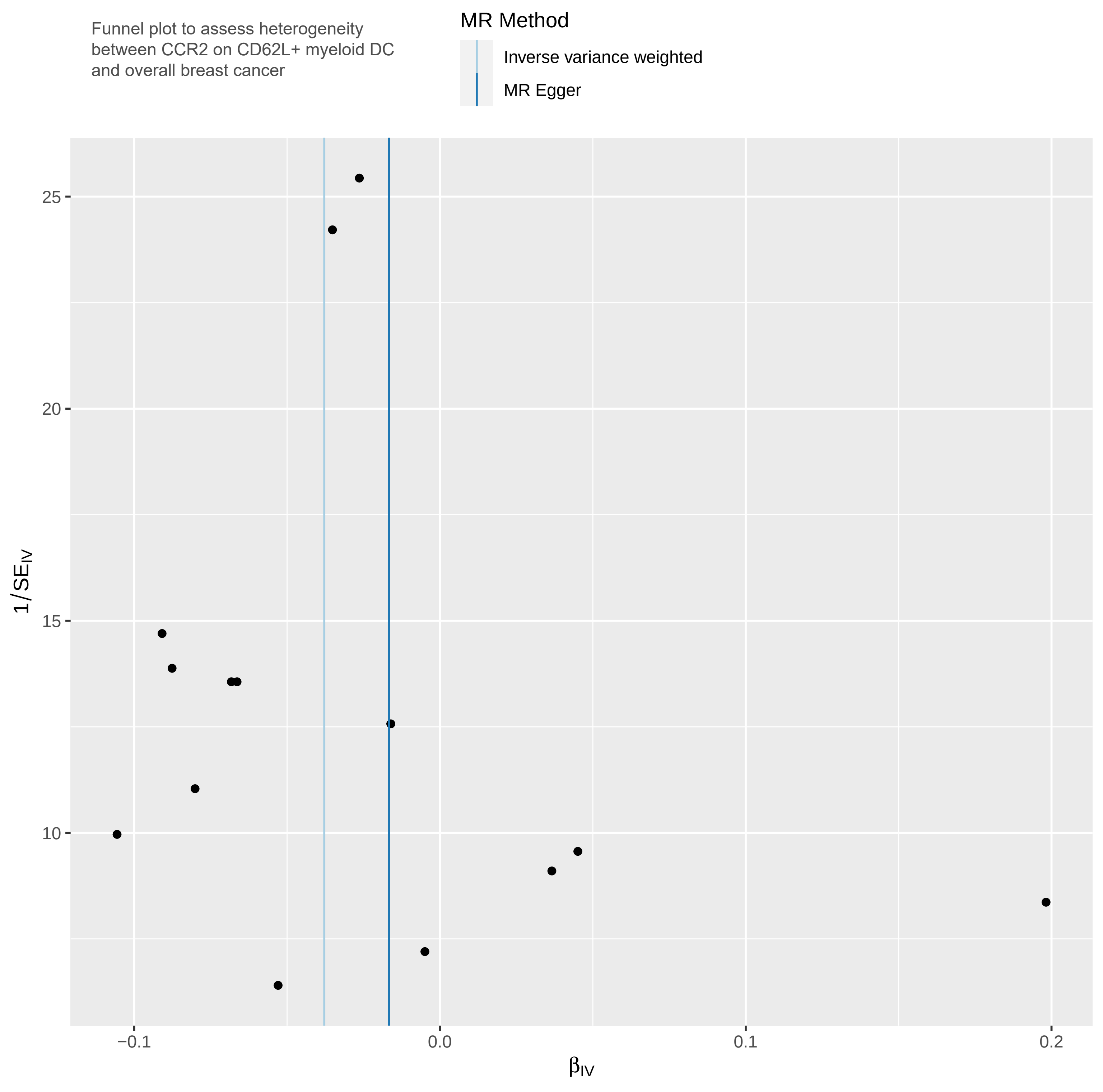
Funnel plot to assess heterogeneity between CCR2 on CD62L+ myeloid DC and overall breast cancer
MR Method
Inverse variance weighted
MR Egger
25
20
15
10
1/SE_IV
−0.1
0.0
0.1
0.2
β_IV

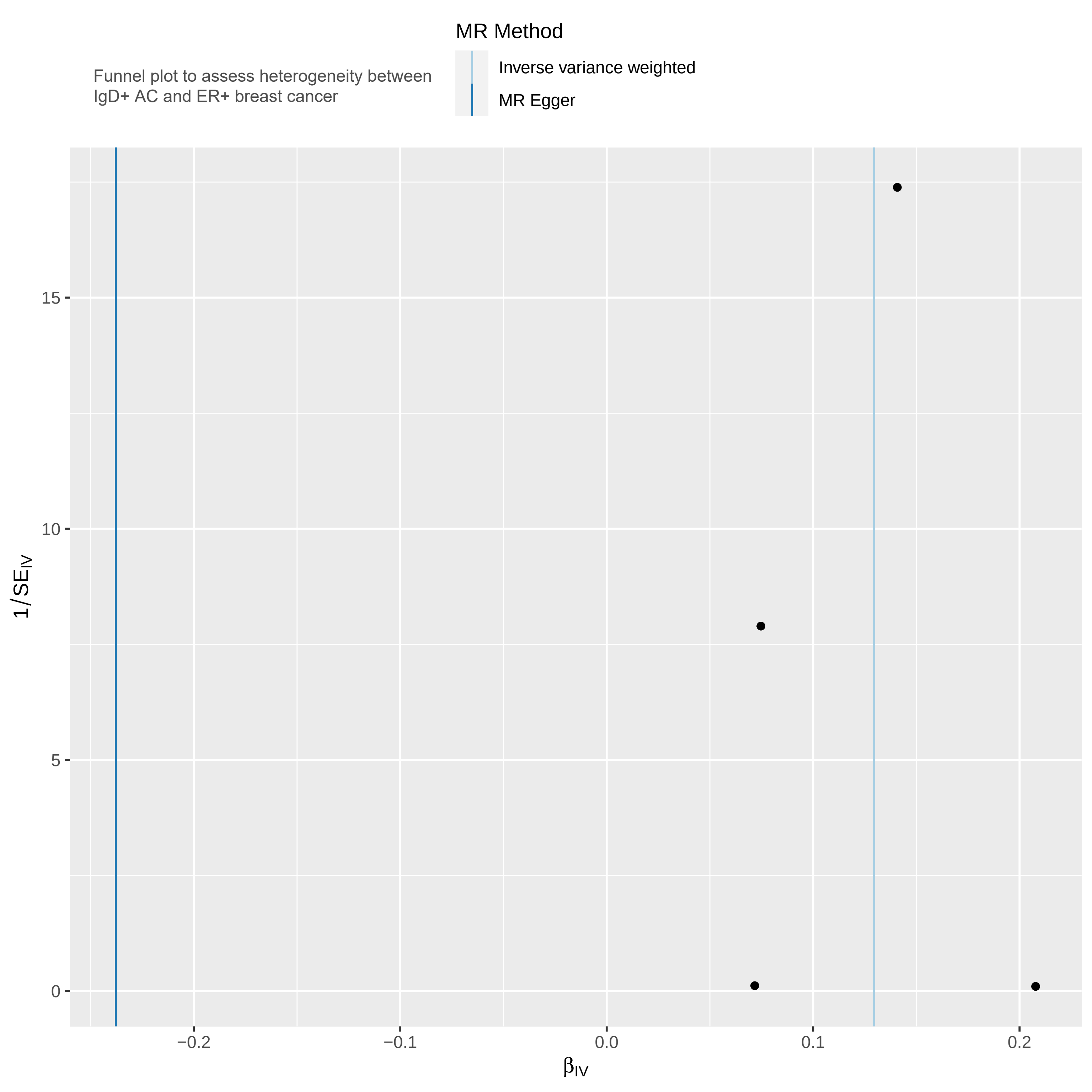

MR Method
Inverse variance weighted
MR Egger
Funnel plot to assess heterogeneity between
IgD+ AC and ER+ breast cancer
15
10
5
0
1/SE_IV
−0.2
−0.1
0.0
0.1
0.2
β_IV

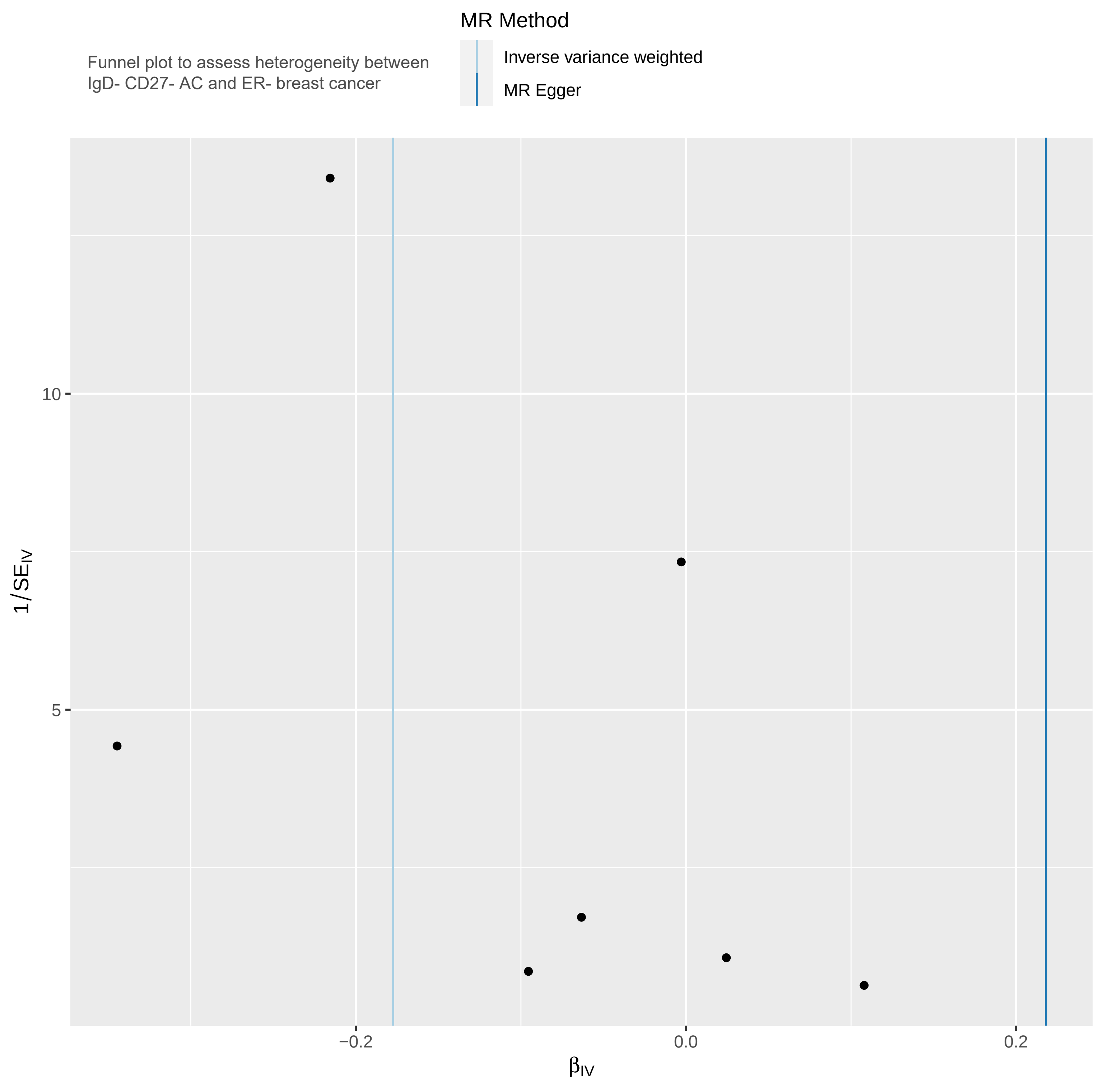

Funnel plot to assess heterogeneity between
IgD- CD27- AC and ER- breast cancer
MR Method
Inverse variance weighted
MR Egger
10
5
1/SE$_{IV}$
−0.2
0.0
0.2
β$_{IV}$

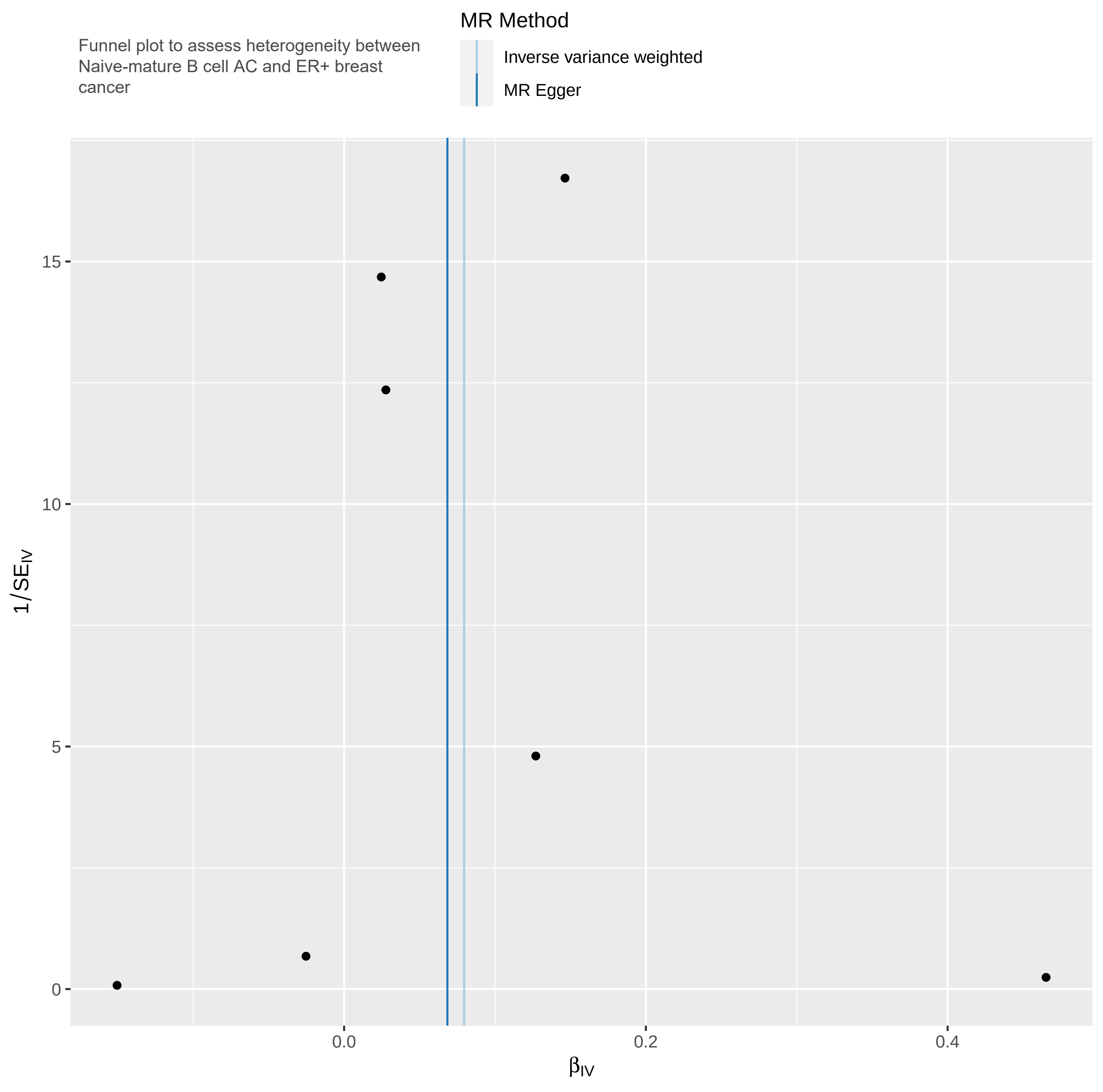

Funnel plot to assess heterogeneity between Naive-mature B cell AC and ER+ breast cancer
MR Method
Inverse variance weighted
MR Egger
15
10
5
0
1/SE$_{IV}$
0.0
0.2
0.4
β$_{IV}$

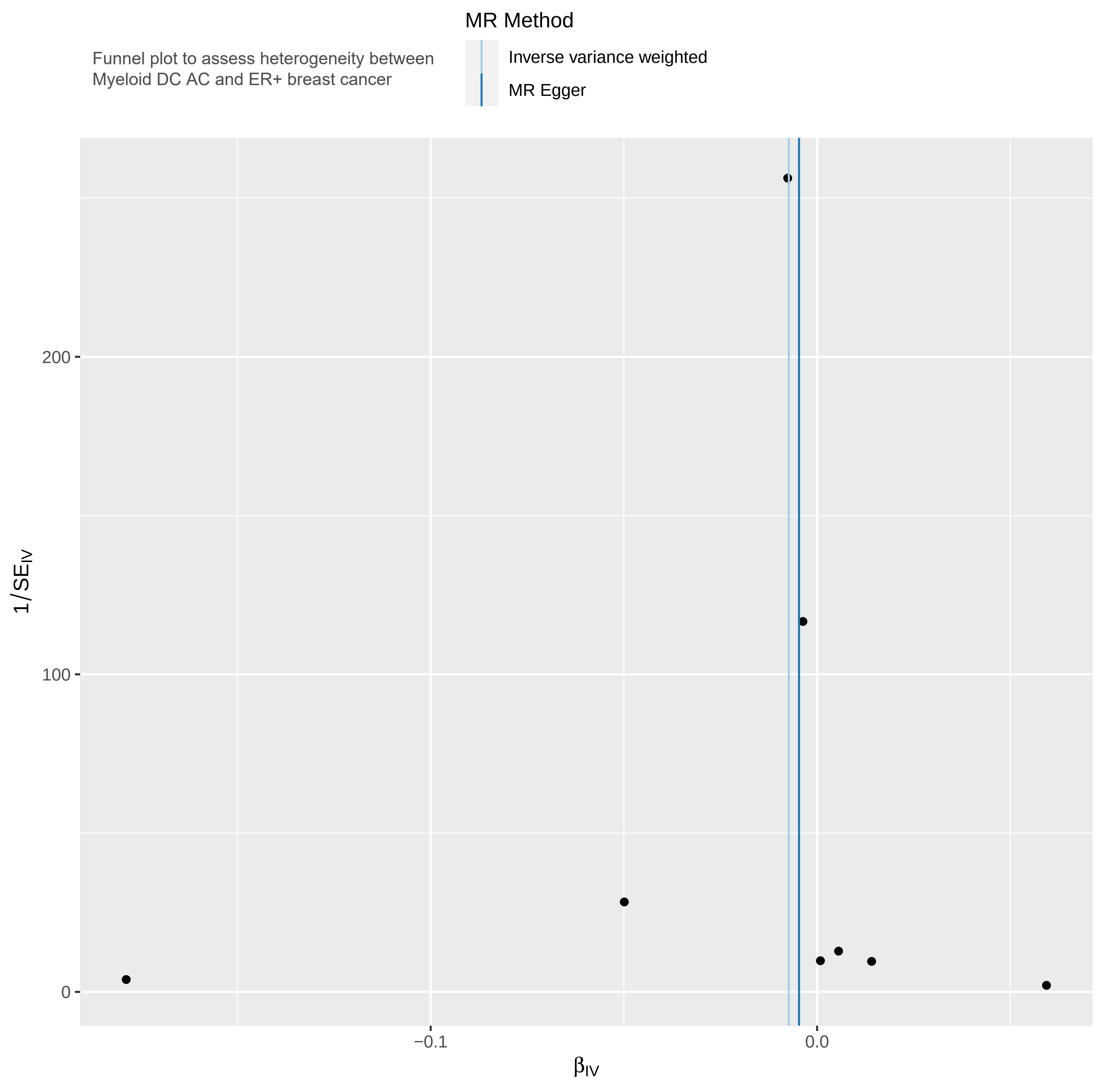

MR Method
Funnel plot to assess heterogeneity between
Myeloid DC AC and ER+ breast cancer
Inverse variance weighted
MR Egger
200
100
0
1/SE_IV
−0.1
0.0
β_IV

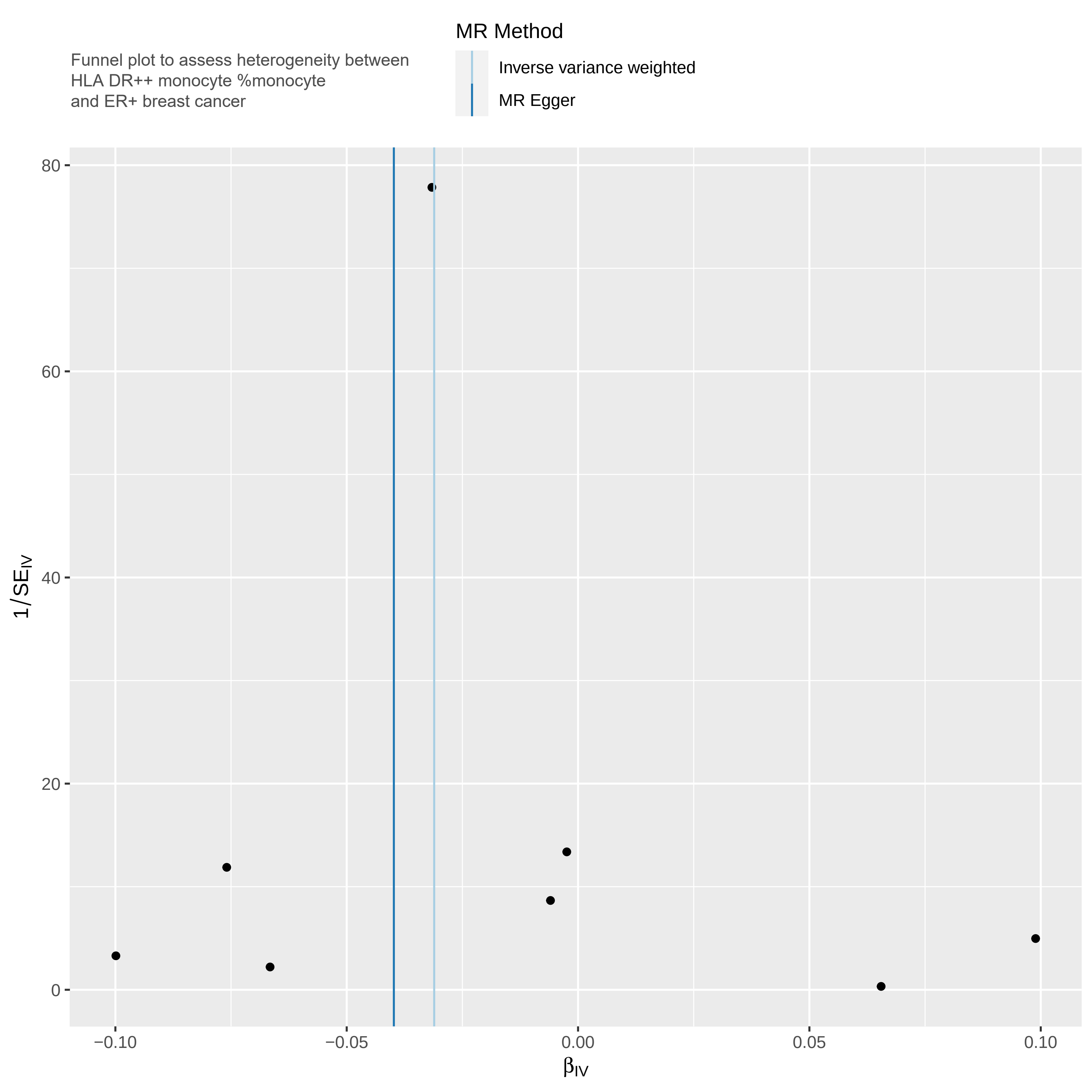
MR Method
Inverse variance weighted
MR Egger
Funnel plot to assess heterogeneity between
HLA DR++ monocyte %monocyte
and ER+ breast cancer
80
60
40
20
0
1/SE_IV
−0.10
−0.05
0.00
0.05
0.10
β_IV

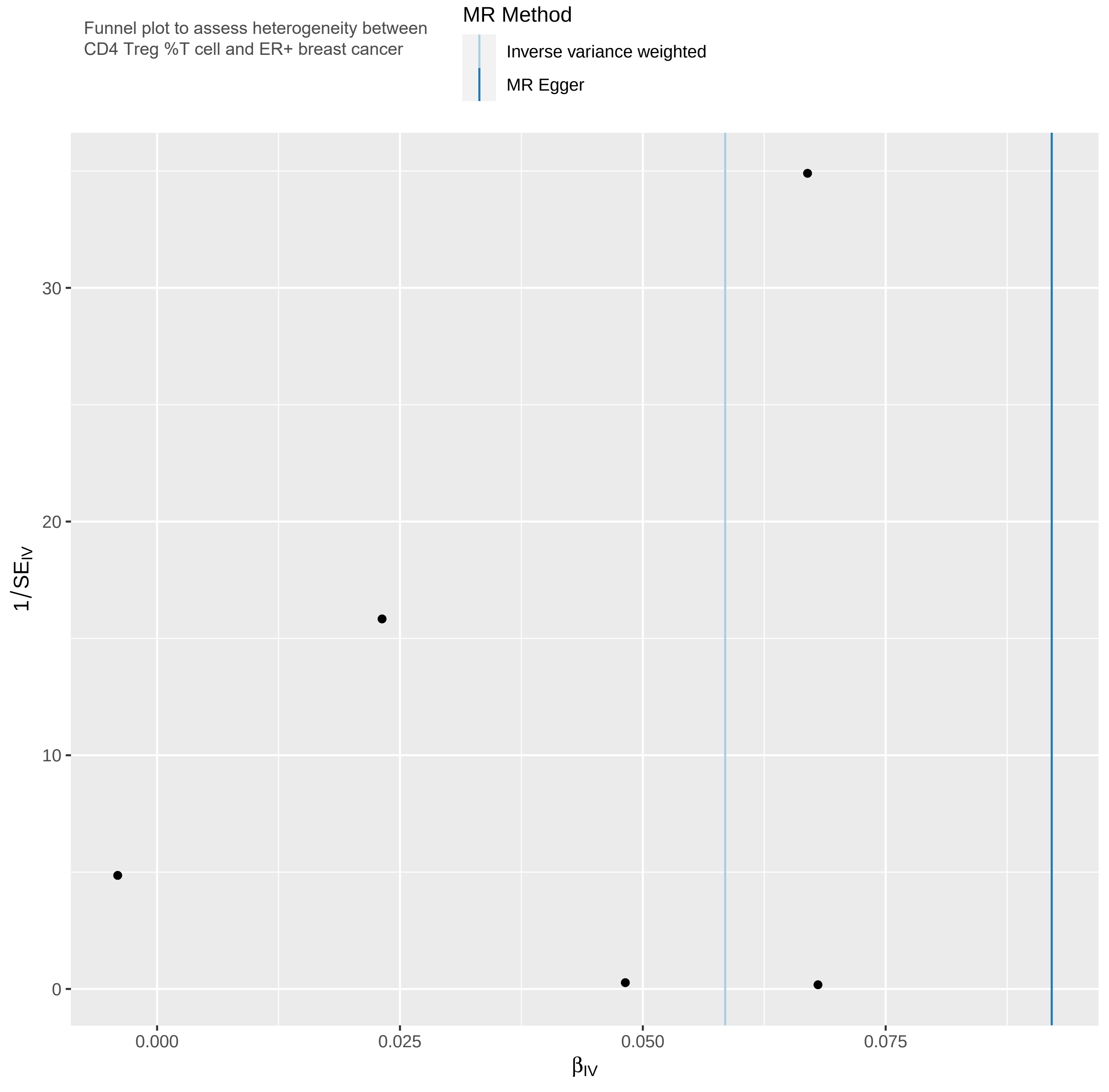
Funnel plot to assess heterogeneity between
CD4 Treg %T cell and ER+ breast cancer
MR Method
Inverse variance weighted
MR Egger
30
20
10
0
1/SE$_{IV}$
0.000
0.025
0.050
0.075
β$_{IV}$

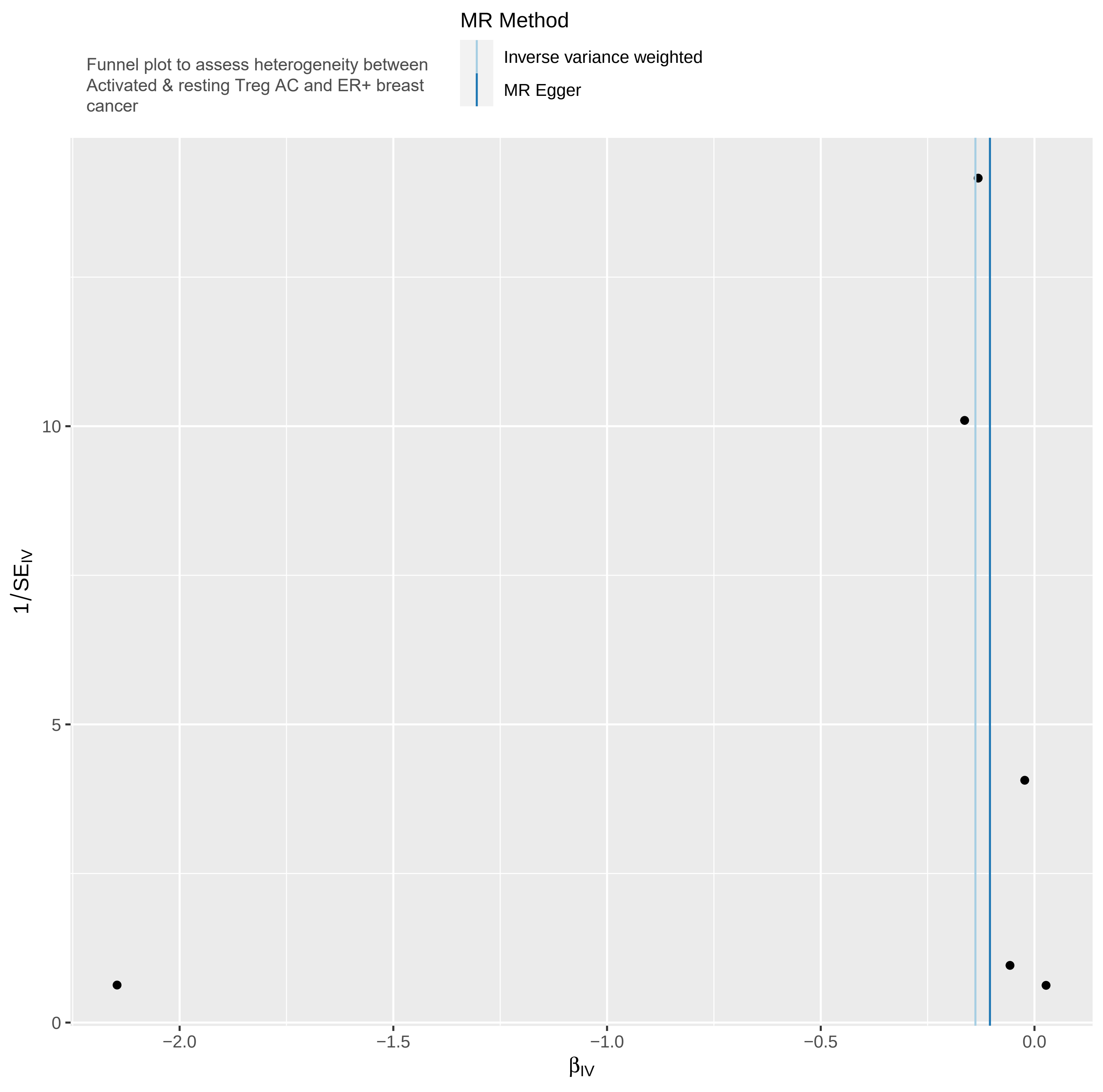

Funnel plot to assess heterogeneity between Activated & resting Treg AC and ER+ breast cancer
MR Method
Inverse variance weighted
MR Egger
1/SE_IV
β_IV
10
5
0
−2.0
−1.5
−1.0
−0.5
0.0

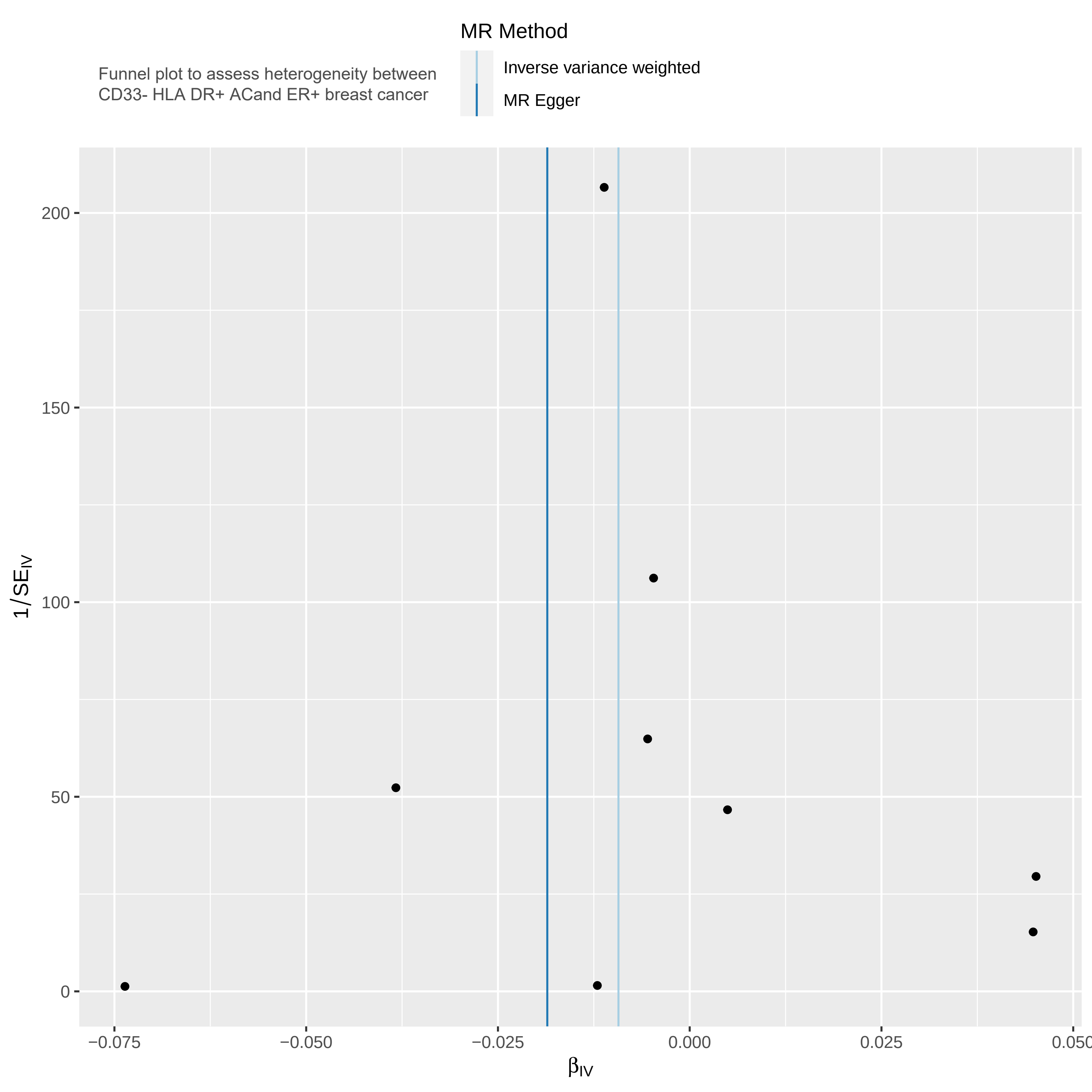

Funnel plot to assess heterogeneity between
CD33- HLA DR+ ACand ER+ breast cancer
MR Method
Inverse variance weighted
MR Egger
200
150
100
50
0
1/SE_IV
−0.075
−0.050
−0.025
0.000
0.025
0.050
β_IV

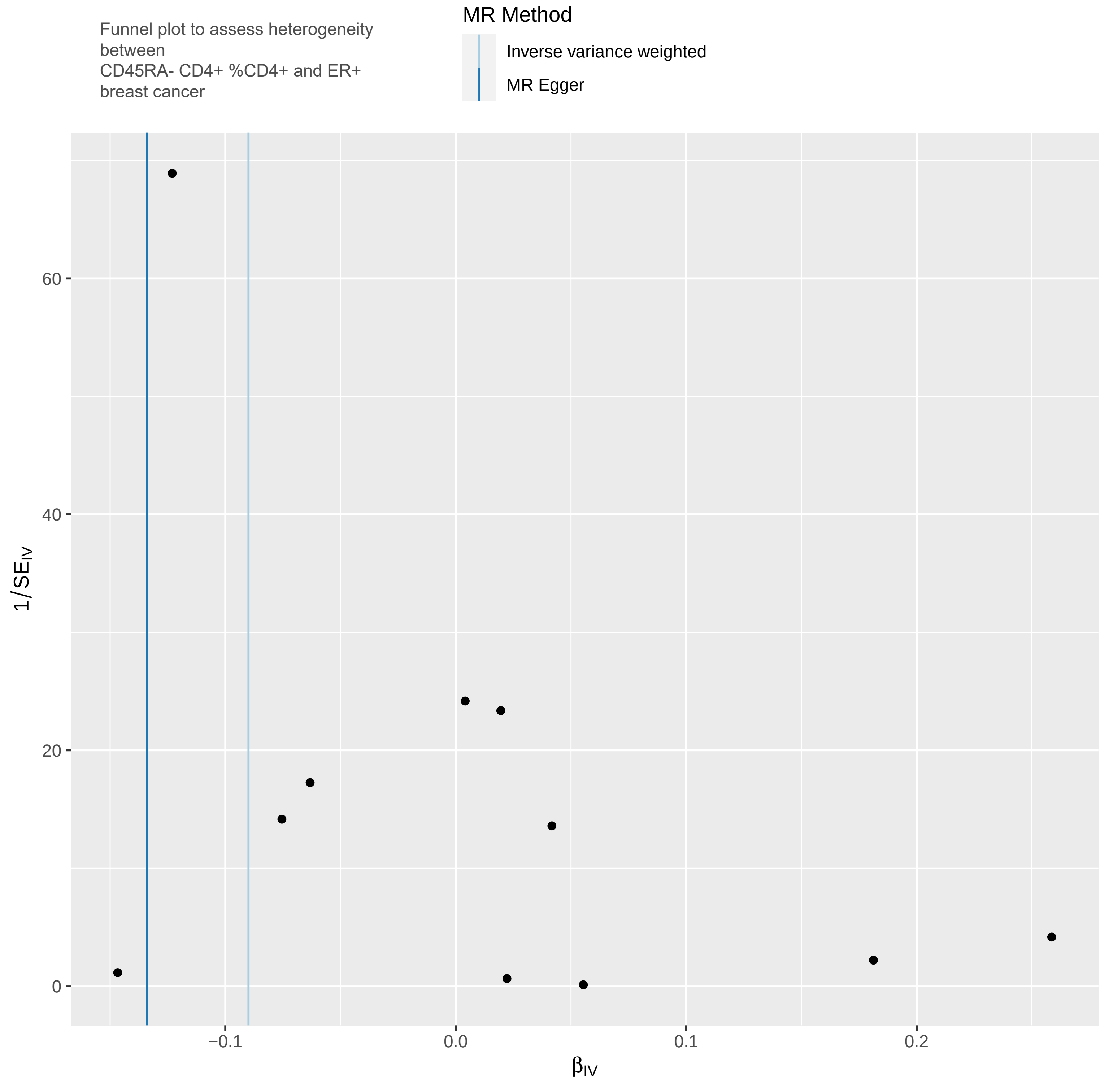
Funnel plot to assess heterogeneity between
CD45RA- CD4+ %CD4+ and ER+ breast cancer
MR Method
Inverse variance weighted
MR Egger
60
40
20
0
1/SE_IV
−0.1
0.0
0.1
0.2
β_IV

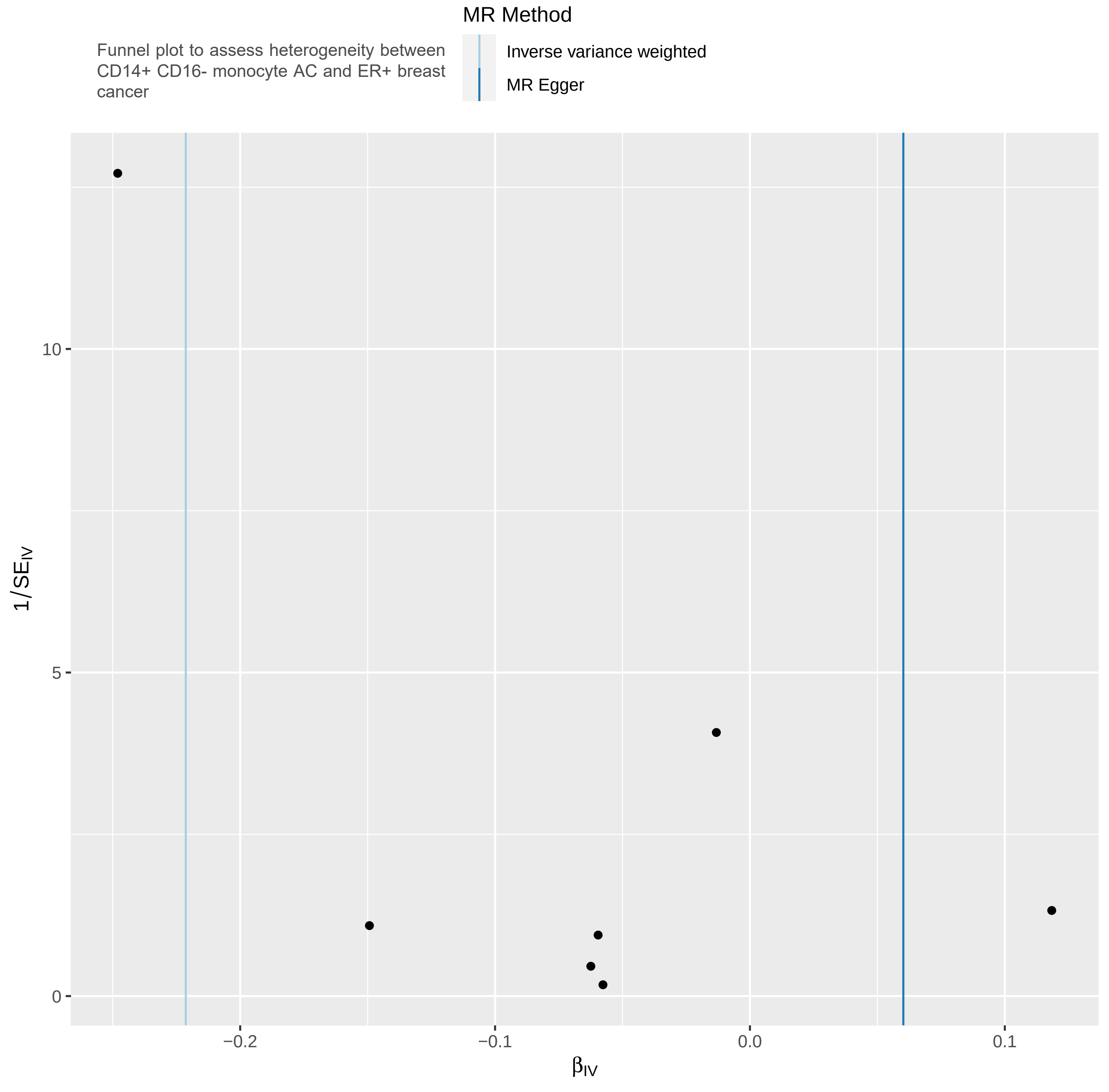

Funnel plot to assess heterogeneity between CD14+ CD16- monocyte AC and ER+ breast cancer
MR Method
Inverse variance weighted
MR Egger
10
5
0
−0.2
−0.1
0.0
0.1
$1/SE_{IV}$
β_{IV}

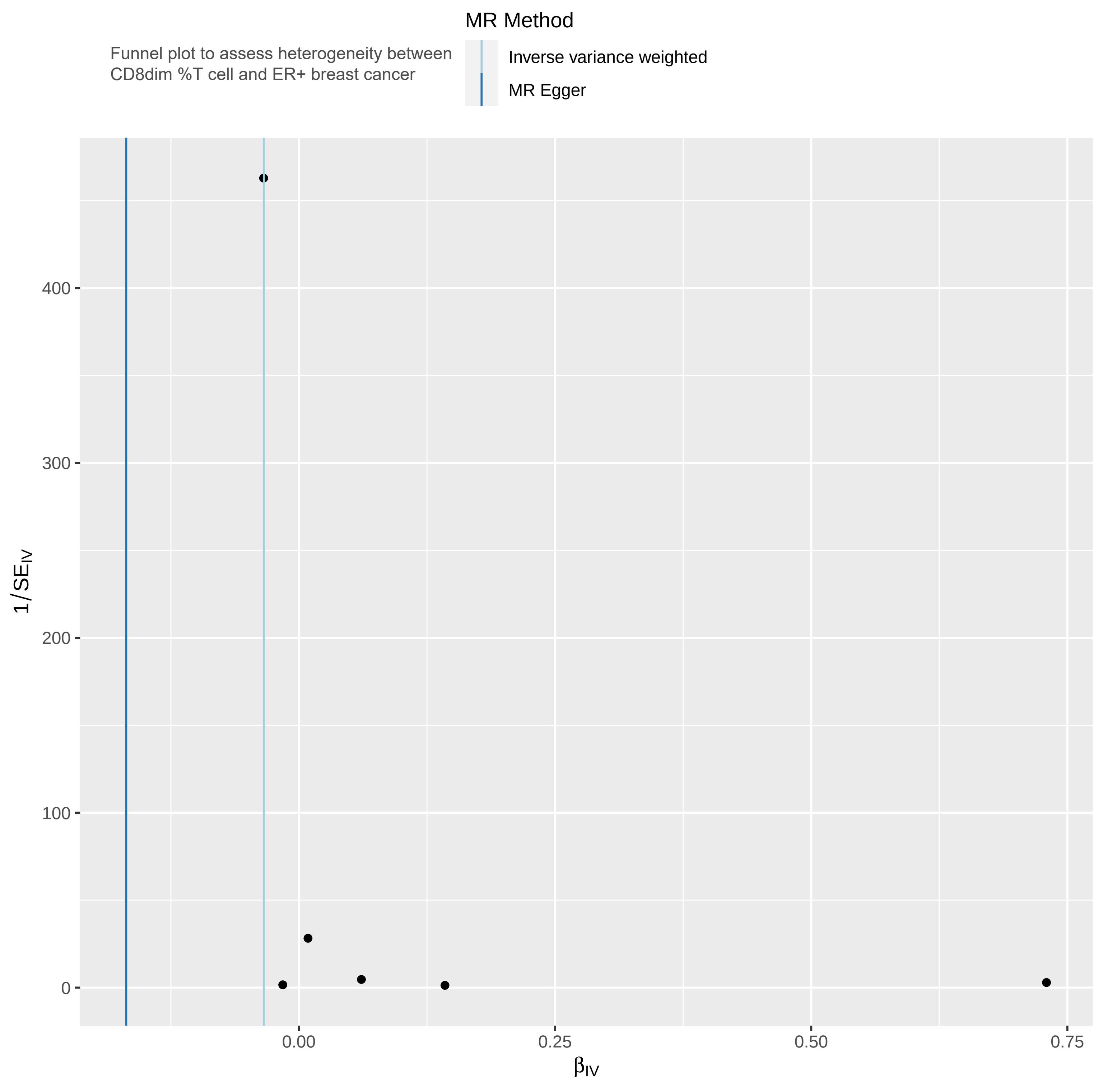

MR Method
Inverse variance weighted
MR Egger
Funnel plot to assess heterogeneity between
CD8dim %T cell and ER+ breast cancer
400
300
200
100
0
1/SE$_{IV}$
0.00
0.25
0.50
0.75
β$_{IV}$

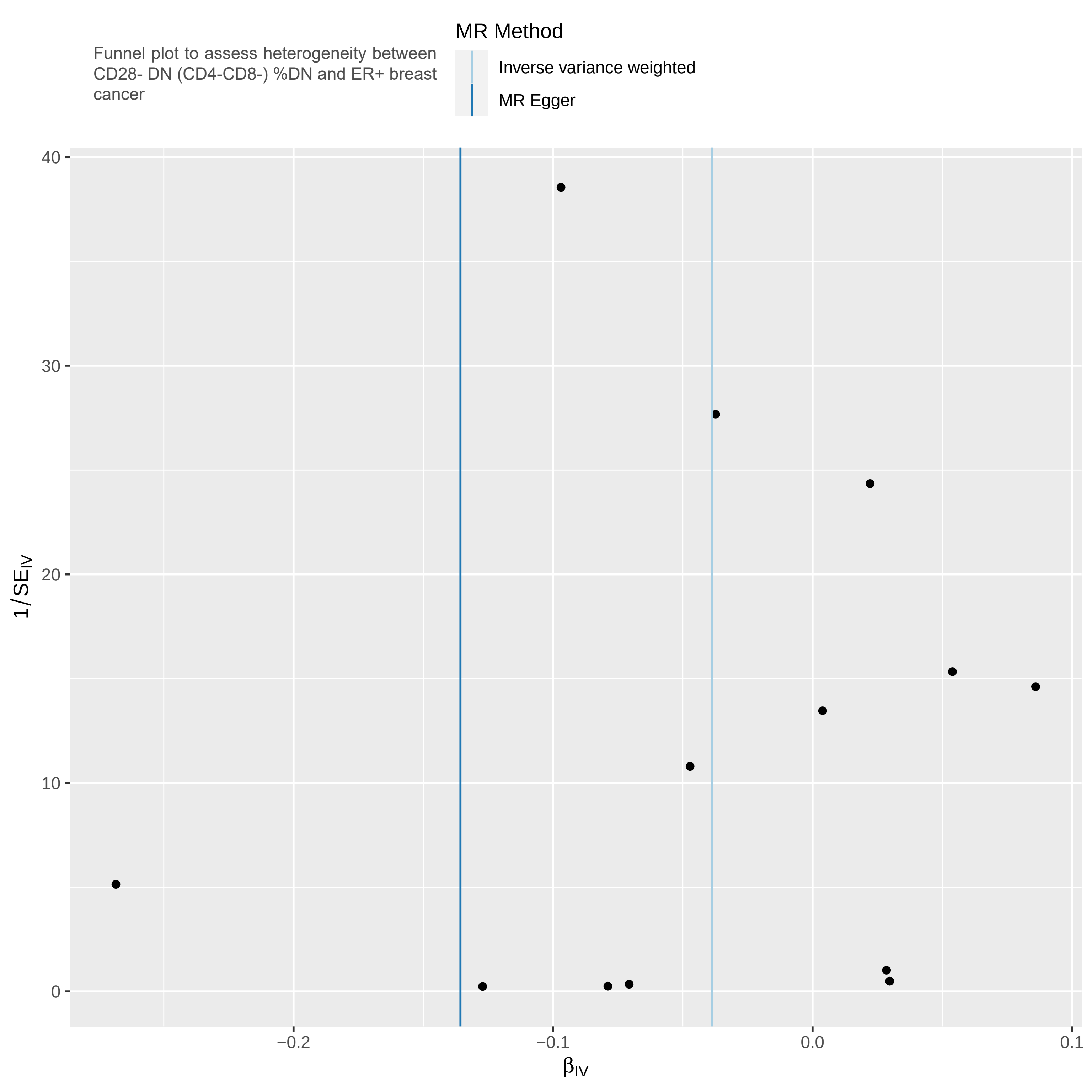
Funnel plot to assess heterogeneity between CD28- DN (CD4-CD8-) %DN and ER+ breast cancer
MR Method
Inverse variance weighted
MR Egger
40
30
20
10
0
−0.2
−0.1
0.0
0.1
$1/SE_{IV}$
β_{IV}

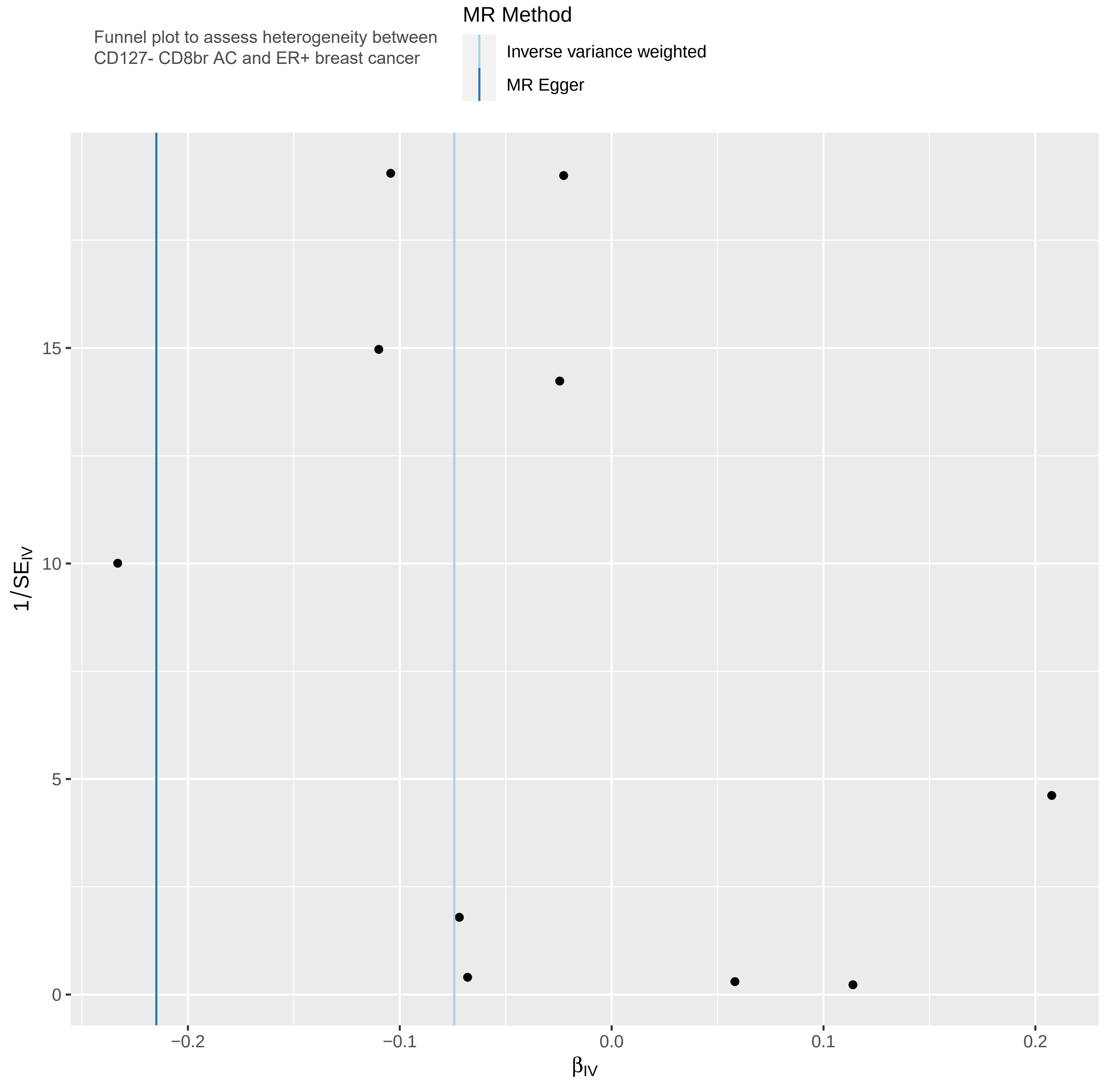
Funnel plot to assess heterogeneity between
CD127- CD8br AC and ER+ breast cancer
MR Method
Inverse variance weighted
MR Egger
1/SE$_{IV}$
β_{IV}
0
5
10
15
−0.2
−0.1
0.0
0.1
0.2

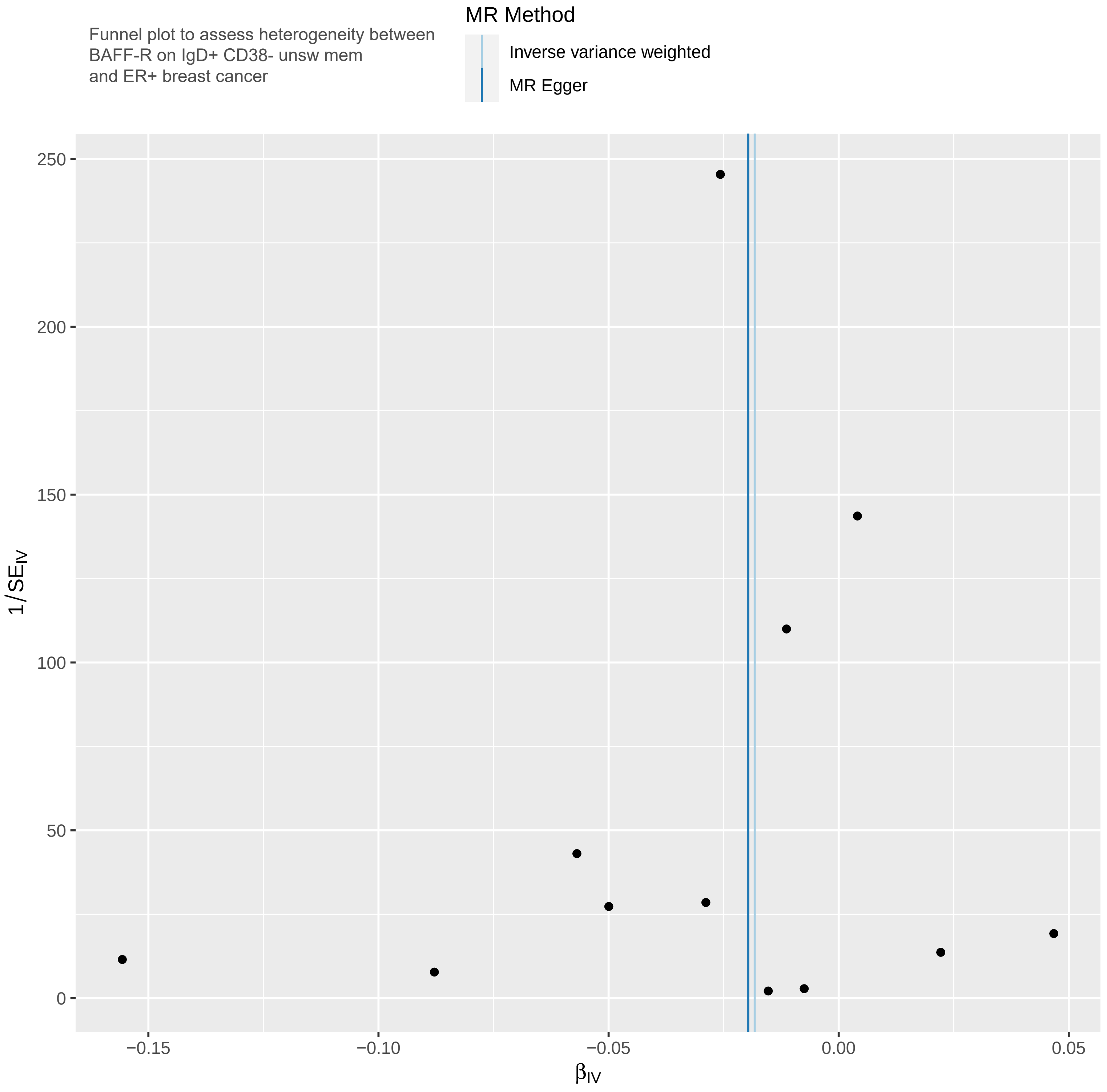
Funnel plot to assess heterogeneity between
BAFF-R on IgD+ CD38- unsw mem
and ER+ breast cancer
MR Method
Inverse variance weighted
MR Egger
250
200
150
100
50
0
1/SE$_{IV}$
−0.15
−0.10
−0.05
0.00
0.05
β$_{IV}$

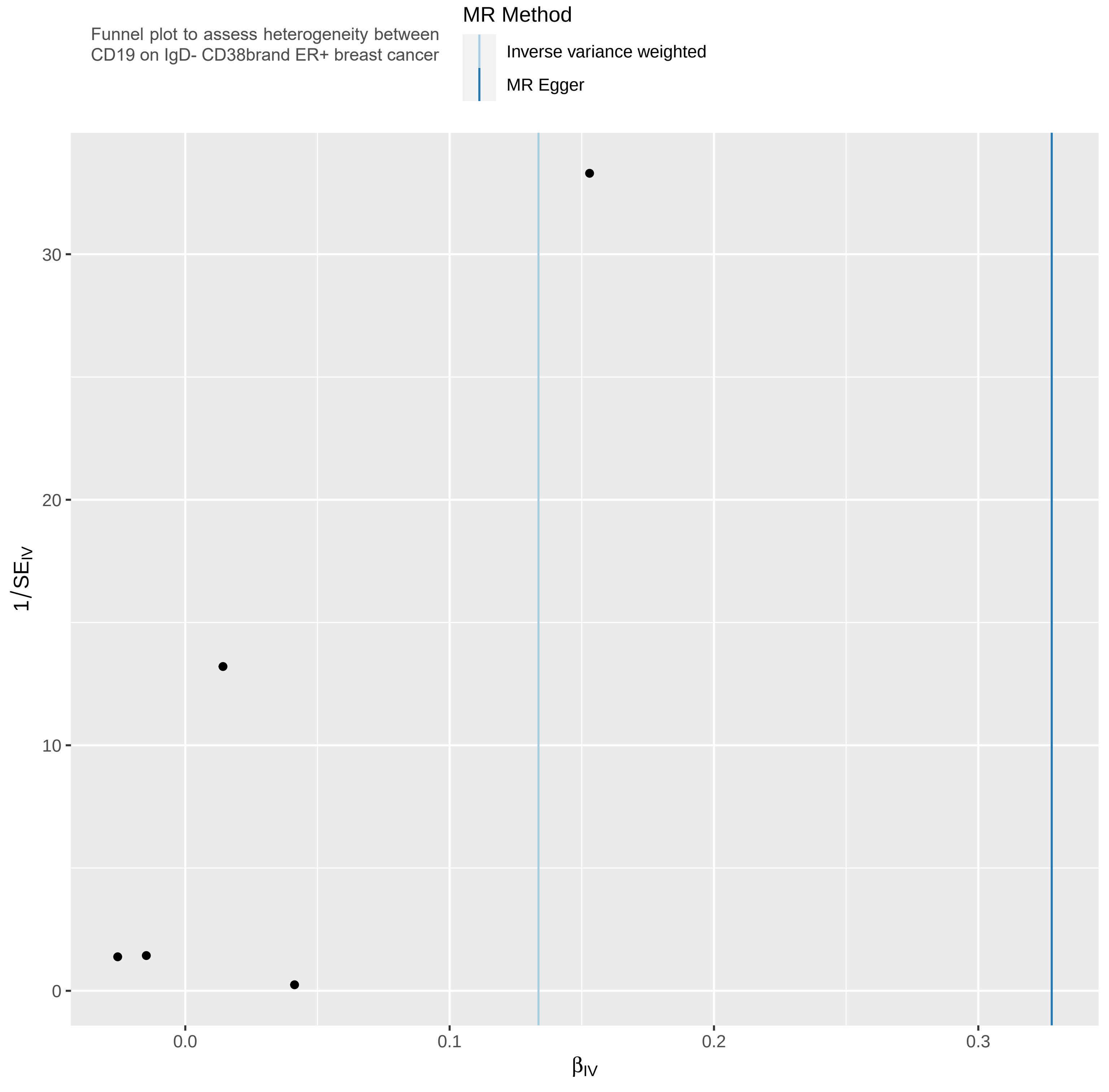

Funnel plot to assess heterogeneity between
CD19 on IgD- CD38brand ER+ breast cancer
MR Method
Inverse variance weighted
MR Egger
30
20
10
0
$1/SE_{IV}$
0.0
0.1
0.2
0.3
β_{IV}

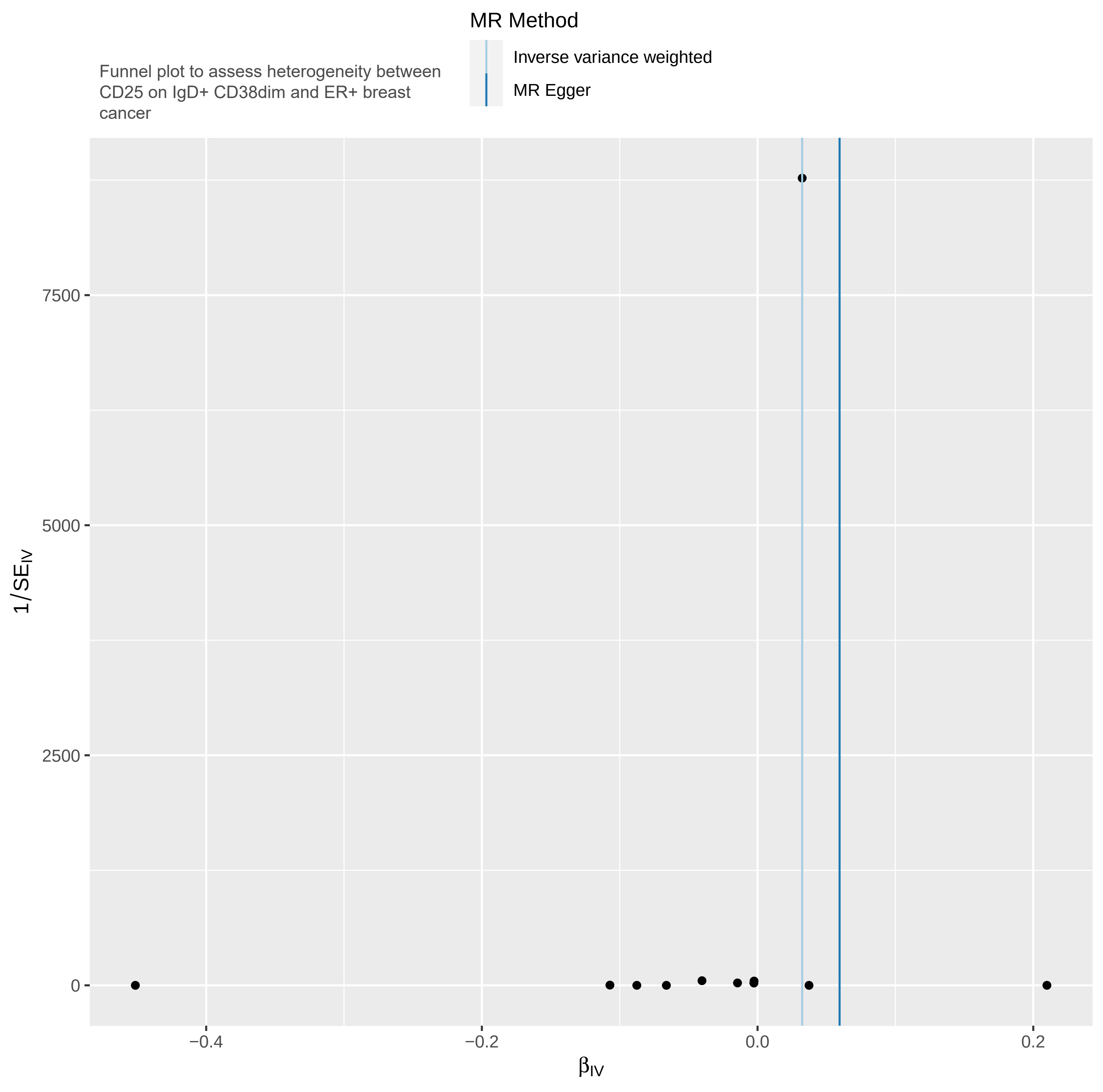
MR Method
Inverse variance weighted
MR Egger
Funnel plot to assess heterogeneity between CD25 on IgD+ CD38dim and ER+ breast cancer
7500
5000
2500
0
1/SE_IV
−0.4
−0.2
0.0
0.2
β_IV

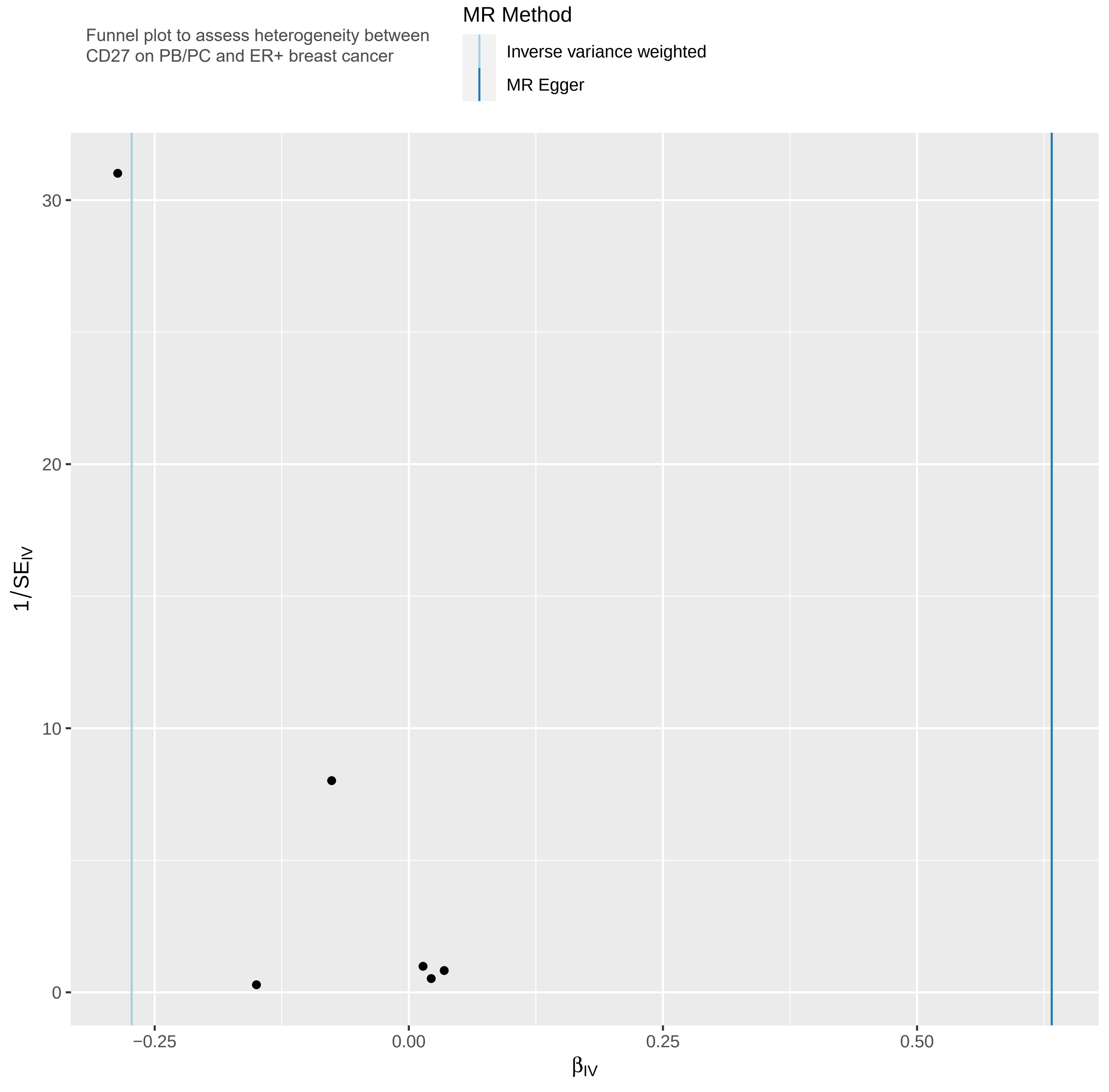
Funnel plot to assess heterogeneity between
CD27 on PB/PC and ER+ breast cancer
MR Method
Inverse variance weighted
MR Egger
30
20
10
0
1/SE$_{IV}$
−0.25
0.00
0.25
0.50
β$_{IV}$

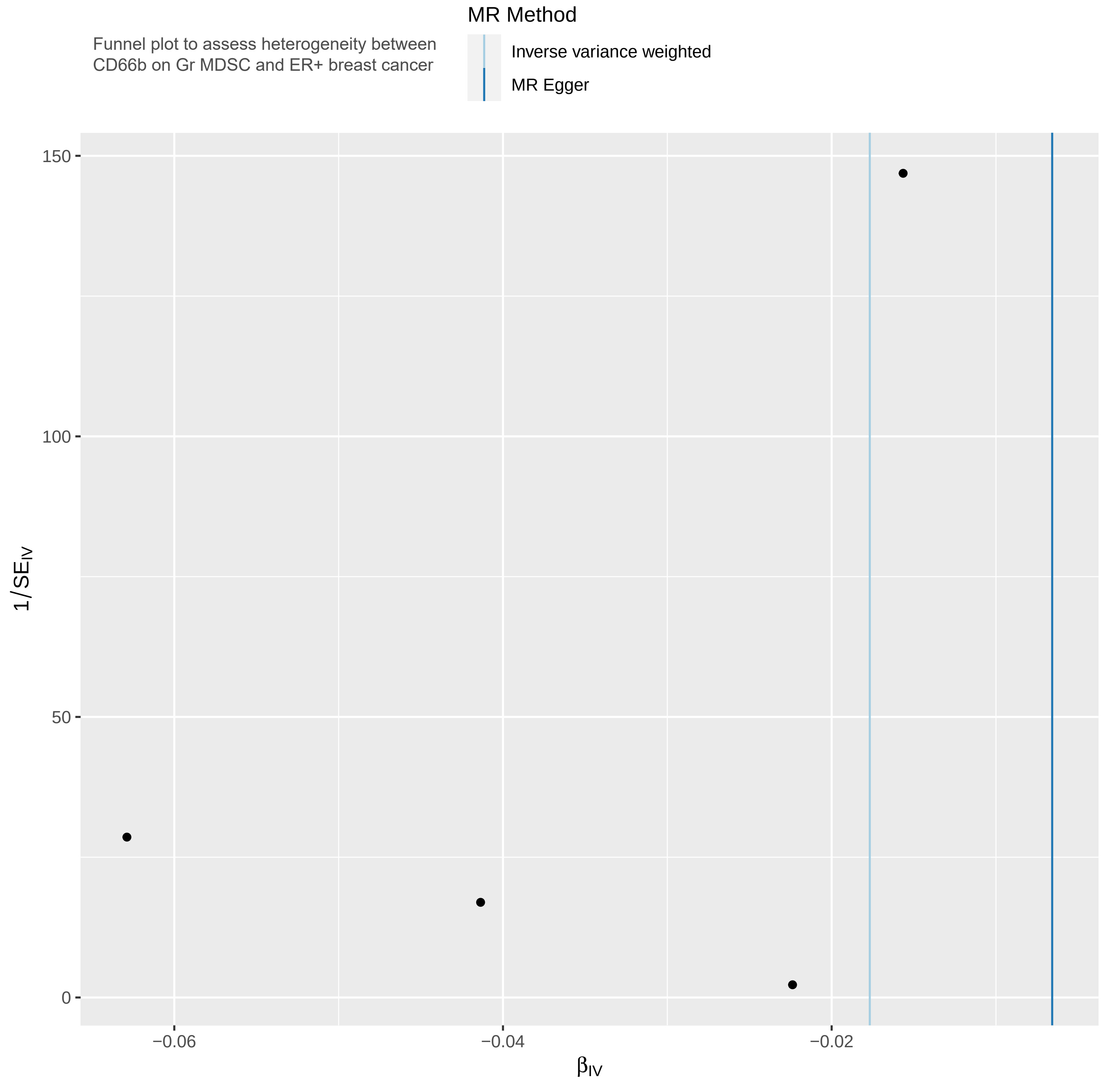

Funnel plot to assess heterogeneity between
CD66b on Gr MDSC and ER+ breast cancer
MR Method
Inverse variance weighted
MR Egger
150
100
50
0
−0.06
−0.04
−0.02
$1/SE_{IV}$
β_{IV}

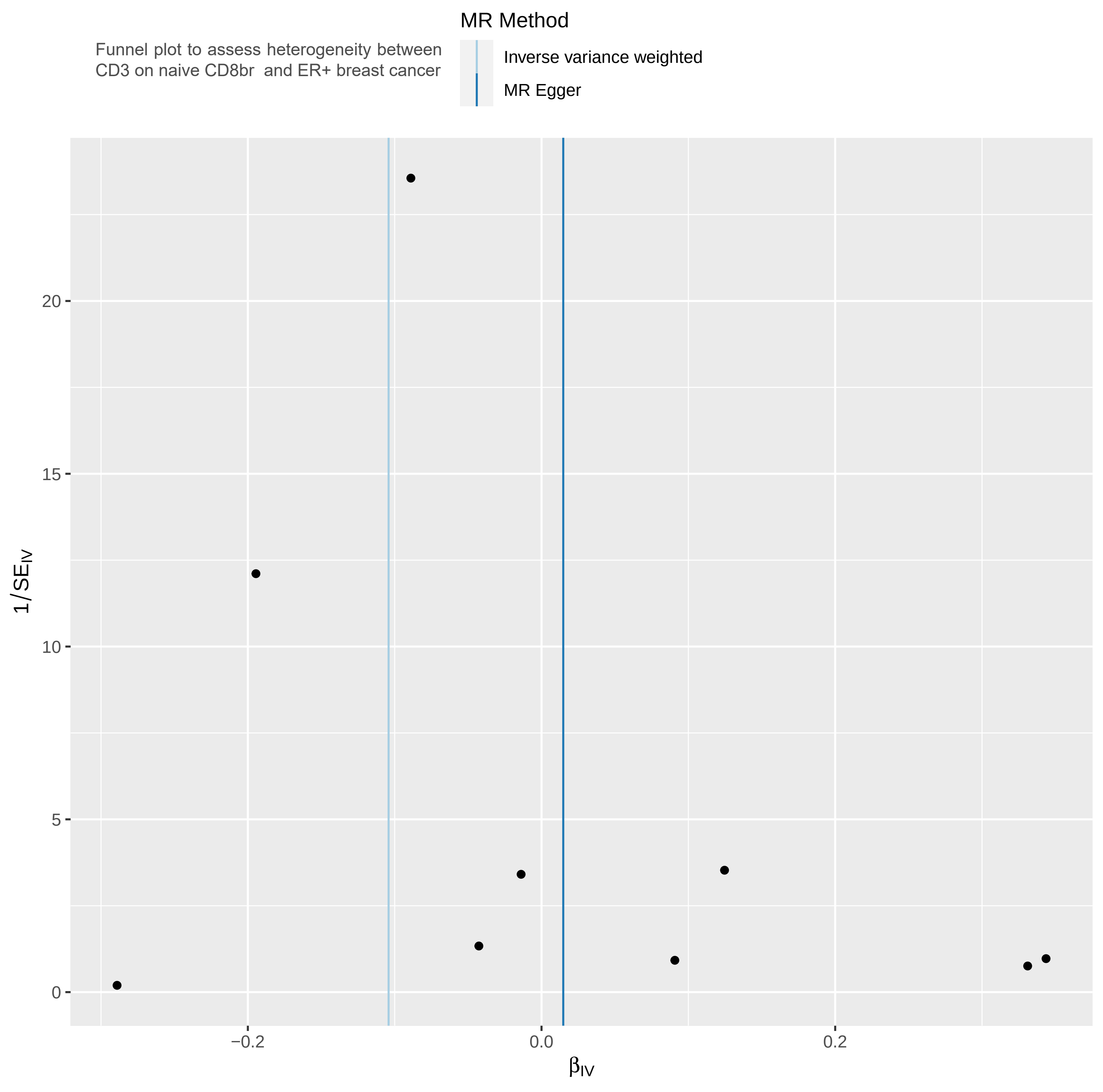

Funnel plot to assess heterogeneity between
CD3 on naive CD8br and ER+ breast cancer
MR Method
Inverse variance weighted
MR Egger
20
15
10
5
0
1/SE_IV
−0.2
0.0
0.2
β_IV

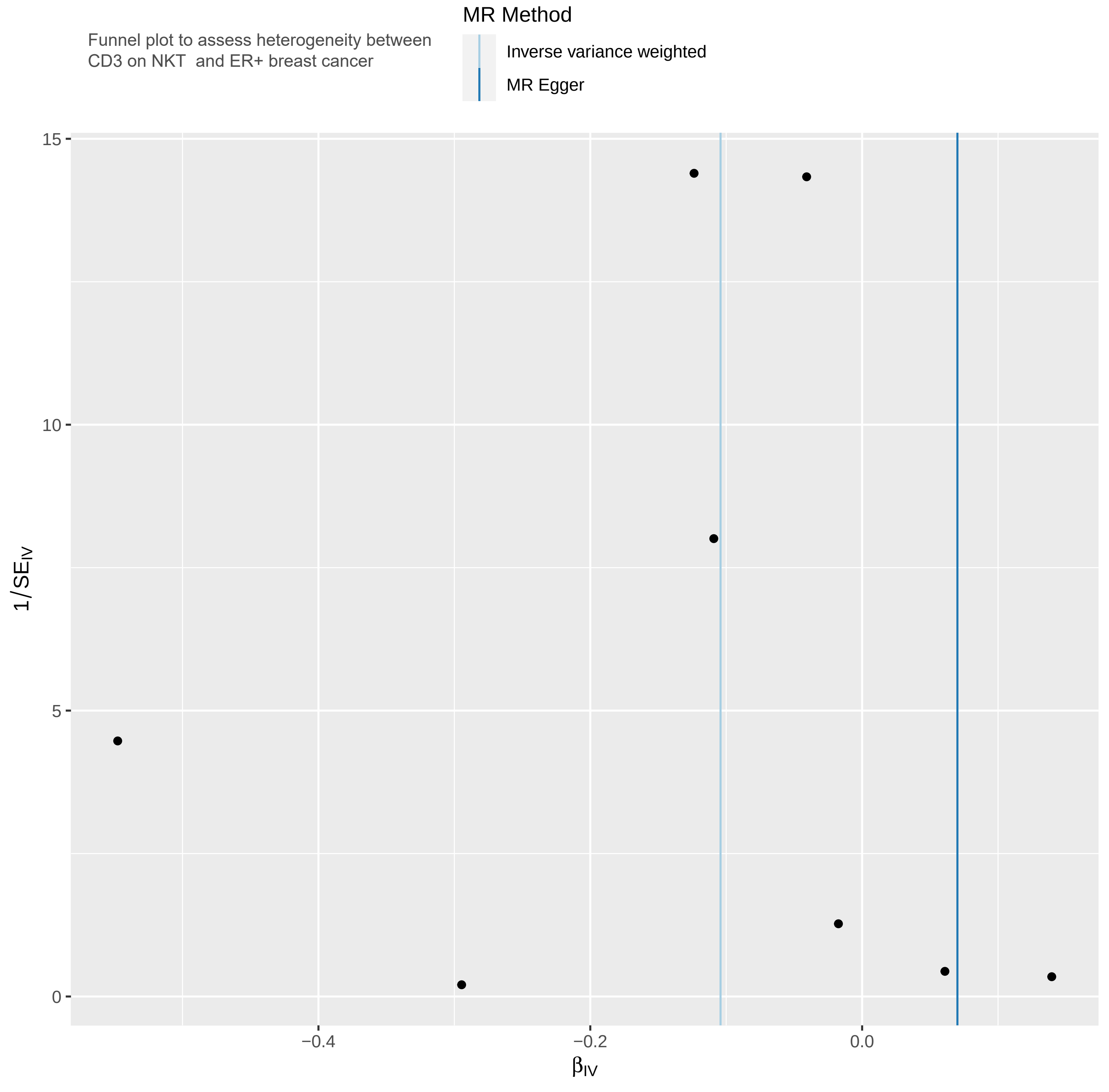
Funnel plot to assess heterogeneity between
CD3 on NKT and ER+ breast cancer
MR Method
Inverse variance weighted
MR Egger
15
10
5
0
1/SE_IV
−0.4
−0.2
0.0
β_IV

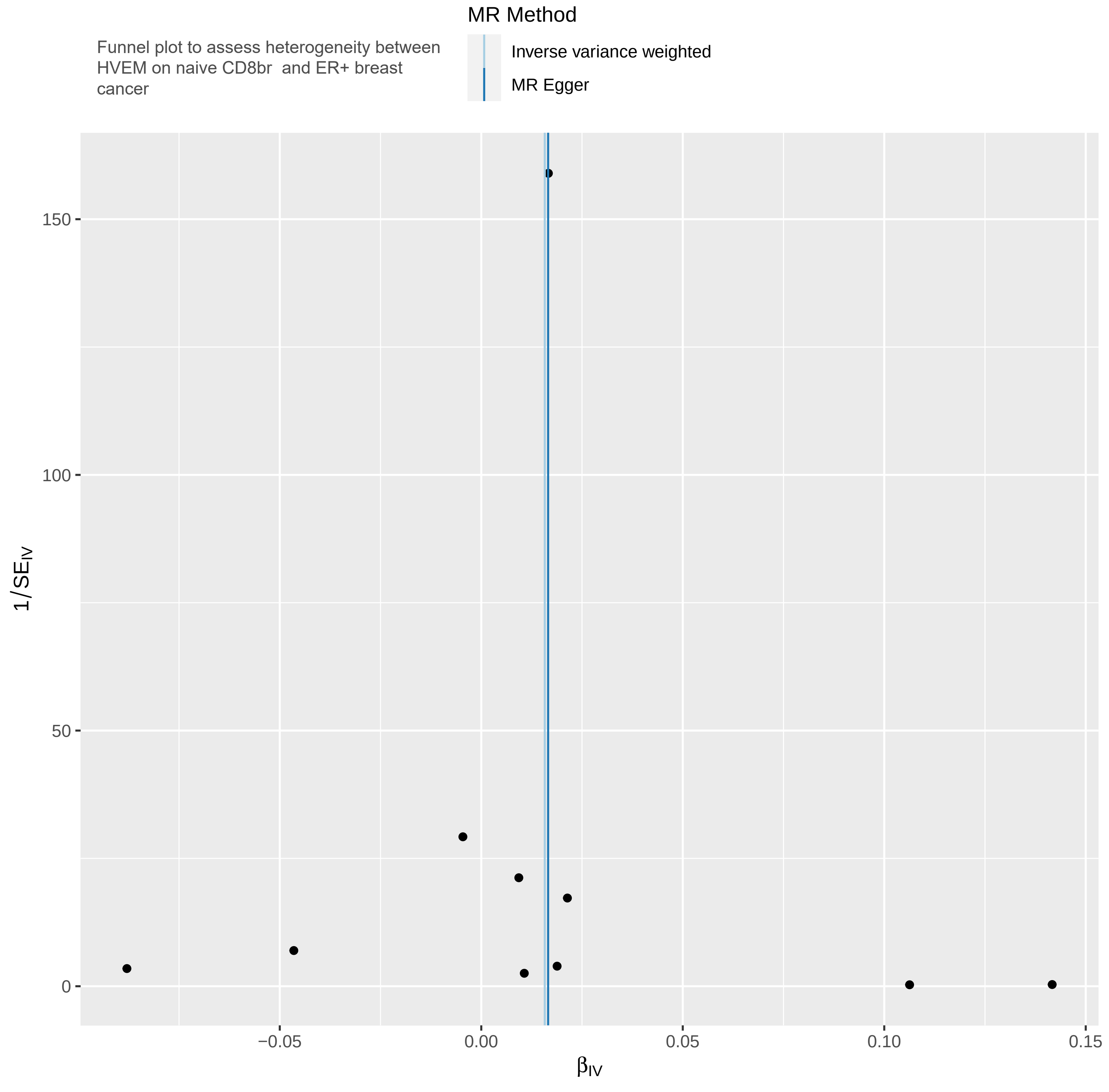

Funnel plot to assess heterogeneity between HVEM on naive CD8br and ER+ breast cancer
MR Method
Inverse variance weighted
MR Egger
150
100
50
0
1/SE_IV
−0.05
0.00
0.05
0.10
0.15
β_IV

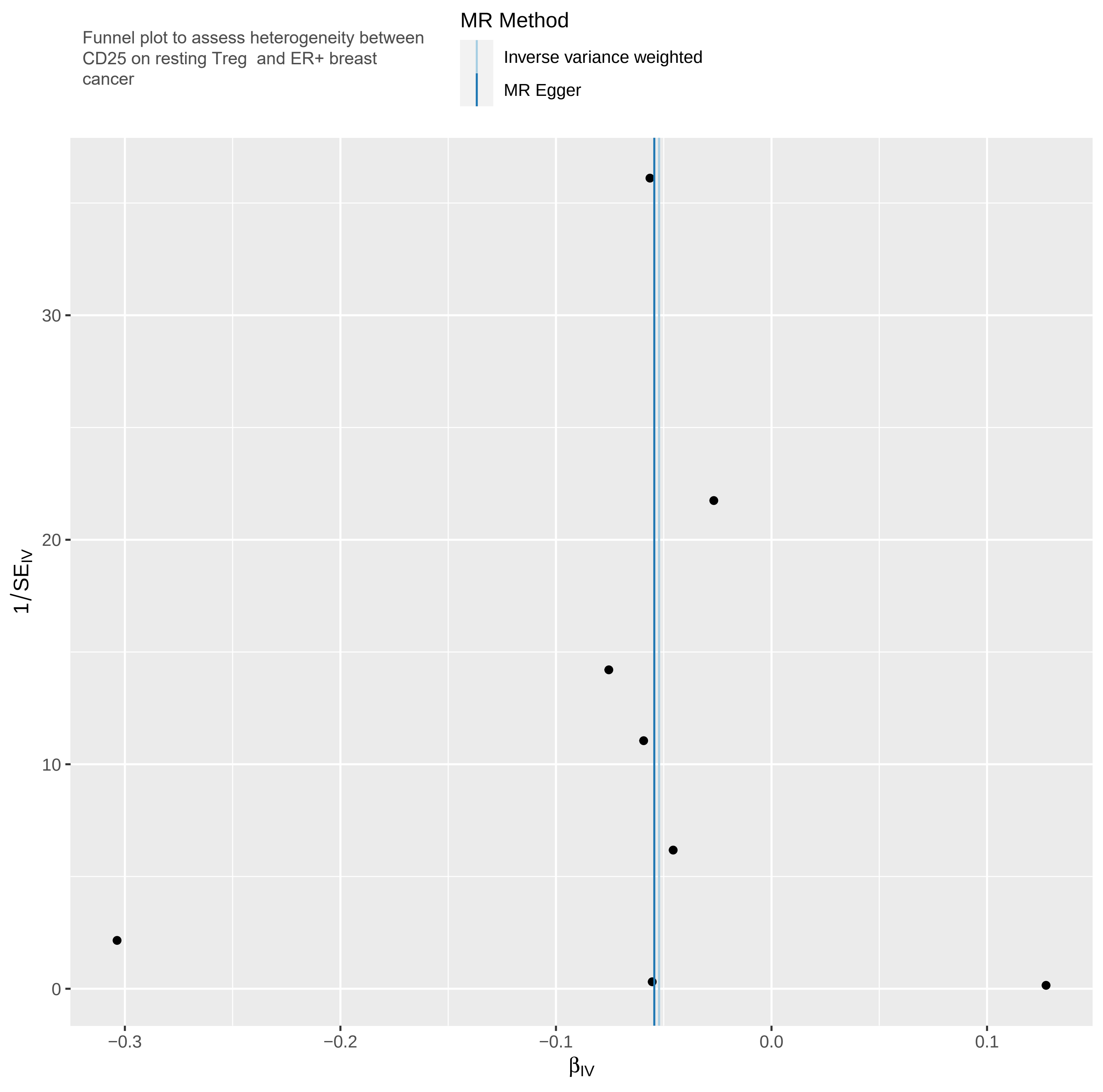
Funnel plot to assess heterogeneity between CD25 on resting Treg and ER+ breast cancer
MR Method
Inverse variance weighted
MR Egger
30
20
10
0
1/SE$_{IV}$
−0.3
−0.2
−0.1
0.0
0.1
β$_{IV}$

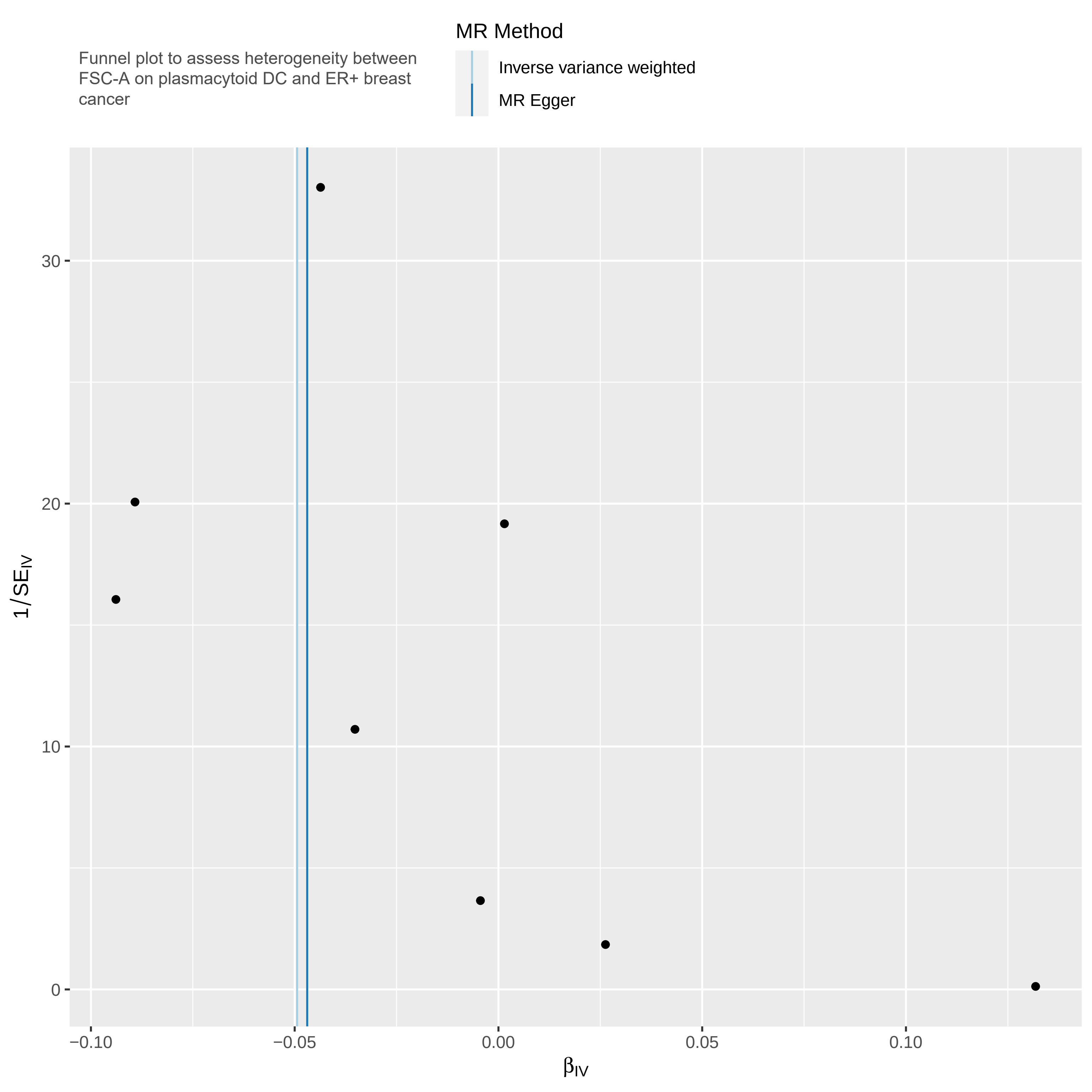
Funnel plot to assess heterogeneity between FSC-A on plasmacytoid DC and ER+ breast cancer
MR Method
Inverse variance weighted
MR Egger
30
20
10
0
1/SE_IV
−0.10
−0.05
0.00
0.05
0.10
β_IV

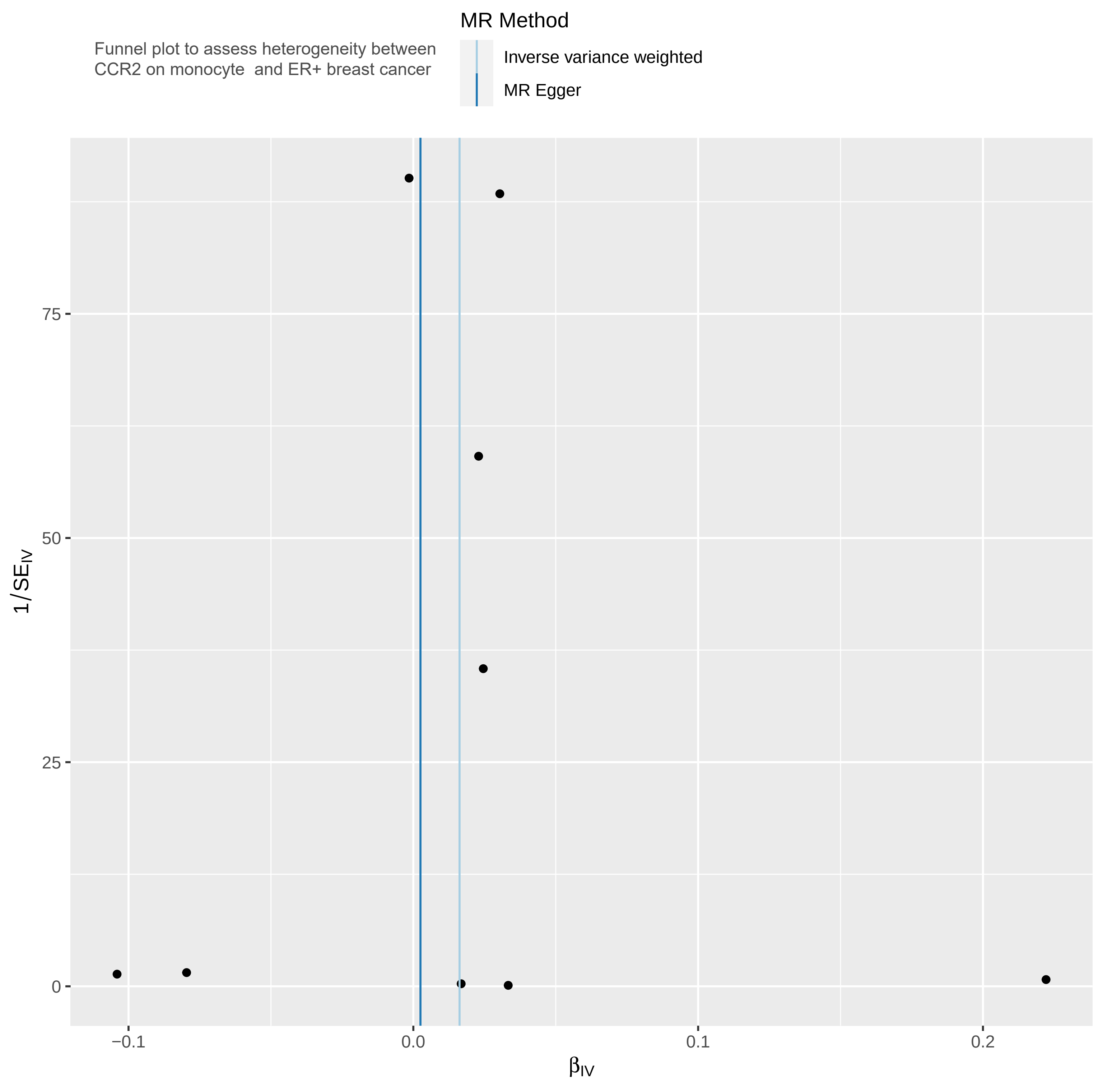
Funnel plot to assess heterogeneity between
CCR2 on monocyte and ER+ breast cancer
MR Method
Inverse variance weighted
MR Egger
75
50
25
0
1/SE_IV
−0.1
0.0
0.1
0.2
β_IV

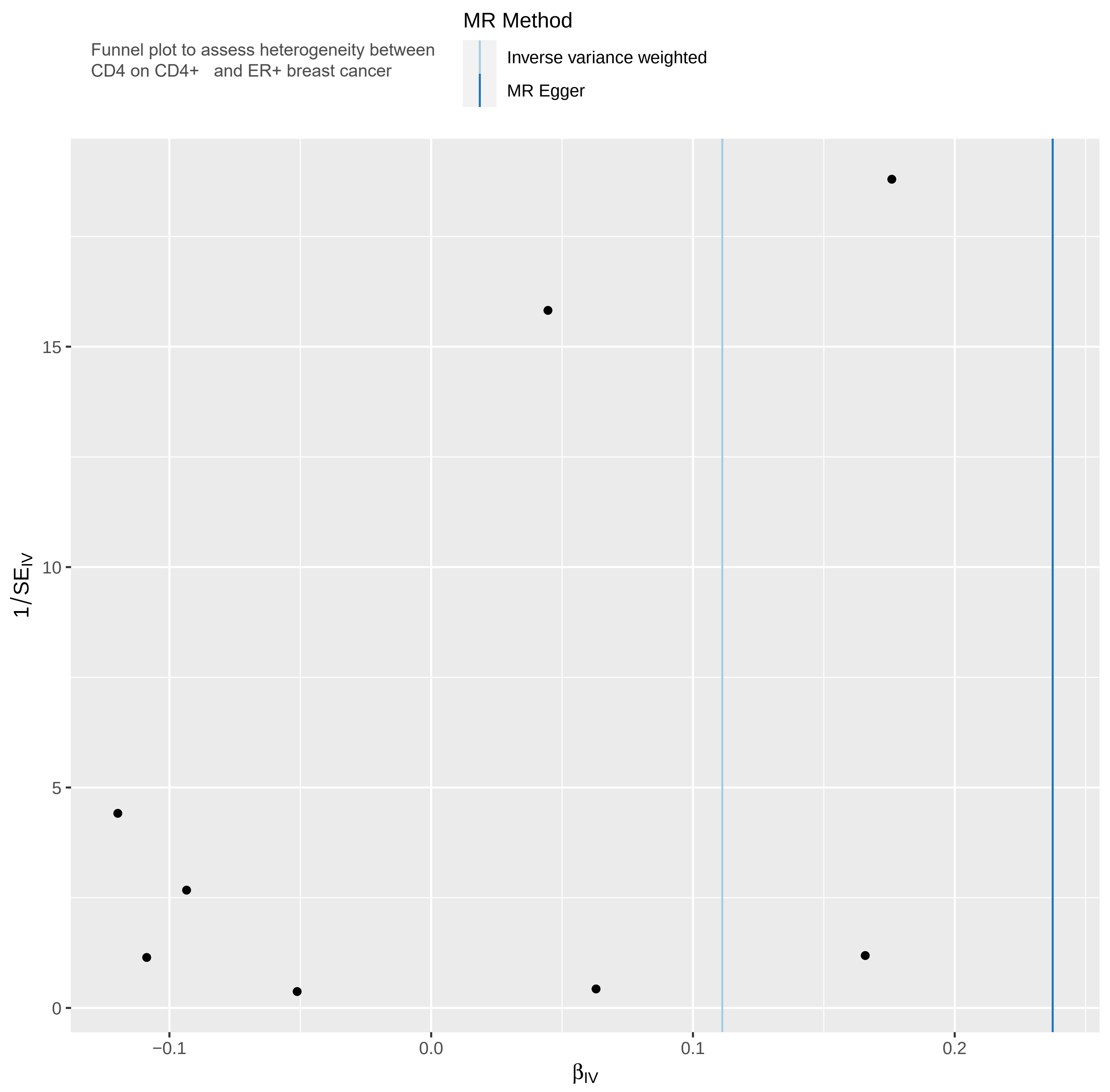
Funnel plot to assess heterogeneity between
CD4 on CD4+ and ER+ breast cancer
MR Method
Inverse variance weighted
MR Egger
1/SE$_{IV}$
0
5
10
15
β$_{IV}$
−0.1
0.0
0.1
0.2

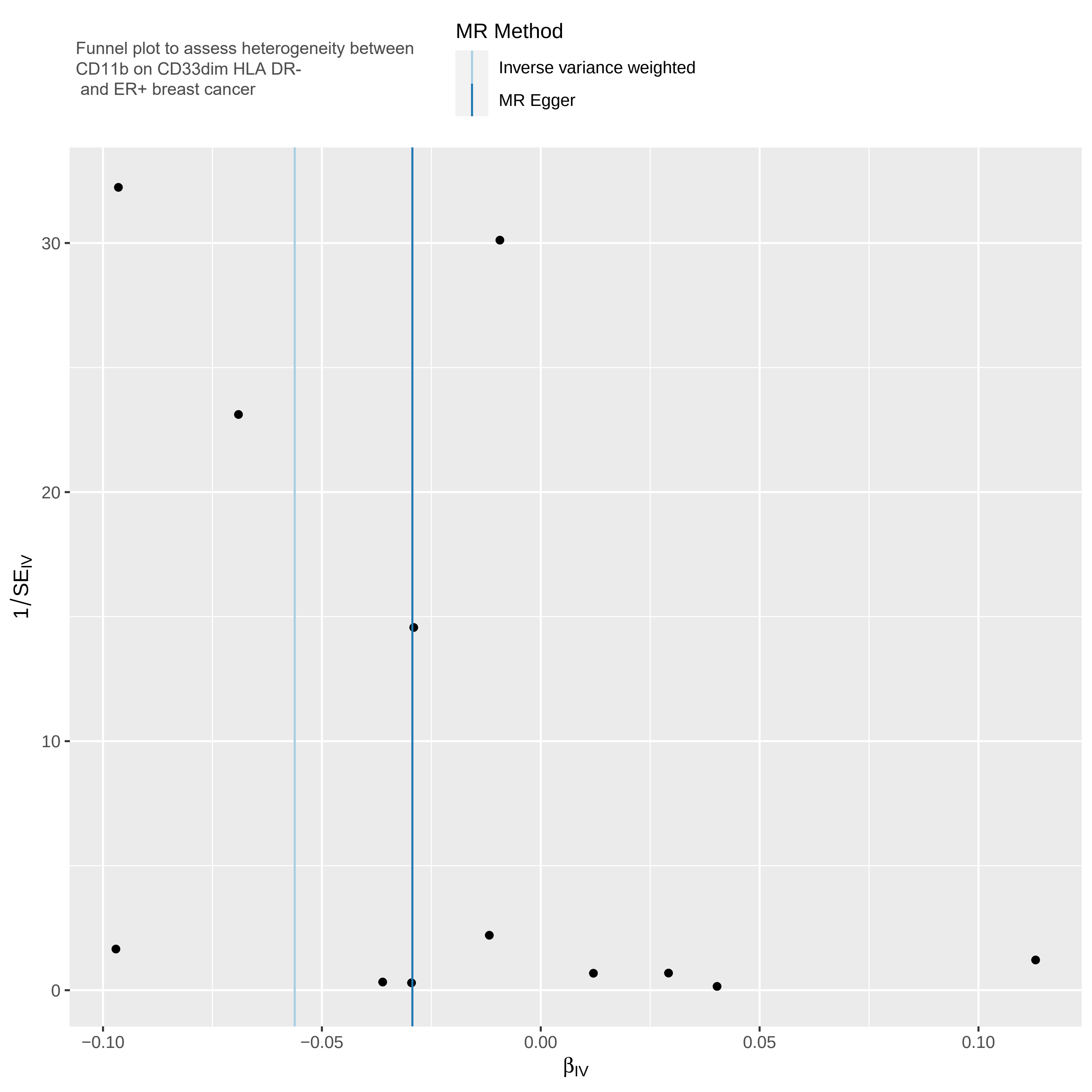
Funnel plot to assess heterogeneity between
CD11b on CD33dim HLA DR-
and ER+ breast cancer
MR Method
Inverse variance weighted
MR Egger
30
20
10
0
−0.10
−0.05
0.00
0.05
0.10
$1/SE_{IV}$
β_{IV}

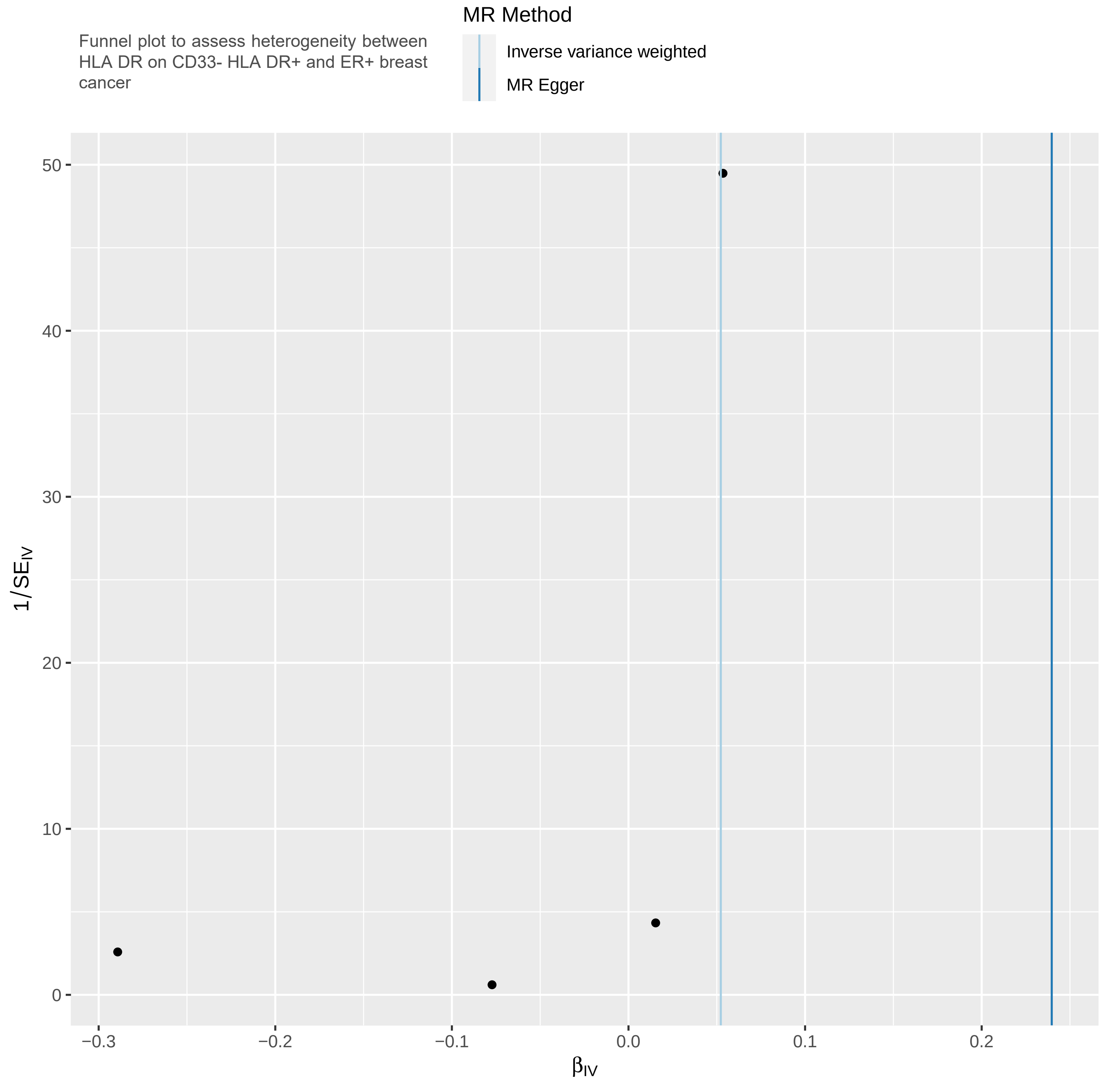

Funnel plot to assess heterogeneity between HLA DR on CD33- HLA DR+ and ER+ breast cancer
MR Method
Inverse variance weighted
MR Egger
1/SEIV
βIV
50
40
30
20
10
0
−0.3
−0.2
−0.1
0.0
0.1
0.2

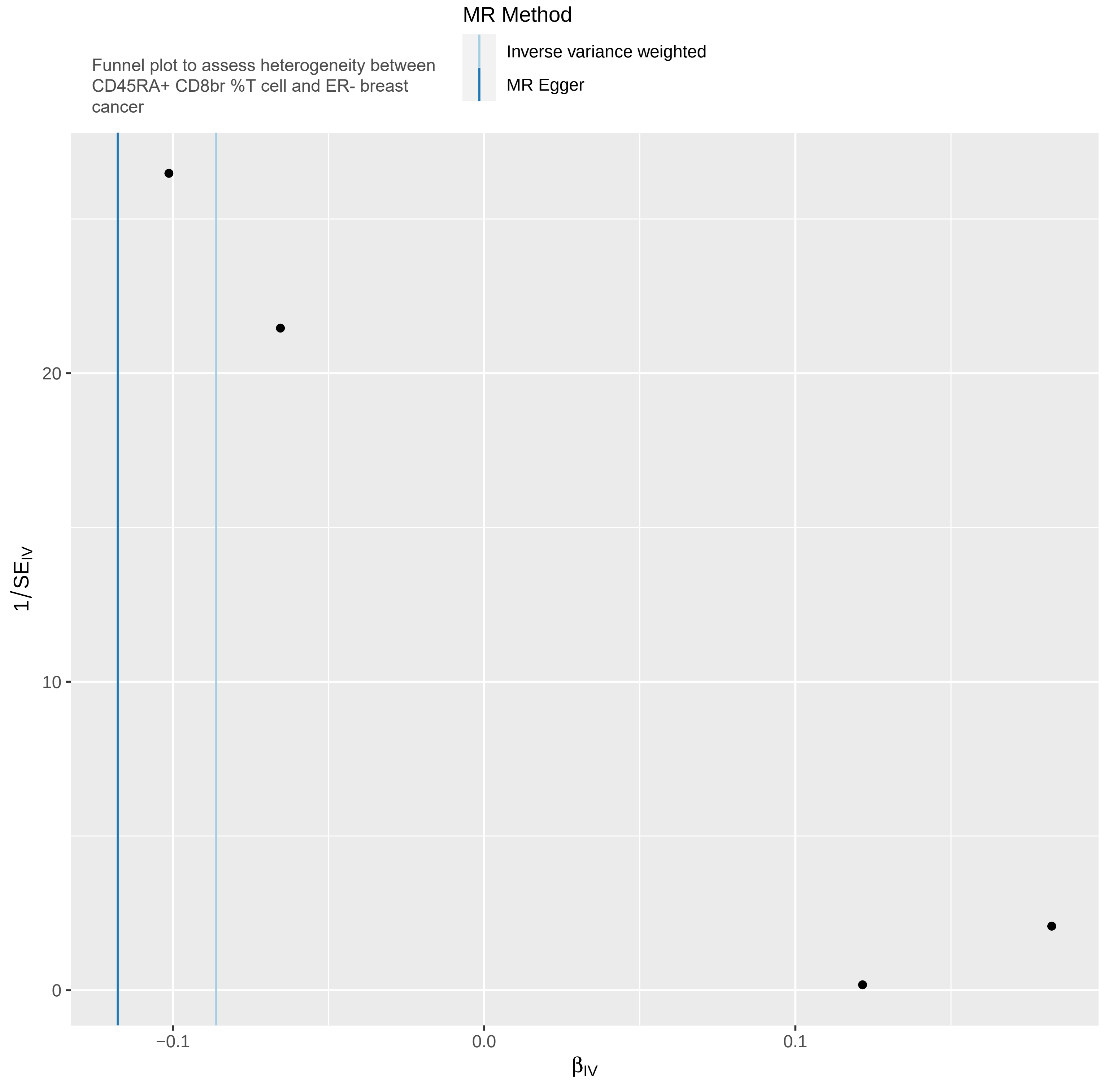

MR Method
Inverse variance weighted
MR Egger
Funnel plot to assess heterogeneity between CD45RA+ CD8br %T cell and ER- breast cancer
20
10
0
1/SE_IV
−0.1
0.0
0.1
β_IV

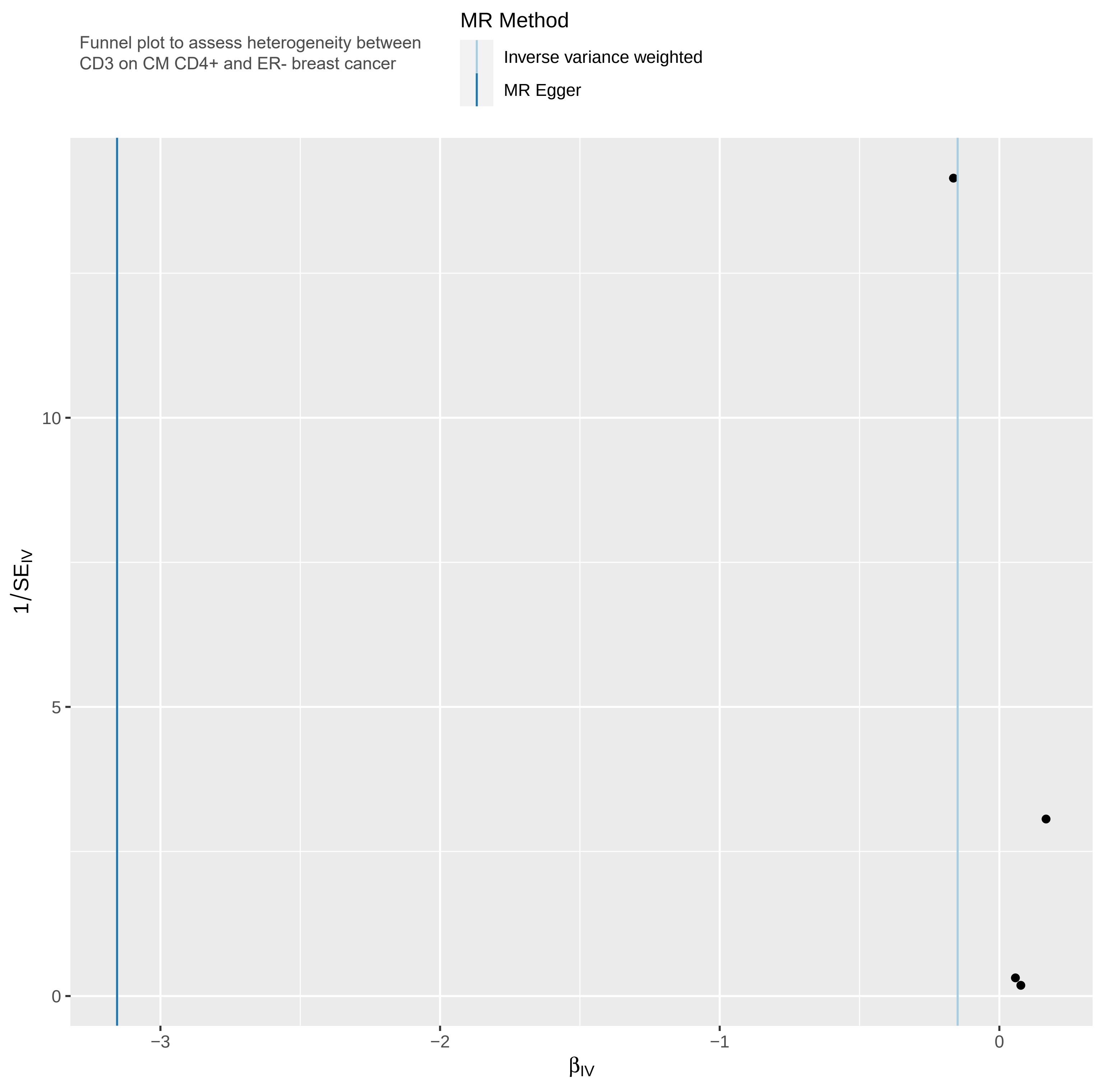

Funnel plot to assess heterogeneity between
CD3 on CM CD4+ and ER- breast cancer
MR Method
Inverse variance weighted
MR Egger
10
5
0
1/SE$_{IV}$
−3
−2
−1
0
β$_{IV}$

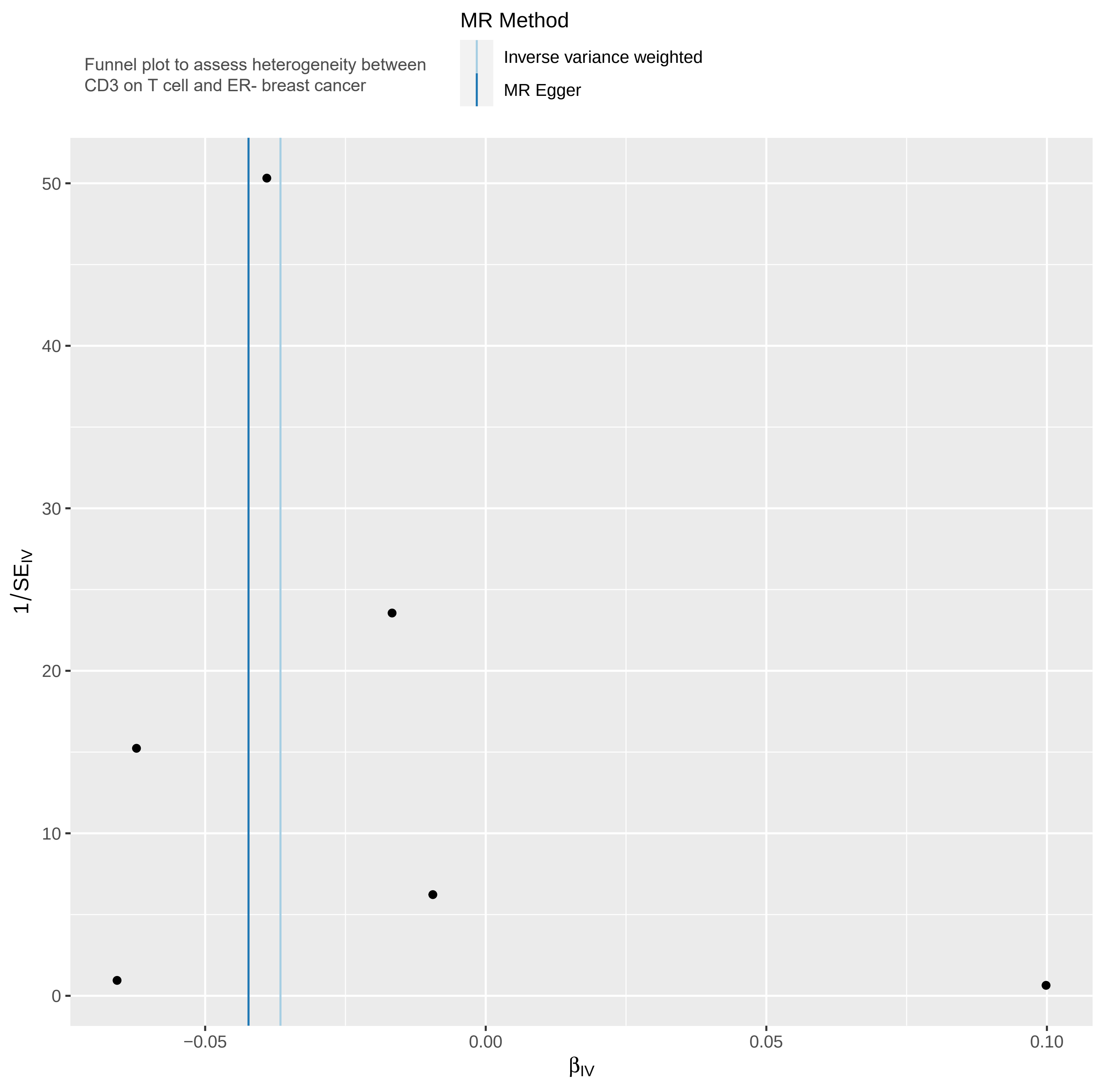

Funnel plot to assess heterogeneity between
CD3 on T cell and ER- breast cancer
MR Method
Inverse variance weighted
MR Egger
50
40
30
20
10
0
1/SE_IV
−0.05
0.00
0.05
0.10
β_IV

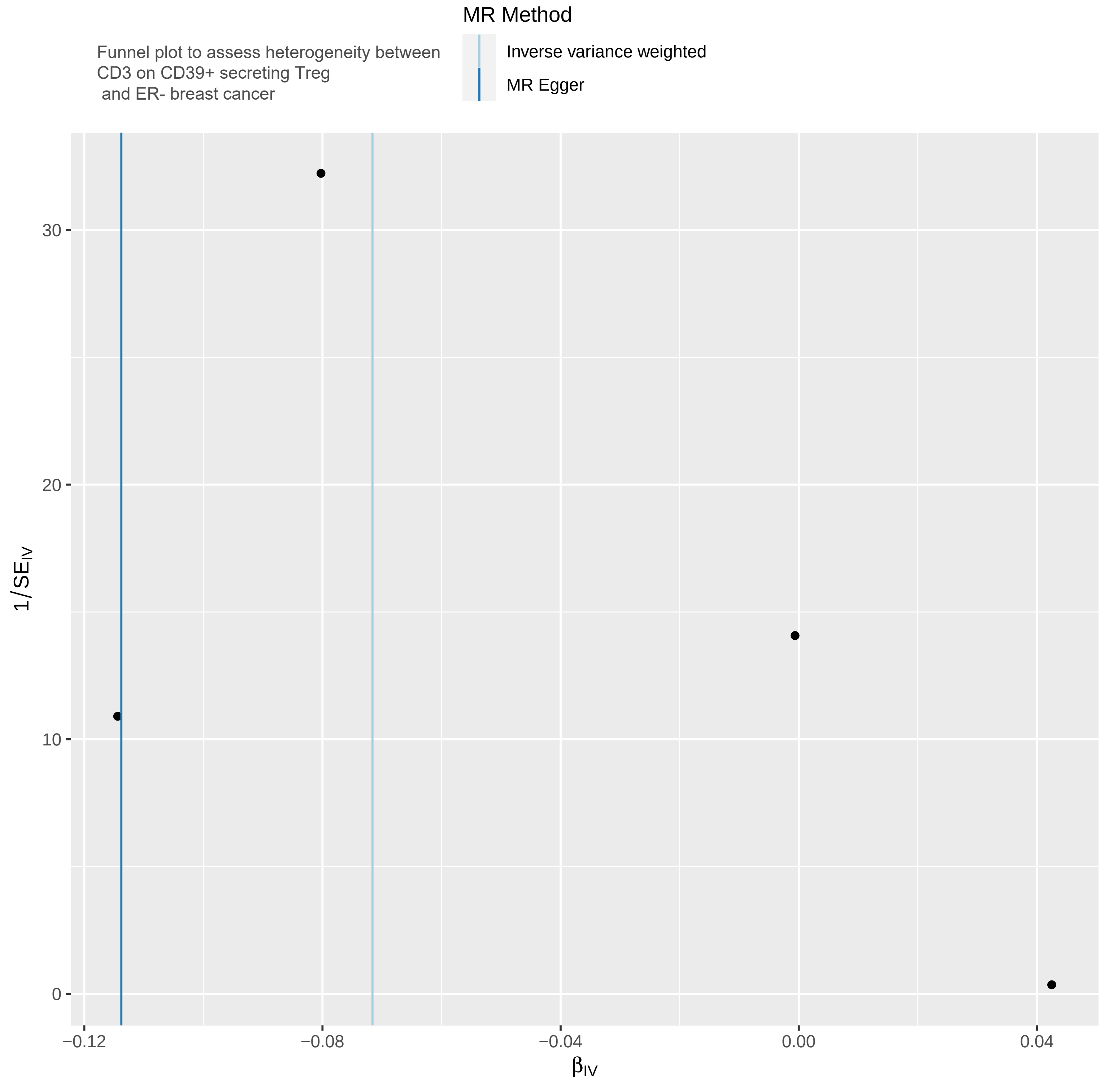
Funnel plot to assess heterogeneity between
CD3 on CD39+ secreting Treg
and ER- breast cancer
MR Method
Inverse variance weighted
MR Egger
1/SE$_{IV}$
30
20
10
0
−0.12
−0.08
−0.04
0.00
0.04
β$_{IV}$

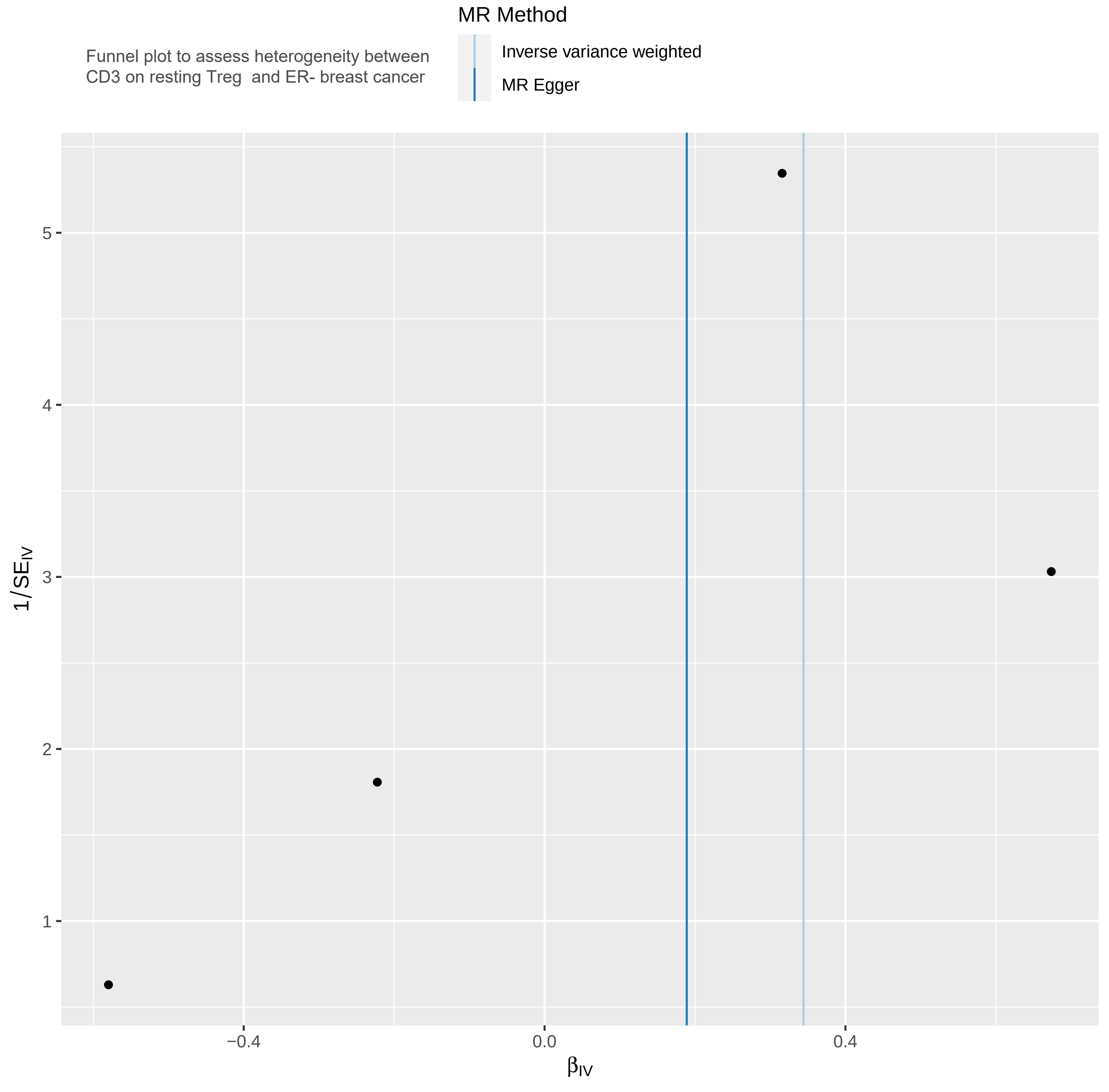

Funnel plot to assess heterogeneity between
CD3 on resting Treg and ER- breast cancer
MR Method
Inverse variance weighted
MR Egger
5
4
3
2
1
1/SE_IV
−0.4
0.0
0.4
β_IV

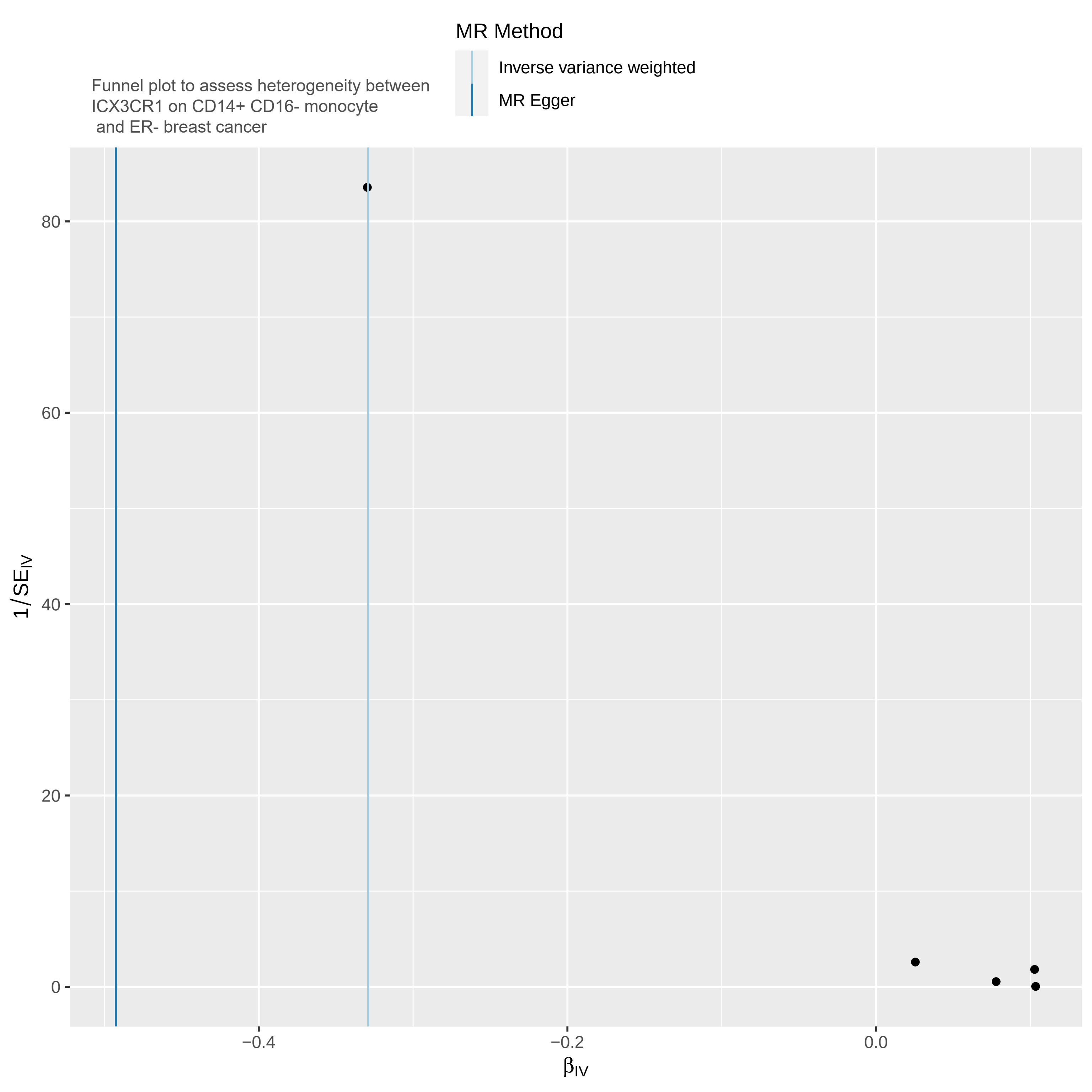

MR Method
Inverse variance weighted
MR Egger
Funnel plot to assess heterogeneity between
ICX3CR1 on CD14+ CD16- monocyte
and ER- breast cancer
80
60
40
20
0
1/SE_IV
−0.4
−0.2
0.0
β_IV

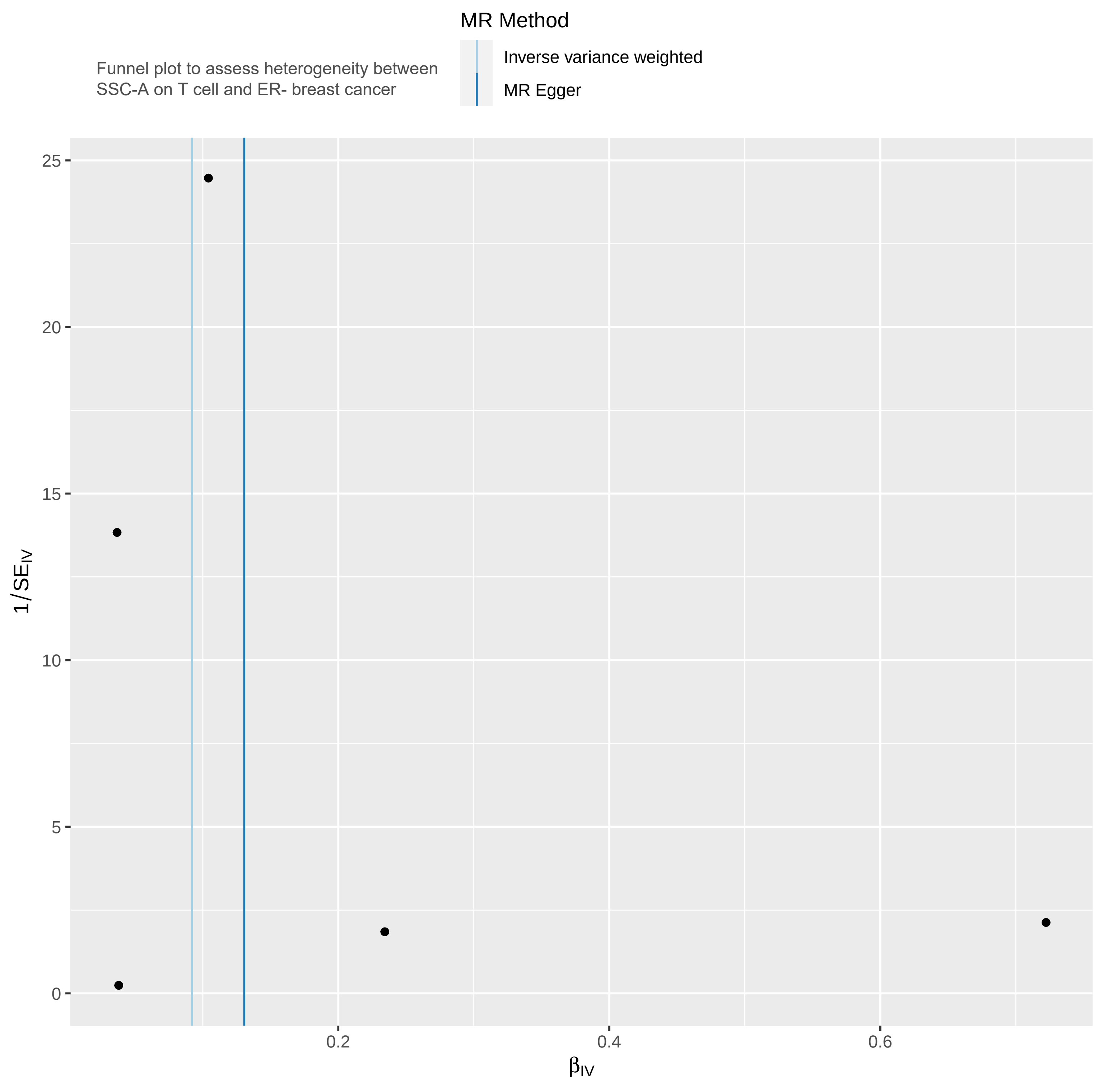
MR Method
Inverse variance weighted
MR Egger
Funnel plot to assess heterogeneity between
SSC-A on T cell and ER- breast cancer
25
20
15
10
5
0
$1/SE_{IV}$
0.2
0.4
0.6
β_{IV}
